# Application of Palladium-Mediated ^18^F-Fluorination to PET Radiotracer Development: Overcoming Hurdles to Translation

**DOI:** 10.1371/journal.pone.0059187

**Published:** 2013-03-12

**Authors:** Adam S. Kamlet, Constanze N. Neumann, Eunsung Lee, Stephen M. Carlin, Christian K. Moseley, Nickeisha Stephenson, Jacob M. Hooker, Tobias Ritter

**Affiliations:** 1 Department of Chemistry and Chemical Biology, Harvard University, Cambridge, Massachusetts, United States of America; 2 Athinoula A. Martinos Center for Biomedical Imaging, Massachusetts General Hospital and Harvard Medical School, Charlestown, Massachusetts, United States of America; 3 Division of Nuclear Medicine and Molecular Imaging, Department of Radiology, Massachusetts General Hospital, Boston, Massachusetts, United States of America; Northwestern University, United States of America

## Abstract

New chemistry methods for the synthesis of radiolabeled small molecules have the potential to impact clinical positron emission tomography (PET) imaging, if they can be successfully translated. However, progression of modern reactions from the stage of synthetic chemistry development to the preparation of radiotracer doses ready for use in human PET imaging is challenging and rare. Here we describe the process of and the successful translation of a modern palladium-mediated fluorination reaction to non-human primate (NHP) baboon PET imaging–an important milestone on the path to human PET imaging. The method, which transforms [^18^F]fluoride into an electrophilic fluorination reagent, provides access to aryl–^18^F bonds that would be challenging to synthesize via conventional radiochemistry methods.

## Introduction

Positron emission tomography (PET) is a non-invasive imaging technique used to characterize and diagnose disease [1], [2]. Historically, PET imaging has been limited by chemistry. There are few convenient and efficient methods to incorporate positron-emitting isotopes into small molecules of imaging interest. Several positron-emitting isotopes are used for PET imaging, including carbon-11, nitrogen-13, oxygen-15, and copper-64, but due to its suitable half-life for synthesis and imaging, fluorine-18 (^18^F) is the most common and most relevant isotope for clinical PET [3], [4]. Additionally, fluorine is present in a variety of biologically active molecules and pharmaceuticals [5], [6]. However, synthesis with^18^F is difficult due to time restrictions (half-life of ^18^F: 110 min) and mass restrictions (scale of synthesis: nanomoles), among others [7]. Thus the general chemical challenges associated with carbon–fluorine bond formation [8], [9], [10] are exacerbated for ^18^F-radiotracer synthesis. Modern methods for the construction of carbon–fluorine bonds are changing the landscape of molecules considered viable for PET [9]. The new chemical methods hold the unrealized potential of changing radiotracer design and development. A critical, non-trivial step is the development of modern fluorination methods from the stage of synthetic chemistry to the preparation of radiotracer doses ready for *in vivo* PET imaging. Here we describe the first successful development of a modern palladium-mediated fluorination reaction to be used for non-human primate (NHP) PET imaging–an important milestone on the path to human PET imaging.

The imaging candidates reported here were chosen for structural motifs that are representative of drug-like small molecules, and were intended to highlight the utility of transition-metal-mediated carbon–fluorine bond formation to overcome chemical challenges associated with ^18^F chemistry. The challenges include: 1) ^18^F radiochemistry requires fast reaction rates because the concentration of the limiting reagent (fluoride) is small (µM) compared to typical synthetic reactions (mM to M). Slow reaction rates are often overcome by elevating reaction temperatures, which can lead to side reactions and product decomposition, especially for complex small molecules. 2) ^18^F is available in nucleophilic and electrophilic forms, but only the nucleophilic form, [^18^F]fluoride, is readily available and can be made in high specific activity [7], [11]. Specific activity is a measurement of the amount of radioactivity per molar amount of sample. High specific activity, though not necessary for all PET applications, is important for imaging biological targets of low concentration, like imaging of neurotransmitter receptors in the brain [4]. For pharmacokinetic measurements, specific activity is less critical. 3) [^18^F]Fluoride is created by the proton bombardment of [^18^O]H_2_O. While removal of the bulk water is common for synthesis with ^18^F, the large hydration energy [12], [13], [14] and the small amount of fluoride prevents rigorous drying. Hydrated fluoride is less nucleophilic than dry fluoride and therefore cannot be used in many nucleophilic fluorination reactions. Most recently developed fluorination reactions do not meet or have yet to meet one or more of the above considerations. For example, reactions that require dry solvent or long reaction times are difficult to transition to radiochemistry, even if yields are high for the fluorination reactions with ^19^F, the natural isotopologue.

Once chemistry with ^18^F is feasible (i.e. a fluorination reaction can be accomplished with ^18^F, and a radiochemical yield (RCY) can be determined), the reaction still may not lead to a practical synthesis method for molecules useful for PET imaging experiments [15]. Most importantly, radiochemistry techniques must allow for efficient purification due to time constraints. The formation of constitutional isomers that are difficult-to-separate must be minimized during synthesis, and starting materials as well as byproducts must be removable from the desired sample. In particular hydro-defluoro and halo-defluoro (halo = chloro, bromo, iodo) can be difficult to separate from radiofluorinated material. Final samples must be radiochemically and chemically pure and sterile. Additionally, a substantial quantity of radioactivity must be obtained at the end of synthesis; typically 5–10 mCi per experiment for non-human primates is suitable for imaging [1]. Automated robotic procedures are utilized to handle this amount of radioactivity.

**Figure 1:**
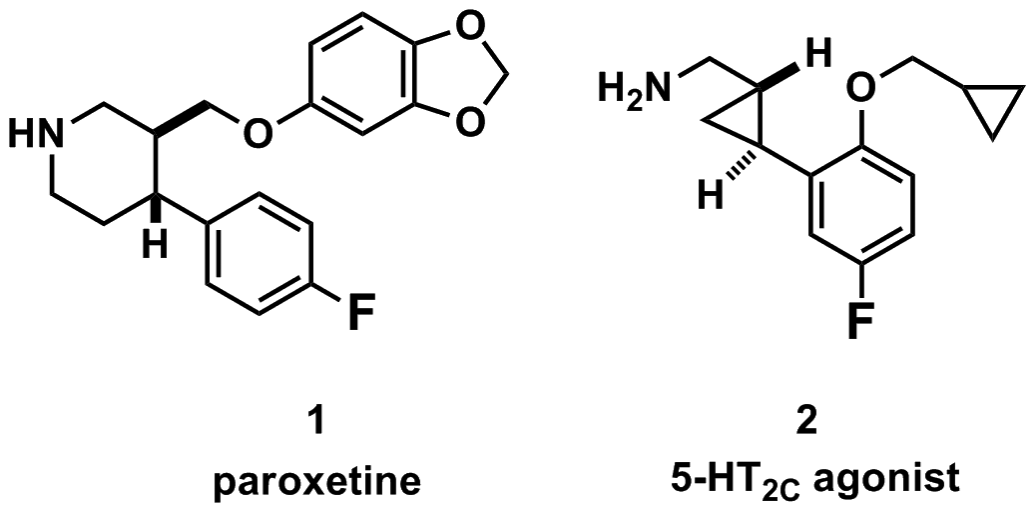
Two small molecule aryl fluorides chosen for radiofluorination and non-human primate PET imaging presented here.

Two molecules, targeting the serotonin neurotransmitter system, were identified and selected for transitioning our new fluorination method to PET imaging applications [16]. Each molecule represents one example of the two general functional uses of PET imaging in drug discovery: 1) labeling a drug to assess its biodistribution as well as pharmacokinetic and metabolic profile; and 2) labeling a bioactive molecule to evaluate its potential as a radiotracer, a marker of a biological system [17]. For the labeled drug example, paroxetine (**1**), an antidepressant selective serotonin reuptake inhibitor (SSRI) medication with nearly 13 million prescriptions in the US in 2010 [18], was selected (Figure 1). Despite its widespread use, the pharmacokinetic profile and distribution of paroxetine binding in the human brain are unknown. There has been previous interest in labeling paroxetine for PET imaging in humans [19], but only paroxetine derivatives [20], not paroxetine itself, could be labeled with ^18^F, presumably due to lack of appropriate chemistry methods. As a representative molecule for the second class, the 5-HT_2C_ agonist **2** [21] was selected for evaluation as a potential radiotracer for imaging 5-HT_2C_ receptors, an important target in the development of new drugs to treat obesity [22] and a target for which no radiotracer exists (5-HT = 5-hydroxytryptamine = serotonin) [23]. Both molecules would be difficult to radiolabel with ^18^F using conventional fluorination strategies.

We recently reported a conceptually different arene radiofluorination reaction that relies on C–F bond formation at a transition metal center [24]. The novelty of the method consists of [^18^F]fluoride capture to form an electrophilic fluorination reagent that could be used for late-stage fluorination. The development and translation of the method into a reliable, reproducible arene radiofluorination method that can afford radiolabeled molecules on a scale suitable for PET imaging in non-human primates (NHPs) is presented here. The complete method from acquisition of ^18^F, through automated synthesis, purification and formulation for intravenous injection can be performed in less than 100 minutes. The method enabled the first syntheses of **[^18^F]-1** and **[^18^F]-2**, provided material sufficient in quality and quantity for PET brain imaging studies in NHPs, and showcased the ability to obtain ^18^F-labeled molecules in high specific activity for PET imaging in NHP that would be challenging to obtain with conventional fluorination methods. Our results establish that the palladium-mediated fluorination has the potential to serve as a research tool for *in vivo* imaging in drug discovery and radiotracer development.

## Results and Discussion

### Development of an Electrophilic Fluorination Reagent Derived from Fluoride

Radiofluorination with electrophilic ^18^F is challenging because the reagent most often used, [^18^F]fluorine gas ([^18^F]F_2_), is difficult to work with, requires specialized equipment, and is made in low specific activity [3]. To efficiently liberate ^18^F from the target, the sample is purposefully diluted with [^19^F]F_2_. While progress has been made to improve the isotopic enrichment of [^18^F]F_2_ [11], the specific activity of molecules synthesized from [^18^F]F_2_ cannot reach the level of [^18^F]fluoride because only one of the fluorine atoms of [^18^F]F_2_ is ^18^F. Furthermore, radiochemical yield can be at most 50% because only one of the fluorine atoms is transferred, with essentially equal probability. Methods have been devised to synthesize more practical electrophilic radiofluorination reagents [25], [26], [27], but they have all been derived ultimately from [^18^F]F_2_. Nucleophilic [^18^F]fluoride is a more practical source of ^18^F, because it is widely available and produced without purposeful dilution with ^19^F. However, nucleophilic fluorination reactions are challenging to transition to radiochemistry given hydrated fluoride’s limited nucleophilicity. A practical electrophilic radiofluorination reaction using high specific activity [^18^F]fluoride may enable new areas of PET imaging research.

**Figure 2:**
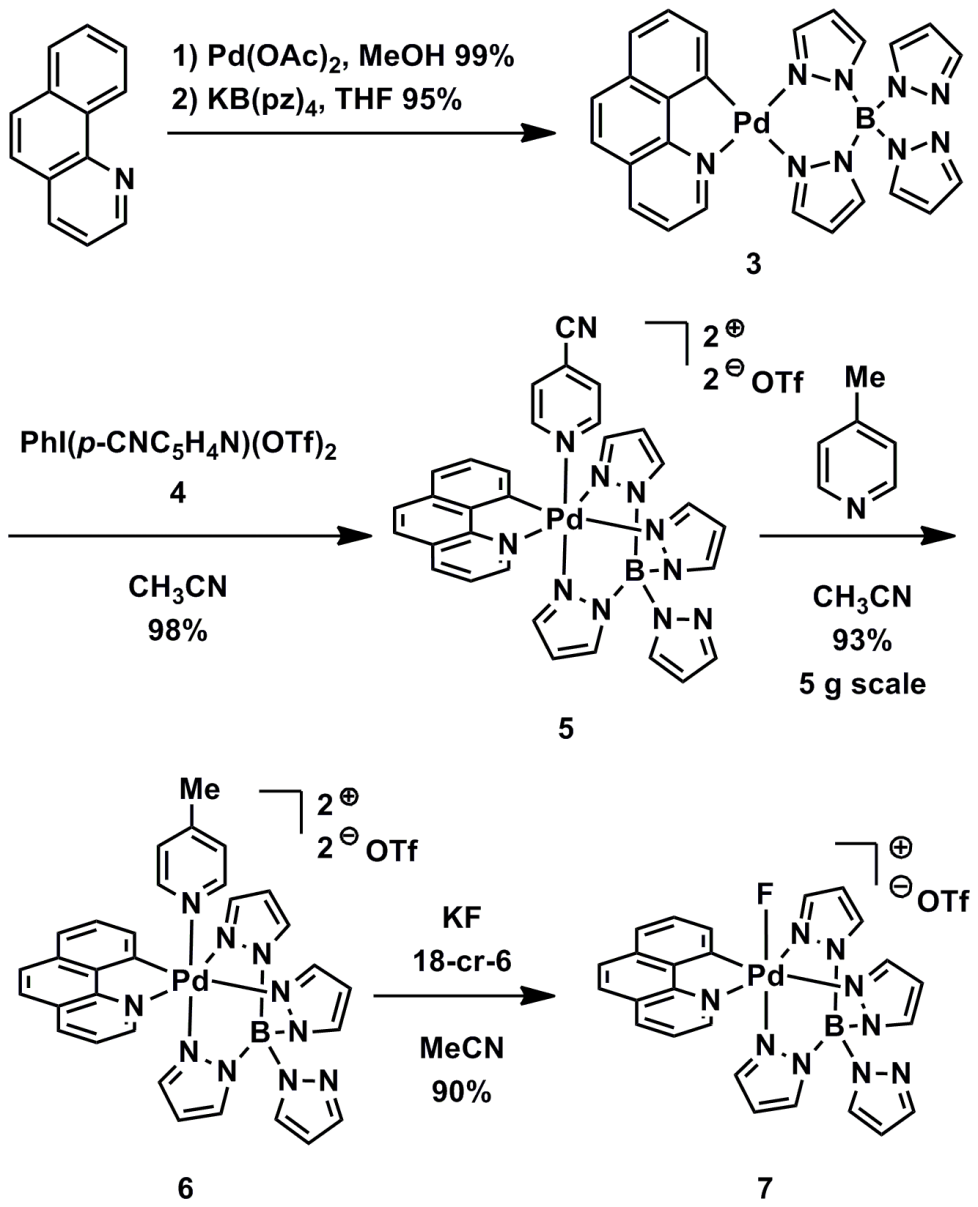
New electrophilic fluorination reagent. Synthesis of high-valent palladium complex 7 that is an electrophilic fluorination reagent derived from complex 6 through fluoride capture.

We reported the first high-valent transition metal fluoride complexes observed to undergo reductive elimination to form aryl fluorides [28], [29]. We subsequently discovered that high-valent palladium fluoride complexes could behave as electrophilic fluorination reagents, when other reductive processes such as reductive elimination were inhibited by an appropriate choice of ligands [24]. But, high-valent transition metal fluorides, including those described in our original report [28], are typically themselves synthesized from electrophilic fluorination reagents and not from fluoride, which would not overcome the specific activity challenge when dealing with ^18^F. Therefore, we designed a high-valent organometallic complex **6** that can capture fluoride via ligand exchange to form a high-valent transition metal fluoride complex **7**, which can serve as an electrophilic fluorination reagent (Figure 2). The complex was synthesized via benzo[*h*]quinoline cyclopalladation, followed by ligand exchange with potassium tetrapyrazolyl borate to afford palladium(II) complex **3** that was oxidized to high-valent palladium(IV) complex **5** using the λ_3_-iodinane reagent **4**. Exchange of the apical 4-cyanopyridine ligand with picoline provided complex **6** that can capture fluoride. Complex **6**, which can be synthesized on multi-gram scale, is not unique in its ability to capture fluoride. For example the pyridine (instead of picoline) analog of **6** can capture fluoride as well (see Supporting Information S1). Nevertheless, **6** is currently the complex of choice because the picoline ligand imparts enough electron-donating character to the electron-deficient palladium center for long-term storage, but can quickly be displaced by fluoride.

### Important Characteristics of High-valent Organometallic Complexes 6 and 7

**Figure 3:**
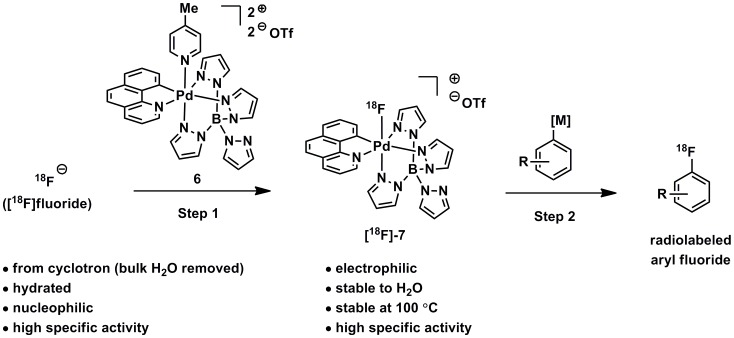
Two-step procedure for synthesis of radiolabeled aryl fluorides. The procedure consists of capture of [^18^F]fluoride by palladium(IV) complex **6** to form electrophilic ^18^F-fluorination reagent **[^18^F]-7** and fluorine transfer to aryl metal complexes.

**Figure 4:**
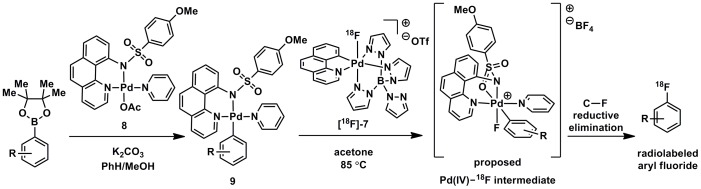
General palladium-mediated synthesis of radiolabeled aryl fluorides. C–F bond formation is proposed to occur by oxidation of **9** with **[^18^F]-7** leading to a proposed Pd(IV)–^18^F intermediate followed by C–F reductive elimination.

Complex **6** can capture ^18^F from samples of [^18^F]fluoride (Figure 3, Step 1) and then serve as an electrophilic ^18^F fluorination reagent (Figure 3, Step 2). Complex**[^18^F]-7** can be used as the electrophilic reagent, for the fluorination of pyridylsulfonamide-stabilized palladium (II) complexes **9** (see Figure 4) [30], and can provide access to electron-rich, electron-neutral, and electron-poor functionalized aryl fluorides. High-valent palladium complex **7** is likely the first of a class of complexes that exhibit the desired reactivity (capture and electrophilic transfer of fluoride to make C–F bonds).

The ability of **6** and **7** to capture and transfer fluoride is unusual. Complex **7** functions as an electrophilic reagent, similar to conventional electrophilic fluorination reagents previously used [30]. Yet, unequivocally, fluoride is the original source of fluorine. The generation of an electrophilic fluorination reagent from fluoride, without electrochemical methods, is challenging because fluorine is the strongest elemental oxidant known. From an electrostatic standpoint, the electropositive palladium center of complex **6** is able to attract the fluoride anion. High-valent palladium(IV) fluoride **7** was designed to prevent unproductive C–F reductive elimination by the inclusion of multidentate ligands. Reductive elimination from octahedral complexes is typically preceded by ligand dissociation to a penta-coordinate species [29], [31], which is disfavored by multidentate ligands. Additionally, tetrapyrazole borate ligands possess the ability to coordinate in either a bidentate or tridentate mode and can switch between them to accommodate the appropriate coordination chemistry needed. Specifically, tridentate coordination is important to stabilize the octahedral geometry for Pd(IV)d^6^, but, with minimal structural reorganization, bidentate coordination, of a square planar Pd(II)d^8^ complex is possible, the geometry of the complex resulting after fluoride transfer. The ability to access different coordination modes may lower the activation barrier to fluorination. Finally the triflate counterion was chosen because it is non-coordinating so as to not displace fluoride from the palladium center, and it does not contain readily exchangeable fluoride. Common, non-coordinating counterions such as BF_4_^–^ and SbF_6_^–^ would introduce exogenous ^19^F into the sample and lower the specific activity of the final molecule.

### Synthesis and Radiofluorination of Complex Small Molecules

**Figure 5:**
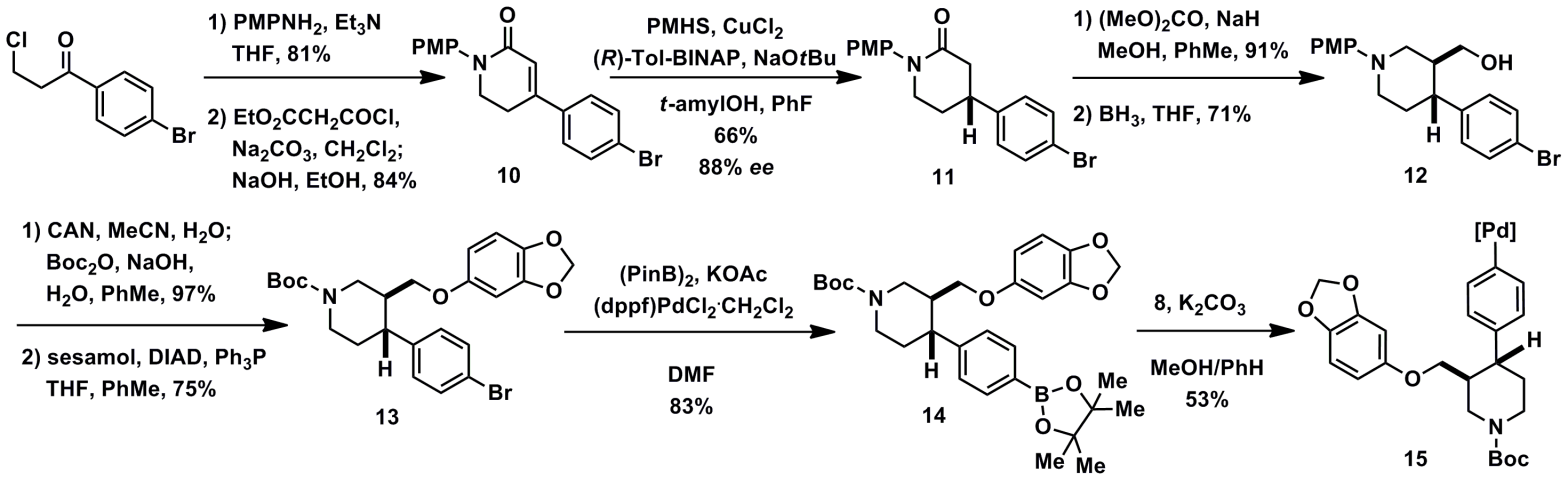
Synthesis of palladium(II) aryl substrate 15.

**Figure 6:**
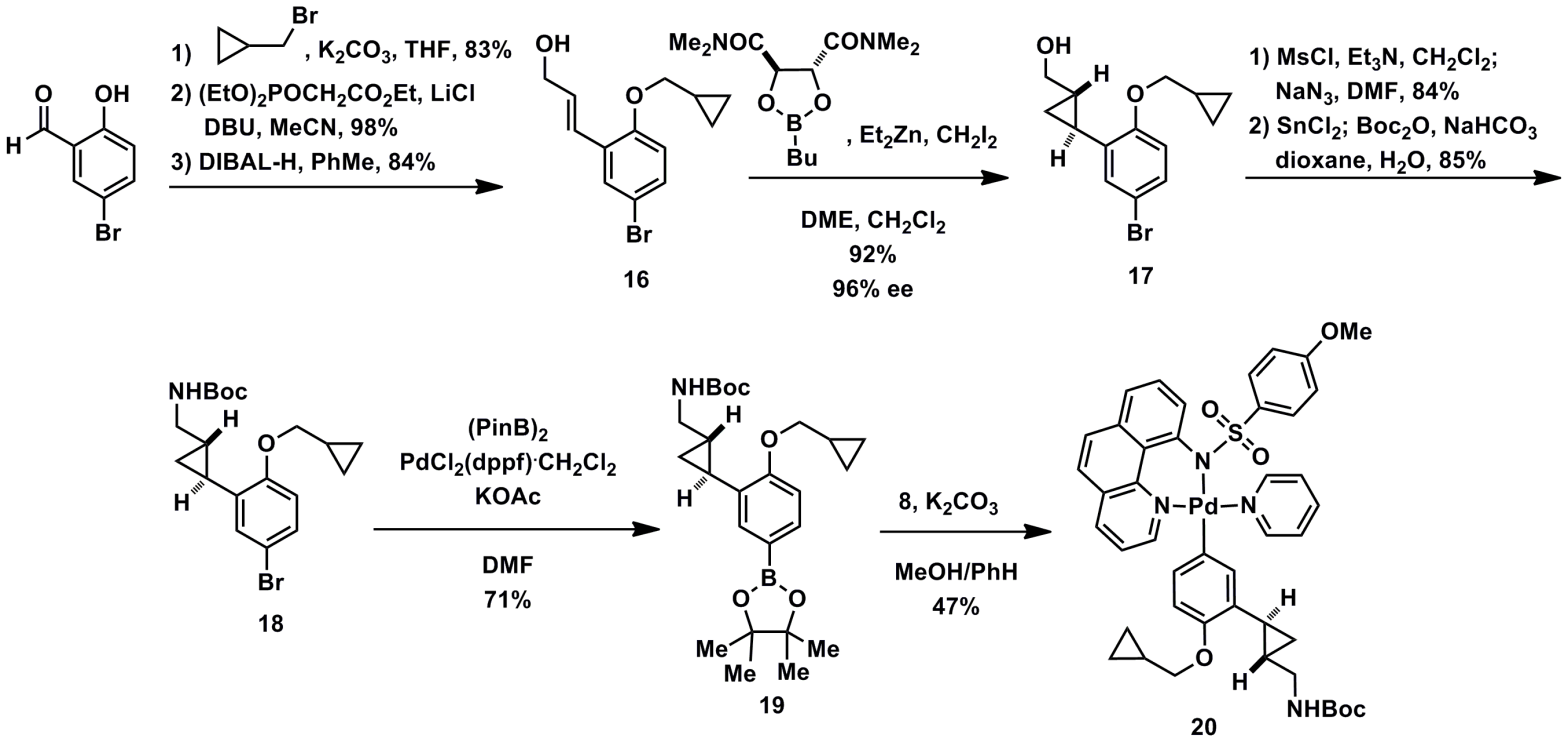
Synthesis of palladium(II) aryl substrate 20.

Radiolabeled **[^18^F]-7** can be used as the electrophilic reagent in the palladium-mediated fluorination reaction we have previously disclosed [30] to form radiolabeled aryl fluorides (Figure 4). The mechanism of fluorination is proposed to occur by oxidation of and transfer of fluorine from **[^18^F]-7** to **9** to form a new Pd(IV)–^18^F complex, which can then undergo C–F reductive elimination to afford ^18^F-labeled aryl fluoride. Evidence for oxidative fluorine transfer has been established: A high-valent aryl Pd(IV)–F complex could be observed by NMR spectroscopy after oxidation of an aryl Pd(II) complex, specifically **9** with the aryl and pyridine substituent linked, with **7** [24]. Complex small molecule substrates can be made in a modular fashion by transmetalation of aryl boronic acid derivatives with palladium acetate complex **8**, synthesized in four steps from commercially available reagents (see Supporting Information S1). Many palladium(II) aryl complexes, for example **15** and **20** (substrates leading to **1** and **2**), can be purified by chromatography on silica gel, are stable to air and moisture, and can be stored for extended periods of time without decomposition (>1 year). Syntheses of palladium aryl complexes **15** and **20** are shown in Figures 5 and 6.

Palladium complexes **15** and **20** were constructed from commercially available starting materials using a placeholder functional group at the eventual site of fluorination. For syntheses via late-stage fluorination of **1** and **2**, aryl bromides are used as the placeholders then changed to aryl boronic esters that can undergo transmetalation on to the palladium center in the penultimate step. There are no radiochemistry time constraints associated with the synthesis of the palladium aryl complexes, because ^18^F is only incorporated after the palladium substrates are synthesized. For the non-chemists, fluorination precursors (e.g. **15**) are not trivial to make, but could be made available for purchase. Palladium complex **15**, the precursor to paroxetine (**1**) is synthesized via a route similar to a published route [32], but with an aryl bromide in lieu of an aryl fluoride, which simplifies access to the desired substrate. Alkylation, acylation, and cyclization reactions starting form of 1-(4-bromophenyl)-3-chloropropan-1-one affords dihydropyridone **10** (Figure 5). Asymmetric conjugate reduction using conditions developed by Buchwald [32] provide enantioenriched δ-lactam 11 in 88% *ee*. Recrystallization of an intermediate later in the synthesis (**12**) provides material that is >99% *ee*. A Claisen condensation followed by reduction gives alcohol **12**. Protecting group exchange followed by Mitsunobu reaction with sesamol and DIAD affords **13** that possesses the full carbon skeleton of paroxetine. Borylation yields **14** and transmetalation provides palladium complex **15**.

For the palladium complex **20** leading to 5-HT_2C_ agonist **2**, alkylation, olefination, and reduction of 5-bromosalicaldehyde affords allylic alcohol **16** that is used in a Charette asymmetric cyclopropanation reaction [33] to yield cyclopropane **17** in 96% *ee* (Figure 6). The enantiomeric excess can be upgraded at a later point (**18**) via recrystallization to >99% *ee*. Alcohol to amine conversion and deprotection leads to the aryl bromide **18** that undergoes palladium-catalyzed borylation to afford **19**. Aryl boronic ester is converted to palladium complex **20** through transmetalation.

**Figure 7:**
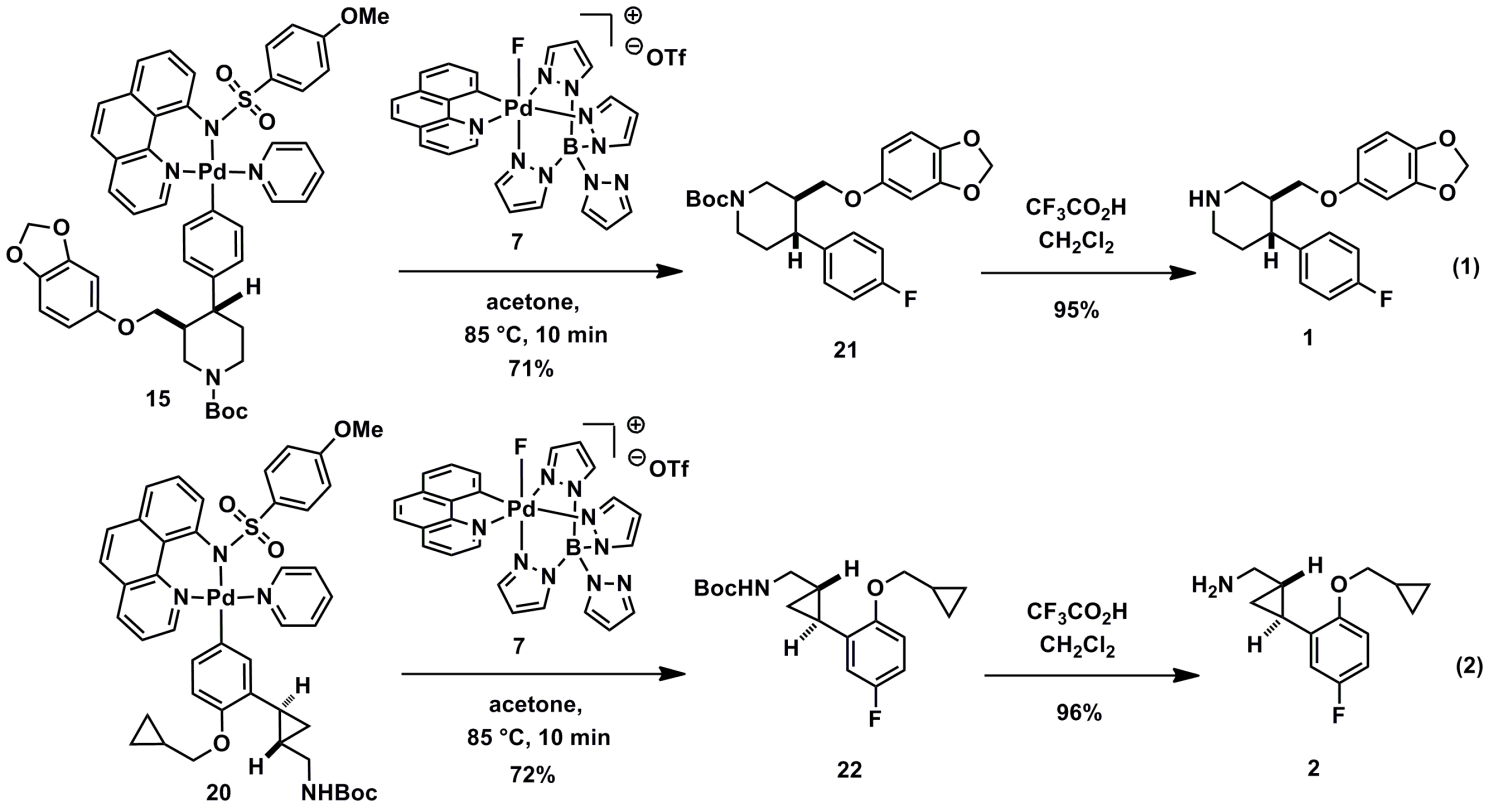
Synthesis of aryl fluorides 1 and 2 via late-stage fluorination with electrophilic reagent 7.

We have found that the yield of the “cold” (^19^F) fluorination reactions for a given substrate roughly correlates with the radiochemical yield of the “hot” (^18^F) fluorination reactions. Palladium aryl substrates **15** and **20** were fluorinated with the non-radiolabeled (^19^F) version of palladium complex **6** to afford small molecule aryl fluorides **21** and **22** in 71% and 72% yield, respectively (Figure 7; Equations 1 and 2). Removal of protecting groups reveals paroxetine (**1**) and the 5-HT_2C_ agonist **2**.

### Scope and Limitations of Fluorination Reaction

Fluorination of **15** and **20** demonstrates the functional group tolerance of our protocol, and establishes that late-stage fluorination in the presence of a variety of functional group is possible for electron-rich arenes, that are often challenging to prepare with conventional fluorination reactions. Currently, our method does not tolerate nucleophilic, basic functional groups, such as certain amines. While pyridine-substituents are tolerated, amines of higher basicity such as tertiary alkyl amines are not. We have overcome this limitation in some cases through the use of protecting groups such as the Boc groups used for substrates **15** and **20**. Additionally, substitution besides hydrogen ortho to the site of fluorination reduces the efficiency of palladium aryl substrate synthesis and fluorination. An additional limitation is the required synthesis of the palladium complexes themselves. While generally stable, the synthesis requires some knowledge of organometallic chemistry.

### Practical and Procedural Considerations for the Transition to Radiochemistry

The syntheses of **[^18^F]-1** and **[^18^F]-2** with [^18^F]fluoride were accomplished with procedural modifications based on the physical and practical differences between fluorination chemistry with ^18^F and ^19^F. First, [^18^F]fluoride was prepared following common radiochemistry protocols from a sample produced in [^18^O]H_2_O by a cyclotron [34]. Second, we adapted the procedures for the difference in stoichiometry between reactions with ^19^F and ^18^F. For high specific activity [^18^F]fluoride reactions only nanomoles of the electrophilic fluorination reagent are synthesized, which remain in the presence of a large excess of **6**, the palladium precursor. In practice, we found that it was not feasible or advantageous to rapidly and cleanly isolate **[^18^F]-7** from the mixture. Therefore, the ^18^F method was modified into a two-step process with the reagent **[^18^F]-7** made *in situ.*

**Figure 8:**
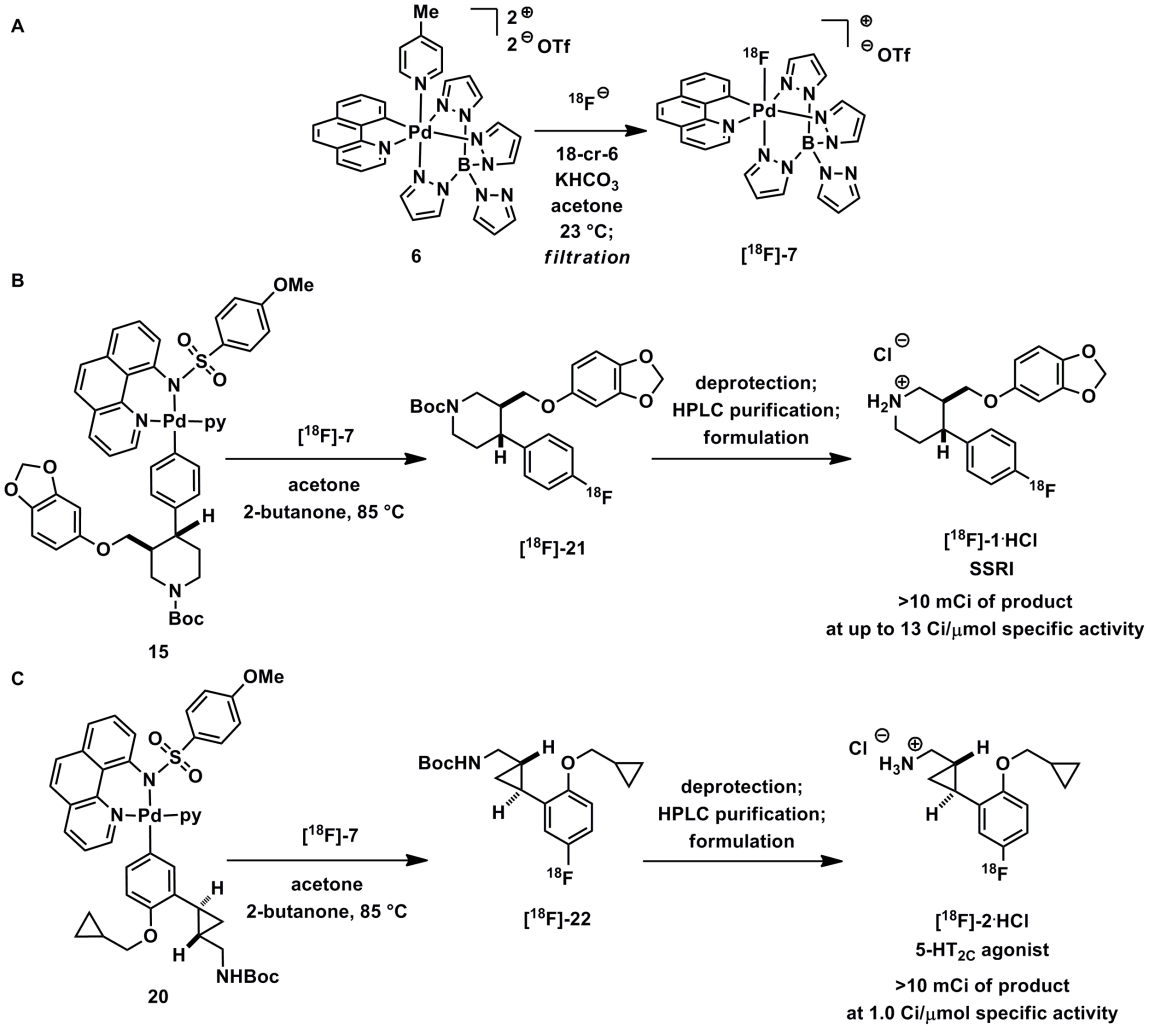
Palladium-mediated synthesis of [^18^F]-1 and [^18^F]-2 using [^18^F]-7 derived from [^18^F]fluoride. (A) Synthesis of the electrophilic fluorination reagent **[^18^F]-7** for reaction with palladium aryl complexes. (B) Synthesis of SSRI **[^18^F]-1** on a scale suitable for NHP PET imaging. (C) Synthesis of 5-HT_2C_ agonist **[^18^F]-2** on a scale suitable for NHP PET imaging.

The two-step fluorination reaction can afford radiolabeled aryl fluorides on a scale suitable for PET imaging (Figure 8). Several variables were explored and optimized to accomplish efficient and quick fluorination with **[^18^F]-7**. Samples of [^18^F]fluoride in H_2_O with KHCO_3_ and the common phase transfer reagent, 18-crown-6, were azeotropically dried with MeCN and acetone (Figure 8A). 18-Crown-6 was chosen instead of common cryptand phase transfer reagents (e.g. Kryptofix® 2.2.2), because **5** reacts unproductively with the amine functionality found in Kryptofix® 2.2.2. Initial variability in terms of overall radiochemical yield was often associated with the drying process (“dry-down”) and re-solubilizing of fluoride. While the reaction tolerates the presence of water, the efficiency decreases as water concentration increases. Next, palladium complex **5**, dissolved in acetone, was added for fluoride capture to form fluorinating reagent **[^18^F]-7** in less than 10 minutes. Before fluorination, the reaction mixture is filtered over a pyridine-functionalized resin, JandaJel™, as a quick intermediate purification step, which resulted in higher yields compared to experiments omitting filtration. Filtration removes byproducts that precipitate during the reaction. Pyridine-functionalized solid support is more effective than other filtration methods, which may be due to pyridine capturing **5** by displacing the picoline ligand. After filtration, the palladium aryl substrate is added, and the resulting solution was heated at 85°C in a sealed vial.

Generally, radiochemistry methods are first explored using hand manipulations on a small amount of radioactivity (<500 µCi of [^18^F]fluoride). However, a typical PET imaging experiment in NHPs requires 5–10 mCi of final radiolabeled pure product, which along with the efficiency of the reaction dictates that the starting amount of [^18^F]fluoride needs to be anywhere from 50 mCi to 1 Ci of radioactivity. Currently, our fluorination method requires starting with 1 Ci to produce enough for NHP PET imaging experiments. Due to safety considerations, automated synthesis protocols were developed using a commercially-available platform and took place inside of a hot cell. Synthesis modules were programmed to run the reaction (azeotropic drying, fluoride capture, filtration, and fluorination; see Supporting Information S1). The automated procedures can now be used to produce tens of mCi of radiolabeled aryl fluorides prior to purification (Figure 8B and 3C).

Post synthesis protocols include 1) filtration through a plug of silica to remove bulk metal contaminants, 2) removal of protecting groups, 3) HPLC purification for isolation, and 4) formulation in a sterile solution for administration. Post synthesis manipulations are currently conducted by hand, though ideally will be automated in the future.

While protecting groups are common in radiochemistry, deprotection reactions must be fast and efficient. For the two radiolabeled molecules in this research, we chose to protect the primary and secondary amines with easily removable *t*-butoxycarbonyl (Boc) groups. Exposure of **[^18^F]-21** and **[^18^F]-22** to trifluoroacetic acid for two minutes revealed the ammonium salts.

Because the mass of fluorinated material using high specific activity [^18^F]fluoride is typically less than 10 µg among milligrams of reaction reagents, HPLC can be an efficient and often used [35] tool to remove contaminants and byproducts. We have devised routine HPLC methods that purify **[^18^F]-1** and **[^18^F]-2** with standard semi-preparatory columns in less than 15 minutes. Final samples are chemically and radiochemically pure and have less than 5 parts per billion palladium (ppb) content, well below suggested guidelines for use in humans (<1000 ppb) [36]. The residual amount of palladium was determined by inductively coupled plasma mass spectroscopy (ICP-MS) analysis.

After HPLC purification, the sample is captured with a solid phase extraction column and formulated in a sterile saline solution. Final samples were analyzed for purity, residual solvent, pH, pyrogenicity, and sterility and passed all quality control protocols generally used for PET imaging in NHPs.

Starting with an approximated 1 Ci of radioactivity, our method can be used to produce >10 mCi of formulated product in up to 13 Ci/µmol specific activity at time of injection (TOI), suitable for imaging primates. *The entire process from obtaining aqueous samples of ^18^F through formulation can be accomplished in less than 100 min.*

### Non-human Primate PET Imaging with [^18^F]-1 and [^18^F]-2

Using the procedure outlined above, we prepared **[^18^F]-1** and **[^18^F]-2** for use in four separate MR-PET imaging experiments in baboons (*Papio anubis*). The objective of these studies was to determine the brain pharmacokinetic profile and distribution of **[^18^F]-1**and **[^18^F]-2** and compare the binding and kinetic profile to scans performed after a drug challenge. This “test-block” procedure provides information about the nature of the radioactive signal that is observed. The initial protocol is designed to determine the contribution of specific and non-specific binding to the PET signal. Specific binding interactions, (i.e. **[^18^F]-1** with SERT [37]; **[^18^F]-2** with 5-HT_2C_), will undergo competition with drug pretreatment resulting in an overall reduced PET signal; however binding interactions that are non-specific and not in competition with the drug will remain the same. Determining the extent to which the signal observed is a specific binding interaction is the first and most crucial step in radiotracer validation for receptor systems in the brain; it is also one of the least predictable outcomes in radiotracer development [38]. We chose blocking drugs (citalopram [39] 5.0 mg/kg for SERT; ritanserin [40] 0.1 mg/kg for 5-HT_2C_) that were known to be safe for administration intravenously [41], [42] rather than performing self-saturation experiments with the non-radioactive analogs of **[^18^F]-1** and **[^18^F]-2**.

Radiolabeling drug molecules with positron-emitting isotopes has intrinsic value, which is particularly true of central nervous system (CNS) drugs. Inferences about brain penetration and pharmacokinetic profile (PK) in humans are often made through extrapolation from studies in lower mammals and drug concentrations in human plasma, because observing drug concentration at target sites *in vivo* in humans is challenging. Many drug molecules, commonly prescribed for the human CNS, are used without any knowledge of human brain PK. Understanding the brain distribution and kinetic profile of drugs or drug candidates in humans can help understanding drug failures and may ultimately provide better predictive tools for success in drug development [43], [44]. While there are many reasons why drugs are not often labeled and evaluated with PET, a striking number of molecules cannot be labeled with either of the two most common PET isotopes, carbon-11 and fluorine-18. Paroxetine, a selective serotonin reuptake inhibitor, is a good example of a molecule that was previously considered inaccessible for high specific activity labeling approaches. The challenge of labeling paroxetine (**1)** was overcome using palladium-mediated fluorination and provided **[^18^F]-1** in suitable yield for initial studies in NHPs.

### PET Study to Evaluate Biodistribution and Pharmacokinetic Profile

**Figure 9:**
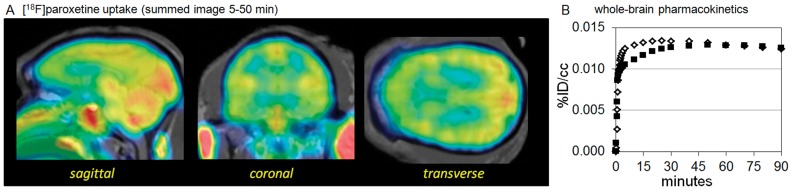
Distribution and pharmacokinetic profile of [^18^F]paroxetine ([^18^F]-1) from MR-PET imaging in non-human primates. (A) Summed images from 5–50 min of the dynamic PET collected following injection of **[^18^F]-1**. The images are fused with a structural (T1 weighted) MR image. Although **[^18^F]-1** is a potent and selective ligand for the serotonin transporter (SERT), the observed binding does not match the distribution of SERT. This is indicative of high non-specific binding. The images highlight the overall high blood-brain-barrier penetration of **[^18^F]-1** and heterogeneity of binding observed. (B) Whole-brain average time-activity curves from **[^18^F]-1** from baseline and pretreatment (citalopram) studies.

Two MR-PET imaging studies were performed with **[^18^F]-1**, a baseline and a challenge with citalopram, a competitive serotonin reuptake inhibitor. The baseline scan revealed modest brain penetration (0.013% ID/cc) corresponding to about 1.7% of the total injected dose accumulating in the brain. Penetration into and accumulation in the brain were rapid (Figure 9), reaching a plateau between 15–30 min. Washout kinetics were markedly slow with a t_1/2_ calculated from the data (35–90 min) of 21. 3 h. This is consistent with the plasma half-life range (21–24 h) reported in the monograph for Paxil [45] (one of the trade-names for paroxetine). The accumulation of **[^18^F]-1** represents predominantly non-specific uptake in the brain. Heterogeneous binding was observed, but was inconsistent with the known SERT distribution from PET studies with validated radiotracers such as [^11^C]DASB [23]. The lack of specific binding was confirmed by citalopram challenge, in which there was no significant change in the accumulation and distribution of **[^18^F]-1** relative to baseline. Although data analysis with **[^18^F]-1** may be limited by the high degree of non-specific binding, the prevalence of paroxetine used to treat depression warrants evaluation of brain PK and distribution in humans.

### PET Study to Evaluate a Potential PET Radiotracer

Our choice to evaluate **[^18^F]-2** as a selective radiotracer for the serotonin 2c (5-HT_2C_) receptor was based upon the growing associations between 5-HT_2C_ and brain-related disorders [46], [47], [48], [49]. Although the associations exist, the 5-HT_2C_ receptor has previously received less attention in psychopharmacology and neuroimaging than its homolog, 5-HT_2A_. To date, selective 5-HT_2C_ radioligands for autoradiography and 5-HT_2C_ radiotracers for imaging have not been described, a fact which has no doubt hindered research in neurobiology. Due to the recent development of novel scaffolds for potent and selective ligands of 5-HT_2C_, *in vivo* imaging of 5-HT_2C_ has become a realistic goal [46]. Very few, if any, of the molecules that selectively target 5-HT_2C_ are obvious labeling candidates for either carbon-11 or fluorine-18; much less, compounds for which *in vivo* data support the likelihood for radiotracer development success. Agonist **2** was chosen from the literature not only because it was well-suited for the chemistry described, but also because of its selectivity profile (40-fold and 14-fold selective for 5-HT_2C_ over 5-HT_2A_ and 5-HT_2B_, respectively) and its demonstrated efficacy in an animal model of antipsychotic drug activity [21]. To our knowledge, this is the first reported evaluation of a 5-HT_2C_-targeted molecule for use in PET.

**Figure 10:**
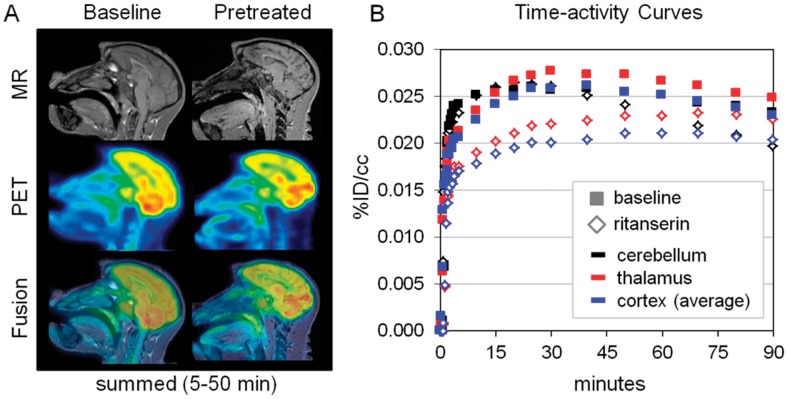
Preliminary [^18^F]-2 MR-PET imaging in non-human primates. (A) Summed images from 5-50 min of the dynamic PET collected following two studies (baseline and ritanserin pretreated) with **[^18^F]-2**. The images highlight the overall high blood-brain-barrier penetration of **[^18^F]-2** and heterogeneity of binding observed. The highest concentrations were noted in the thalamus, cerebellum, and occipital cortex. (B) Time-activity curves from region-of-interest (ROI) analysis from the dynamic data represented in A. As seen in the kinetic profiles, ritanserin did not fully block the uptake of **[^18^F]-2**; however it did reduce binding and alter the pharmacokinetic profile in the thalamus and cortex but not the cerebellum. The extent to which this represents 5HT_2c_ binding will require validation by additional imaging experiments.

Two preliminary imaging studies in non-human primates were used to determine whether **[^18^F]-2** exhibited suitable blood-brain penetration, pharmacokinetic profile, and specific binding suitable for use as a 5-HT_2C_ PET tracer (Figure 10). Data from the baseline study indicate that **[^18^F]-2** has excellent brain penetration with an average brain : plasma ratio of 1.5 at 30 minutes post injection. Analysis of arterial plasma from this experiment suggested that the compound had suitable metabolic stability (nearly 70% of radioactivity in plasma was associated with intact **[^18^F]-2** at 60 min) to warrant continuation to pharmacological challenge studies. For the initial drug challenge, we chose ritanserin, which is a non-selective 5-HT_2A_ (EC_50_ = 4.6 nM), 5-HT_2B_ (2.2 nM), and 5-HT_2c_ (6.6 nM) antagonist [50]. We observed a modest 15% decrease in uptake of **[^18^F]-2** following ritanserin administration. The decrease was observed in the thalamus and throughout the cortex (frontal, medial parietal and temporal), but not in the cerebellum. Decreases in the thalamus and cortex are reasonably correlated with expectations from 5-HT_2C_ mRNA mapping in the monkey [51] and human brains [52] *ex vivo*. The lack of blockade in the cerebellum is also consistent with the assumed 5HT_2c_ receptor distribution. One notable exception is that higher levels of uptake and blockade were anticipated in the choroid plexus. Our preliminary data, which indicate modest specific binding, are encouraging and warrant additional studies to more accurately quantify the reduction in binding potential of **[^18^F]-2** during pharmacological challenge.

## Methods

See Supporting Information S1.

### Conclusions

We have developed a modern fluorination reaction with large substrate scope that can be used for the synthesis of complex ^18^F-labeled aryl fluorides suitable for imaging in NHPs. Our synthesis of radiolabeled aryl fluorides employs [^18^F]fluoride, is fast, high-yielding, and affords only one aryl fluoride constitutional isomers and no inseparable byproducts. We have demonstrated that our method can be translated to synthesize radiolabeled aryl fluorides **[^18^F]-1** and **[^18^F]-2** in quantity and quality suitable for PET imaging studies in baboons to obtain pharmacokinetic data. Our method can tolerate a large variety of functional groups, and is suitable for electron-rich and –deficient arenes, which is a challenge for conventional fluorination reactions. Current limitations of the method are the inability to tolerate certain nucleophilic functional groups, most notably tertiary amines, and the substantially lower yields for substrates with ortho substitution. Moreover, our method is not as simple to perform as a conventional nucleophilic aromatic substitution reaction and requires the synthesis of organotransition metal reagents.

We see utility in our method for the predictable synthesis of ^18^F-labeled molecules that can support drug development, for example, small molecule drug candidates with drug-like structures such as paroxetine can now be labeled. In addition, we provide a tool that enables the synthesis of ^18^F-labeled molecules for evaluation as potential PET tracers that report on a biological function. PET tracer development is challenging beyond synthesis, as exemplified by the high-nonspecific binding of paroxetine, which makes [^18^F]paroxetine (**[^18^F]-1**) unsuitable for imaging of SERT. But the ability to make more complex and structurally more diverse ^18^F-labeled molecules more quickly, more reliably, and more efficiently than previously possible, may shift the future challenge toward PET tracer development rather than PET tracer synthesis. New fluorination methods that expand the number and types of molecules that can be radiolabeled blended with advances in tracer development should increase the impact of PET imaging. In this respect, we are particularly excited about a new method developed in our group, which is based on nickel complexes [53], and may be more easily executed, also by the non-expert.

## Supporting Information

Supporting Information S1 **Contains details on materials and methods, experimental data for chemistry and radiochemistry, and spectroscopic data.**
(PDF)
