# Supplementary material for: Application of Palladium-Mediated 18F-Fluorination to PET Radiotracer Development: Overcoming Hurdles to Translation
Source: PLoS One. 2013 Mar 12;8(3):e59187. doi: 10.1371/journal.pone.0059187 (PMC3595243; doi:10.1371/journal.pone.0059187)
Supplement: Supporting Information S1 — Contains details on materials and methods, experimental data for chemistry and radiochemistry, and spectroscopic data. (PDF) [file pone.0059187.s001.pdf]

Supporting Information S1

Application of Palladium-mediated ^{18}F-Fluorination to PET Radiotracer Development: Overcoming Hurdles to Translation

Adam S. Kamlet[1], Constanze N. Neumann[1], Eunsung Lee[1], Stephen M. Carlin[2], Christian K. Moseley[2], Nickeisha Stephenson[1], Jacob M. Hooker[2,3]*, Tobias Ritter[1,3]*

[1] Department of Chemistry and Chemical Biology, Harvard University, Cambridge, Massachusetts, United States, [2] Athinoula A. Martinos Center for Biomedical Imaging, Massachusetts General Hospital and Harvard Medical School, Charlestown, Massachusetts, United States, [3] Division of Nuclear Medicine and Molecular Imaging, Department of Radiology, Massachusetts General Hospital, Boston, Massachusetts, United States

E-mail: hooker@nmr.mgh.harvard.edu; ritter@chemistry.harvard.edu

Table of Contents

Materials and Methods 4

Experimental Data 5

Synthesis of Pd(IV) complex **6** 5

Benzo[*h*]quinolinyl palladium acetate dimer (**S1**) 5

Potassium tetra(1*H*-pyrazol-1-yl)borate (**S2**) 5

Benzo[*h*]quinolinyl (tetrapyrazolylborate)palladium (**3**) 6

1,1'-(Phenyl-λ^3-iodanediyl)bis(4-cyanopyridinium) bis(trifluoromethanesulfonate) (**4**) 7

Benzo[*h*]quinolinyl (tetrapyrazolylborate) Pd(IV) 4-cyanopyridine trifluoromethanesulfonate (**5**) 7

Benzo[*h*]quinolinyl (tetrapyrazolylborate) Pd(IV) 4-picoline trifluoromethanesulfonate (**6**). 8

Synthesis of Pd(IV) fluoride complex **7** 9

Benzo[*h*]quinolinyl (tetrapyrazolylborate) Pd(IV) fluoride trifluoromethanesulfonate (**7**) 9

Figure S1. NMR Spectra of **7** in 10% aqueous acetonitrile solution 10

Alternative synthesis of **7** 11

Bis(pyridinio-1)iodobenzene bis(trifluoroacetate) (**S3**) 11

Benzo[*h*]quinolinyl (tetrapyrazolylborate) Pd(IV) pyridine trifluoromethanesulfonate (**3**)...11

Benzo[*h*]quinolinyl (tetrapyrazolylborate) Pd(IV) fluoride trifluoromethanesulfonate (**7**) .. 12

Synthesis of aryl palladium complexes (**15** and **20**, examples of **9**) 12

[{(4-Methoxyphenyl)sulfonyl}imino]phenyliodinane (**S5**) 12

Benzo[*h*]quinolinyl palladium chloro dimer (**S6**) 13

Chloro palladium complex (**S7**) 14

Acetato palladium complex (**8**) 14

1-(4-Bromophenyl)-3-((4-methoxyphenyl)amino)propan-1-one (**S8**) 15

4-(4-Bromophenyl)-1-(4-methoxyphenyl)-5,6-dihydropyridin-2(1*H*)-one (**10**) 15

(*R*)-4-(4-Bromophenyl)-1-(4-methoxyphenyl)piperidin-2-one (**11**) 16

(3*S*,4*R*)-Methyl 4-(4-bromophenyl)-1-(4-methoxyphenyl)-2-oxopiperidine-3-carboxylate (**S9**) 17

Figure S2. Enantiodiscriminating HPLC trace of **S9** 18

((3*S*,4*R*)-4-(4-Bromophenyl)-1-(4-methoxyphenyl)piperidin-3-yl)methanol (**12**) 18

Figure S3. Enantiodiscriminating HPLC trace of **12** 19

(3*S*,4*R*)-*t*-Butyl 4-(4-bromophenyl)-3-(hydroxymethyl)piperidine-1-carboxylate (**S10**)...... 19

(3*S*,4*R*)-*t*-Butyl 3-((benzo[*d*][1,3]dioxol-5-yloxy)methyl)-4-(4-bromophenyl)piperidine-1-carboxylate (**13**) 20

(3*S*,4*R*)-*t*-Butyl 3-((benzo[*d*][1,3]dioxol-5-yloxy)methyl)-4-(4-(4,4,5,5-tetramethyl-1,3,2-dioxaborolan-2-yl)phenyl)piperidine-1-carboxylate (**14**) 21

Palladium aryl complex **15** 21

5-Bromo-2-(cyclopropylmethoxy)benzaldehyde (**S11**) 22

(*E*)-Ethyl 3-(5-bromo-2-(cyclopropylmethoxy)phenyl)acrylate (**S12**) 23

(*E*)-3-(5-Bromo-2-(cyclopropylmethoxy)phenyl)prop-2-en-1-ol (**16**) 23

((1*S*,2*S*)-2-(5-Bromo-2-(cyclopropylmethoxy)phenyl)cyclopropyl)methanol (**17**) 24

Figure S4: Enantiodiscriminating HPLC trace of **17** 25

2-((1*S*,2*S*)-2-(Azidomethyl)cyclopropyl)-4-bromo-1-(cyclopropylmethoxy)benzene (**S13**) 26

t-Butyl (((1S,2S)-2-(5-bromo-2-(cyclopropylmethoxy)phenyl)cyclopropyl)methyl) carbamate (**18**) 26

Figure S5. Enantiodiscriminating HPLC trace of **18** 27

t-Butyl (((1S,2S)-2-(2-(cyclopropylmethoxy)-5-(4,4,5,5-tetramethyl-1,3,2-dioxaborolan-2-yl)phenyl)cyclopropyl)methyl)carbamate (**19**) 28

Palladium aryl complex **20** 28

Fluorination of aryl palladium complexes 30

(3*S*,4*R*)-*t*-Butyl 3-((benzo[*d*][1,3]dioxol-5-yloxy)methyl)-4-(4-fluorophenyl)piperidine-1-carboxylate (**21**) 30

Figure S6. Enantiodiscriminating HPLC trace of **21** 31

*t-B*utyl(((1S,2S)-2-(2-(cyclopropylmethoxy)-5-fluorophenyl)cyclopropyl)methyl)carbamate (**22**) 31

Deprotection of aryl fluorides 32

(3*S*,4*R*)-3-((Benzo[*d*][1,3]dioxol-5-yloxy)methyl)-4-(4-fluorophenyl)piperidine (**1**) 32

((1S,2S)-2-(2-(Cyclopropylmethoxy)-5-fluorophenyl)cyclopropyl)methanaminium 2,2,2-trifluoroacetate (**2**) 33

Radiochemistry 34

General methods 34

Radiosynthesis of ^{18}F-labeled Molecules 34

Automated syntheses of **[^{18}F]-1** and **[^{18}F]-2** using high specific activity [^{18}F]fluoride 35

Figure S7. Preparatory HPLC chromatograph for synthesis of **[^{18}F]-1** 36

Figure S8. Preparatory HPLC chromatograph for synthesis of **[^{18}F]-2** 37

Characterization of ^{18}F-labeled molecules 37

Figure S9. Characterization of purified **[^{18}F]-1** 38

Figure S10. Characterization of purified **[^{18}F]-2** ... 39

Determination of specific activity of **[^{18}F]-1**and **[^{18}F]-2** ... 40

Table S1. Data for standard curve of UV absorbance vs amount of **1** ... 40

Figure S11. Standard curve of UV absorbance vs amount of **1** ... 40

Table S2. Data for standard curve of UV absorbance vs amount of **2** ... 41

Figure S12. Standard curve of UV absorbance vs amount of **2** ... 41

Determination of palladium content in purified sample of **[^{18}F]-2** ... 42

Table S3. ICP/MS data for standard curve and reformulated samples ... 42

Figure S13. Standard curve of relative ion count vs palladium concentration ... 42

Baboon PET Imaging Experiments ... 43

Spectroscopic Data ... 44

References ... 114

Materials and Methods

All air- and moisture-insensitive reactions were carried out under an ambient atmosphere, magnetically stirred, and monitored by thin layer chromatography (TLC) using EMD TLC plates pre-coated with 250 μm thickness silica gel 60 F254 plates and visualized by fluorescence quenching under UV light. Flash chromatography was performed on Dynamic Adsorbents Silica Gel 40–63 μm particle size using a forced flow of eluent at 0.3–0.5 bar pressure. All air- and moisture-sensitive manipulations were performed using oven-dried glassware, including standard Schlenk and glovebox techniques under an atmosphere of nitrogen. Methylene chloride was purged with nitrogen, dried by passage through activated alumina, and stored over 3Å molecular sieves.[1] Benzene, benzene-d_6, diethyl ether, toluene, pentane, dioxane, and THF were distilled from deep purple sodium benzophenone ketyl. Methylene chloride-d_2 was dried over CaH_2 and vacuum-distilled. Acetonitrile and acetonitrile-d_3 were dried over P_2O_5 and vacuum-distilled. Pyridine was dried over CaH_2 and distilled. DMSO was distilled from sodium triphenylmethanide and stored over 3Å sieves.[2] Acetone was distilled over B_2O_3. MeOH was degassed at –30 ºC under dynamic vacuum (10^{-4} Torr) for one hour and stored over 3Å sieves. Anhydrous DMF and dioxane bottles equipped with a SureSeal™ were purchased from Sigma Aldrich®. 18-Crown-6 was sublimed. KF was ground finely and dried at 200 ºC under dynamic vacuum (10^{-4} Torr) before use. Chloroform-d_1, D_2O, $Pd(OAc)_2$, AgOAc, and all other chemicals were used as received. All deutrated solvents were purchased from Cambridge Isotope Laboratories. $Pd(OAc)_2$, AgOAc, KBH_4, and 18-crown-6 were purchased from Strem Chemicals. Benzo[*h*]quinoline was purchased from TCI. (Diacetoxyiodo)benzene, potassium fluoride, 4-cyanopyridine, α-tetralone, pyrrolidine, *p*-toluenesulfonic acid, *p*-methoxybenzenesulfonamide, and F-TEDA-BF_4 (Selectfluor®) were purchased from Sigma-Aldrich®. Pyrazole, TMSOTf, and trifluoroacetic acid were purchased from Oakwood Products. Soda lime glass bottles were purchased from Qorpak®. NMR spectra were recorded on either a Varian Unity/Inova 600 spectrometer operating at 600 MHz for ^1H acquisitions, a Varian Unity/Inova 500 spectrometer operating at 500 MHz and 125 MHz for ^1H and ^{13}C acquisitions, respectively, a Varian Mercury 400 spectrometer operating at 375 MHz and 101 MHz for ^{19}F and ^{13}C acquisitions, respectively, or a Varian Mercury 300 spectrometer operating at 100 MHz for ^{11}B acquisitions. Chemical shifts were referenced to the residual proton solvent peaks (^1H: $CDCl_3$, δ 7.26; C_6D_6, δ 7.16; CD_2Cl_2, δ 5.32; D_2O, δ 4.79; $(CD_3)_2SO$, δ 2.50; CD_3CN, δ 1.94), solvent ^{13}C signals ($CDCl_3$, δ 77.16; C_6D_6, δ 128.06; CD_2Cl_2, δ 53.84; CD_3CN, δ 1.32, $(CD_3)_2SO$, δ 39.52),[3] dissolved or external neat PhF (^{19}F, δ –113.15 relative to $CFCl_3$) or dissolved 3-nitrofluorobenzene (–112.0 ppm). Signals are listed in ppm, and multiplicity identified as s = singlet, br = broad, d = doublet, t = triplet, q = quartet, quin = quintet, sep = septet, m = multiplet; coupling constants in Hz; integration. Concentration under reduced pressure was performed by rotary evaporation at 25–30 ºC at appropriate pressure. Purified compounds were further dried under high vacuum (0.01–0.05 Torr). Yields refer to purified and spectroscopically pure compounds. Elemental analysis was performed by Robertson Microlit Laboratories.

Experimental Data

Synthesis of Pd(IV) complex 6

Benzo[*h*]quinolinyl palladium acetate dimer (S1)

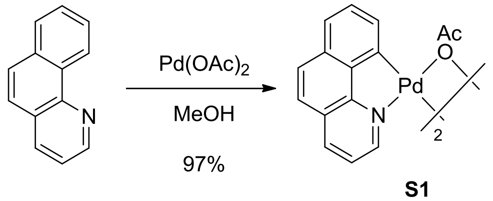

S1

Based on a reported procedure:[4] To benzo[*h*]quinoline (12.1 g, 67.6 mmol, 1.00 equiv) in MeOH (500 mL) in a round-bottom flask open to air at 23 ºC was added $Pd(OAc)_2$ (15.2 g, 67.6 mmol, 1.00 equiv). After stirring for 24 hours at 23 ºC, the solid was collected by filtration and washed with MeOH (100 mL) and diethyl ether (100 mL) to afford 22.5 g of the title compound as a yellow solid (97% yield).

NMR Spectroscopy: ^{1}H NMR (500 MHz, $CDCl_3$, 23 ºC, δ): 7.80 (dd, J = 5.5, 1.5 Hz, 1H), 7.43 (dd, J = 8.0, 1.5 Hz, 1H), 7.24–7.18 (m, 3H), 7.08 (dd, J = 7.0, 1.5 Hz, 1H), 6.97 (d, J = 9.0 Hz, 1H), 6.46 (dd, J = 7.5, 5.0 Hz, 1H), 2.38 (s, 3H). ^{13}C NMR (125 MHz, $CDCl_3$, 23 ºC, δ): 182.3, 152.9, 148.6, 148.5, 139.7, 135.0, 132.2, 128.7, 127.6, 127.4, 124.7, 122.6, 121.8, 119.5, 24.9. These spectroscopic data correspond to previously reported data.[4]

Potassium tetra(1*H*-pyrazol-1-yl)borate (S2)

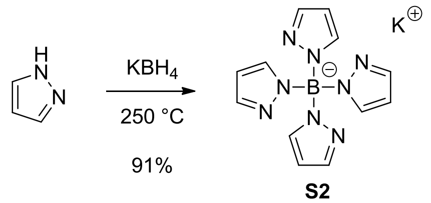

S2

Based on a reported procedure:[5] As solids, KBH_4 (7.00 g, 0.130 mol, 1.00 equiv) and pyrazole (44.2 g, 0.649 mol, 5.00 equiv) were combined in a round-bottom flask equipped with a reflux condenser under a N_2 atmosphere. This mixture was heated at 250 ºC for 16 hours. The melt was then cooled to 23 °C. The residue was dissolved in methanol (200 mL). The solution was added to diethyl ether (600 mL). A precipitate formed that was isolated by filtration. The precipitate was washed with additional diethyl ether (2 × 100 mL), affording 37.6 g of the title compound as a colorless solid (91% yield).

Melting Point: 248–249 ºC. NMR Spectroscopy: ^{1}H NMR (600 MHz, D_2O, 23 ºC, δ): 7.49 (s,

4H), 7.19 (d, J = 2.0 Hz, 4H), 6.14 (s, 4H). ^{13}C NMR (125 MHz, D_2O, 23 ºC, δ): 138.9, 132.8, 102.4. ^{11}B NMR (100 MHz, D_2O, 23 ºC, δ): –1.3. Mass Spectrometry: LRMS-FIA (m/z): calcd for $C_{12}H_{12}BN_8$ $[M - K]^-$, 279.1; found, 279.1. These spectroscopic data correspond to previously reported data.[5]

Benzo[*h*]quinolinyl (tetrapyrazolylborate)palladium (3)

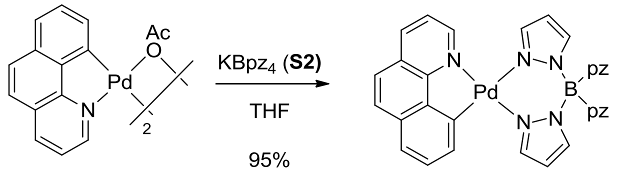

Based on a reported procedure:[6] To benzo[*h*]quinolinyl palladium acetate dimer (**S1**) (11.0 g, 16.0 mmol, 1.00 equiv) in a round-bottom flask open to air in THF (350 mL) was added potassium tetra(1*H*-pyrazol-1-yl)borate ($KBpz_4$) (**S2**) (10.2 g, 32.0 mmol, 2.00 equiv) in one portion at 23 ºC. The solution was stirred at 23 °C for 12 hours and then concentrated *in vacuo*. The residue was dissolved in CH_2Cl_2 (500 mL), filtered through Celite eluting with additional CH_2Cl_2 (100 mL), and the solution was concentrated *in vacuo*. The residual solid was triturated with diethyl ether (300 mL), collected by filtration, and subsequently dried to afford 18.0 g of the title compound as a light yellow solid (95%).

NMR Spectroscopy: ^{1}H NMR (400 MHz, $CDCl_3$, 23 ºC, δ): 8.50 (d, J = 4.8 Hz, 1H), 8.19 (d, J = 8.6 Hz, 1H), 7.95 (br s, 1H), 7.89 (br s, 1H), 7.75 (br s, 1H), 7.69 (d, J = 8.6 Hz, 1H), 7.66 (br s, 1H), 7.60 (br s, 1H), 7.57 (d, J = 7.6 Hz, 1H), 7.48 (d, J = 8.6 Hz, 1H), 7.43 (dd, J = 7.6, 7.6 Hz, 1H), 7.36 (dd, J = 7.0, 5.7 Hz, 1H), 7.30 (d, J = 7.6 Hz, 1H), 6.92 (br s, 1H), 6.43 (br s, 2H), 6.29 (br s, 1H), 6.01 (br s, 1H). ^{1}H NMR (400 MHz, $CDCl_3$, –25 ºC, δ): 8.46 (d, J = 5.1 Hz, 1H), 8.10 (d, J = 8.1 Hz, 1H), 7.94 (s, 1H), 7.89 (s, 1H), 7.75 (s, 1H), 7.67 (d, J = 2.6 Hz, 1H), 7.60 (d, J = 9.0, 1H), 7.55–7.52 (m, 3H), 7.43 (dd, J = 7.5, 7.5 Hz, 1H), 7.38 (d, J = 6.5 Hz, 1H), 7.37 (s, 1H), 7.33–7.29 (m, 2H), 6.83 (d, J = 2.1, 1H), 6.44 (d, J = 1.7, 2H), 6.30 (dd, J = 1.9, 1.9 Hz, 1H), 6.05 (s, 1H). ^{13}C NMR (125 MHz, $CDCl_3$, 23 ºC, δ): 155.5, 152.4, 148.2, 144.0 (br), 142.4 (br), 141.9 (br), 141.7, 141.1 (br), 137.5, 137.4 (br), 137.0 (br), 135.7 (br), 134.1 (br), 133.4, 132.1, 129.5, 128.6, 126.9, 123.1, 121.1, 106.3 (br), 106.2 (br), 105.3 (br). ^{13}C NMR (101 MHz, $CDCl_3$, –25 ºC, δ): 155.0, 152.3, 148.0, 144.0, 142.4, 142.1, 141.3, 141.3, 137.5, 137.1, 136.7, 135.7, 134.0, 133.1, 131.9, 129.1, 128.5, 126.6, 123.1, 123.1, 121.1, 106.5, 106.3, 105.5, 105.2. Anal: calcd for $C_{25}H_{20}BN_9Pd$: C, 53.27; H, 3.58; N, 22.36; found: C, 53.09; H, 3.64; N, 22.17.

1,1'-(Phenyl-λ^3-iodanediyl)bis(4-cyanopyridinium) bis(trifluoromethanesulfonate) (4)

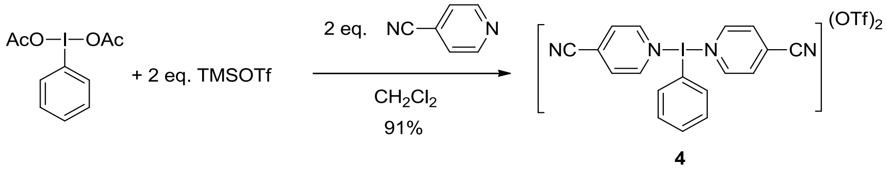

Based on a reported procedure:[7] All manipulations were carried out in a dry box under a N_2 atmosphere. To (diacetoxyiodo)benzene (10.00 g, 31.1 mmol, 1.00 equiv) dissolved in CH_2Cl_2 (250 mL) in a round-bottom flask was added TMSOTf (14.0g, 63.0 mmol, 2.03 equiv) dropwise over 5 minute at 23 °C. 4-Cyanopyridine (6.46 g, 62.1 mmol, 2.00 equiv) in CH_2Cl_2 (20 mL) was added to the solution dropwise over 5 minutes to give a colorless precipitate and the mixture was stirred for 30 min vigorously at 23 °C. The solid was filtered off and washed with CH_2Cl_2 (3 × 30 mL) and subsequently dried under vacuum to afford 20.1 g of the title compound as a colorless solid (91%).

NMR Spectroscopy: ^{1}H NMR (500 MHz, CD_3CN, 23 ºC, δ): 9.21 (d, J = 5.3 Hz, 4H), 8.74 (d, J = 7.5 Hz, 2H), 8.11 (d, J = 6.4 Hz, 4H), 7.87 (t, J = 7.5 Hz, 1H), 7.71 (dd, J = 8.0, 8.0 Hz, 2H). ^{13}C NMR (125 MHz, CD_3CN, 23 ºC, δ): 150.1, 137.4, 136.8, 134.7, 132.4, 128.8, 124.0, 121.9 (q, J = 319 Hz, triflate), 115.4. ^{19}F NMR (375 MHz, CD_3CN, 23 ºC, δ): –77.5. Anal: calcd for $C_{20}H_{13}F_6IN_4O_6S_2$: C, 33.82; H, 1.84; N, 7.89; found: C, 33.63; H, 1.67; N, 7.68.

Benzo[*h*]quinolinyl (tetrapyrazolylborate) Pd(IV) 4-cyanopyridine trifluoromethanesulfonate (5)

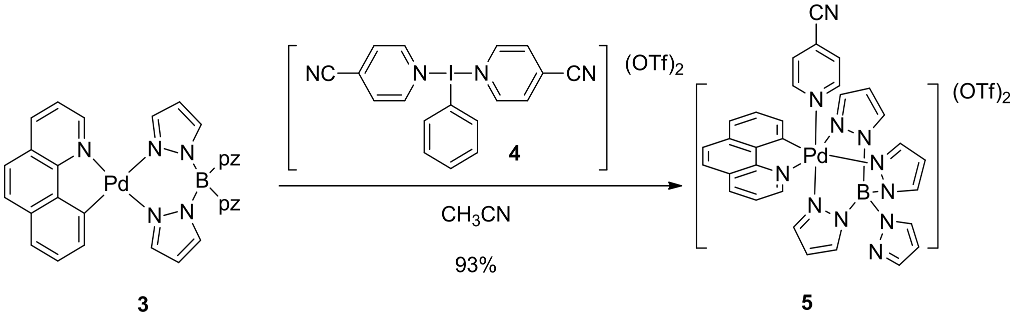

All manipulations were carried out in a dry box under a N_2 atmosphere. To benzo[*h*]quinolinyl (tetrapyrazolylborate)palladium (**3**) (10.00 g, 17.7 mmol, 1.00 equiv) in a round-bottom flask in CH_3CN (100 mL) at 23 ºC was added 1,1'-(phenyl-λ^3-iodanediyl)bis(4-cyanopyridinium) bis(trifluoromethanesulfonate) (**4**) (13.0 g, 18.1 mmol, 1.02 equiv). After stirring for 30 minutes, the reaction mixture was concentrated *in vacuo*. The resulting residue was triturated with THF (3 × 70 mL) and collected by filtration as a light brown solid. The solid was re-dissolved in CH_3CN (50 mL), and the solution was concentrated *in vacuo* to afford 16.0 g of the title compound as a brown solid (93%).

NMR Spectroscopy: ^{1}H NMR (500 MHz, CD_3CN, 23 ºC, δ): 9.10 (d, *J* = 8.6 Hz, 1H), 8.97 (s, 1H), 8.97 (d, *J* = 9.6 Hz, 1H), 8.49 (d, *J* = 8.5 Hz, 1H), 8.41 (d, *J* = 9.6 Hz, 1H), 8.39 (d, *J* = 7.5 Hz, 1H), 8.35 (s, 1H), 8.26 (d, *J* = 2.1 Hz, 1H), 8.09 (s, 1H), 8.06 (d, *J* = 2.1 Hz, 1H), 8.05 (s, 1H), 7.97 (dd, *J* = 7.0, 7.0 Hz, 1H), 7.85 (dd, *J* = 8.0, 8.0 Hz, 1H), 7.77–7.70 (m, 5H), 7.43 (d, *J* = 2.3 Hz, 1H), 6.86 (s, 1H), 6.82 (dd, *J* = 2.7, 2.7 Hz, 1H), 6.80 (s, 1H), 6.21 (d, *J* = 2.1 Hz, 1H), 6.10 (dd, *J* = 2.1, 2.1 Hz, 1H). ^{13}C NMR (125 MHz, CD_3CN, 23 ºC): 169.5, 153.5, 152.3, 148.2, 144.5, 144.4, 144.1, 144.0, 142.7, 140.8, 140.4, 140.0, 139.8, 137.7, 134.0, 133.7, 133.5, 132.0, 131.7, 130.4, 130.3, 128.7, 127.7, 127.0, 121.9 (q, *J* = 319 Hz, triflate), 115.1, 112.2, 110.5, 110.5, 110.4, 109.6. ^{19}F NMR (375 MHz, CD_3CN, 23 ºC, δ): –77.5. Anal: calcd for $C_{33}H_{24}BF_6N_{11}O_6PdS_2$: C, 41.03; H, 2.50; N, 15.95; found: C, 40.78; H, 2.47; N, 15.67.

Benzo[*h*]quinolinyl (tetrapyrazolylborate) Pd(IV) 4-picoline trifluoromethanesulfonate (6)

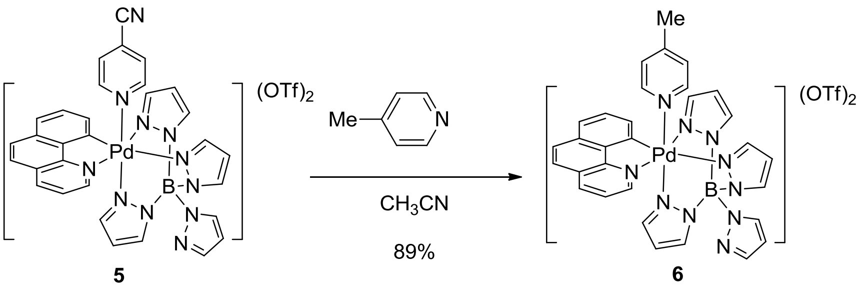

All manipulations were carried out in a dry box under a N_2 atmosphere. To benzo[*h*]quinolinyl (tetrapyrazolylborate) Pd(IV) 4-cyanopyridine trifluoromethanesulfonate (**5**) (5.00 g, 5.16 mmol, 1.00 equiv) in a round-bottom flask in CH_3CN (15 mL) at 23 ºC was added 4-picoline (769 mg, 8.26 mmol, 1.60 equiv). After stirring for 2 minutes the reaction mixture was added dropwise over 5 minutes to 200 mL of diethyl ether (200 mL) while stirring vigorously at 23 ºC. The resulting precipitate was collected by filtration as a light brown solid. The solid was re-dissolved in CH_3CN (10 mL), and the solution was concentrated *in vacuo* to afford 4.40 g of the title compound as a brown solid (89%).

NMR Spectroscopy: ^{1}H NMR (500 MHz, CD_3CN, 23 ºC, δ): 9.09 (d, *J* = 8.5 Hz, 1H), 8.97 (d, *J* = 8.5 Hz, 1H), 8.97 (s, 1H), 8.47 (d, *J* = 9.6 Hz, 1H), 8.40 (d, *J* = 8.5 Hz, 1H), 8.38 (d, *J* = 8.6 Hz, 1H), 8.27 (d, *J* = 9.6 Hz, 2H), 8.08 (s, 1H), 8.05 (d, *J* = 2.1 Hz, 1H), 7.98–7.95 (m, 2H), 7.84 (dd, *J* = 8.1, 8.1 Hz, 1H), 7.73 (d, *J* = 8.5 Hz, 1H), 7.40 (d, *J* = 3.2 Hz, 1H), 7.32 (d, *J* = 7.5 Hz, 2H), 7.20 (d, *J* = 6.4 Hz, 2H), 6.85 (dd, *J* = 2.1, 2.1 Hz, 1H), 6.81 (s, 2H), 6.20 (d, *J* = 2.1 Hz, 1H), 6.09 (dd, *J* = 2.1, 2.1 Hz, 1H), 2.38 (s, 3H). ^{13}C NMR (125 MHz, CD_3CN, 23 ºC, δ): 169.2, 158.7, 152.0, 151.1, 148.5, 144.4, 144.3, 144.1, 143.9, 142.6, 140.6, 140.2, 139.9, 139.6, 137.7, 134.3, 133.5, 133.4, 131.7, 130.4, 130.2, 130.0, 128.6, 126.9, 121.9 (q, *J* = 319 Hz, triflate), 112.0, 110.3, 110.3, 109.6, 21.2. ^{19}F NMR (375 MHz, CD_3CN, 23 ºC, δ): –77.5. Anal: calcd for $C_{33}H_{27}BF_6N_{10}O_6PdS_2$: C, 41.50; H, 2.85; N, 14.67; found: C, 41.45; H, 2.72; N, 14.41.

Synthesis of Pd(IV) fluoride complex 7

Benzo[*h*]quinolinyl (tetrapyrazolylborate) Pd(IV) fluoride trifluoromethanesulfonate (7)

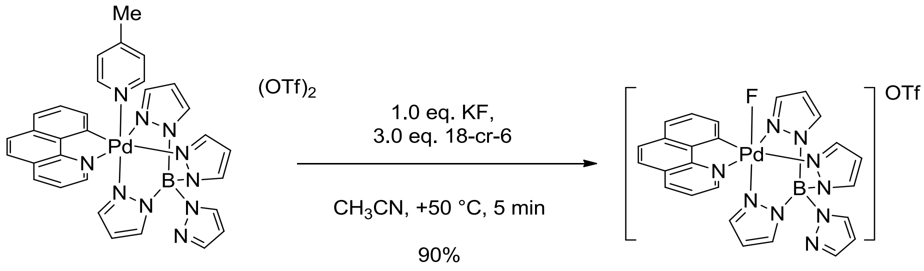

6 **7**

In a glove box, to benzo[*h*]quinolinyl (tetrapyrazolylborate) Pd(IV) 4-picoline trifluoromethanesulfonate (**6**) (284 mg, 0.297 mmol, 1.00 equiv) dissolved in CH_3CN (15 mL) in a soda lime glass bottle was added KF (17.3 mg, 0.297 mmol, 1.00 equiv) and 18-crown-6 (235 mg, 0.891 mmol, 3.00 equiv) in one portion at 23 ºC. The bottle was sealed, taken out of the glove box, sonicated at 23 °C for 5 minutes, immersed in an oil bath heated at 50 °C for 5 minutes while vigorously stirring the suspension. CH_3CN (10 mL) was added to the solution, and the solution was filtered through Celite, eluting with additional CH_3CN (10 mL). The filtrate was concentrated *in vacuo*. The residue was triturated with THF (3 × 15 mL) and subsequently dried *in vacuo* to afford 195 mg of the title compound as an orange solid (90%).

NMR Spectroscopy: ^{1}H NMR (500 MHz, CD_3CN, 23 ºC, δ): 9.01 (d, J = 5.3 Hz, 1H), 8.96 (d, J = 8.5 Hz, 1H), 8.79 (d, J = 3.2 Hz, 1H), 8.32 (s, 2H), 8.29 (d, J = 9.6 Hz, 1H), 8.27 (d, J = 2.1 Hz, 1H), 8.23 (d, J = 8.6 Hz, 1H), 8.21 (d, J = 8.6 Hz, 1H), 8.16 (s, 1H), 7.97 (d, J = 5.4 Hz, 1H), 7.96 (d, J = 6.4 Hz, 1H), 7.83 (dd, J = 8.1, 8.1 Hz, 1H), 7.62 (d, J = 8.5 Hz, 1H), 6.79–6.72 (m, 4H), 6.54 (d, J = 2.1 Hz, 1H), 6.11 (s, 1H). ^{13}C NMR (125 MHz, DMSO-d_6, 23 ºC, δ): 165.0, 149.4, 149.2, 149.4, 149.2, 143.4, 143.0, 142.7, 142.7, 142.2, 138.5, 137.6, 137.6, 137.0, 136.7, 134.8, 132.1, 130.3, 129.6, 127.6, 127.6, 126.4, 120.7 (q, J = 323 Hz, triflate), 109.9, 109.6, 108.5, 108.5. ^{19}F NMR (375 MHz, CD_3CN, 23 ºC, δ): –77.5 (s), –319.5 (s). Anal: calcd for $C_{26}H_{20}BF_4N_9O_3PdS$: C, 42.67; H, 2.75; N, 17.23; found: C, 42.95; H, 2.95; N, 17.04.

Thermal stability of **7**: **7** was placed in a vial and heated for 24 hours at 100 ºC under dynamic vacuum (10^{-4} Torr). The solid was analyzed by ^{1}H and ^{19}F NMR spectroscopy, and showed no decomposition.

Tolerance of **7** toward water: 2.4 mg of **7** (3.3 μmol) and THF (2.0 μL) (internal standard) were dissolved in CD_3CN (0.55 mL) in an NMR tube. D_2O (61 μL) was added to the solution. The solution was kept at +23 ºC for 3 hours and monitored by ^{1}H and ^{19}F NMR spectroscopy, which showed no decomposition (Figure S1).

Figure S1. NMR Spectra of 7 in 10% aqueous acetonitrile solution

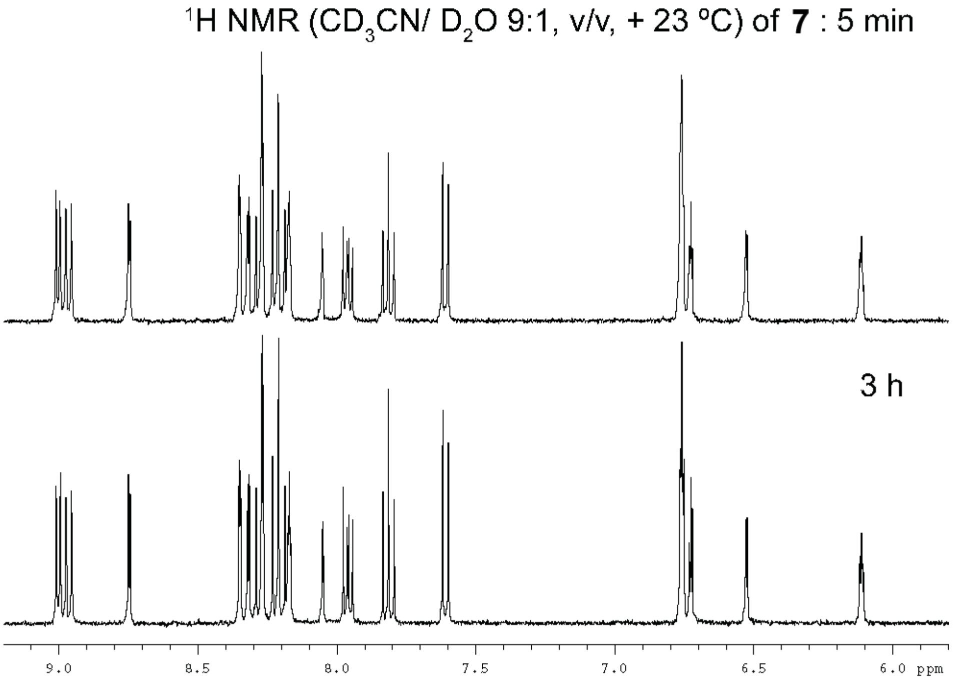

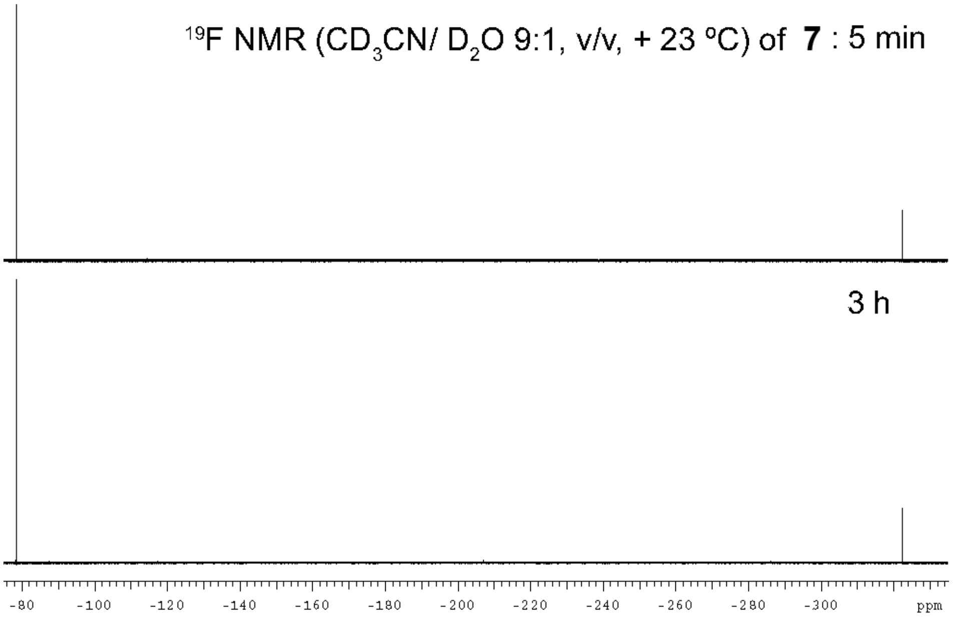

Alternative synthesis of 7

Bis(pyridinio-1)iodobenzene bis(trifluoroacetate) (S3)

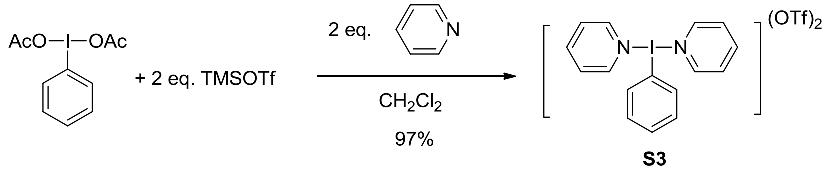

Based on a reported procedure:[7] All manipulations were carried out in a dry box under a N_2 atmosphere. To (diacetoxyiodo)benzene (15.0 g, 46.6 mmol, 1.00 equiv) dissolved in 300 mL CH_2Cl_2 in a round-bottom flask was added TMSOTf (20.7 g, 93.1 mmol, 2.00 equiv) dropwise over 5 minute at 23 °C. Pyridine (7.37 g, 7.53 mL, 93.1 mmol, 2.00 equiv) in 50 mL CH_2Cl_2 was added to the solution dropwise to give a white precipitate and the mixture was stirred for 30 min vigorously at 23 °C. The solid was filtered off and washed with 50 mL CH_2Cl_2 three times and subsequently dried under vacuum to afford 29.9 g of the title compound as a colorless solid (97%).

NMR Spectroscopy: ^{1}H NMR (500 MHz, CD_3CN, 23 ºC, δ): 8.97 (d, J = 5.4 Hz, 4H), 8.64 (d, J = 8.0 Hz, 2H), 8.39 (t, J = 9.6 Hz, 2H), 7.84–7.80 (m, 5H), 7.67 (t, J = 8.0 Hz, 2H). ^{19}F NMR (375 MHz, CD_3CN, 23 ºC, δ): –77.5 (s). ^{13}C NMR (125 MHz, CD_3CN, 23 ºC, δ): 148.59, 146.17, 136.75, 136.18, 134.40, 129.89, 124.67, 121.88 (q, J = 319.4 Hz, triflate). These spectroscopic data correspond to previously reported data.[7]

Benzo[*h*]quinolinyl (tetrapyrazolylborate) Pd(IV) pyridine trifluoromethanesulfonate (3)

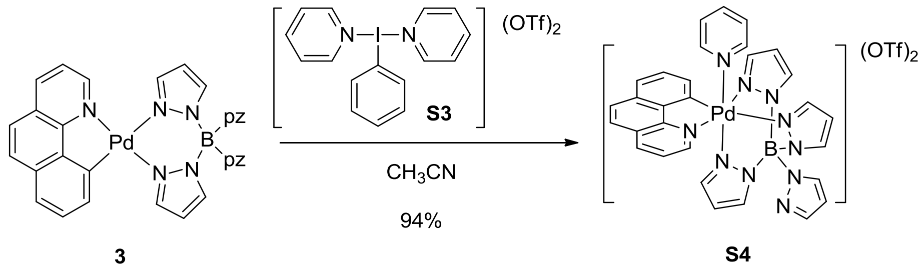

All manipulations were carried out in a dry box under a N_2 atmosphere. To benzo[*h*]quinolinyl (tetrapyrazolylborate)palladium (**3**) (10.00 g, 17.7 mmol, 1.00 equiv) in CH_3CN (50 mL) at 23 ºC was added bis(pyridinio-1)iodobenzene bis(trifluoroacetate) (**S3)** (12.0 g, 18.1 mmol, 1.02 equiv). After stirring for 20 min the reaction mixture was concentrated *in vacuo*. The resulting residue was triturated with THF (30 mL) and collected on a frit by filtration as a light brown solid. The collected solid was further washed with THF (30 mL) three times and dried *in vacuo*. The solid was redissolved in 30 mL CH_3CN and volatiles including residual THF were removed *in vacuo* to afford 18.0 g of the title compound as a brown solid (94%).

NMR Spectroscopy: ^{1}H NMR (500 MHz, CD_3CN, 23 ºC, δ): 9.07 (d, J = 7.5 Hz, 1H), 8.98 (d, J = 2.1 Hz, 1H), 8.96 (d, J = 6.4 Hz, 1H), 8.49 (d, J = 9.0 Hz, 1H), 8.43 (d, J = 9.0 Hz, 1H), 8.37 (d, J = 7.5 Hz, 1H), 8.24 (d, J = 2.1 Hz, 2H), 8.11–7.94 (m, 5H), 7.84 (t, J = 8.0 Hz, 1H), 7.74 (d, J = 8.6 Hz, 1H), 7.51 (s, 1H), 7.50 (s, 1H), 7.39–7.36 (m, 3H), 6.85 (t, J = 2.1 Hz, 1H), 6.80 (s, 2H), 6.20 (d, J = 2.1 Hz, 1H), 6.09 (t, J = 2.1 Hz, 1H). ^{19}F NMR (375 MHz, CD_3CN, 23 ºC, δ): −77.5. ^{13}C NMR (125 MHz, $CDCl_3$, 23 ºC, δ): 169.38, 152.20, 152.08, 148.41, 144.95, 144.40, 144.32, 144.14, 143.98, 142.64, 140.63, 140.24, 139.85, 139.62, 137.71, 134.24, 133.58, 133.40, 131.65, 130.23, 130.07, 129.97, 128.63, 126.87, 121.88 (q, J = 319.4 Hz, triflate), 112.07, 110.34, 109.57. Anal: calcd for $C_{32}H_{25}BF_6N_{10}O_6PdS_2$: C, 40.85; H, 2.68; N, 14.89; found: C, 40.84; H, 2.81; N, 14.89.

Benzo[*h*]quinolinyl (tetrapyrazolylborate) Pd(IV) fluoride trifluoromethanesulfonate (7)

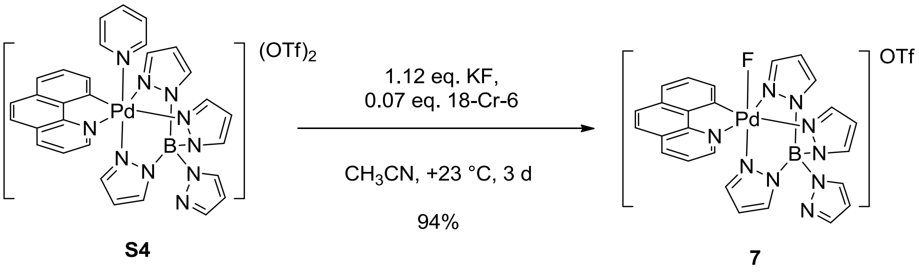

In a glovebox, to benzo[h]quinolinyl (tetrapyrazolylborate) Pd(IV) pyridine trifluoromethanesulfonate (**S4**) (7.80 g, 8.29 mmol, 1.00 equiv) dissolved in 150 mL CH_3CN was added KF (0.54 g, 9.26 mmol, 1.12 equiv) and 18-crown-6 (0.16 g, 0.62 mmol, 0.07 equiv) in one portion at room temperature. After the solution was vigorously stirred for 3 days at room temperature and another 350 mL of CH_3CN was added to the reaction solution. The flask was sealed, taken out of the glove box, immersed in a water bath heated at +50 °C until the turbid solution became clear, which took less than 30 min, and the solution was filtered through Celite eluting with 100 mL of dry CH_3CN. The filtrate was concentrated *in vacuo*. The residue was triturated with dry THF (3 × 50 mL), filtered off, and subsequently dried *in vacuo* to afford 5.80 g of the title compound as an orange solid (94%).

Synthesis of aryl palladium complexes (15 and 20, examples of 9)

[{(4-Methoxyphenyl)sulfonyl}imino]phenyliodinane (S5)

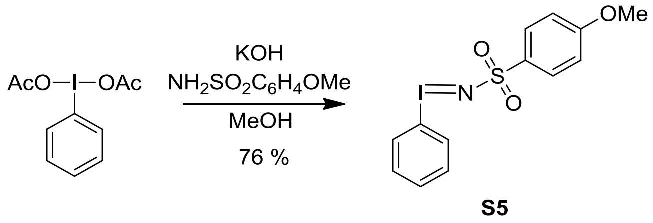

Based on a reported procedure:[8,9] To *p*-methoxybenzenesulfonamide (5.00 g, 26.7 mmol, 1.00 equiv) in a round-bottom flask open to air in methanol (100 mL) at 23 ºC was added potassium hydroxide (3.75 g, 66.8 mmol, 2.50 equiv). The reaction mixture was stirred at 23 ºC for 10 minutes and subsequently cooled to 0 ºC. To the reaction mixture at 0 ºC was added iodobenzene diacetate (8.60 g, 26.7 mmol, 1.00 equiv). The reaction mixture was stirred at 0 ºC for 10 minutes and further stirred at 23 ºC for 2.0 hours. The reaction mixture was poured into cold water (700 mL) and kept at 0 ºC for 4 hours. The suspension was filtered and the filter cake was washed with water (2 × 200 mL) and methanol (2 × 200 mL) to afford 7.90 g of the title compound as a colorless solid (76% yield).

NMR Spectroscopy: ^{1}H NMR (500 MHz, DMSO-d_6, 23 ºC, δ): 7.70 (d, J = 7.5 Hz, 2H), 7.49–7.44 (m, 3H), 7.32–7.28 (m, 2H), 6.78 (d, J = 8.5 Hz, 2H), 3.74 (s, 3H). ^{13}C NMR (125 MHz, DMSO-d_6, 23 ºC, δ): 160.6, 136.9, 133.2, 130.5, 130.2, 128.0, 117.0, 113.4, 55.4. These spectroscopic data correspond to previously reported data.[9]

Benzo[*h*]quinolinyl palladium chloro dimer (S6)

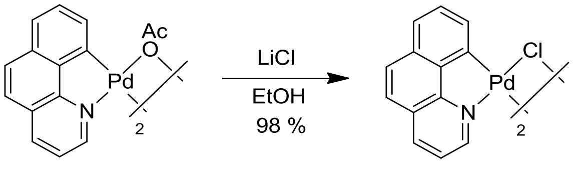

Based on a reported procedure:[10] To benzo[*h*]quinolinyl palladium acetate dimer (**S1**) (4.27 g, 12.4 mmol, 1.00 equiv) in a round-bottom flask open to air in EtOH (100 mL) at 0 ºC was added lithium chloride (10.5 g, 24.8 mmol, 20.0 equiv). The reaction mixture was warmed to 23 ºC and stirred for 1.0 hours. The reaction mixture was filtered and the filter cake was washed with water (3 × 100 mL), MeOH (2 × 100 mL), and diethyl ether (100 mL) to afford 3.89 g of the title compound as a pale yellow solid (98% yield).

NMR Spectroscopy: ^{1}H NMR (500 MHz, DMSO-d_6, 23 ºC, δ): 9.44 (d, J = 4.5 Hz, 1H), 8.72 (br, 0.25H), 8.67 (d, J = 7.5 Hz, 1H), 8.61 (br, 0.25H), 8.22 (d, J = 7.0 Hz, 1H), 7.91 (d, J = 9.0 Hz, 1H), 7.86–7.74 (m, 3H), 7.73 (br, 0.25H), 7.60 (br, 0.25H), 7.53 (dd, J = 7.5, 7.0 Hz 1H), 7.38 (br, 0.25H); ^{13}C NMR (125 MHz, DMSO-d_6, 23 ºC, δ): 153.9, 152.2, 150.7, 150.6, 148.0, 141.7, 139.9, 134.4, 130.8, 129.6, 129.4, 127.5, 125.1, 124.4, 123.0, 122.9. Note: The complicated ^{1}H and ^{13}C NMR spectra are probably due to a mixture of the title compound and solvent adduct in DMSO-d_6. The title compound was not soluble in non-coordinating solvents.

Chloro palladium complex (S7)

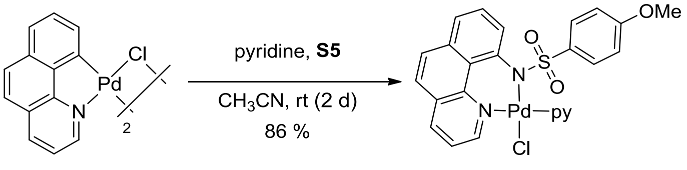

S6 **S7**

Based on a reported procedure:[11] In a glove box under a N_2 atmosphere, to chloropalladium dimer (**S6**) (6.00 g, 18.7 mmol, 1.00 equiv) in a round-bottom flask in CH_3CN (100 mL) at 23 ºC was added pyridine (6.06 mL, 75.0 mmol, 4.00 equiv) and [{(4-methoxyphenyl)sulfonyl}imino]phenyliodinane (**S5**) (10.9 g, 28.1 mmol, 1.50 equiv). The reaction mixture was stirred at 23 ºC for 48 hours and subsequently taken out of the glove box. The reaction mixture was filtered and the filter cake was washed with diethyl ether (3 × 30 mL) to afford 9.70 g of the title compound as a yellow solid (86% yield).

NMR Spectroscopy: ^{1}H NMR (500 MHz, $CDCl_3$, 23 ºC, δ): 9.21 (dd, J = 5.2, 1.5 Hz, 1H), 9.01–8.99 (m, 2H), 8.08 (dd, J = 7.9, 1.8 Hz, 1H), 7.88–7.73 (m, 5H), 7.47–7.43 (m, 3H), 7.35 (dd, J = 7.9, 5.5 Hz, 1H), 7.11–7.08 (m, 2H), 6.19–6.15 (m, 2H), 3.56 (s, 3H); ^{13}C NMR (125 MHz, $CDCl_3$, 23 ºC, δ): 160.9, 154.2, 152.6, 141.9, 139.0, 138.6, 138.4, 136.0, 134.3, 130.4, 129.8, 128.4, 128.1, 127.7, 126.8, 125.6, 125.0, 124.2, 122.1, 112.4, 55.4. These spectroscopic data correspond to previously reported data.[11]

Acetato palladium complex (8)

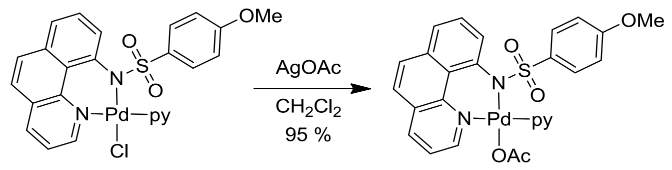

S7 **8**

To chloro palladium complex (**S7**) (5.00 g, 8.34 mmol, 1.00 equiv) in a round-bottom flask fitted with a reflux condenser open to air in CH_2Cl_2 (300 mL) at 23 ºC was added AgOAc (4.87 g, 29.2 mmol, 3.50 equiv). The suspension was stirred at 40 ºC for 3 hours. After cooling to 23 ºC, the suspension was filtered through a plug of Celite, eluting with additional CH_2Cl_2 (50 mL). The filtrate was concentrated in vacuo and the residue was triturated with diethyl ether (100 mL). The solid was collected by filtration and washed with diethyl ether (2 × 50 mL) to afford 5.07 g of the title compound as a yellow solid (95% yield).

NMR Spectroscopy: ^{1}H NMR (500 MHz, $CDCl_3$, 23 ºC, δ): 8.93 (d, J = 5.5 Hz, 2H), 8.70 (d, J = 5.5 Hz, 1H), 8.01 (d, J = 7.9 Hz, 1H), 7.83 (d, J = 6.7 Hz, 1H), 7.80 (t, J = 7.6 Hz, 1H), 7.74–7.68 (m, 3H), 7.41–7.36 (m, 3H), 7.27 (dd, J = 7.6, 5.2 Hz, 1H), 7.15 (d, J = 8.5 Hz, 2H), 6.13 (d, J = 8.5 Hz, 2H), 3.48 (s, 3H), 1.78 (s, 3H); ^{13}C NMR (125 MHz, $CDCl_3$, 23 ºC, δ): 177.4, 160.7,

151.6, 151.2, 141.7, 139.0, 138.4, 138.2, 135.8, 134.4, 130.1, 129.9, 128.9, 128.1, 127.3, 126.7, 125.5, 124.8, 124.0, 121.8, 112.3, 55.2, 23.8. Anal: calcd for $C_{27}H_{23}N_3O_5PdS$: C, 53.34; H, 3.81; N, 6.91; found: C, 53.31; H, 3.69; N, 6.89.

1-(4-Bromophenyl)-3-((4-methoxyphenyl)amino)propan-1-one (S8)

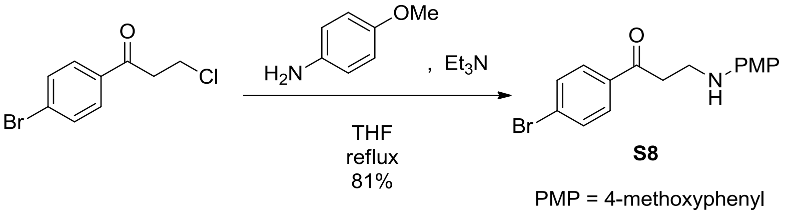

To 1-(4-bromophenyl)-3-chloropropan-1-one (43.4 g, 175 mmol, 1.00 equiv) and 4-methoxyaniline (23.8 g, 193 mmol, 1.10 equiv) in THF (250 mL) in a round-bottom flask fitted with a reflux condenser open to air was added triethylamine (21.3 g, 29.3 mL, 210 mmol, 1.20 equiv). The reaction mixture was heated at reflux for 4 hours and then cooled. The reaction mixture was poured into H_2O (400 mL). EtOAc (300 mL) was added and the organic phase was collected, washed with brine (150 mL), dried with Na_2SO_4, and concentrated *in vacuo*. The residual solid was triturated with diethyl ether (100 mL), collected by filtration, and dried *in vacuo* to afford 47.4 g of the title compound as a light green solid (81% yield).

R*f* = 0.30 (hexanes/EtOAc 3:1 (v/v)). NMR Spectroscopy: ^{1}H NMR (500 MHz, $CDCl_3$, 23 °C, δ): 7.82–7.79 (m, 2H), 7.61–7.59 (m, 2H), 6.80–6.77 (m, 2H), 6.63–6.60 (m, 2H), 3.83 (br s, 1H), 3.75 (s, 3H), 3.56 (t, J = 6.3 Hz, 2H), 3.22 (t, J = 6.2 Hz, 2H). ^{13}C NMR (125 MHz, $CDCl_3$, 23 °C, δ): 198.5, 152.6, 141.9, 135.6, 132.1, 129.7, 128.7, 115.1, 114.8, 55.9, 39.9, 37.8. HRMS-FIA (m/z): calcd for $C_{16}H_{16}BrNNaO_2$ $[M + Na]^+$, 356.0262; found, 356.0254.

4-(4-Bromophenyl)-1-(4-methoxyphenyl)-5,6-dihydropyridin-2(1*H*)-one (10)

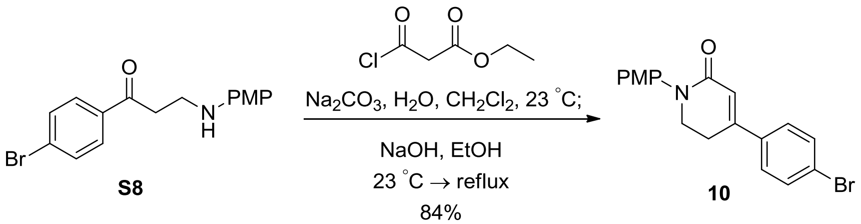

To 1-(4-bromophenyl)-3-((4-methoxyphenyl)amino)propan-1-one (**S8**) (19.7 g, 58.8 mmol, 1.00 equiv) suspended in CH_2Cl_2 (200 mL) in a round-bottom flask open to air was added ethyl 3-chloro-3-oxopropanoate (9.32 g, 7.84 mL, 58.8 mmol, 1.00 equiv) and a saturated aqueous solution of Na_2CO_3 (100 mL). The reaction mixture was stirred at 23 °C for 1 hour and then poured onto H_2O (100 mL) in a separatory funnel. The separatory funnel was shaken and the organic phase collected and concentrated *in vacuo*. The residue was dissolved in CH_2Cl_2 (200 mL) and ethyl 3-chloro-3-oxopropanoate (4.66 g, 3.92 mL, 29.4 mmol, 0.500 equiv) and a

saturated aqueous solution of Na_2CO_3 (100 mL) were added. The reaction mixture was stirred at 23 ºC for 1 hour and then poured onto H_2O (100 mL) in a separatory funnel. The separatory funnel was shaken and the organic phase collected. The aqueous phase was extracted from with CH_2Cl_2 (100 mL). The combined organic phases were washed with brine (100 mL), dried with Na_2SO_4, and concentrated *in vacuo*. The residue was dissolved in EtOH (400 mL). NaOH (9.41 g, 235 mmol, 4.00 equiv) was added, and the reaction mixture was stirred at 23 ºC for 15 minutes and then heated at reflux for 30 minutes. The reaction mixture was cooled to 23 ºC and then poured into an Erlenmeyer flask containing H_2O (600 mL). The reaction suspension was cooled to 10 ºC in a refrigerator and the precipitate was collected on a frit. Azeotropic removal of water by suspending the solid in benzene and concentration *in vacuo* (3 × 50 mL) afforded 17.6 g of the title compound as a beige yellow solid (84% yield).

R*f* = 0.20 (hexanes/EtOAc 2:1 (v/v)). NMR Spectroscopy: ^1H NMR (500 MHz, $CDCl_3$, 23 ºC, δ): 7.57–7.54 (m, 2H), 7.42–7.40 (m, 2H), 7.28–7.24 (m, 2H), 6.94–6.91 (m, 2H), 6.43 (t, *J* = 1.0 Hz, 1H), 3.93 (t, *J* = 6.8 Hz, 2H), 3.81 (s, 3H), 2.90 (dt, *J* = 6.8, 1.0 Hz, 2H). ^{13}C NMR (125 MHz, $CDCl_3$, 23 ºC, δ): 164.8, 157.9, 148.7, 136.4, 135.5, 132.2, 127.4, 126.5, 124.1, 121.1, 114.4, 55.6, 48.9, 27.1. HRMS-FIA (m/z): calcd for $C_{18}H_{17}BrNO_2[M + H]^+$, 358.0443; found, 358.0440.

(*R*)-4-(4-Bromophenyl)-1-(4-methoxyphenyl)piperidin-2-one (11)

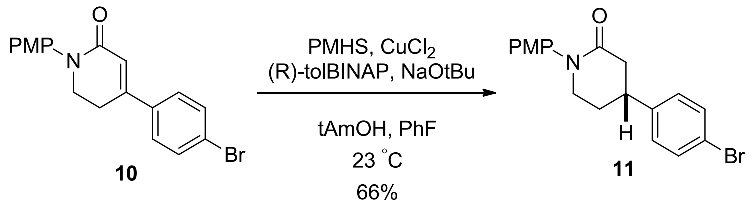

Following procedures previously described for a similar transformation:[12] To 4-(4-bromophenyl)-1-(4-methoxyphenyl)-5,6-dihydropyridin-2(1*H*)-one (**10**) (28.8 g, 80.0 mmol, 1.00 equiv) suspended in PhF (224 mL) in a round-bottom flask open to air was added *t*-amyl alcohol (113 g, 141 mL, 1.29 mol, 16.0 equiv), polymethylhydrosiloxane (PMHS) (77.0 g, 76.5 mL, 1.29 mol, 16.0 equiv of [H^-]), and (*R*)-2,2'-bis(di-*p*-tolylphosphino)-1,1'-binaphthalene ((*R*)-tol-BINAP) (1.36 g, 2.01 mmol, 0.0250 equiv) and then $CuCl_2$ (0.270 g, 2.01 mmol, 0.0250 equiv) and NaO^{t}Bu (0.773 g, 8.04 mmol, 0.100 equiv). Gas evolved and the reaction mixture turned orange and then was stirred at 23 ºC for 7 hours. The reaction mixture was poured onto a 1.0 M aqueous solution of HCl (1000 mL), and the aqueous mixture was extracted with EtOAc (2 × 500 mL). The combined organic phases were washed with brine (300 mL), dried with Na_2SO_4, and concentrated *in vacuo*. The residual solid was triturated with diethyl ether (100 mL), collected by filtration, and dried *in vacuo* to afford 19.1 g of the title compound as a colorless solid (66% yield and 88% *ee* as determined by analysis of (3*S*,4*R*)-methyl 4-(4-bromophenyl)-1-(4-methoxyphenyl)-2-oxopiperidine-3-carboxylate (**11**) (see next section and Figure S2). Racemic **11** was synthesized using the above procedures using a 1:1 mixture of (*R*)-tol-BINAP and (*S*)-tol-

BINAP.

R*f* = 0.20 (hexanes/EtOAc 1:1 (v/v)). NMR Spectroscopy: ^{1}H NMR (500 MHz, $CDCl_3$, 23 ºC, δ): 7.50–7.48 (m, 2H), 7.19–7.14 (m, 4H), 6.95–6.92 (m, 2H), 3.81 (s, 3H), 3.74 (ddd, *J* = 12.1, 10.1, 4.6 Hz, 1H), 3.61 (ddd, *J* = 12.4, 5.3, 3.9 Hz, 1H), 3.23 (dddd, *J* = 10.5, 10.5, 5.0, 3.2 Hz, 1H), 2.86 (ddd, *J* = 17.4, 5.3, 2.1 Hz, 1H), 2.64 (dd, *J* = 17.4, 11.0 Hz, 1H), 2.24–2.19 (m, 1H), 2.10 (dddd, *J* = 13.4, 10.7, 10.7, 5.3 Hz, 1H). ^{13}C NMR (125 MHz, $CDCl_3$, 23 ºC, δ): 169.3, 158.4, 142.5, 135.9, 132.0, 128.4, 127.5, 120.8, 114.7, 55.6, 50.9, 39.8, 38.4, 30.7. HRMS-FIA (m/z): calcd for $C_{18}H_{19}BrNO_2$ $[M + H]^+$, 360.0599; found, 360.0588.

(3*S*,4*R*)-Methyl 4-(4-bromophenyl)-1-(4-methoxyphenyl)-2-oxopiperidine-3-carboxylate (S9)

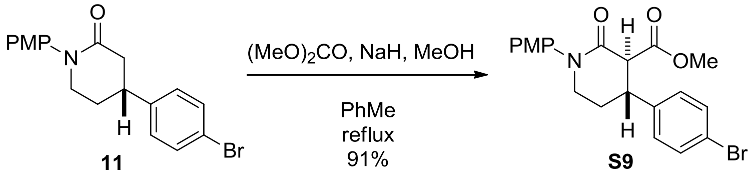

To (*R*)-4-(4-bromophenyl)-1-(4-methoxyphenyl)piperidin-2-one (**11**) (19.0 g, 53.7 mmol, 1.00 equiv) and NaH (13 g (60% by weight NaH), 0.32 mol, 6.0 equiv) in a flame-dried round-bottom flask fitted with a reflux condenser was added toluene (270 mL), MeOH (5.07 g, 6.40 mL, 158 mmol, 3.00 equiv), and dimethyl carbonate (14.3 g, 13.3 mL, 158 mmol, 3.00 equiv). The reaction mixture was heated at reflux for 4 hours, cooled to 23 ºC, and poured onto H_2O (300 mL). The aqueous layer was extracted from with EtOAc (2 × 300 mL). The combined organic phases were washed with brine (300 mL), dried with Na_2SO_4, and concentrated *in vacuo*. The residual solid was triturated with hexanes/EtOAc (150 mL, 1:1 (v/v)), collected by filtration, and dried *in vacuo* to afford 20.0 g of the title compound as a colorless solid (91% yield). Enantiomeric excess (*ee*) of the product was determined to be 88% on a Chiralpak IB column with 35% isopropanol/hexanes eluent (see Figure S2).

R*f* = 0.40 (hexanes/EtOAc 1:1 (v/v)). NMR Spectroscopy: ^{1}H NMR (500 MHz, $CDCl_3$, 23 ºC, δ): 7.49–7.46 (m, 2H), 7.21–7.18 (m, 2H), 7.15–7.13 (m, 2H), 6.93–6.90 (m, 2H), 3.88–3.80 (m, 4H), 3.70 (d, *J* = 10.5 Hz, 1H), 3.66–3.56 (m, 5H), 2.23–2.17 (m, 2H). ^{13}C NMR (125 MHz, $CDCl_3$, 23 ºC, δ): 170.3, 165.7, 158.5, 140.6, 135.2, 132.2, 128.7, 127.3, 121.4, 114.6, 56.7, 55.6, 52.6, 50.7, 42.1, 29.9. HRMS-FIA (m/z): calcd for $C_{20}H_{21}BrNO_4$$[M + H]^+$, 418.0654; found, 418.0659.

Figure S2. Enantiodiscriminating HPLC trace of S9

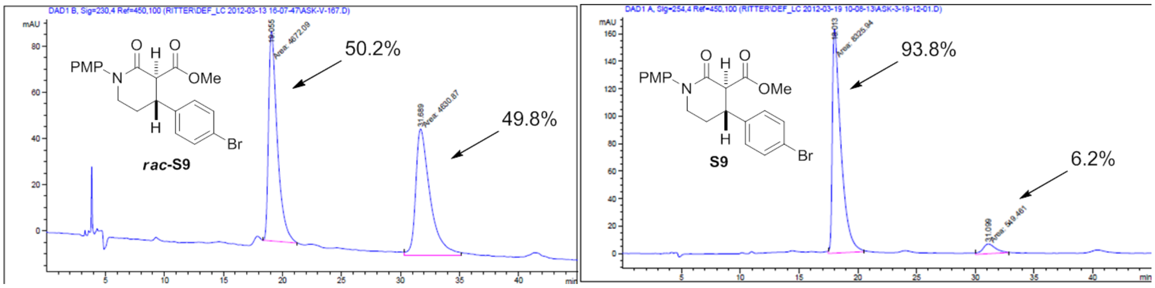

HPLC method: Chiralpak IB column with 35% isopropanol/hexanes eluent for racemic **S9** and enantioenriched **S9**. Percent of total integration listed for each peak.

((3*S*,4*R*)-4-(4-Bromophenyl)-1-(4-methoxyphenyl)piperidin-3-yl)methanol (12)

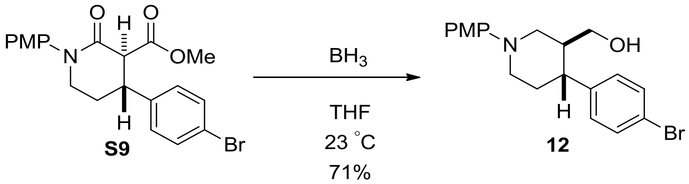

To (3*S*,4*R*)-methyl 4-(4-bromophenyl)-1-(4-methoxyphenyl)-2-oxopiperidine-3-carboxylate (**S9**) (5.33 g, 12.7 mmol, 1.00 equiv) in a 340 mL pressure tube open to air was added BH_3 (1.0 M solution in THF, 76 mL, 76 mmol, 6.0 equiv). The pressure tube was sealed and the reaction mixture was heated at 90 ºC for 18 hours and then cooled. The reaction mixture was added dropwise over 1 minute to a 1.0 M aqueous solution of NaOH (100 mL). The reaction mixture was extracted from with EtOAc (3 × 60 mL). The combined organic phases were washed with brine (50 mL), dried with Na_2SO_4, and concentrated *in vacuo*. The residue was purified by chromatography on silica gel, eluting with a gradient of 33–50% EtOAc in hexanes (v/v). The resulting solid was then triturated with pentane/diethyl ether (50 mL, 1:1 (v/v)), collected by filtration, and dried *in vacuo* to afford 4.05 g of the title compound as a colorless solid (85% yield). The enantioenriched product could be recrystallized by suspending the solid in MeOH (30 mL), heating the suspension to reflux to dissolve the solid, cooling the solution, and collecting the solid by filtration, affording 3.40 g (71% yield) of the title compound in >99% *ee* as determined on a Chiracel OD-H column with 10% isopropanol/hexanes eluent (see Figure S3).

R*f* = 0.20 (hexanes/EtOAc 2:1 (v/v)). NMR Spectroscopy: ^{1}H NMR (600 MHz, $CDCl_3$, 23 ºC, δ): 7.44–7.43 (m, 2H), 7.12–7.10 (m, 2H), 6.99–6.97 (m, 2H), 6.87–6.84 (m, 2H), 3.82 (ddd, *J* = 11.8, 3.8, 1.8 Hz, 1H), 3.78 (s, 3H), 3.61–3.58 (br m, 1H), 3.48–3.44 (br m, 1H), 3.31–3.27 (br m, 1H), 2.71 (ddd, *J* = 12.0, 12.0, 2.9 Hz, 1H), 2.59 (dd, *J* = 11.5, 11.5 Hz, 1H), 2.42 (ddd, *J* = 11.6, 11.6, 4.1 Hz, 1H), 2.15–2.09 (m, 1H), 1.95 (ddd, *J* = 25.0, 12.3, 4.1 Hz, 1H) 1.90–1.87 (m, 1H). ^{13}C NMR (125 MHz, $CDCl_3$, 23 ºC, δ): 154.1, 146.2, 143.4, 131.9, 129.4, 120.3, 119.2, 114.6, 63.9, 55.7, 55.3, 52.2, 44.2, 44.1, 34.5. HRMS-FIA (m/z): calcd for $C_{19}H_{23}BrNO_2$ $[M + H]^+$, 376.0912;

found, 376.0915.

Figure S3. Enantiodiscriminating HPLC trace of 12

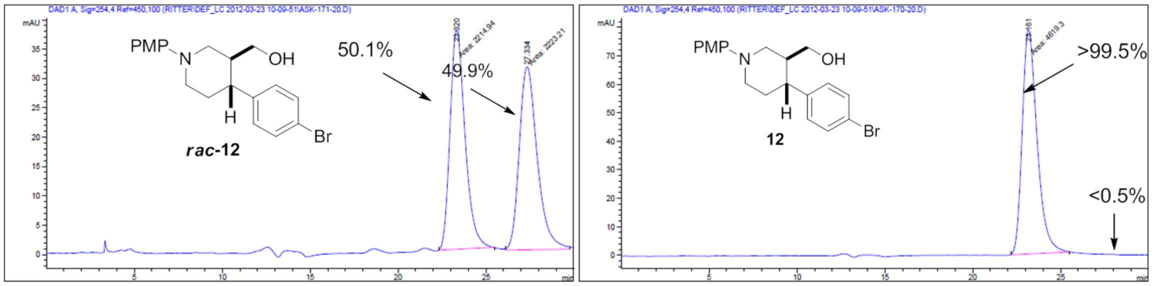

HPLC method: Chiralcel OD-H column with 10% isopropanol/hexanes eluent for racemic **12** and enantioenriched **12**. Percent of total integration listed for each peak.

(3*S*,4*R*)-*t*-Butyl 4-(4-bromophenyl)-3-(hydroxymethyl)piperidine-1-carboxylate (S10)

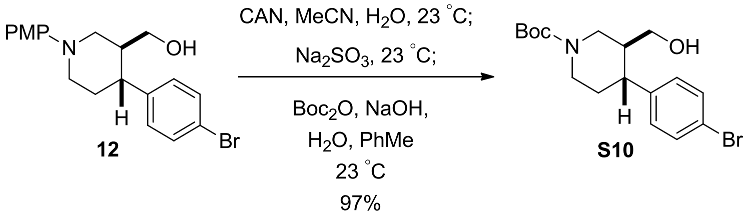

To ((3*S*,4*R*)-4-(4-bromophenyl)-1-(4-methoxyphenyl)piperidin-3-yl)methanol (**12**) (6.00 g, 16.0 mmol, 1.00 equiv) suspended in 3:1 solution MeCN/H_2O (320 mL) in a round-bottom flask open to air was added ceric ammonium nitrate (CAN) (35.0 g, 63.8 mmol, 4.00 equiv). The reaction mixture turned purple/red and then red/orange upon stirring for 30 minutes at 23 ºC. Sodium sulfite (5.02 g, 39.9 mmol, 2.50 equiv) was added and the reaction mixture turned yellow upon stirring for 15 minutes at 23 ºC. The suspension was concentrated *in vacuo* to approximately 50 mL. H_2O (100 mL) and PhMe (150 mL) were added followed by a 3.0 M aqueous solution of NaOH until the reaction mixture pH was greater than 10. Boc_2O (6.96 g, 7.33 mL, 31.9 mmol, 2.00 equiv) was added and the reaction mixture was stirred at 23 ºC for 3 hours. The reaction mixture was filtered through celite. The celite was washed with EtOAc (200 mL) and combined with the reaction mixture in a separatory funnel. The funnel was shaken and the organic phase collected. The aqueous phase was extracted from with EtOAc (2 × 200 mL). The combined organic phases were washed with brine (150 mL), dried with Na_2SO_4, and concentrated *in vacuo*. The residue was purified by chromatography on silica gel, eluting with a gradient of 25–50% EtOAc in hexanes (v/v) to afford 5.70 g of the title compound as a colorless solid (97% yield).

R*f* = 0.20 (hexanes/EtOAc 3:1 (v/v)). NMR Spectroscopy: ^{1}H NMR (500 MHz, $CDCl_3$, 23 ºC, δ): 7.47–7.44 (m, 2H), 7.08–7.05 (m, 2H), 4.38 (br s, 1H), 4.22 (br s, 1H), 3.97 (dd, *J* = 10.1, 2.7 Hz, 1H), 3.81 (dd, *J* = 10.1, 6.8 Hz, 1H), 2.78–2.71 (br m, 2H) 2.53 (ddd, *J* = 11.9, 11.9, 3.8 Hz, 1H),

2.08–2.02 (m, 1H), 1.80 (dd, J = 13.3, 2.7 Hz, 1H), 1.66 (ddd, J = 25.6, 12.6, 4.4 Hz, 1H), 1.49 (s, 9H). ^{13}C NMR (125 MHz, CDCl$_3$, 23 ºC, δ): 154.7, 141.5, 132.2, 129.2, 121.1, 80.2, 69.5, 44.3, 41.0, 37.3, 34.0, 28.6. Note: The ^{13}C NMR spectrum contains one less carbon signal than is expected. HRMS-FIA (m/z): calcd for $C_{17}H_{23}BrNO_3$ [M - X]$^-$, 368.0861; found, 368.0839.

(3*S*,4*R*)-*t*-Butyl 3-((benzo[*d*][1,3]dioxol-5-yloxy)methyl)-4-(4-bromophenyl)piperidine-1-carboxylate (13)

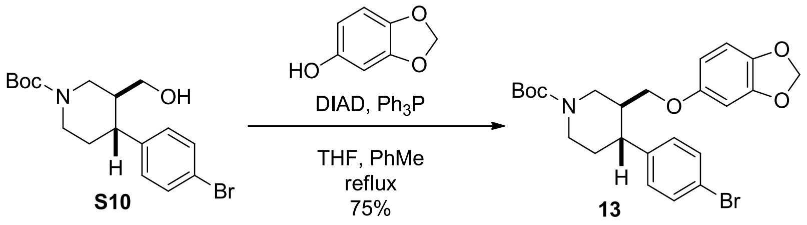

To (3*S*,4*R*)-*t*-Butyl 4-(4-bromophenyl)-3-(hydroxymethyl)piperidine-1-carboxylate (**S10**) (1.00 g, 2.70 mmol, 1.00 equiv), sesamol (0.746 g, 5.40 mmol, 2.00 equiv), triphenyl phosphine (0.921 g, 3.51 mmol, 1.30 equiv) in a 5:1 solution THF:PhMe (18 mL) in a flame-dried round-bottom flask fitted with a reflux condenser under nitrogen was added diisopropyl azodicarboxylate (DIAD) (0.710 g, 0.691 mL, 3.51 mmol, 1.30 equiv). The reaction mixture was heated at reflux for 18 hours, cooled to 23 ºC, and then poured into a 1.0 M aqueous solution of NaOH in a separatory funnel. The reaction mixture was extracted from with diethyl ether (3 × 50 mL). The combined organic phases were washed with brine (100 mL), dried with $MgSO_4$, and concentrated *in vacuo*. The residue was purified by chromatography on silica gel, eluting with a gradient of 10–20% EtOAc in hexanes (v/v) to afford 0.987 g of the title compound as a colorless solid (75% yield).

R*f* = 0.30 (hexanes/EtOAc 5:1 (v/v)). NMR Spectroscopy: ^{1}H NMR (600 MHz, CDCl$_3$, 23 ºC, δ): 7.42–7.40 (m, 2H), 7.07–7.05 (m, 2H), 6.63 (d, J = 8.4 Hz, 1H), 6.35 (d, J = 2.5 Hz, 1H), 6.13 (dd, J = 8.5, 2.5 Hz, 1H), 5.88 (s, 2H), 4.43 (br s, 1H), 4.24 (br s, 1H), 3.60 (dd, J = 9.5, 2.8 Hz, 1H), 3.45 (dd, J = 9.4, 6.5 Hz, 1H), 2.82–2.78 (br m, 2H), 2.66 (br dd, J = 10.3, 10.3 Hz, 1H), 2.04–1.98 (br m, 1H), 1.80–1.79 (br m, 1H), 1.70 (br ddd, J = 25.3, 12.6, 4.1 Hz, 1H), 1.49 (s, 9H). ^{13}C NMR (125 MHz, CDCl$_3$, 23 ºC, δ): 154.9, 154.4, 148.3, 142.6, 141.8, 132.0, 129.3, 120.6, 108.0, 105.7, 101.3, 98.1, 79.9, 68.9, 44.4, 41.8, 33.9, 28.6. Note: The ^{13}C NMR spectrum contains two less carbon signals than is expected. HRMS-FIA (m/z): calcd for $C_{24}H_{29}BrNO_5$ [M + H]$^+$, 490.1229; found, 490.1207.

(3*S*,4*R*)-*t*-Butyl 3-((benzo[*d*][1,3]dioxol-5-yloxy)methyl)-4-(4-(4,4,5,5-tetramethyl-1,3,2-dioxaborolan-2-yl)phenyl)piperidine-1-carboxylate (14)

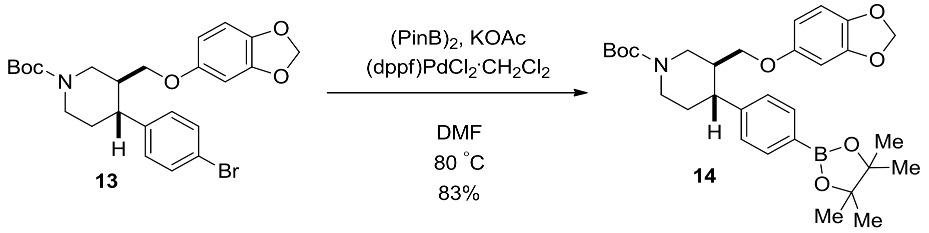

A flame-dried Schlenk tube under a N_2 atmosphere was charged with (3*S*,4*R*)-*t*-butyl 3-((benzo[*d*][1,3]dioxol-5-yloxy)methyl)-4-(4-bromophenyl)piperidine-1-carboxylate (**13**) (975 mg, 1.99 mmol, 1.00 equiv), bis(pinacolato)diboron (555 mg, 2.19 mmol, 1.10 equiv), potassium acetate (585 mg, 5.96 mmol, 3.00 equiv), and $PdCl_2(dppf)·CH_2Cl_2$ (49.0 mg, 60.0 µmol, 0.0300 equiv). DMF (30 mL) was added via syringe. The reaction mixture was degassed via 2 consecutive freeze/pump/thaw cycles. The Schlenk tube was then backfilled with N_2 and heated at 80 °C for 2.5 hours. The reaction mixture was cooled and poured into H_2O (100 mL). The reaction mixture was extracted from with diethyl ether (3 × 100 mL). The combined organic phases were washed with brine (100 mL), dried with $MgSO_4$, and concentrated *in vacuo*. The residue was purified by chromatography on silica gel, eluting with a gradient of 15–25% EtOAc in hexanes (v/v) to afford 882 mg of the title compound as a colorless solid (83% yield).

R*f* = 0.25 (hexanes/EtOAc 5:1 (v/v)). NMR Spectroscopy: ^{1}H NMR (500 MHz, $CDCl_3$, 23 °C, δ): 7.77–7.75 (m, 2H), 7.22–7.21 (m, 2H), 6.63 (d, *J* = 8.2 Hz, 1H), 6.36 (d, *J* = 2.7 Hz, 1H), 6.14 (dd, *J* = 8.5, 2.6 Hz, 1H), 5.89 (s, 2H), 4.48 (br s, 1H), 4.27 (br s, 1H), 3.62 (dd, *J* = 9.2, 2.8 Hz, 1H), 3.45 (dd, *J* = 9.6, 6.9 Hz, 1H), 2.82–2.77 (br m, 2H), 2.69 (br dd, *J* = 9.4, 9.4 Hz, 1H), 2.15–2.09 (br m, 1H), 1.84–1.72 (br m, 2H), 1.52 (s, 9H), 1.35 (s, 12H). ^{13}C NMR (125 MHz, $CDCl_3$, 23 °C, δ): 154.9, 154.4, 148.2, 146.9, 141.7, 135.3, 127.0, 107.9, 105.7, 101.2, 98.2, 83.9, 79.7, 69.0, 45.2, 41.6, 33.8, 28.6, 25.0. Note: The ^{13}C NMR spectrum contains three less carbon signals than is expected. HRMS-FIA (m/z): calcd for $C_{30}H_{41}BNO_7$ $[M + H]^+$, 538.2976; found, 538.2991.

Palladium aryl complex 15

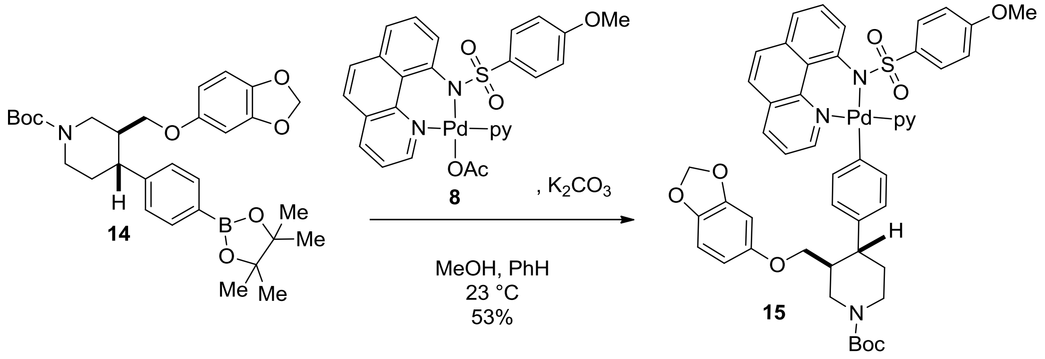

To (3*S*,4*R*)-*t*-butyl 3-((benzo[*d*][1,3]dioxol-5-yloxy)methyl)-4-(4-(4,4,5,5-tetramethyl-1,3,2-dioxaborolan-2-yl)phenyl)piperidine-1-carboxylate (**14**) (820 mg, 1.53 mmol, 1.00 equiv) in a round-bottom flask open to air in a 1:1 solution PhH:MeOH (30 mL) was added palladium acetate complex **8** (928 mg, 1.53 mmol, 1.00 equiv) and potassium carbonate (316 mg, 2.29 mmol, 1.50 equiv). The suspension was stirred at 23 ºC for 6 hours. The reaction mixture was filtered through a frit eluting with CH_2Cl_2 (30 mL). The solution was concentrated *in vacuo* and the residue was purified by chromatography on silica gel, eluting with a gradient of 33–80% EtOAc in hexanes to afford 795 mg of the title compound as a yellow solid (54% yield).

R*f* = 0.40 (hexanes/EtOAc 1:3 (v/v)). NMR Spectroscopy: ^{1}H NMR (600 MHz, CD_2Cl_2, 23 ºC, δ): 8.99 (ddd, *J* = 5.0, 3.4, 1.6 Hz, 2H), 8.32 (ddd, *J* = 16.7, 5.4, 1.7 Hz, 1H), 8.00 (ddd, *J* = 17.9, 4.0, 1.6 Hz, 1H), 7.79–7.76 (m, 2H), 7.65–7.60 (m, 3H), 7.44 (dd, *J* = 8.8, 4.1 Hz, 1H), 7.32 (ddd, *J* = 7.6, 5.1, 1.5 Hz, 2H), 7.12–7.09 (m, 2H), 6.94 (ddd, *J* = 15.5, 7.9, 5.3 Hz, 1H), 6.85 (dd, *J* = 9.1, 8.1 Hz, 2H), 6.65–6.61 (m, 3H), 6.27 (dd, *J* = 14.0. 2.4 Hz, 1H), 6.24–6.21 (m, 2H), 6.09 (ddd, *J* = 8.5, 2.3, 2.3 Hz, 1H), 5.91 (dd, *J* = 4.5, 1.3 Hz, 1H), 5.90 (dd, *J* = 2.0, 1.4 Hz, 1H), 4.38 (br s, 1H), 4.15 (br s, 1H), 3.55 (s, 3H), 3.47 (ddd, *J* = 9.4, 9.4, 3.0 Hz, 1H), 3.33 (ddd, *J* = 19.7, 9.7, 7.8 Hz, 1H), 2.73–2.63 (br m, 2H), 2.37–2.31 (m, 1H), 2.91–1.85 (br m, 1H), 1.69–1.56 (m, 2H), 1.45 (s, 9H). ^{13}C NMR (125 MHz, CD_2Cl_2, 23 ºC, δ): 160.5, 154.9, 154.8, 154.3, 153.4, 148.5, 145.0, 143.8, 141.9, 138.5, 138.1, 138.0, 136.9, 136.6, 135.1, 130.0. 127.9, 127.7, 127.3, 126.3, 126.3, 125.1, 124.5, 123.7, 121.3, 112.5, 108.1, 106.1, 101.1, 98.1, 97.9, 79.4, 69.7, 55.5, 44.7, 42.3, 42.1, 33.9, 28.5. Note: The ^{13}C NMR spectrum contains two less carbon signals than is expected. δ):. Anal: calcd for $C_{49}H_{48}N_4O_8PdS$: C, 61.34; H, 5.04; N, 5.84; found: C, 61.09; H, 4.84; N, 5.74.

5-Bromo-2-(cyclopropylmethoxy)benzaldehyde (S11)

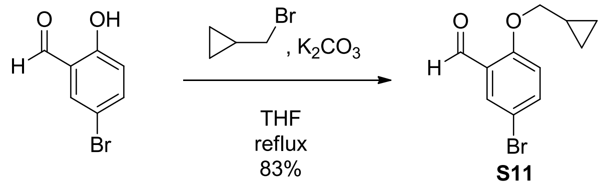

To 5-bromo-2-hydroxybenzaldehyde (1.00 g, 4.97 mmol, 1.00 equiv) and K_2CO_3 (3.44 g, 24.9 mmol, 5.00 equiv) in THF (10 mL) in an oven-dried round-bottom flask fitted with a reflux condenser under a N_2 atmosphere at 23 ºC was added (bromomethyl)cyclopropane (1.01 g, 0.724 mL, 7.46 mmol, 1.50 equiv). The reaction mixture was warmed in an oil heating bath at a temperature of 70 ºC and heated at reflux with vigorous stirring for 40 hours. The reaction mixture was cooled to 23 ºC and poured into H_2O (30 mL) in a separatory funnel. $CHCl_3$ (30 mL) was added, the funnel was shaken and the organic phase collected. The aqueous phase was then extracted with $CHCl_3$ (2 × 30 mL). The combined organic phases were washed with brine (30 mL), dried with Na_2SO_4, and concentrated in vacuo. The residue was purified by chromatography on silica gel, eluting with 2–7% EtOAc in hexanes (v/v) to afford 1.05 g of the

title compound as a colorless solid (83% yield).

R*f* = 0.30 (hexanes/EtOAc 19:1 (v/v)). NMR Spectroscopy: ^{1}H NMR (600 MHz, $CDCl_3$, 23 ºC, δ): 10.45 (s, 1H), 7.91 (d, *J* = 2.5 Hz, 1H), 7.58 (dd, *J* = 8.9, 2.6 Hz, 1H), 6.84 (d, *J* = 8.9 Hz, 1H), 3.91 (d, *J* = 7.2 Hz, 2H), 1.32–1.26 (m, 1H), 0.71–0.63 (m, 2H), 0.41–0.34 (m, 2H). ^{13}C NMR (125 MHz, $CDCl_3$, 23 ºC, δ): 188.7, 160.5, 138.3. 130.9, 126.5, 115.0, 113.5, 73.9, 10.1, 3.4. HRMS-FIA (m/z): calcd for $C_{11}H_{11}BrNaO_2$ $[M + Na]^+$, 276.9840; found, 276.9820.

(*E*)-Ethyl 3-(5-bromo-2-(cyclopropylmethoxy)phenyl)acrylate (S12)

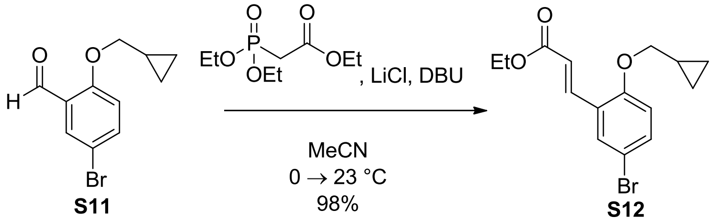

To 5-bromo-2-(cyclopropylmethoxy)benzaldehyde (**S11**) (3.10 g, 12.2 mmol, 1.00 equiv) and LiCl (0.541 g, 12.8 mmol, 1.05 equiv) in MeCN (45 mL) in a round-bottom flask under a N_2 atmosphere at 0 ºC was added triethyl phosphonoacetate (3.00 g, 2.68 mL, 13.4 mmol, 1.10 equiv) and 1,8-diazabicycloundec-7-ene (DBU) (2.04 g, 2.02 mL, 13.4 mmol, 1.10 equiv). Upon the addition of DBU, the reaction mixture turned yellow. The reaction mixture was warmed to 23 ºC and stirred for 15 hours. The reaction mixture was poured into H_2O (75 mL) in a separatory funnel. $CHCl_3$ (75 mL) was added and the funnel was shaken and the organic phase collected. The aqueous phase was extracted from with $CHCl_3$ (2 × 50 mL). All organic phases were combined and washed with brine (50 mL), dried with Na_2SO_4, and concentrated in vacuo. The residue was purified by chromatography on silica gel, eluting with 5–10% EtOAc in hexanes (v/v) to afford 3.89 g of the title compound as a colorless solid (98% yield).

R*f* = 0.25 (hexanes/EtOAc 19:1 (v/v)). NMR Spectroscopy: ^{1}H NMR (500 MHz, $CDCl_3$, 23 ºC, δ): 7.93 (d, *J* = 16.1 Hz, 1H), 7.60 (d, *J* = 2.4 Hz, 1H), 7.37 (dd, *J* = 8.8, 2.5 Hz, 1H), 6.74 (d, 8.8 Hz, 1H), 6.53 (d, *J* = 16.1 Hz, 1H), 4.26 (q, *J* = 6.8 Hz, 2H), 3.84 (d, *J* = 6.8 Hz, 2H), 1.34–1.25 (m, 4H), 0.70–0.61 (m, 2H), 0.40–0.31 (m, 2H). ^{13}C NMR (125 MHz, $CDCl_3$, 23 ºC, δ): 167.3, 156.9, 138.7, 133.7, 131.3, 125.9, 120.0, 114.4, 113.0, 73.9, 60.6, 14.4, 10.2, 3.4. HRMS-FIA (m/z): calcd for $C_{15}H_{18}BrO_3$ $[M + H]^+$, 325.0439; found, 325.0428.

(*E*)-3-(5-Bromo-2-(cyclopropylmethoxy)phenyl)prop-2-en-1-ol (16)

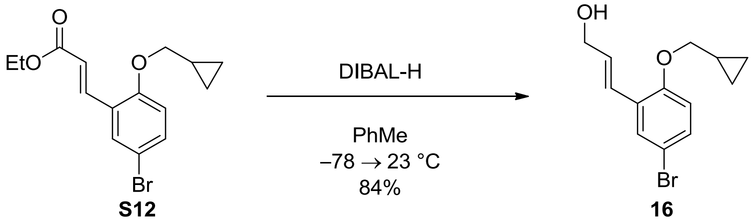

To (*E*)-ethyl 3-(5-bromo-2-(cyclopropylmethoxy)phenyl)acrylate (**S12**) (3.78 g, 11.6 mmol, 1.00 equiv) in PhMe (30 mL) in a flame-dried round-bottom flask under a N_2 atmosphere at –78 ºC was added a 1.0 M solution of diisobutylaluminum hydride (DIBAL-H) in PhMe (26 mL, 26 mmol, 2.2 equiv) in 6 portions dropwise every 10 minutes for 1 hour. The reaction was warmed to 0 ºC over 2 hours and then warmed to 23 ºC and stirred at this temperature for 1 hour. The reaction mixture was poured onto a concentrated aqueous Rochelle's salt (potassium sodium tartrate) solution (400 mL). EtOAc (400 mL) was added and the mixture was stirred for 3 hour until two liquid phases separated cleanly. The phases were partitioned and the aqueous phase was extracted from with EtOAc (300 mL). The organic phases were combined and washed with brine (200 mL), dried with Na_2SO_4, and concentrated in vacuo. The residue was purified by chromatography on silica gel, eluting with a gradient of 10–25% EtOAc in hexanes (v/v) to afford 2.77 g of the title compound as a colorless solid (84% yield).

R_f = 0.15 (hexanes/EtOAc 6:1 (v/v)). NMR Spectroscopy: ^{1}H NMR (500 MHz, $CDCl_3$, 23 ºC, δ): 7.53 (d, *J* = 2.4 Hz, 1H), 7.26 (dd, *J* = 8.8, 2.4 Hz, 1H), 6.88 (d, *J* = 16.1 Hz, 1H), 6.69 (d, *J* = 8.8 Hz, 1H), 6.39 (dt, *J* = 16.1, 5.9 Hz, 1H), 4.33 (br dd, *J* = 4.6, 4.6 Hz, 2H), 3.79 (d, *J* = 6.8 Hz, 2H), 1.71 (br t, *J* = 5.1 Hz, 1H), 1.31–1.23 (m, 1H), 0.68–0.58 (m, 2H), 0.38–0.30 (m, 2H). ^{13}C NMR (125 MHz, $CDCl_3$, 23 ºC, δ): 155.4, 131.2, 130.5, 129.7, 128.2, 125.0, 114.2, 113.2, 73.7, 64.1, 10.3, 3.4. HRMS-FIA (m/z): calcd for $C_{13}H_{15}BrNaO_2$ $[M + Na]^+$, 305.0153; found, 305.0123.

((1*S*,2*S*)-2-(5-Bromo-2-(cyclopropylmethoxy)phenyl)cyclopropyl)methanol (17)

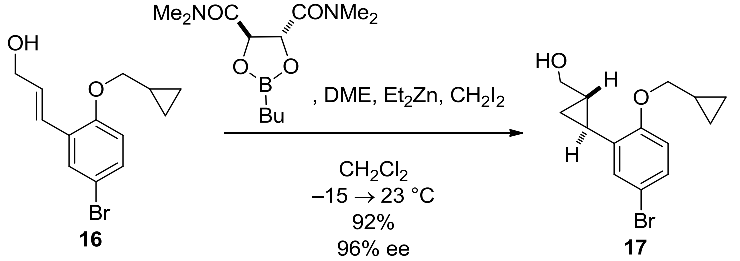

Following a published procedure for asymmetric allylic cyclopropanation:[13] To dimethoxyethane (DME) (1.39 g, 1.60 mL, 15.4 mmol, 1.90 equiv) in CH_2Cl_2 (50 mL) in a flame-dried round-bottom flask under a N_2 atmosphere cooled in an ethyleneglycol/CO_2 bath at –15 ºC was added diethylzinc (2.01 g, 1.67 mL, 16.3 mmol, 2.00 equiv), while maintaining the bath temperature between –15 and –10 ºC. CH_2I_2 (8.70 g, 2.62 mL, 32.5 mmol, 4.00 equiv) was added dropwise over 20 minutes at –15 ºC. The reaction mixture was stirred at –15 ºC for 10 minutes. A solution of (4*R*,5*R*)-2-butyl-*N*,*N*,*N'*,*N'*-tetramethyl-1,3,2-dioxaborolane-4,5-dicarboxamide (2.63 g, 2.46 mL, 9.75 mmol, 1.20 equiv) in CH_2Cl_2 (10 mL) from a separate flame-dried round-bottom flask under a N_2 atmosphere was added over 5 minutes via syringe. A solution of (*E*)-3-(5-bromo-2-(cyclopropylmethoxy)phenyl)prop-2-en-1-ol (**16**) (2.30 g, 8.12 mmol, 1.00 equiv) in CH_2Cl_2 (10 mL) from a separate flame-dried round-bottom flask under a N_2 atmosphere was added over 5 minutes via syringe. The reaction mixture was allowed to warm to 23 ºC and stirred for 20 hours.

Saturated aqueous NH_4Cl solution (10 mL) and 1M HCl (50 mL) were added to the reaction mixture. The reaction mixture was transferred to a separatory funnel. Diethyl ether (200 mL) was added and the separatory funnel was shaken and the organic phase was separated. The aqueous phase was extracted from with diethyl ether (200 mL) and then again with diethyl ether (100 mL). The combined organic phases were transferred to an Erlenmeyer flask. 2 M NaOH solution (60 mL) and 30% H_2O_2 solution (15 mL) were added. The reaction mixture was stirred vigorously for 5 minutes. The reaction mixture was transferred into a separatory funnel and partitioned. The organic phase was washed with 1.0 M aqueous HCl (75 mL), saturated aqueous Na_2CO_3 solution (75 mL), saturated aqueous $NaHCO_3$ solution (75 mL) and brine (75 mL). The organic phase was dried with $MgSO_4$, and concentrated in vacuo. The residue was purified by chromatography on silica gel, eluting with a gradient of 10–30% EtOAc in hexanes (v/v) to afford 2.21 g of the title compound as a colorless oil (92% yield and 96% *ee* as determined on a Chiracel ODH column with 5% isopropanol/hexanes eluent (see Figure S4). Racemic **17** was synthesized using the above procedures omitting the addition of (4*R*,5*R*)-2-butyl-*N*,*N*,*N'*,*N'*-tetramethyl-1,3,2-dioxaborolane-4,5-dicarboxamide. Absolute stereochemistry was assigned by analogy.[13]

R_f = 0.20 (hexanes/EtOAc 6:1 (v/v)). NMR Spectroscopy: ^{1}H NMR (500 MHz, $CDCl_3$, 23 ºC, δ): 7.24 (dd, J = 8.8, 2.4 Hz, 1H), 7.09 (d, J = 2.4 Hz, 1H), 6.65 (d, J = 8.8 Hz, 1H), 3.95 (ddd, J = 10.7, 8.8, 4.9 Hz, 1H), 3.82 (d, J = 7.3 Hz, 2H), 3.19 (ddd, J = 10.7, 10.7, 2.0, 1H), 2.40 (dd, J = 8.5, 2.0 Hz, 1H), 1.86 (ddd, J = 8.5, 5.0, 5.0 Hz, 1H) 1.34–1.27 (m, 1H), 1.20–1.15 (m, 1H), 1.14–1.09 (m, 1H), 0.86 (ddd, J = 9.0, 5.0, 5.0 Hz, 1H), 0.71–0.65 (m, 2H), 0.40–0.34 (m, 2H). ^{13}C NMR (125 MHz, $CDCl_3$, 23 ºC, δ): 157.2, 132.4, 130.2, 129.9, 112.8, 112.6, 73.6, 67.3, 24.5, 17.2, 10.2, 9.9, 3.7, 3.2. HRMS-FIA (m/z): calcd for $C_{14}H_{17}BrNaO_2$ $[M + Na]^+$, 319.0310; found, 319.0327.

Figure S4: Enantiodiscriminating HPLC trace of 17

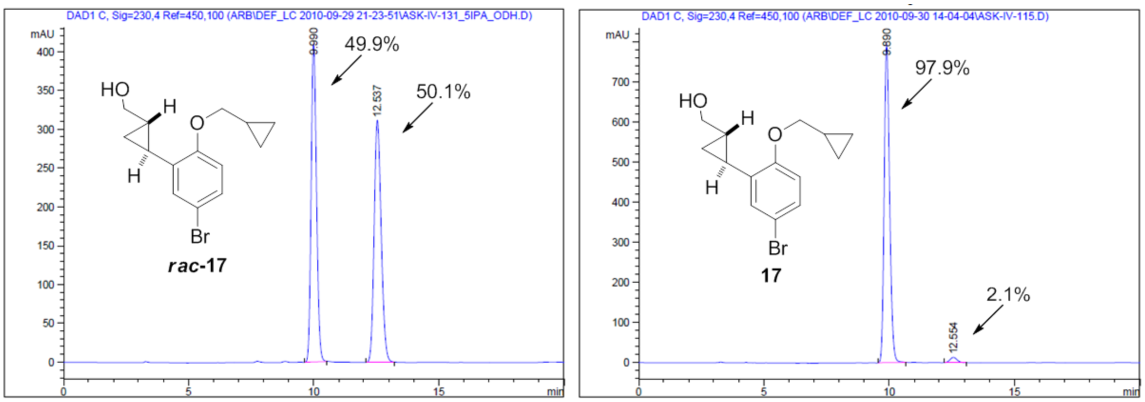

HPLC method: Chiracel ODH column with 5% isopropanol/hexanes eluent for racemic **17** and enantioenriched **17**. Percent of total integration listed for each peak.

2-((1*S*,2*S*)-2-(Azidomethyl)cyclopropyl)-4-bromo-1-(cyclopropylmethoxy)benzene (S13)

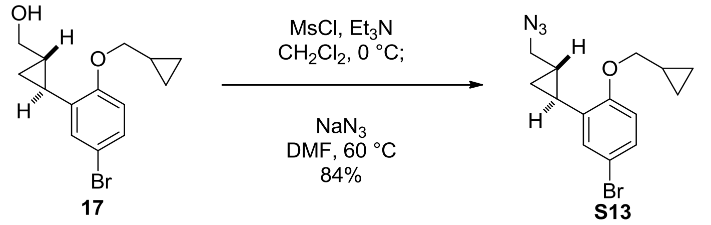

To ((1*S*,2*S*)-2-(5-bromo-2-(cyclopropylmethoxy)phenyl)cyclopropyl)methanol (**17**) (2.15 g, 7.23 mmol, 1.00 equiv) in CH_2Cl_2 (30 mL) in an oven-dried round-bottom flask under a N_2 atmosphere at 0 ºC was added Et_3N (2.20 g, 3.03 mL, 21.7 mmol, 3.00 equiv) and MsCl (1.66 g, 1.13 mL, 14.5 mmol, 2.00 equiv). The reaction mixture was stirred at 0 ºC for 2 hours. The reaction mixture turned yellow and a precipitate formed. The reaction mixture was poured into a separatory funnel with saturated NH_4Cl solution (40 mL). The funnel was shaken and the organic phase collected. The aqueous phase was extracted from with diethyl ether (3 × 75 mL). The organic phases were combined and washed with saturated $NaHCO_3$ (100 mL) and brine (100 mL), dried with $MgSO_4$, and concentrated in vacuo. The residue was dissolved in DMF (30 mL) and NaN_3 (1.88 g, 28.9 mmol, 4.00 equiv) was added. The reaction mixture was heated at 60 ºC for 1 hour. The reaction mixture was cooled and poured into 60 mL of water. The reaction mixture was extracted from with diethyl ether (3 × 75 mL). The combined organic phases were washed with brine (100 mL), dried with $MgSO_4$, and concentrated in vacuo. The residue was purified by chromatography on silica gel, eluting with a gradient of 5–10% EtOAc in hexanes (v/v) to afford 1.95 g of the title compound as a colorless oil (84% yield).

R_f = 0.60 (hexanes/EtOAc 19:1 (v/v)). NMR Spectroscopy: ^{1}H NMR (500 MHz, $CDCl_3$, 23 ºC, δ): 7.21 (dd, *J* = 8.7, 2.3 Hz, 1H), 6.96 (d, *J* = 2.3 Hz, 1H), 6.66 (d, *J* = 8.7 Hz, 1H), 3.84-3.78 (m, 2H), 3.40 (dd, *J* = 12.8, 6.4, 1H), 3.24 (dd, *J* = 12.8, 7.1 Hz, 1H), 2.11 (ddd, *J* = 8.7, 5.0, 5.0 Hz, 1H), 1.38–1.32 (m, 1H), 1.31–1.25 (m, 1H), 1.08–1.04 (m, 1H), 0.98–0.94 (m, 1H), 0.68–0.58 (m, 2H), 0.40–0.31 (m, 2H). ^{13}C NMR (125 MHz, $CDCl_3$, 23 ºC, δ): 156.9, 132.8, 129.5, 128.8, 113.4, 112.9, 73.3, 55.3, 20.8, 16.2, 12.8, 10.4, 3.3, 3.2. HRMS-FIA (m/z): calcd for $C_{14}H_{16}BrN_3NaO$ [M + Na]$^+$, 344.0374; found, 344.0363.

***t*-Butyl (((1S,2S)-2-(5-bromo-2-(cyclopropylmethoxy)phenyl)cyclopropyl)methyl) carbamate (18)**

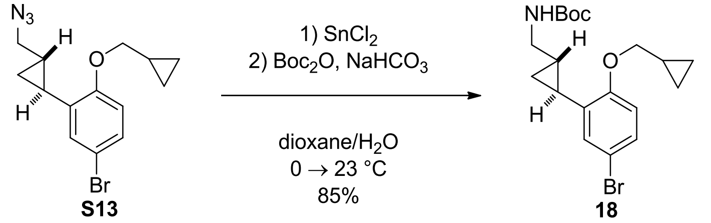

To 2-((1*S*,2*S*)-2-(azidomethyl)cyclopropyl)-4-bromo-1-(cyclopropylmethoxy)benzene (**S13**) (1.90 g, 5.90 mmol, 1.00 equiv) in a round-bottom flask open to air in a 2:1 solution of dioxane:H_2O (45 mL) cooled to 0 ºC was added tin(II) chloride (5.59 g, 29.5 mmol, 5.00 equiv). The reaction mixture was allowed to warm to 23 ºC and stirred for 15 hours. Saturated aqueous $NaHCO_3$ solution (50 mL) was carefully added. The addition was accompanied by foaming. H_2O (15 mL) was added followed by Boc_2O (3.86 g, 4.11 mL, 17.7 mmol, 3.00 equiv). The reaction mixture was stirred for 3 hours and then transferred to a separatory funnel. The reaction mixture was extracted from with EtOAc (3 × 75 mL). The combined organic phases were washed with brine (75 mL), dried with Na_2SO_4, and concentrated in vacuo. The residue was purified by chromatography on silica gel, eluting with a gradient of 5–20% EtOAc in hexanes (v/v) to afford 1.96 g of the title compound as a colorless solid (85% yield). The enantioenriched product could be recrystallized by suspending the solid in hexanes (10 mL), heating the suspension to reflux to dissolve the solid, cooling the solution, and collecting the solid by filtration, affording the title compound in >99% *ee* as determined on a Chiracel ODH column with 5% isopropanol/hexanes eluent (see Figure S5).

R_f = 0.25 (hexanes/EtOAc 19:1 (v/v)). NMR Spectroscopy: ^{1}H NMR (500 MHz, $CDCl_3$, 23 ºC, δ): 7.23 (dd, *J* = 8.3, 2.4 Hz, 1H), 7.06 (br d, *J* = 2.0 Hz, 1H), 6.66 (d, *J* = 8.8 Hz, 1H), 5.27 (br, 1H), 3.97 (dd, *J* = 9.5, 7.1 Hz, 1H), 3.72–3.66 (m, 2H), 2.66 (br dd, *J* = 10.0, 10.0, 1H), 1.83 (ddd, *J* = 6.6, 6.6, 4.9 Hz, 1H), 1.43 (br, 10H), 1.06–0.99 (br m, 2H), 0.83–0.80 (br m, 1H), 0.67 (br m, 2H), 0.38 (br m, 2H). ^{13}C NMR (125 MHz, $CDCl_3$, 23 ºC, δ): 157.2, 155.9, 132.6, 130.3, 129.7, 112.8, 112.7, 79.1, 73.5, 45.7, 28.6, 21.1, 17.4, 10.6, 10.3, 3.5. HRMS-FIA (m/z): calcd for $C_{19}H_{26}BrNNaO_3$ $[M + Na]^+$, 418.0988; found, 418.0994.

Figure S5. Enantiodiscriminating HPLC trace of 18

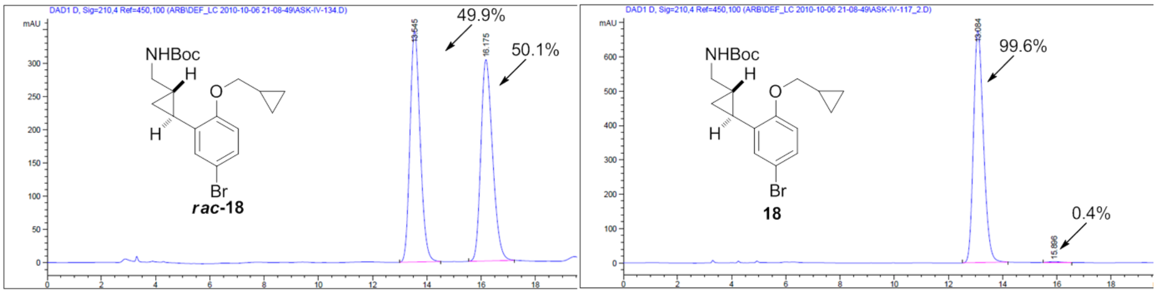

HPLC method: Chiracel ODH column with 5% isopropanol/hexanes eluent for racemic **18** and enantioenriched **18**. Percent of total integration listed for each peak.

t-Butyl (((1S,2S)-2-(2-(cyclopropylmethoxy)-5-(4,4,5,5-tetramethyl-1,3,2-dioxaborolan-2-yl)phenyl)cyclopropyl)methyl)carbamate (19)

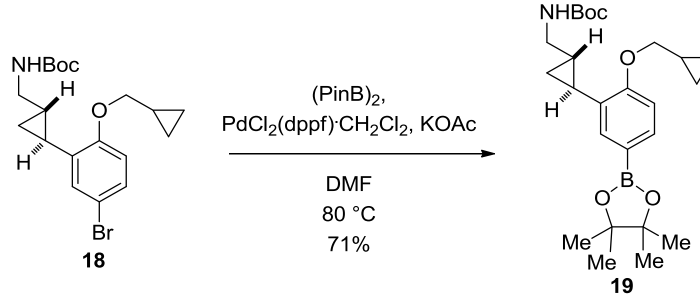

A flame-dried Schlenk tube under a N_2 atmosphere was charged with *t*-butyl (((1S,2S)-2-(5-bromo-2-(cyclopropylmethoxy)phenyl)cyclopropyl)methyl) carbamate (**18**) (250. mg, 0.631 mmol, 1.00 equiv), bis(pinacolato)diboron (176 mg, 0.694 mmol, 1.10 equiv), potassium acetate (186 mg, 1.89 mmol, 3.00 equiv), and $PdCl_2$(dppf)·CH_2Cl_2 (15.5 mg, 18.9 umol, 0.0300 equiv). DMF (25 mL) was added via syringe. The reaction mixture was degassed via 2 consecutive freeze/pump/thaw cycles. The Schlenk tube was then backfilled with N_2 and heated at 80 ºC for 16 hours. The reaction mixture was cooled and poured into H_2O (25 mL). The reaction mixture was extracted from with diethyl ether (4 × 30 mL). The combined organic phases were washed with brine (50 mL), dried with $MgSO_4$, and concentrated in vacuo. The residue was purified by chromatography on silica gel, eluting with a gradient of 5–20% EtOAc in hexanes (v/v) to afford 198 mg of the title compound as a colorless solid (71% yield).

R_f = 0.40 (hexanes/EtOAc 4:1 (v/v)). NMR Spectroscopy: ^{1}H NMR (500 MHz, $CDCl_3$, 23 ºC, δ): 7.63 (d, *J* = 8.2 Hz, 1H), 7.42 (s, 1H), 6.80 (d, *J* = 8.2 Hz, 1H), 5.36 (br, 1H), 4.04 (dd, *J* = 9.6, 6.9 Hz, 1H), 3.77 (dd, *J* = 9.6, 7.8 Hz, 1H), 3.70 (br m, 1H), 2.63 (br dd, *J* = 10.8, 10.8 Hz, 1H), 1.82 (ddd, *J* = 6.6, 6.6, 5.5 Hz, 1H), 1.43 (br , 10H), 1.32 (br, 12H), 1.18–1.14 (m, 1H), 1.04–0.98 (br m, 1H), 0.80–0.76 (m, 1H), 0.70–0.64 (br m, 2H), 0.41–0.36 (br m, 2H). ^{13}C NMR (125 MHz, $CDCl_3$, 23 ºC, δ): 160.8, 155.9, 134.7, 134.1, 129.3, 120.1 (br), 110.3, 83.7, 79.0, 73.1, 46.0, 28.6, 25.0, 24.9, 20.5, 17.6, 10.3, 3.5, 3.5. HRMS-FIA (m/z): calcd for $C_{25}H_{38}BNNaO_5$ $[M + Na]^+$, 466.2741; found, 466.2750.

Palladium aryl complex 20

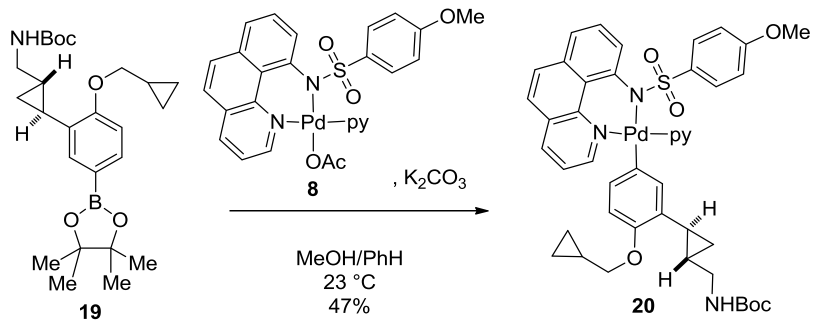

To *t*-butyl (((1S,2S)-2-(2-(cyclopropylmethoxy)-5-(4,4,5,5-tetramethyl-1,3,2-dioxaborolan-2-yl)phenyl)cyclopropyl)methyl)carbamate (**19**) (195 mg, 0.440 mmol, 1.00 equiv) in a round-bottom flask open to air in a 1:1 solution PhH:MeOH (8 mL) was added palladium acetate complex **8** (267 mg, 0.440 mmol, 1.00 equiv) and potassium carbonate (91.0 mg, 0.660 mmol, 1.50 equiv). The suspension was stirred at 23 ºC for 4 hours. The reaction was filtered through microfiber filter paper using CH_2Cl_2 (10 mL). The solution was concentrated in vacuo and the residue was purified by chromatography on silica gel, eluting with a gradient of 25–75% EtOAc in hexanes to afford 180 mg of the title compound as a yellow solid (47% yield).

R_f = 0.30 (hexanes/EtOAc 1:2 (v/v)). NMR Spectroscopy: ^{1}H NMR (500 MHz, CD_2Cl_2, 23 ºC, δ): 8.99 (dd, *J* = 5.1, 1.7 Hz, 2H), 8.33 (ddd, *J* = 9.3, 5.4, 1.5 Hz, 1H), 8.06–8.03 (m, 1H), 7.82–7.77 (m, 2H), 7.66–7.62 (m, 2H), 7.58–7.56 (m, 1H), 7.45 (dd, *J* = 8.5, 5.6 Hz, 1H), 7.35 (dd, *J* = 6.3, 6.3 Hz, 2H), 7.10–7.05 (m, 3H), 6.58 (dd, *J* = 8.3, 1.5, 0.5H), 6.53 (dd, *J* = 8.3, 1.5, 0.5H), 6.37–6.34 (m, 2H), 6.28 (s, 1H), 6.23–6.20 (m, 2H), 5.27 (br, 1H), 3.82 (br dd, *J* = 16.6, 9.8 Hz, 1H), 3.59–3.50 (m, 5H), 2.57–2.52 (br m, 0.5H), 2.58–2.46 (br m, 0.5H), 1.64 (br m, 1H), 1.42–1.29 (m, 10H), 0.84–0.76 (br m, 1H), 0.64–0.53 (m, 4H) 0.31–0.28 (m, 2H). Note: fractional hydrogen integration and broad peaks are possibly due to slow rotation about bonds as seen for similar complexes (*18*). ^{13}C NMR (125 MHz CD_2Cl_2, 23 ºC, δ): 160.6, 155.9, 155.4, 155.4, 154.5, 154.3, 153.6, 153.6, 145.1, 145.1, 144.8, 144.4, 143.9, 138.1, 138.0, 136.8, 136.7, 136.6, 132.7, 132.6, 132.5, 130.0, 129.8, 128.9, 128.6, 127.9, 127.7, 127.6, 127.5, 125.9, 125.1, 124.5, 123.7, 121.5, 121.4, 112.5, 110.5, 110.4, 78.8, 73.2, 55.5, 46.0, 28.5, 21.3, 17.6, 17.5, 10.7, 10.1, 3.4. Note: There are more ^{13}C peaks than could be expected, possibly due to slow rotation about bonds as seen for similar complexes (*18*). Anal: calcd for $C_{44}H_{46}N_4O_6PdS$: C, 61.07; H, 5.36; N, 6.47; found: C, 61.02; H, 5.22; N, 6.20.

Fluorination of aryl palladium complexes

(3*S*,4*R*)-*t*-Butyl 3-((benzo[*d*][1,3]dioxol-5-yloxy)methyl)-4-(4-fluorophenyl)piperidine-1-carboxylate (21)

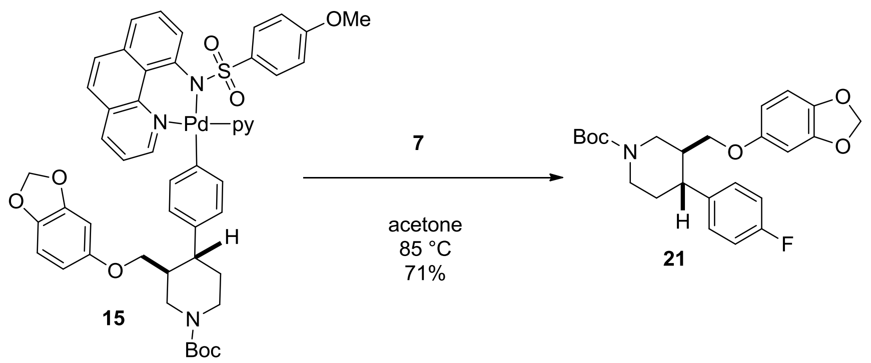

In a glove box under a N_2 atmosphere, palladium aryl complex **15** (135 mg, 141 μmol, 1.00 equiv) was dissolved in acetone (6 mL) and added to a soda lime glass bottle charged with Pd(IV)-F complex **7** (103 mg, 141 μmol, 1.00 equiv). The bottle was sealed, taken out of the glove box, and immersed in an oil bath heated at 85 ºC for 10 minutes. The reaction mixture was cooled and concentrated *in vacuo*. The residue was purified by chromatography on silica gel, eluting with a gradient of 10–20% EtOAc in hexanes to afford 42.7 mg of the title compound as a colorless solid (71% yield). The enantiomeric excess of the title compound remained >99% *ee* as determined on a Chiracel OD-H column with 3% isopropanol/hexanes eluent. The absolute configuration was identical to a sample of the title compound prepared from commercially available paroxetine (see Figure S6).

R_f = 0.25 (hexanes/EtOAc 6:1 (v/v)). NMR Spectroscopy: ^{1}H NMR (500 MHz, $CDCl_3$, 23 ºC, δ): 7.15–7.11 (m, 2H), 6.99–6.96 (m, 2H), 6.62 (d, *J* = 8.3 Hz, 1H), 6.34 (d, *J* = 2.4 Hz, 1H), 6.13 (dd, *J* = 8.8, 2.4 Hz, 1H), 5.88 (s, 2H), 4.43 (br s, 1H), 4.24 (br s, 1H), 3.60 (dd, *J* = 9.7, 2.9 Hz, 1H), 3.44 (dd, *J* = 9.5, 6.5 Hz, 1H), 2.82–2.77 (br m, 2H), 2.67 (br ddd, *J* = 11.5, 11.5, 2.7 Hz, 1H), 2.04–1.99 (br m, 1H), 1.81–1.78 (br m, 1H), 1.70 (br ddd, *J* = 24.9, 12.7, 4.4 Hz, 1H), 1.50 (s, 9H). ^{13}C NMR (125 MHz, $CDCl_3$, 23 ºC, δ): 161.7 (d, *J* = 244 Hz), 155.0, 154.4, 148.3, 141.8, 139.3 (d, *J* = 3 Hz), 128.9 (d, *J* = 7 Hz), 115.6 (d, *J* = 19 Hz), 108.0, 105.7, 101.2, 98.1, 79.8, 69.0, 44.2, 42.1, 34.1, 28.6. Note: The ^{13}C NMR spectrum contains two less carbon signals than is expected. ^{19}F NMR (375 MHz, $CDCl_3$, 23 ºC, δ): –116.0– –116.1 (m). HRMS-FIA (m/z): calcd for $C_{24}H_{29}FNO_5$ $[M + H]^+$, 430.2030; found, 430.1998.

Figure S6. Enantiodiscriminating HPLC trace of 21

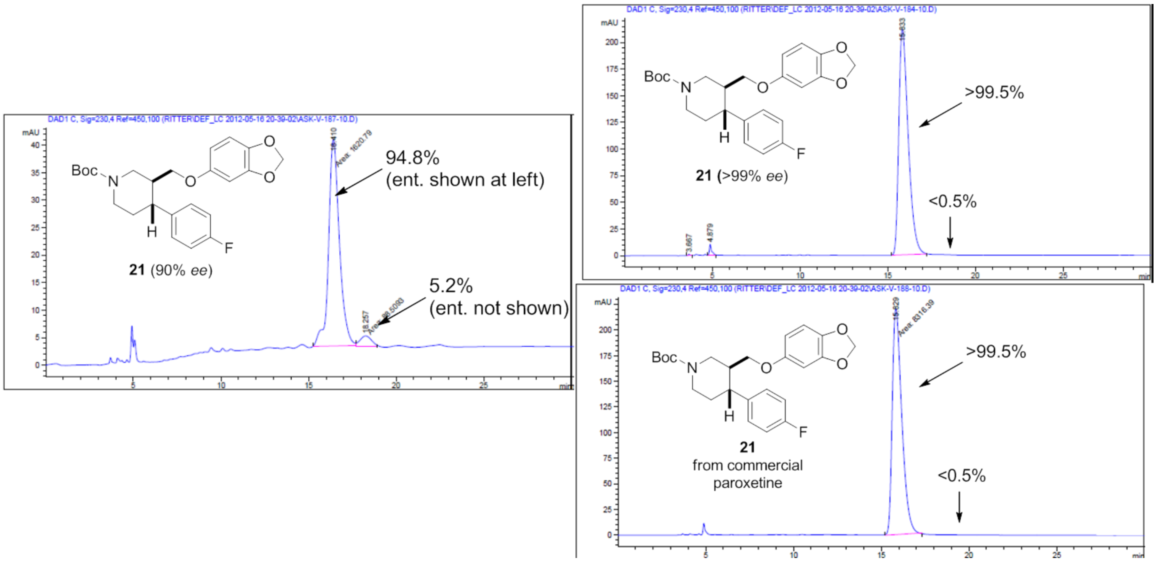

HPLC method: Chiralcel OD-H column with 3% isopropanol/hexanes eluent for 90% ee **21** and for >99% ee **21** and **21** synthesized from commercially available paroxetine. Percent of total integration listed for each peak.

***t-B*utyl(((1S,2S)-2-(2-(cyclopropylmethoxy)-5-fluorophenyl)cyclopropyl)methyl)carbamate (22)**

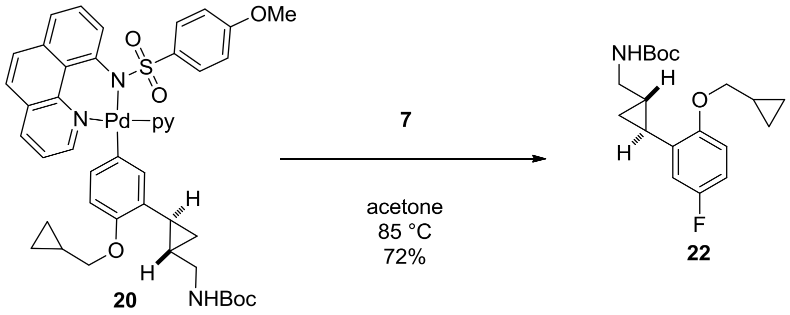

In a glove box under a N_2 atmosphere, palladium aryl complex **20** (25.0 mg, 29.4 umol, 1.20 equiv) was dissolved in acetone (3 mL) and added to a soda lime glass bottle charged with Pd(IV)-F complex **7** (18.0 mg, 24.5 umol, 1.00 equiv). The bottle was sealed, taken out of the glove box, and immersed in an oil bath heated at 85 ºC for 10 minutes. The reaction was cooled and concentrated in vacuo. The residue was purified by chromatography on silica gel, eluting with a gradient of 10–15% EtOAc in hexanes to afford 5.9 mg of the title compound as a colorless solid (72% yield).

R_f = 0.35 (hexanes/EtOAc 85:15 (v/v)). NMR Spectroscopy: ^{1}H NMR (500 MHz, $CDCl_3$, 23 ºC, δ): 6.80 (ddd, *J* = 8.8, 8.8, 2.9 Hz, 1H), 6.72 (dd, *J* = 9.1, 4.9, Hz, 1H), 6.66 (dd, *J* = 9.3, 3.0 Hz,

1H), 5.27 (br s, 1H), 3.96 (dd, J = 9.8, 6.8 Hz, 1H), 3.71 (dd, J = 9.8, 7.8 Hz, 1H), 3.66 (br s, 1H), 2.69 (br m, 1H), 1.88 (ddd, J = 6.8, 6.8, 4.9 Hz, 1H), 1.43–1.38 (m, 10H), 1.05–0.99 (m, 2H), 0.85–0.80 (m, 1H), 0.72–0.62 (m, 2H), 0.42–0.32 (m, 2H). ^{13}C NMR (125 MHz, $CDCl_3$, 23 ºC, δ): 157.2 (d, J = 238 Hz), 155.9, 154.1, 132.3 (d, J = 7 Hz), 114.1 (d, J = 24 Hz), 112.7 (d, J = 23 Hz), 112.2 (d, J = 9 Hz), 79.1, 73.9, 45.7, 28.6, 21.3, 17.5, 10.8, 10.4, 10.2, 3.5. ^{19}F NMR (375 MHz, $CDCl_3$, 23 ºC, δ): –123.8 (dd, J = 12.8, 8.0 Hz). HRMS-FIA (m/z): calcd for $C_{19}H_{26}FNNaO_3$ $[M + Na]^+$, 358.1794; found, 358.1808.

Deprotection of aryl fluorides

(3*S*,4*R*)-3-((Benzo[*d*][1,3]dioxol-5-yloxy)methyl)-4-(4-fluorophenyl)piperidine (1)

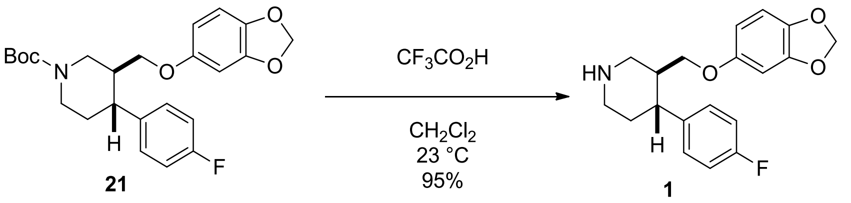

(3*S*,4*R*)-*t*-Butyl 3-((benzo[*d*][1,3]dioxol-5-yloxy)methyl)-4-(4-fluorophenyl)piperidine-1-carboxylate (**21**) (35.0 mg, 81.5 µmol, 1.00 equiv) was dissolved in a 7:1 solution CH_2Cl_2:CF_3CO_2H (2 mL). The solution was stirred at 23 ºC for 15 minutes. The solution was then dripped into saturated aqueous $NaHCO_3$ solution (10 mL). The aqueous layer was extracted with CH_2Cl_2 (3 × 10 mL). The combined organic phases were washed with brine (15 mL), dried with Na_2SO_4, and concentrated *in vacuo*. The residue was purified by chromatography on silica gel that had been washed with Et_3N, eluting with 0–10% MeOH in CH_2Cl_2 (v/v) to afford 25.6 mg of the title compound as a colorless oil (95% yield).

R_f = 0.35 (CH_2Cl_2/MeOH 19:1 (v/v) (Et_3N washed silica)). NMR Spectroscopy: ^1H NMR (600 MHz, $CDCl_3$, 23 ºC, δ): 7.18–7.16 (m, 2H), 6.98–6.96 (m, 2H), 6.61 (d, J = 8.5 Hz, 1H), 6.33 (d, J = 2.3 Hz, 1H), 6.13 (dd, J = 8.5, 2.5 Hz, 1H), 5.86 (s, 2H), 4.65 (br s, 1H), 3.56 (dd, J = 9.4, 2.6 Hz, 1H), 3.56 (dd, J = 9.4, 2.6 Hz, 1H), 3.48 (dd, J = 12.4, 3.6 Hz, 1H), 3.44 (dd, J = 9.2, 6.7 Hz, 1H), 3.28 (d, J = 12.2 Hz, 1H), 2.83–2.76 (m, 2H), 2.65 (ddd, J = 11.0, 11.0, 5.7 Hz, 1H), 2.22–2.17 (m, 1H), 1.91–1.84 (m, 2H). ^{13}C NMR (125 MHz, $CDCl_3$, 23 ºC, δ): 161.7 (d, J = 244 Hz), 154.3, 148.3, 141.8, 139.4 (d, J = 3 Hz), 128.9 (d, J = 8 Hz), 115.6 (d, J = 21 Hz), 107.9, 105.6, 101.2, 98.0, 69.1, 49.6, 46.5, 44.0, 42.2, 34.2. ^{19}F NMR (375 MHz, $CDCl_3$, 23 ºC, δ): -116.0 (tt, J = 8.6, 5.5 Hz). HRMS-FIA (m/z): calcd for $C_{19}H_{21}FNO_3$ $[M + H]^+$, 330.1505; found, 330.1497.

((1S,2S)-2-(2-(Cyclopropylmethoxy)-5-fluorophenyl)cyclopropyl)methanaminium 2,2,2-trifluoroacetate (2)

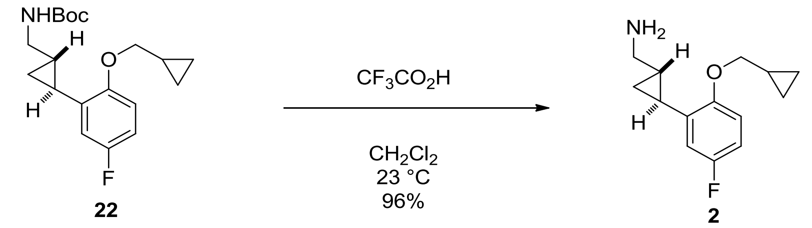

t-Butyl(((1S,2S)-2-(2-(cyclopropylmethoxy)-5-fluorophenyl)cyclopropyl)methyl)carbamate (**22**) (65.0 mg, 0.194 mmol, 1.00 equiv) was dissolved in a 1:1 solution CH_2Cl_2:CF_3CO_2H (2 mL). The solution was stirred at 23 ºC for 10 minutes. The solution was then dripped into saturated aqueous $NaHCO_3$ solution (20 mL). The aqueous layer was extracted with EtOAc (3 × 10 mL). The combined organic phases were washed with brine (15 mL), dried with Na_2SO_4, and concentrated *in vacuo*. The residue was purified by chromatography on silica gel that had been washed with Et_3N, eluting with 0–10% MeOH in CH_2Cl_2 (v/v) to afford the amine base. The title compound was isolated by adding a 10% CF_3CO_2H in CH_2Cl_2 solution (1 mL) to the amine base. The solution was concentrated *in vacuo* and heated *in vacuo* at 50 ºC to provide 65.5 mg of the title compound as its CF_3CO_2H salt (96% yield).

NMR Spectroscopy: ^{1}H NMR (500 MHz, D_2O, 23 ºC, δ): 6.99 (dd, *J* = 9.0, 5.0 Hz, 1H), 6.95 (ddd, *J* = 9.0, 9.0, 3.0 Hz, 1H), 6.78 (dd, *J* = 10.0, 3.0 Hz, 1H), 3.95 (dd, *J* = 10.0, 7.0, 1H), 3.87 (dd, *J* = 10.0, 2.5 Hz, 1H), 3.16 (dd, *J* = 12.0, 7.0 Hz, 1H), 3.02 (dd, *J* = 13.0, 8.0 Hz, 1H), 2.16 (ddd, *J* = 7.0, 7.0, 5.0 Hz, 1H), 1.38–1.27 (m, 2H), 1.16 (ddd, *J* = 5.5, 5.3, 5.3 Hz, 1H), 1.07 (ddd, *J* = 7.3, 7.3, 5.5 Hz, 1H), 0.65–0.62 (m, 2H), 0.37–0.34 (m, 2H). ^{19}F NMR (375 MHz, D_2O, 23 ºC, δ): -76.1 (s, 3F), -123.9 (ddd, *J* = 7.2, 7.2, 6.0, 1F). ^{13}C NMR (125 MHz, D_2O, 23 ºC, δ): 157.7 (d, *J* = 237 Hz), 153.4 (d, *J* = 2 Hz), 132.4 (d, *J* = 7 Hz), 115.4 (d, *J* = 9 Hz), 113.4 (d, *J* = 23 Hz), 113.1 (d, *J* = 24 Hz), 75.8, 43.9, 18.7, 16.7, 12.6, 9.9, 2.9, 2.6. HRMS-FIA (m/z): $[C_{14}H_{19}FNO + Na]^+$ calcd for $C_{14}H_{19}FNNaO$, 258.1270; found, 258.1274.

Radiochemistry

General methods

No-carrier-added [^{18}F]fluoride was produced from water 97% enriched in ^{18}O (Sigma-Aldrich®) by the nuclear reaction ^{18}O(p,n)^{18}F using a Siemens Eclipse HP cyclotron and a silver-bodied target at MGH Athinoula A. Martinos Center for Biomedical Imaging. The produced [^{18}F]fluoride in water was transferred from the cyclotron target by helium push. Liquid chromatographic analysis (LC) was performed with Agilent 1100 series HPLCs connected to a Carol and Ramsey Associates Model 105-S radioactivity detector. An Agilent Eclipse XDB-C18, 5 µm, 4.6 x 150 mm HPLC column was used for analytical analysis and a Macherey-Nagel VP 250/10 Nucleosil 100-5 C18 Nautilus column was used for preparative HPLC. In the analysis of the ^{18}F-labeled compounds, isotopically unmodified reference substances were used for identification. Radioactivity was measured in a Capintec, Inc. CRC-25PET ion chamber. *Solvents and reagents for radiochemical experiments:* Acetone (HPLC grade) was distilled over B_2O_3 and subsequently redistilled before use. 2-Butanone was distilled over B_2O_3. Acetonitrile was distilled over P_2O_5. Water was obtained from a Millipore Milli-Q Integral Water Purification System. 18-crown-6 was sublimed. Potassium bicarbonate (≥99.99%) and JandaJel™-polypyridine (100-200 mesh, extent of labeling: ~8.0 mmol/g loading, 1% cross-linked) were purchased from Sigma-Aldrich® and dried at 23 °C for 24 hours under dynamic vacuum (10^{-4} Torr) before use. Cotton was washed with acetone and water and dried at 150°C.

Radiosynthesis of ^{18}F-labeled Molecules

[^{18}F]Fluoride solution obtained from a cyclotron was loaded onto a Macherey-Nagel SPE Chromafix 30-PS-HCO3 cartridge that had been previously washed with 2.0 mL of 5.0 mg/mL $KHCO_3$ in Millipore Milli-Q water and then 20 mL of Millipore Milli-Q water. After loading, the cartridge was washed with 2 mL of Millipore Milli-Q water. [^{18}F]Fluoride was eluted with 2.0 mL of a 5.0 mg/mL $KHCO_3$ in Millipore Milli-Q water solution. The solution was diluted with 8.0 mL of acetonitrile providing 10. mL of 4:1 MeCN:H_2O solution containing 1.0 mg/mL $KHCO_3$. 1.0 mL of this solution was then put in a magnetic-stir-bar-containing conical vial that had been washed with acetone, deionized water, sodium hydroxide/ethanol solution, and deionized water, and dried at 150 °C prior to use. 0.50 mL of a stock solution containing 18-crown-6 (26.2 mg/mL MeCN) was then added. The solution was evaporated at 108 °C with a constant nitrogen gas stream. At dryness, 0.5 mL of acetonitrile was added and evaporated at 108 °C with a constant nitrogen gas stream. Another 0.5 mL of acetonitrile was added and evaporated at 108 °C with a constant nitrogen gas stream to leave a white precipitate around the bottom and sides of the vial. 0.5 mL of acetone was added and evaporated to dryness at 108 °C with a constant nitrogen gas stream to leave a glassy film on the bottom and sides of the vial. The vial was cooled in a water bath, purged with nitrogen, and sealed with a cap fitted with a septum.

First step: 10. mg of Pd(IV) complex **6** dissolved in 0.5 mL of acetone was added via the septum

to the vial. The vial was sonicated and then the reaction mixure was allowed to stir at 23 °C for 10 minutes. During this time, the orange/brown clear solution became opaque. At the end of 10 minutes, the vial was opened and the suspension was loaded with a glass pipette into another glass pipette containing 10 mg of cotton and 25 mg of JandaJel™-polypyridine that had been suspended in 0.3 mL of acetone for 15 minutes (to swell the JandaJel™-polypyridine) and then drained prior to loading the reaction suspension. The conical vial was washed with 0.5 mL of acetone and the acetone wash was added onto the JandaJel™-polypyridine in the glass pipette. At this point the combined reaction suspension and acetone wash were fully pushed through the JandaJel™-polypyridine and cotton with air into a new 1 dram vial equipped with a magnetic stir bar. An additional 0.5 mL of acetone was used to wash the conical vial. The acetone wash was added onto the JandaJel™-polypyridine in the glass pipette and pushed through with air into the 1 dram vial.

Second step: To the 1.5 mL acetone solution was added 10. mg of the Pd(II) aryl complex **15** or **20**. The vial was capped securely, and the mixture heated at 85 °C. After 10 minutes the solution was cooled. A capillary tube was used to spot the solution on a silica gel TLC plate. The TLC plate was emerged in an appropriate organic solvent mixture. The TLC plate was scanned with a Bioscan AR-2000 Radio TLC Imaging Scanner.

Automated syntheses of [^{18}F]-1 and [^{18}F]-2 using high specific activity [^{18}F]fluoride

Automated syntheses were accomplished using Eckert and Ziegler automated synthesis modules and Modular-Lab in a hot cell. Reaction procedures were identical to the described "by hand" method from the preceding section with the following exceptions: 1) The initial azeotrope contained 0.8 mL MeCN and 0.2 mL H_2O instead of 1.3 mL MeCN and 0.2 mL H_2O, 2) the first step of the reaction was stopped after 7.5 minutes instead of 10 minutes, and 3) 1.0 mL of 2-butanone was used to wash the vial and JandaJel™-polypyridine, so that the second step of the reaction proceeded in a 1.5 mL solution of 2:1 2-butanone:acetone. After the reaction, the solution was filtered through preconditioned SepPak® Plus Waters Accell™ Plus QMA cartridge (to capture leftover [^{18}F]fluoride). The second vial and anion exchange cartridge were washed with 1 mL acetone. The combined organic solutions were taken out of the hot cell.

Using manual manipulations, the reaction mixture was concentrated, and the residue was suspended in a solution of EtOAc:hexanes (1.0 mL, 1:1 (v/v)) and filtered through a 1 inch plug of silica gel in a glass pipette using an addition 2.0 mL as eluent. The reaction mixture was concentrated to dryness and the residue was dissolved in a solution of trifluoroacetic acid:CH_2Cl_2 (1.0 mL, 1:1 (v/v)). The solution was immediately concentrated to dryness and the residue was dissolved in a solution of MeCN:H_2O (0.10 mL, 1:3 (v/v)). The sample was then purified by preparative HPLC on a Macherey-Nagel VP 250/10 Nucleosil 100-5 C18 Nautilus column. (For **[^{18}F]-1:** Method: 29% MeCN/H_2O with 0.1% CF_3CO_2H, 5 mL/min, elution time: 13.5–14.5 min, (see Figure S7); For **[^{18}F]-2:** Method: 32% MeCN/H_2O with 0.1% CF_3CO_2H, 5 mL/min, elution

time: 10.7–11.7 min (see Figure S8)) The purified product in HPLC solvent was diluted to 25 mL using a basic aqueous buffer (0.1 M K_2CO_3, 0.1 M $KHCO_3$) was loaded on to a Grace Extract-Clean™ SPE 50mg/1.5mL column. The column was washed with H_2O (10 mL). The product was eluted from the column using EtOH (0.5 mL), diluted with pH 5 0.1 M NaOAc solution (1 mL) and sterile saline solution (3.5–8.5 mL), and finally filtered through a sterile filter (Millex® Syringe-Driven Filter Unit LG 0.2 µm 25mm). For analytical HPLC of final products compared to authentic reference samples, see Figure S9 for **[^{18}F]-1** and Figure S10 for **[^{18}F]-2.** Automated syntheses of **[^{18}F]-1** or **[^{18}F]-2** for NHP PET imaging experiments were conducted four times, twice each, furnishing 16 and 13 mCi of **[^{18}F]-1** and 14 and 10 mCi of **[^{18}F]-2** before filtration through a sterile filter.

Figure S7. Preparatory HPLC chromatograph for synthesis of [^{18}F]-1

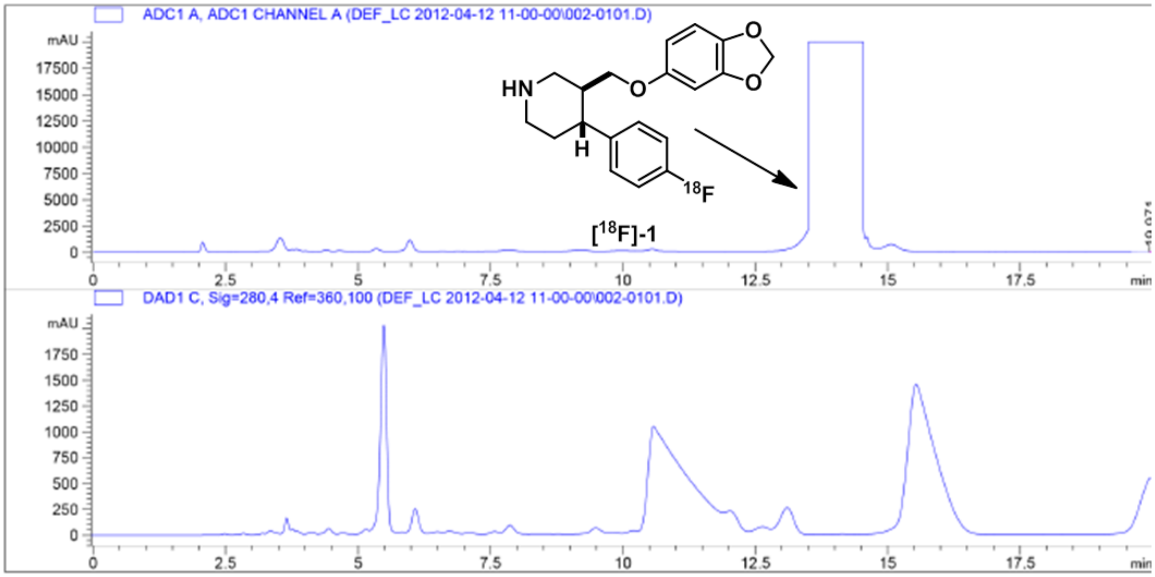

Radioactivity trace (top) and 280 nm UV trace (bottom) demonstrating preparatory HPLC purification. Sample **[^{18}F]-1** collected at 13.5–14.5 min corresponding to large (saturated) radioactivity peak.

Figure S8. Preparatory HPLC chromatograph for synthesis of [^{18}F]-2

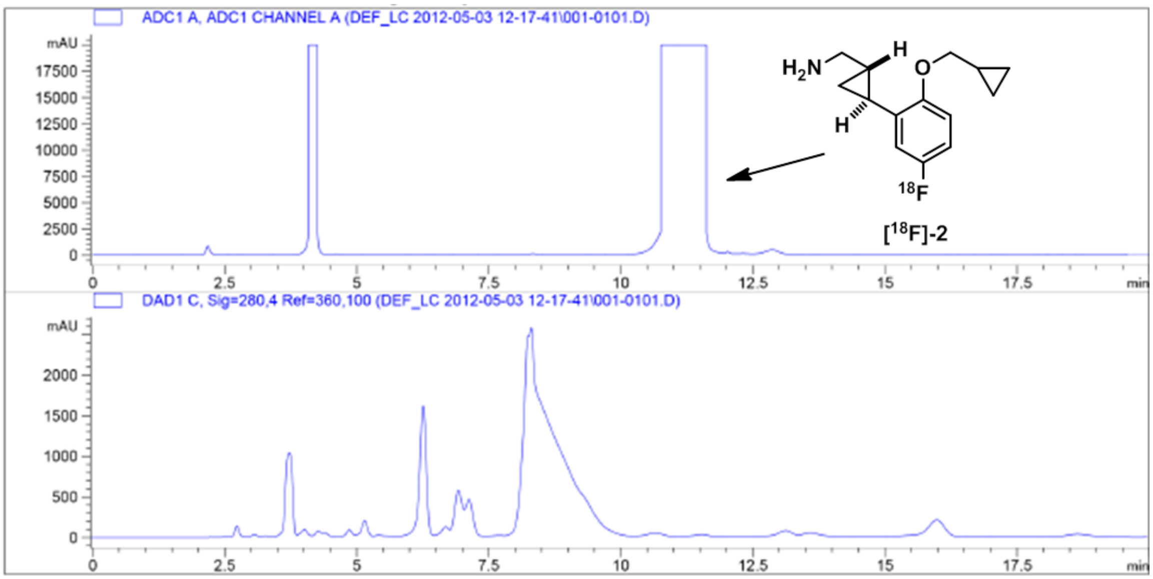

Radioactivity trace (top) and 280 nm UV trace (bottom) demonstrating preparatory HPLC purification. Sample **[^{18}F]-2** collected at 10.7–11.7 min corresponding to large (saturated) radioactivity peak.

Characterization of ^{18}F-labeled molecules

^{18}F-labeled molecules were characterized by comparing the radioactivity HPLC trace of the reaction mixture to the HPLC UV trace of authentic reference sample. An Agilent Eclipse XDB-C18, 5 µm, 4.6 x 150 mm HPLC column was used for analytical HPLC analysis. Note: radioactivity chromatographs have been offset (–0.125 min) to account for the delay volume (time) between the diode array detector and the radioactivity detector.

Figure S9. Characterization of purified [^{18}F]-1

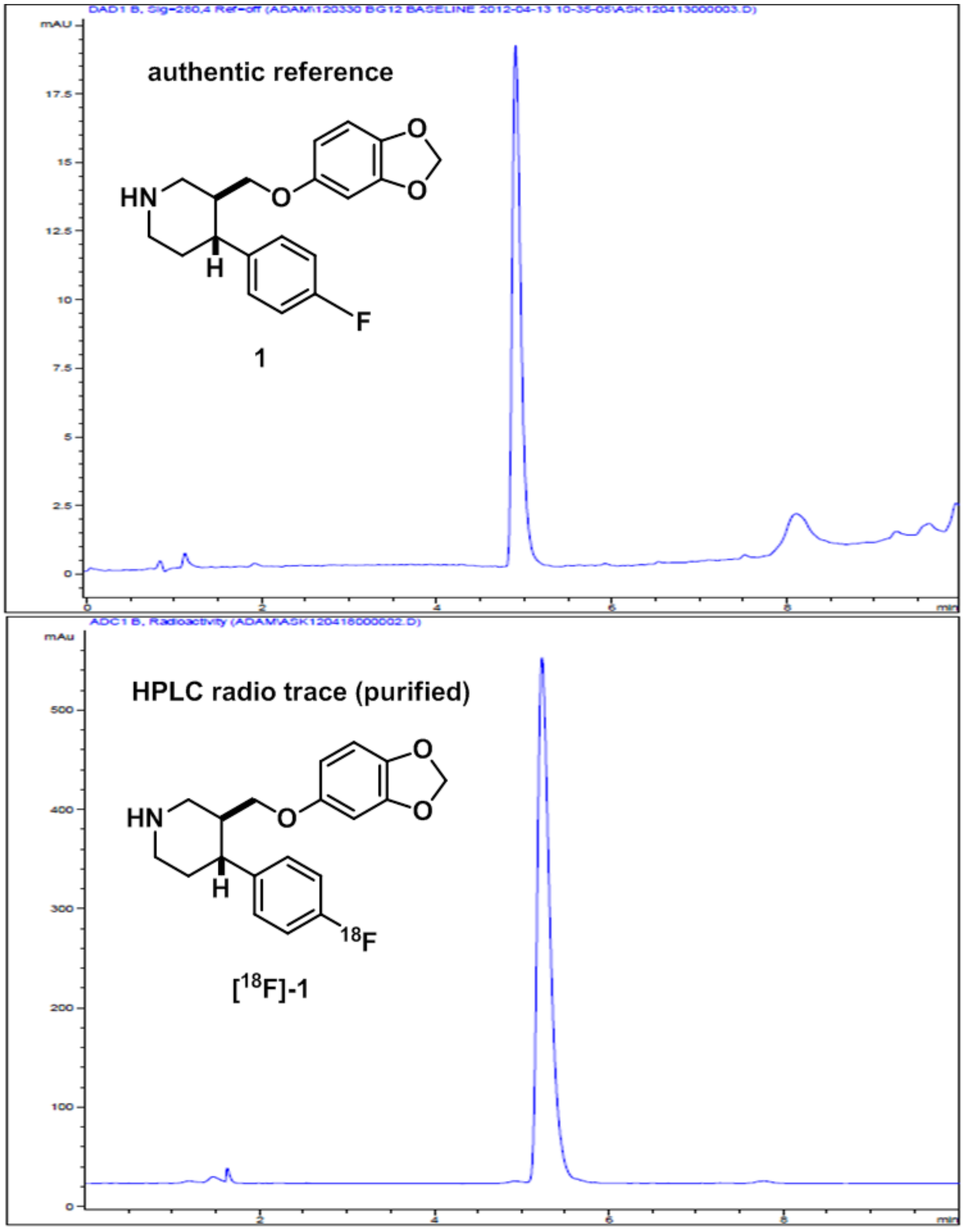

280 nm trace (top) of authentic sample of **1** and radioactivity trace of purified **[^{18}F]-1**. Note: radioactivity chromatographs have been not been offset (–0.125 min) to account for the delay volume (time) between the diode array detector and the radioactivity detector.

Figure S10. Characterization of purified [^{18}F]-2

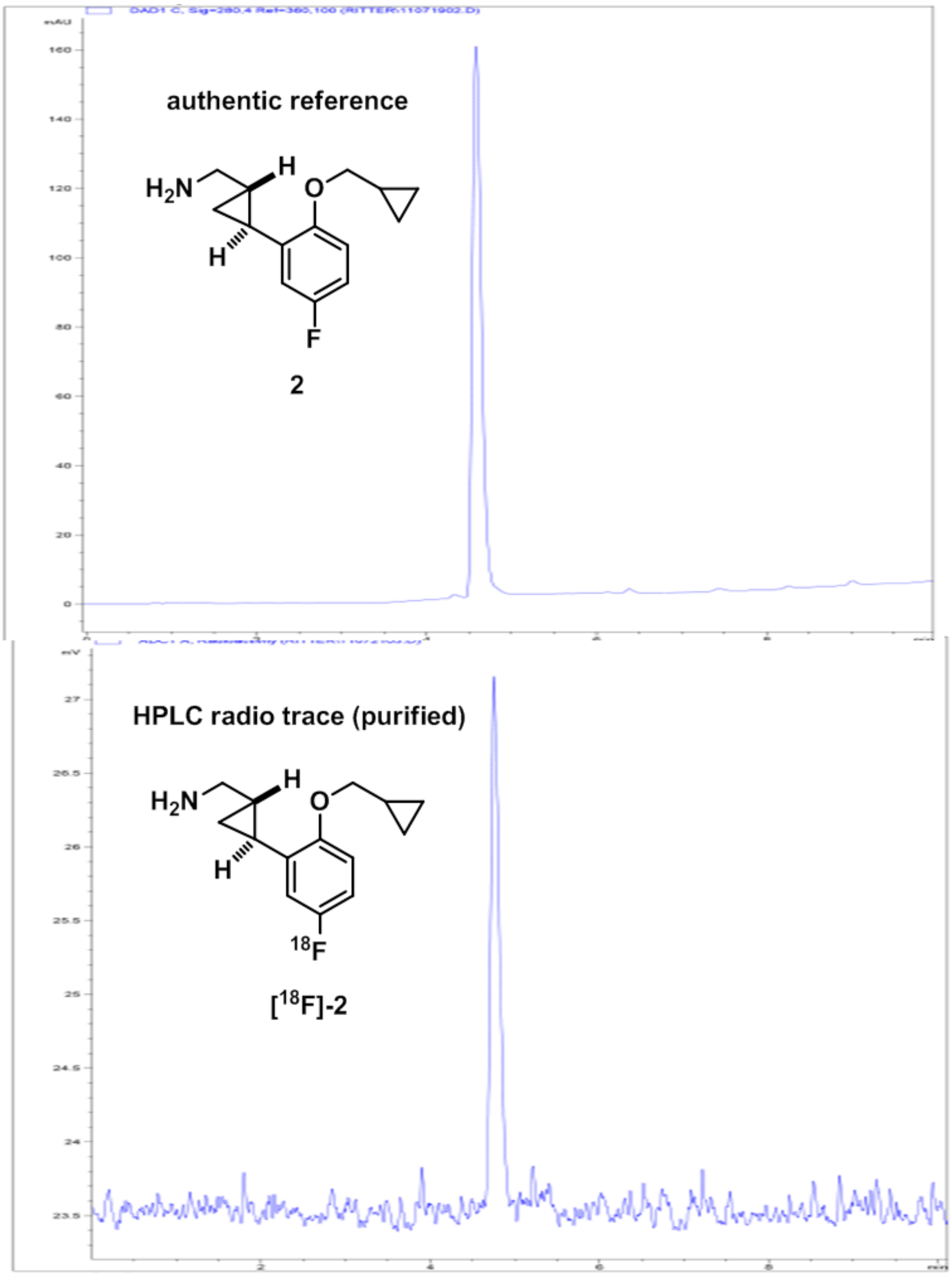

280 nm UV trace (top) of authentic sample of **2** and radioactivity trace of purified **[^{18}F]-2**. Note: radioactivity chromatographs have been not been offset (–0.125 min) to account for the delay volume (time) between the diode array detector and the radioactivity detector.

Determination of specific activity of [^{18}F]-1and [^{18}F]-2

Specific activity of **[^{18}F]-1** and **[^{18}F]-2** were determined by measuring the UV absorbance of a known amount of radioactivity and comparing to a standard curve of UV absorbance vs amount of unlabeled **1** and **2.** For 89 µCi of **[^{18}F]-1** a UV absorbance could not be detected. The smallest amount that has been detected corresponded to 7 pmol for a specific activity of at least 13 Ci/µmol at time of injection (TOI). For 122 µCi of **[^{18}F]-2** a UV absorbance of 5.1 was measured corresponding to 120 pmol for a specific activity of 1.0 Ci/µmol at time of injection (TOI). The standard curves were generated by integration of the UV absorbance signal (at 280 nm) of at least 5 different known amounts of **1** and **2** in triplicate (see Tables S1 and S2 and Figures S11 and S12).

Table S1. Data for standard curve of UV absorbance vs amount of 1

pmol **1**	UV Absorbance
7	2.0
26	8.0
65	25.7
260	133.0
651	341.6
2603	1439.2

Figure S11. Standard curve of UV absorbance vs amount of 1

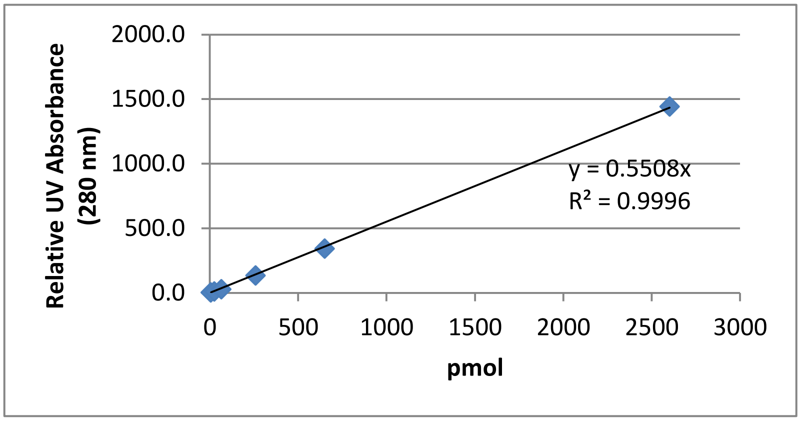

Table S2. Data for standard curve of UV absorbance vs amount of 2

pmol **2**	UV Absorbance
29	0.93
72	1.8
286	8.4
716	27.3
2863	128.1

Figure S12. Standard curve of UV absorbance vs amount of 2

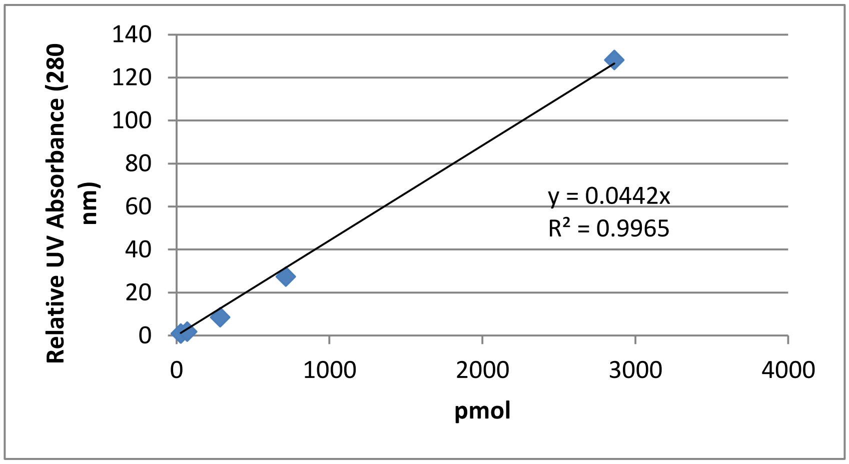

Determination of palladium content in purified sample of [^{18}F]-2

At the end of the synthesis and reformulation of **[^{18}F]-2**, A portion of the sample was analyzed using an Agilent 7500a ICP-MS to determine palladium content. An average of 2 samples (HPLC fraction:ICP diluent 1:99 (m/m)) were compared to a standard curve of relative ion count versus palladium concentration. The standard curve was generated by creating a dilution series of known palladium concentrations (from 0.020 ppb to 50.0 ppb) and an internal lutetium control (see Table S3 and Figure S13). The samples averaged less than 0.02 ppb palladium. The final palladium content of the reformulated sample was 2 ppb palladium.

Table S3. ICP/MS data for standard curve and reformulated samples

ppb Pd	Pd-105 cps	Lu cps	Pd-105/Lu
0.020	204.44	1337281	0.00015288
0.10	801.67	1338268	0.00059904
0.50	4074.44	1346762	0.0030254
2.0	14839.44	1333299	0.011130
10.	77070.56	1322516	0.058276
50.	290764.30	1326804	0.21915
sample 1	62.22	1324434	.000046979
sample 2	69.44	1293053	.000053702

ppb = parts per billion, cps = counts per second

Figure S13. Standard curve of relative ion count vs palladium concentration

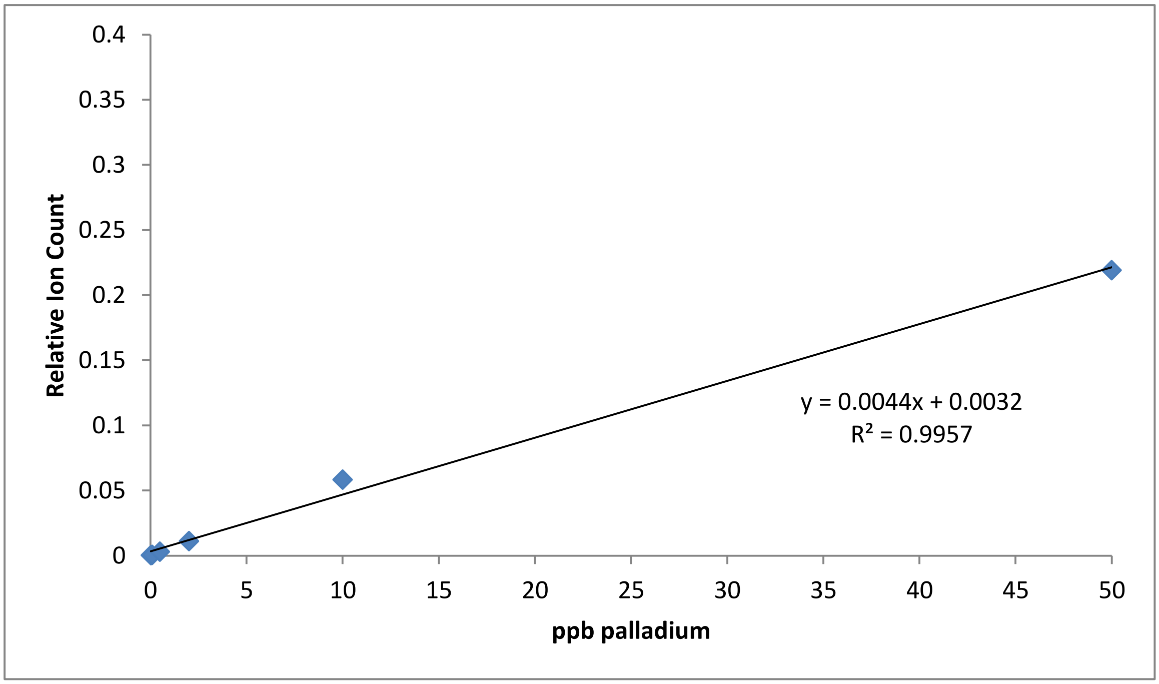

Baboon PET Imaging Experiments

For each study a *Papio anubis* baboon, deprived of food for 12 h prior to the study, was administered intramuscular atropine, diazepam and ketamine successively to induce anesthesia for intubation and transportation from the animal housing facility to the MR-PET scanner. For maintenance of anesthesia throughout the study, the baboon was provided nominally 1% (Forane) in a mixture of medical oxygen (1 L/min). The baboon was catheterized antecubitally for radiotracer injection and a radial arterial line was placed for input function and metabolite analyses. Heart rate, respiration, $ETCO_2$, and blood pressure were monitored and recorded every 5-15 min throughout the study.

MR-PET images were acquired in a Biograph mMR scanner (Siemens, Munich, Germany), with a PET resolution of approximately 6 mm and field of view of 59.4 cm and 25.8 cm (transaxial and axial, respectively). Dynamic PET image acquisition was carried out during and following administration of the radiotracer. When arterial sampling was possible, blood was collected nominally every 10 sec for 3 min to determine the input of the radiotracer. Addition samples were drawn at 5, 10, 20, 30, 45, 60, and 90 min time points for radioactive metabolite analysis. An MEMPRAGE sequence was acquired during the dynamic PET acquisition 30 min during each scan for anatomic coregistration. To characterize the specific binding of **[^{18}F]-1** and **[^{18}F]-2** , the same protocol was following with citalopram (5 mg/kg) or ritanserin (0.1 mg/kg), respectively. These were administered intravenously 10 min before the start of acquisition (time of radiotracer injection). The injected doses were 4.38 mCi (baseline) and 3.40 mCi (blocking experiment) for **[^{18}F]-1** and 4.47 mCi (baseline) and 4.93 mCi (blocking experiment) for **[^{18}F]-2**. Image reconstruction was accomplished using the software provided by Siemens for the Biograph mMR with post acquisition determination of time frames. Analysis was performed using PMOD® software version 3.3.

Spectroscopic Data

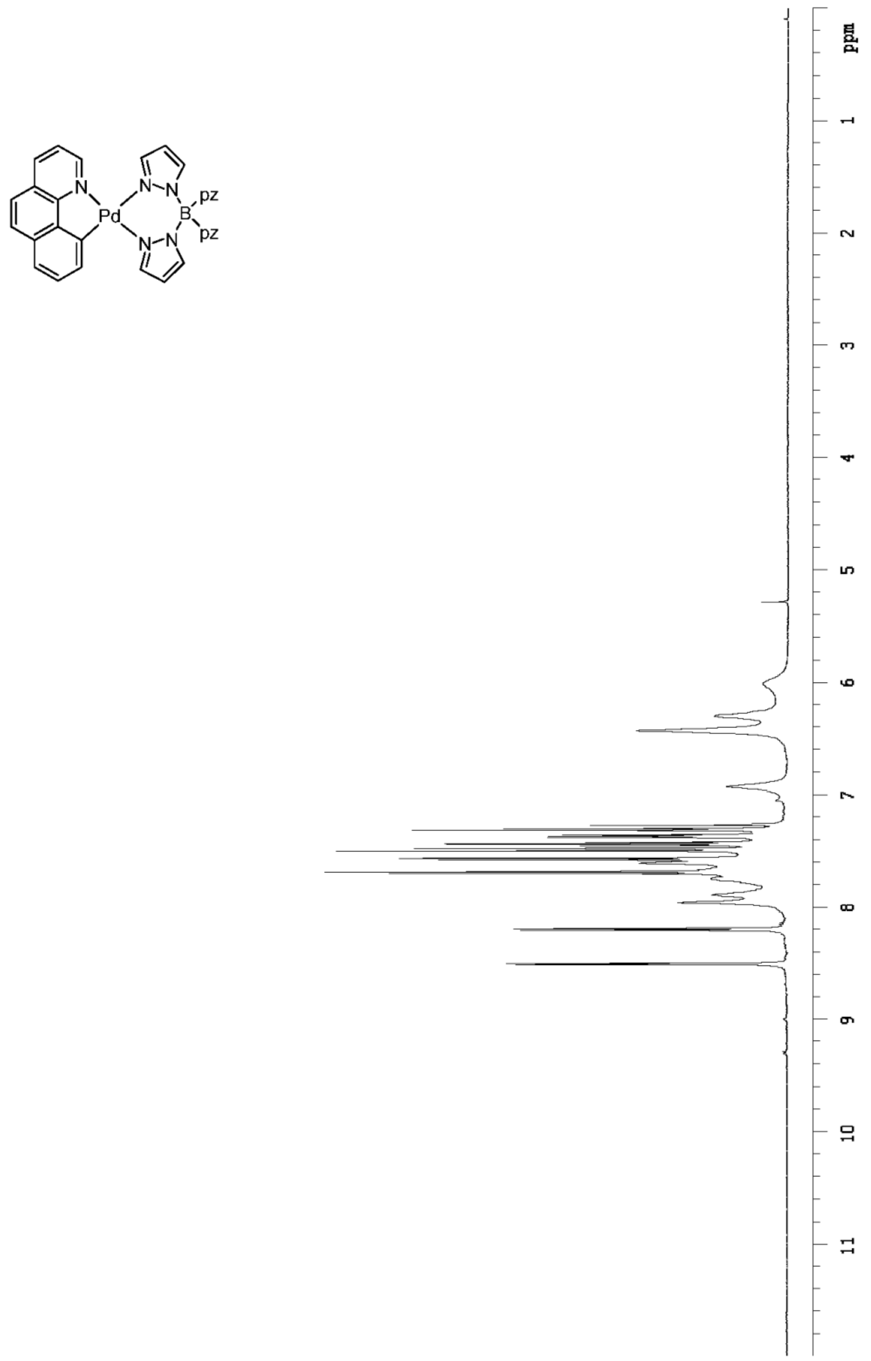

^{1}H NMR ($CDCl_3$, 23 ºC) of **3**

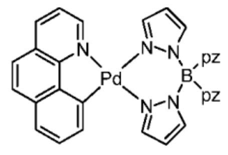

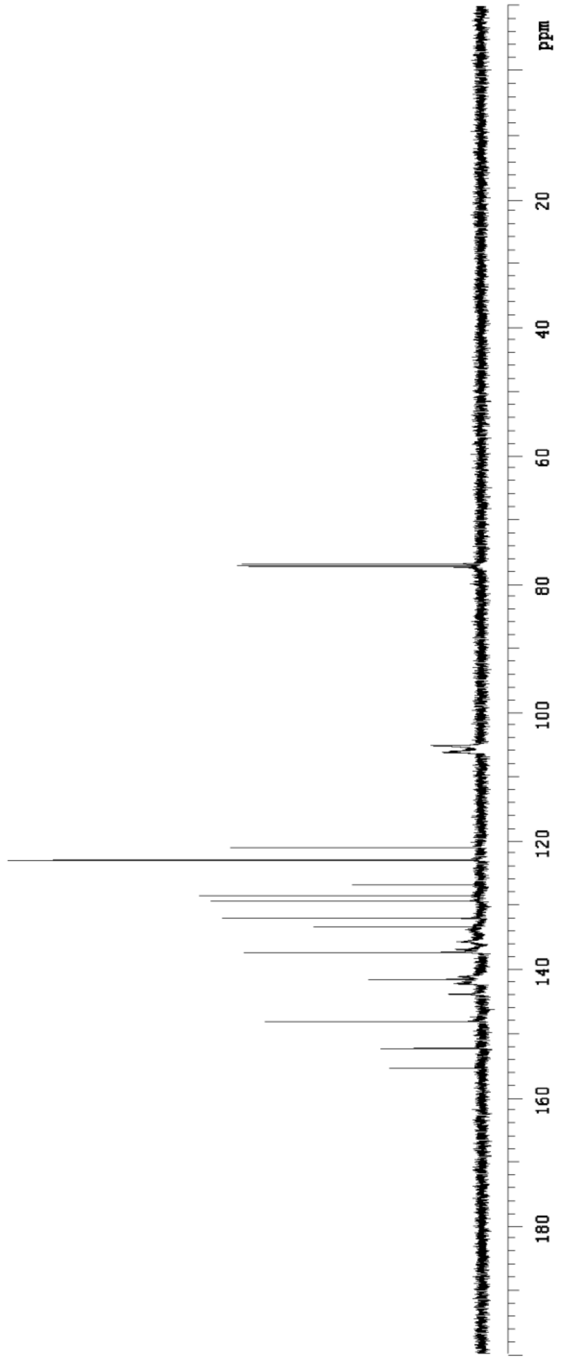

^{13}C NMR ($CDCl_3$, 23 ºC) of **3**

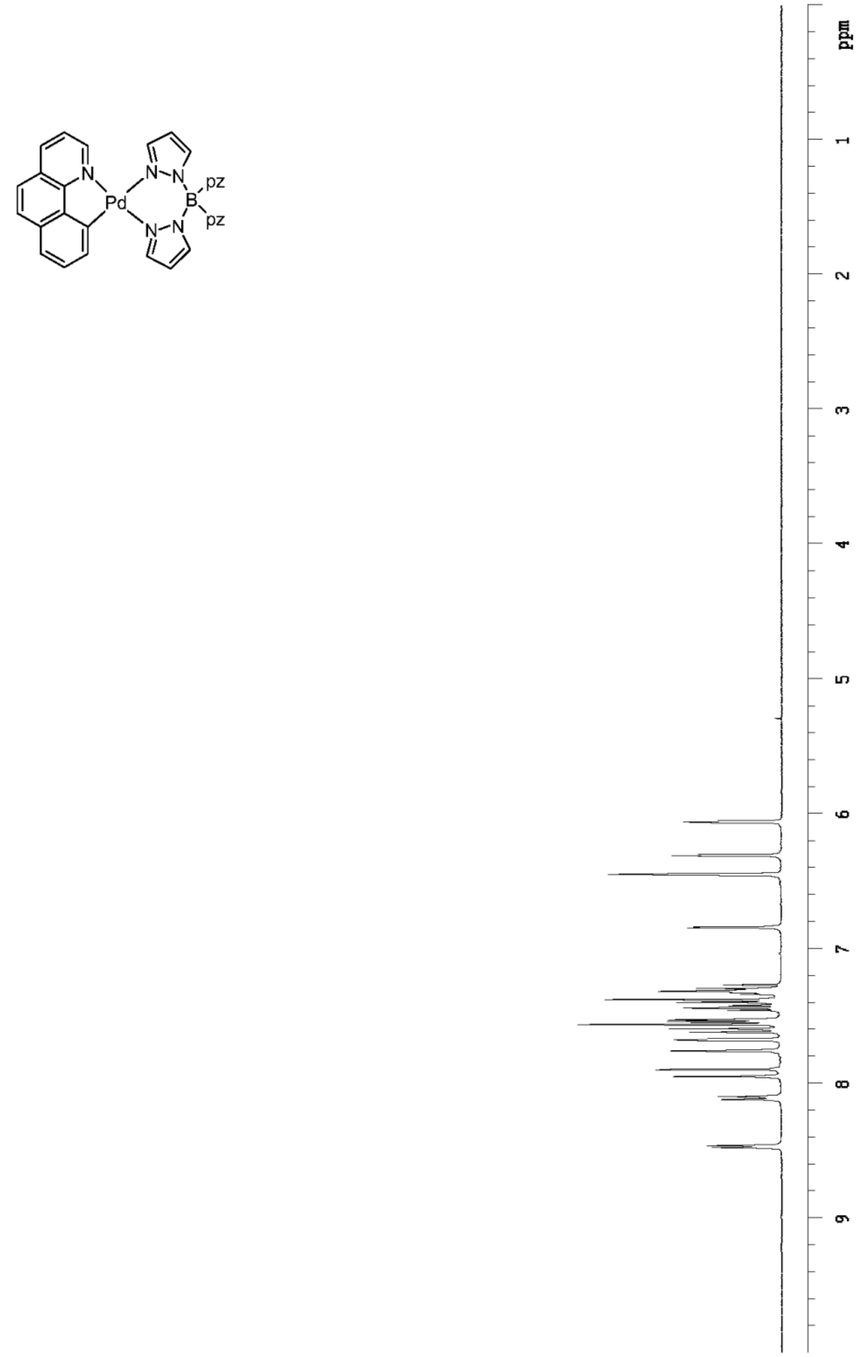

^{1}H NMR ($CDCl_3$, −25 ºC) of **3**

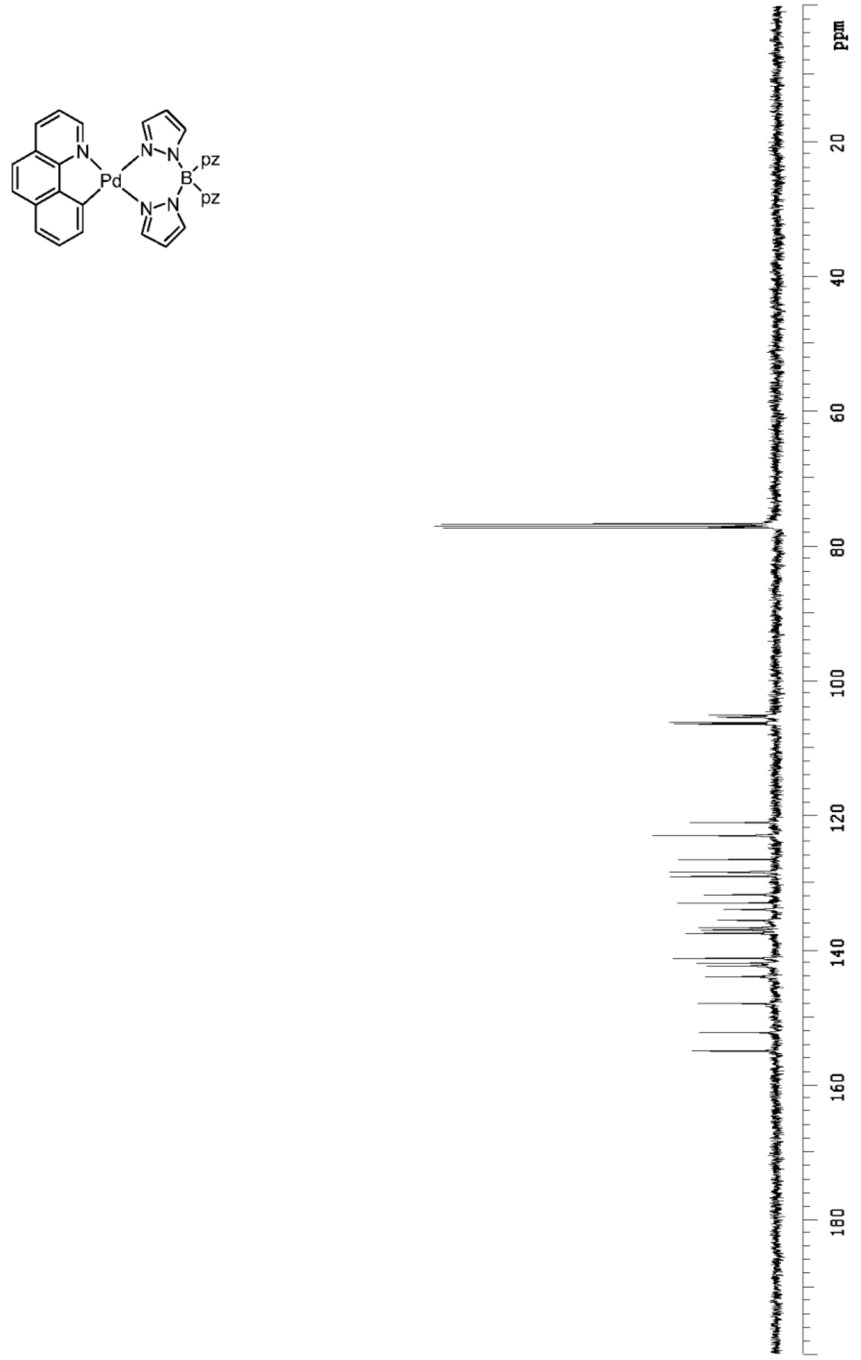

^{13}C NMR ($CDCl_3$, −25 ºC) of **3**

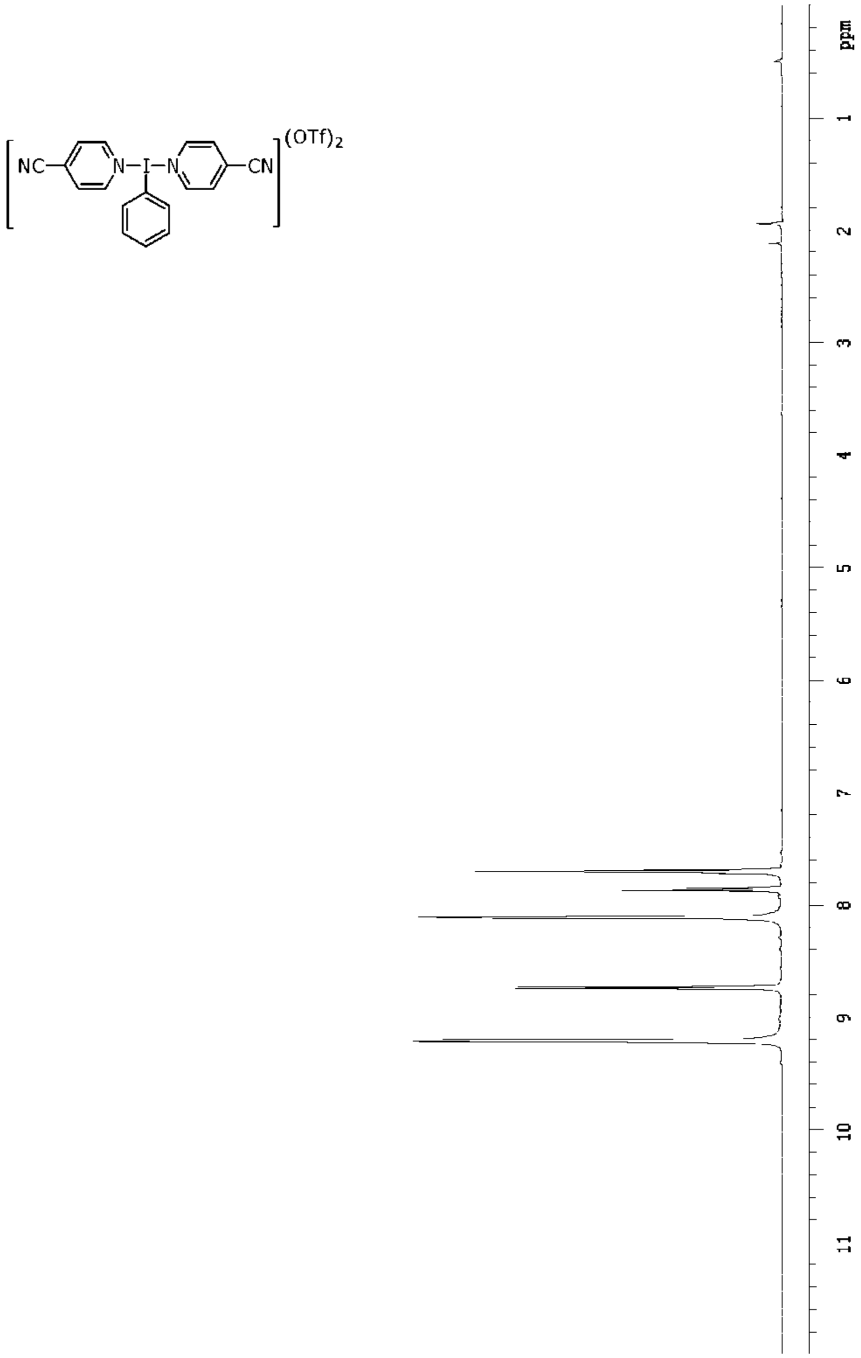

^{1}H NMR (CD_3CN, 23 ºC) of **4**

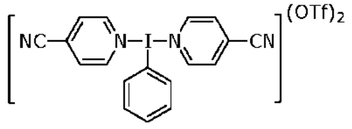

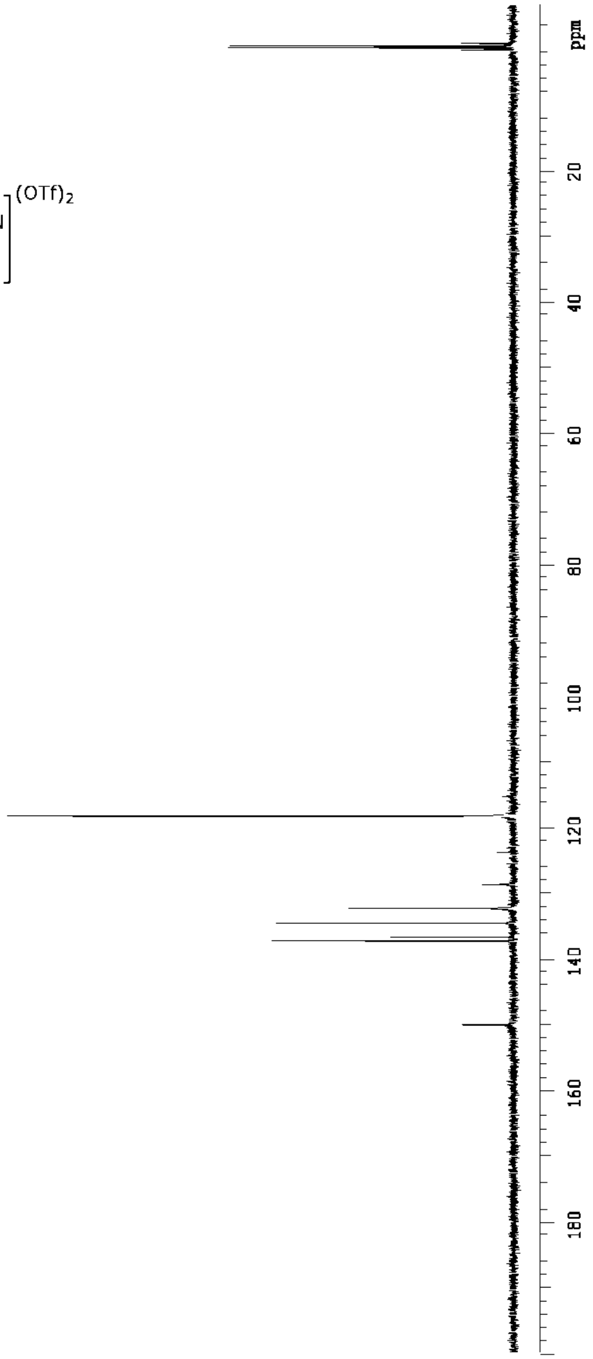

^{13}C NMR (CD_3CN, 23 ºC) of **4**

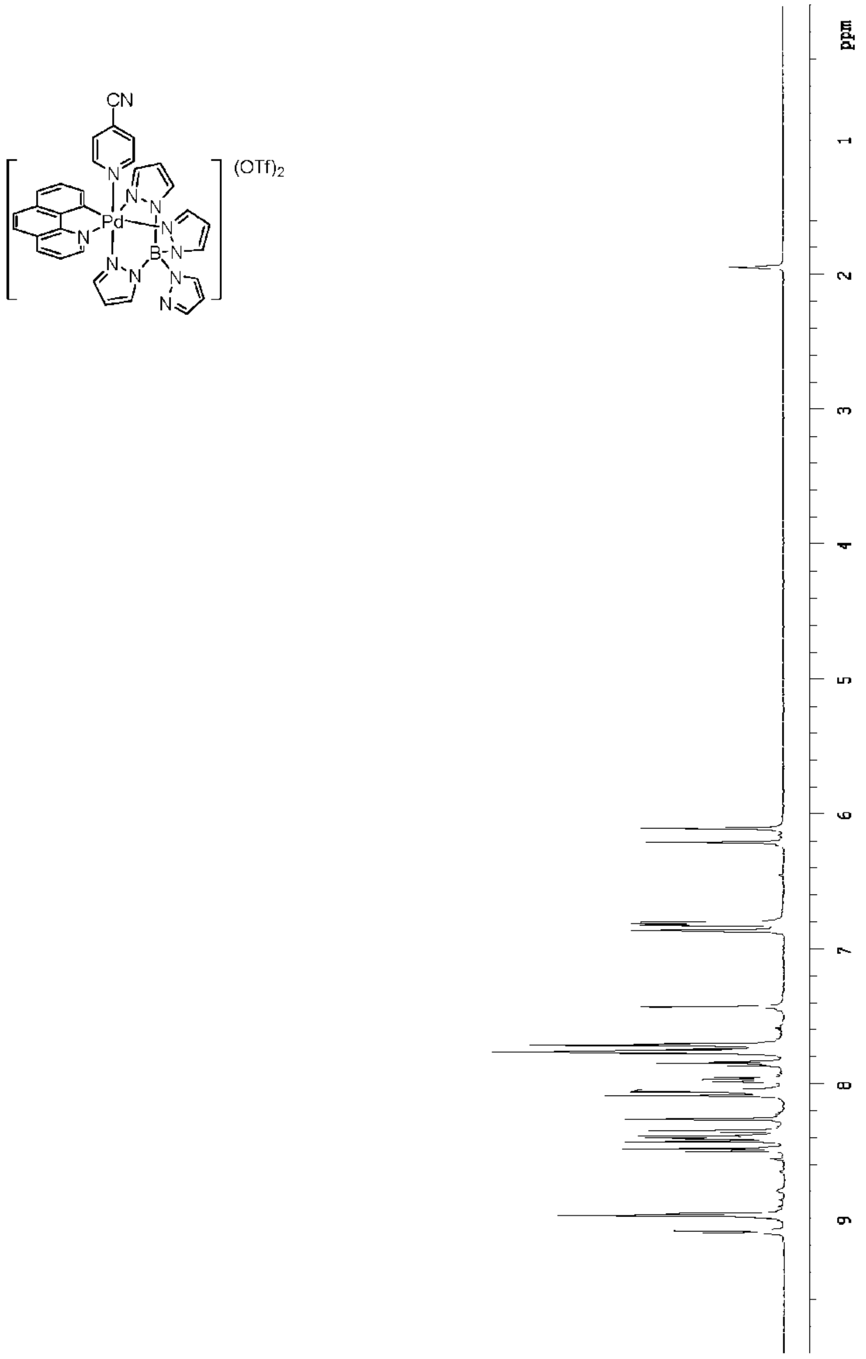

^{1}H NMR (CD_3CN, 23 ºC) of **5**

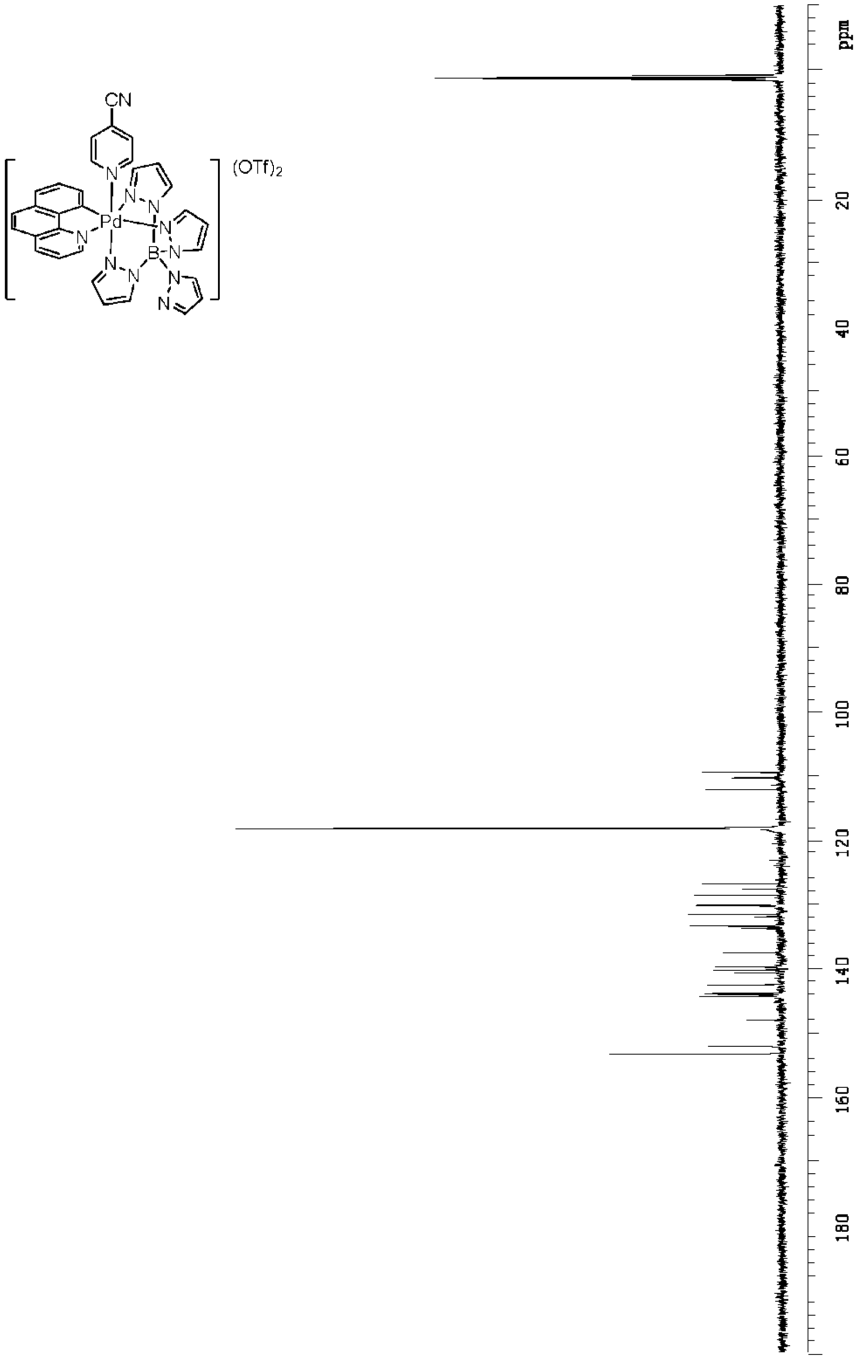

^{13}C NMR (CD_3CN, 23 ºC) of **5**

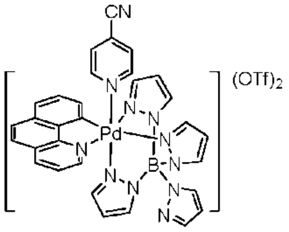

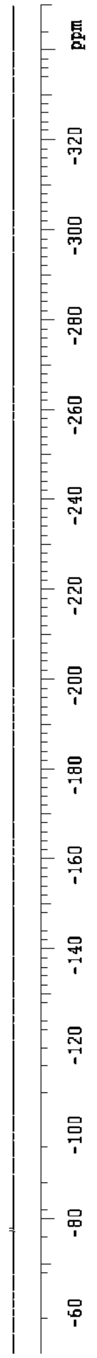

^{19}F NMR (CD_3CN, 23 ºC) of **5**

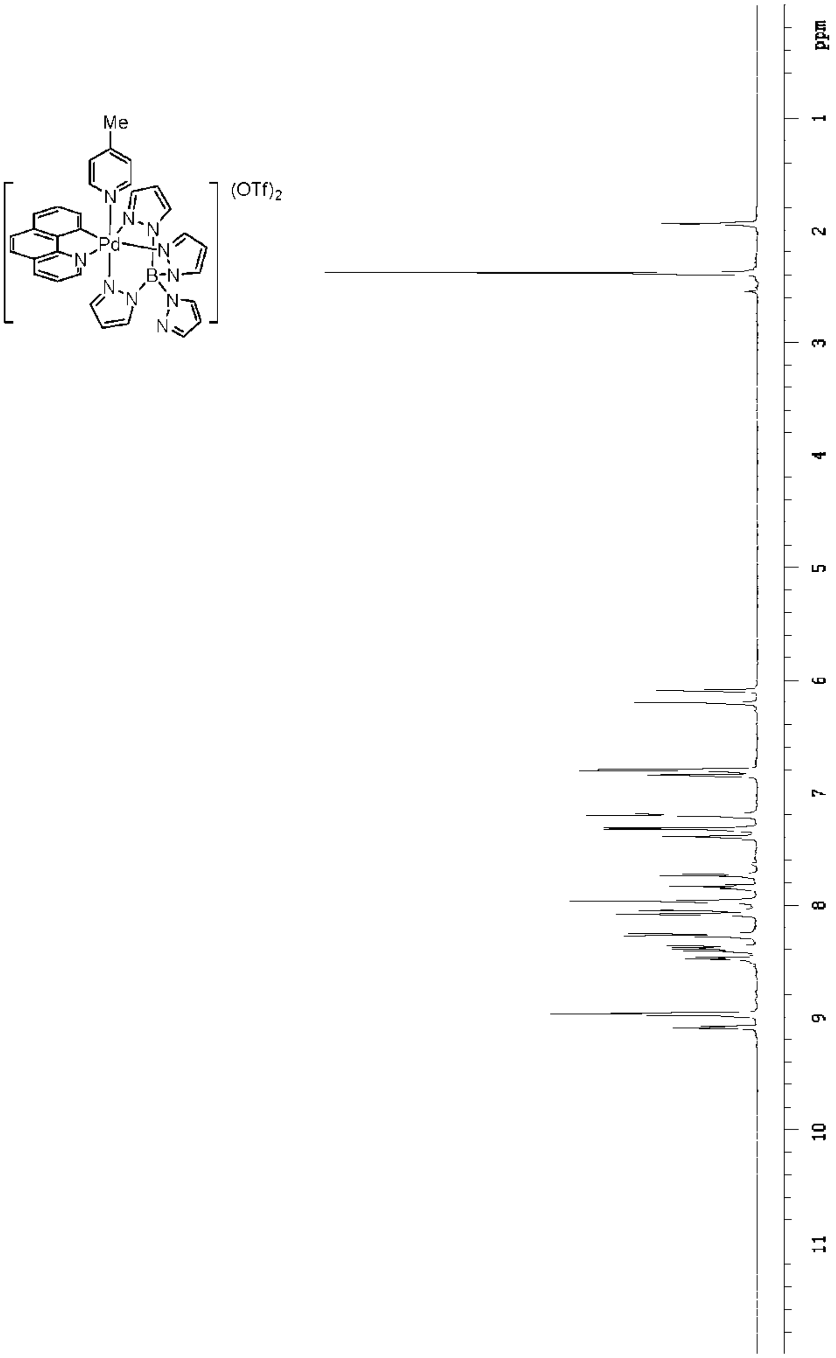

^{1}H NMR (CD_3CN, 23 ºC) of **6**

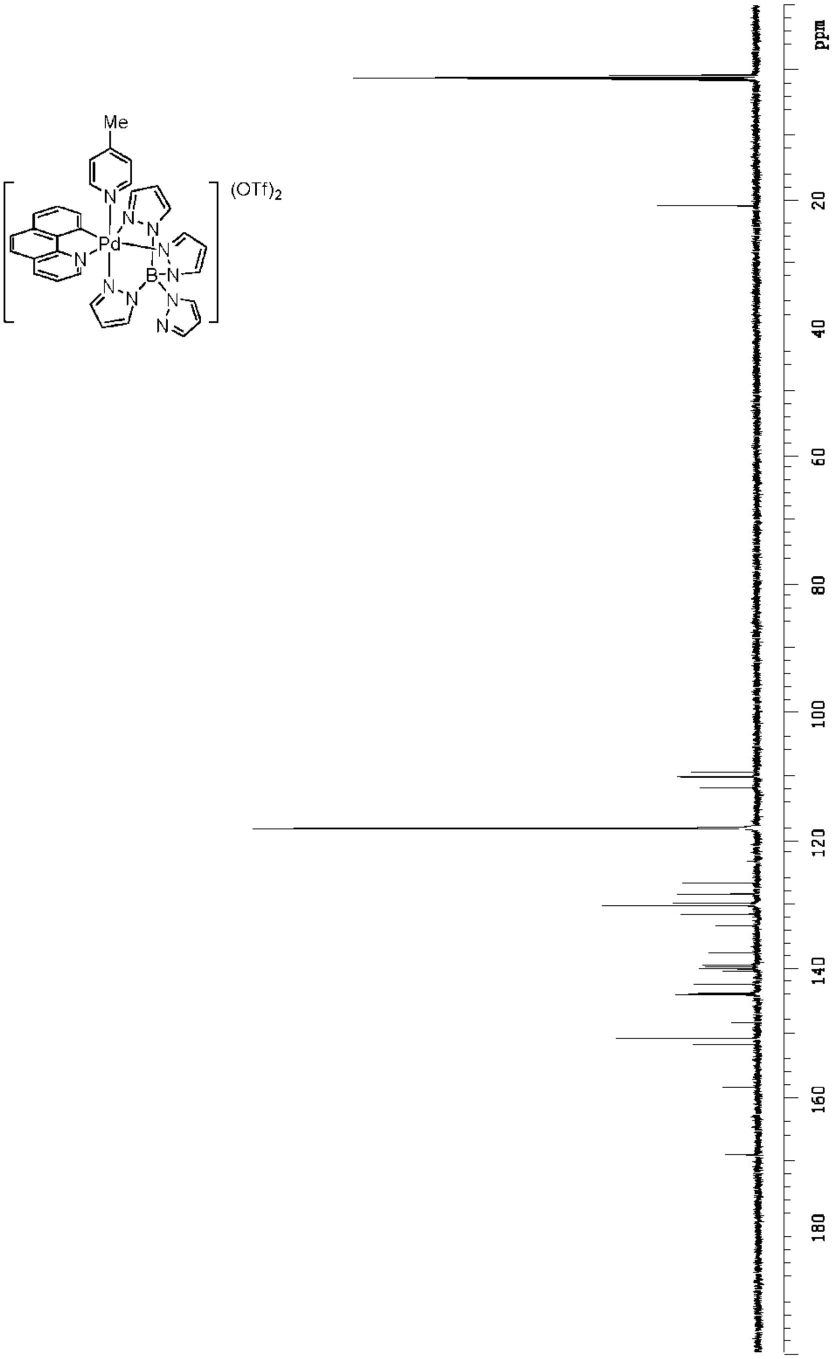

^{13}C NMR (CD_3CN, 23 ºC) of **6**

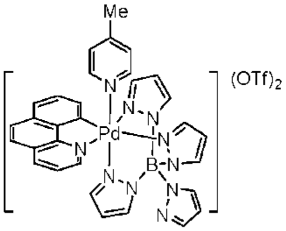

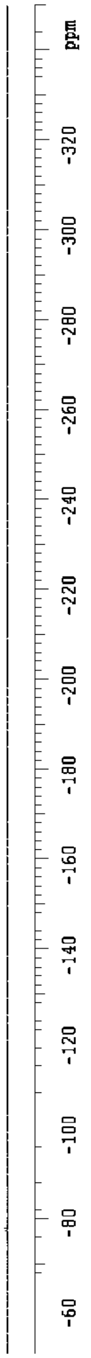

^{19}F NMR (CD_3CN, 23 °C) of **6**

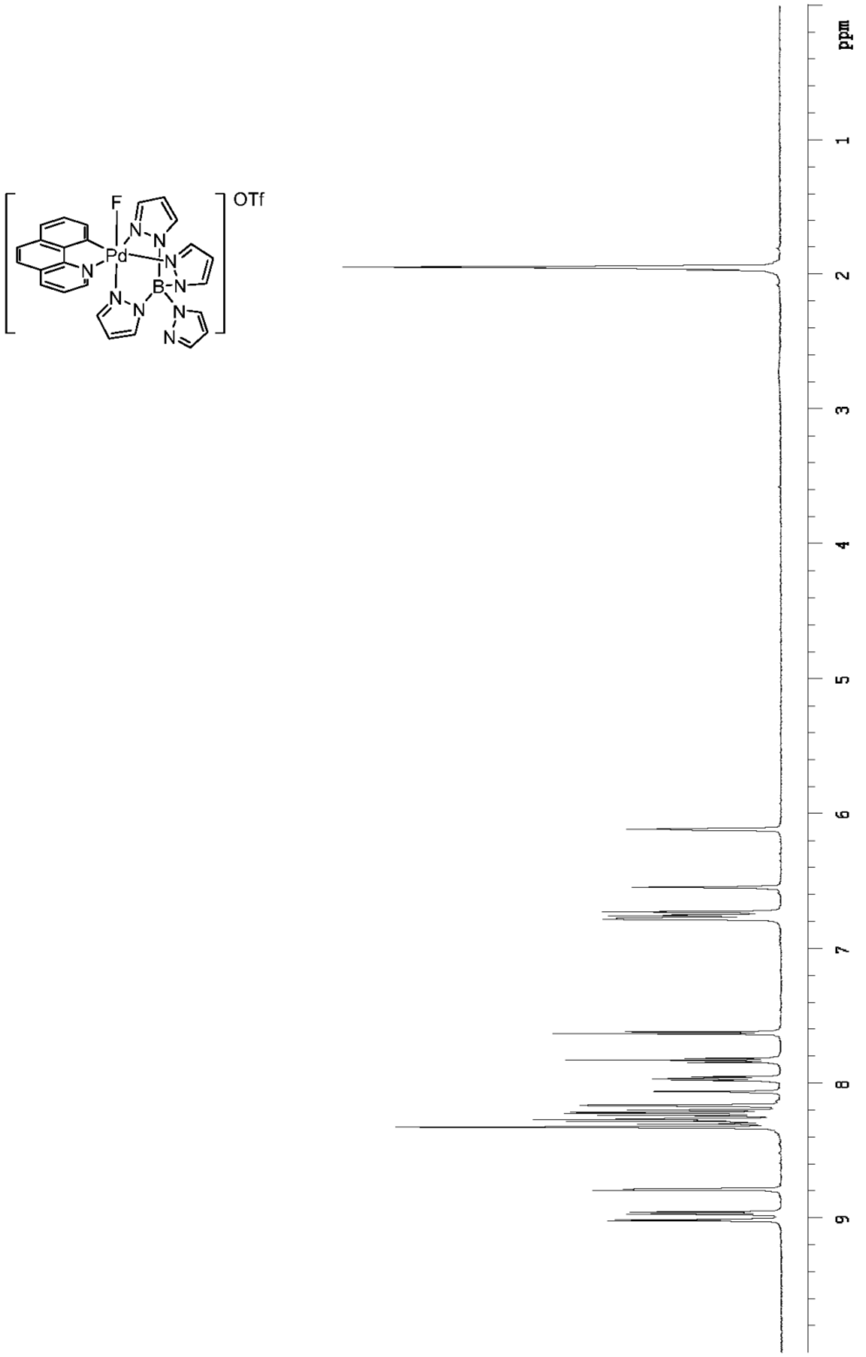

^{1}H NMR (CD_3CN, 23 ºC) of **7**

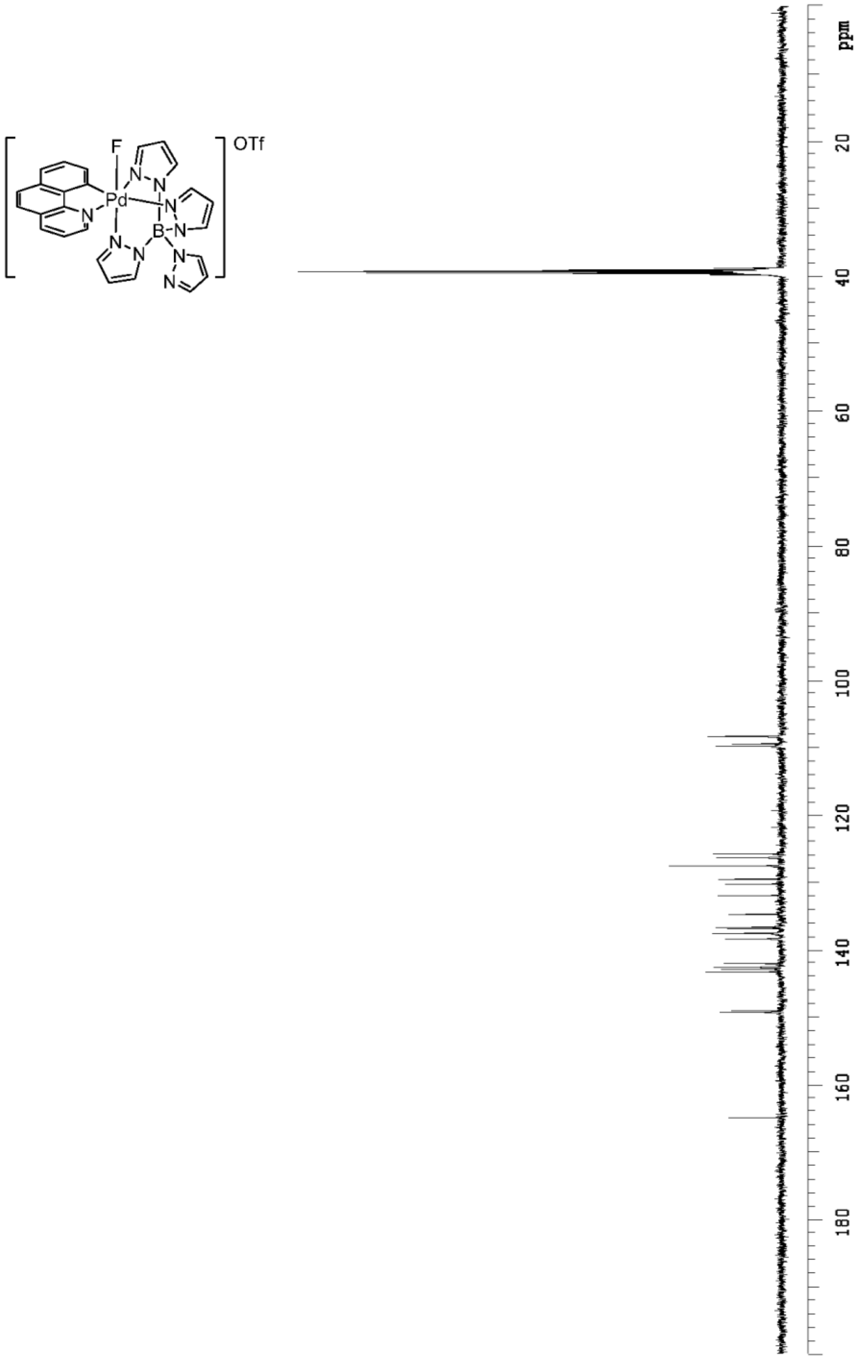

^{13}C NMR (DMSO-d_6, 23 ºC) of **7**

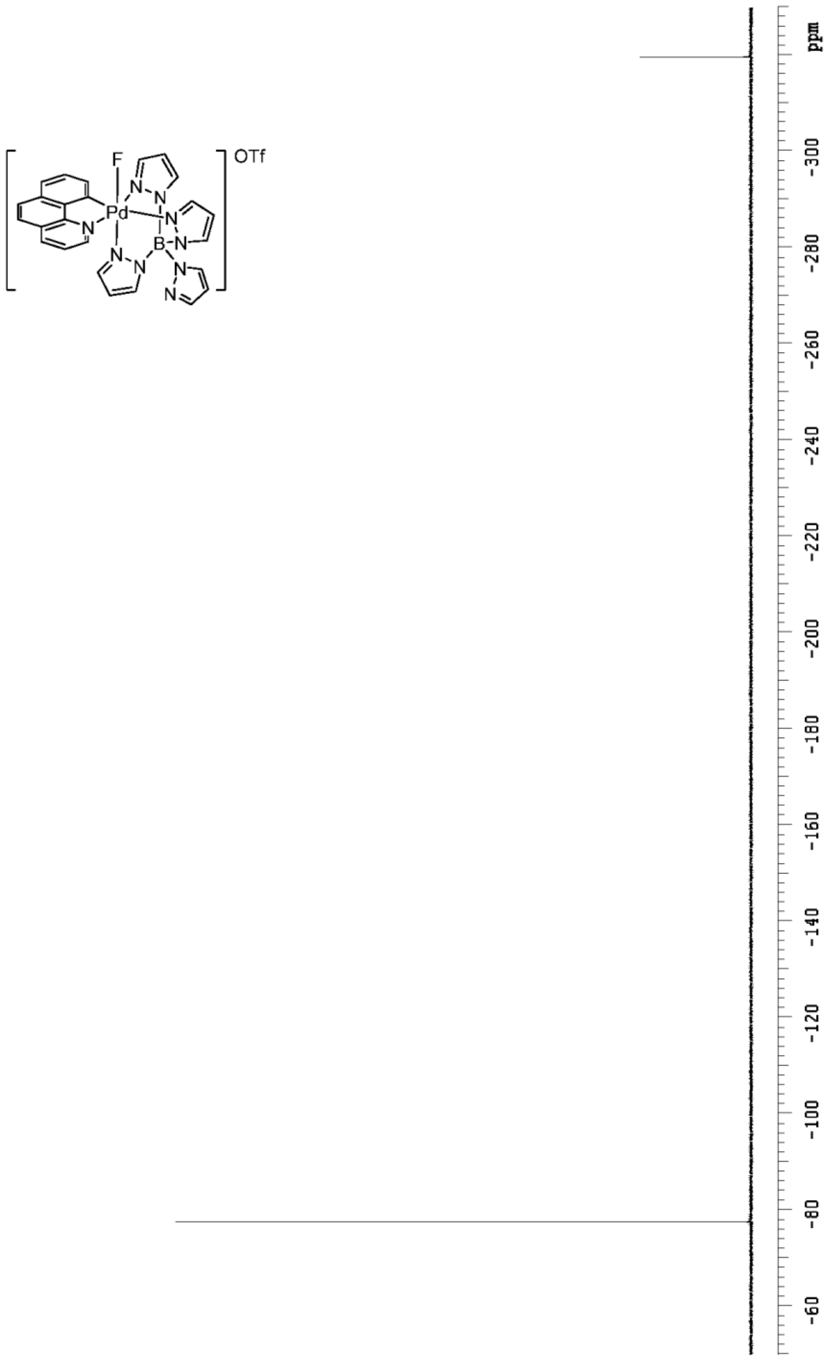

^{19}F NMR (CD_3CN, 23 ºC) of **7**

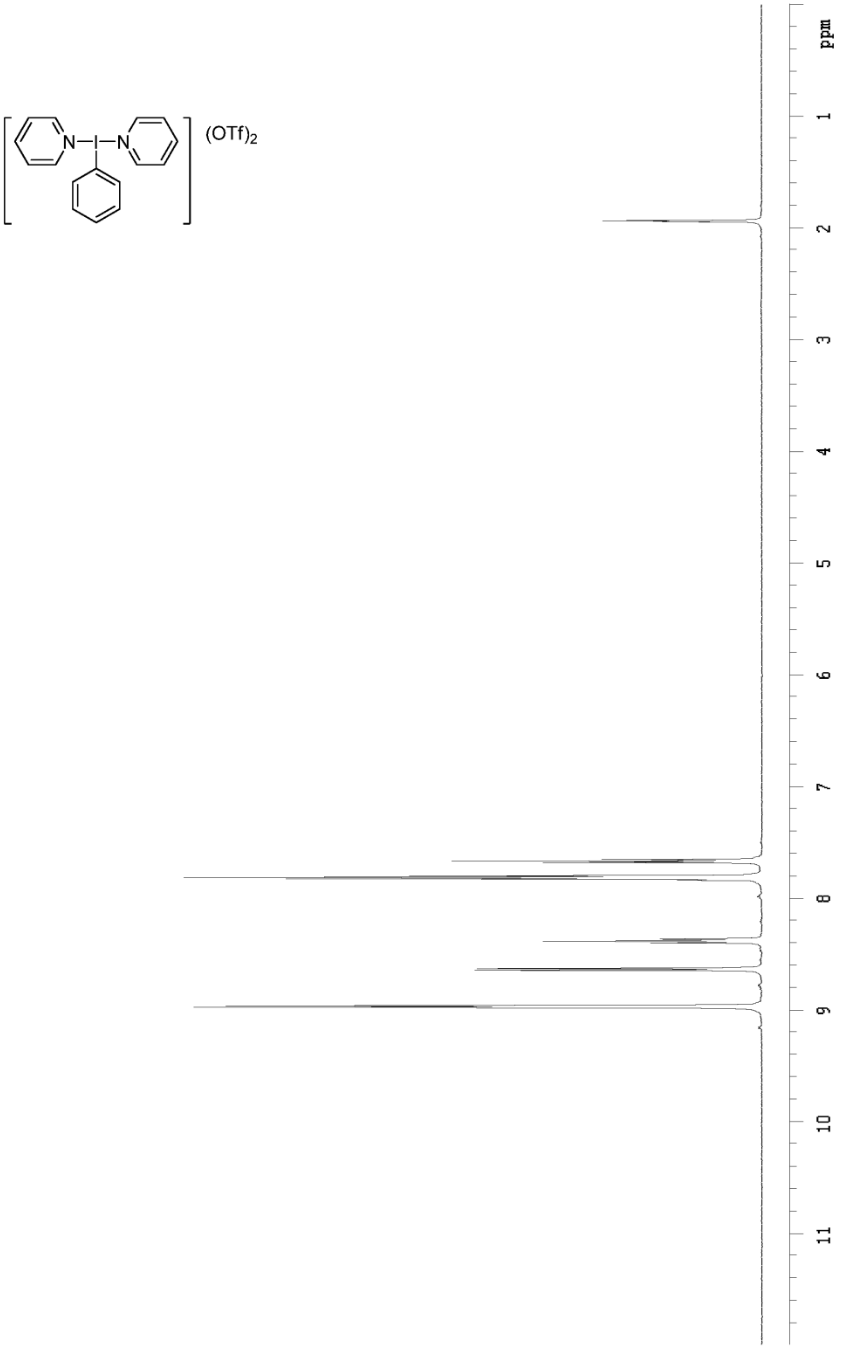

^{1}H NMR (CD_3CN, 23 ºC) of **S3**

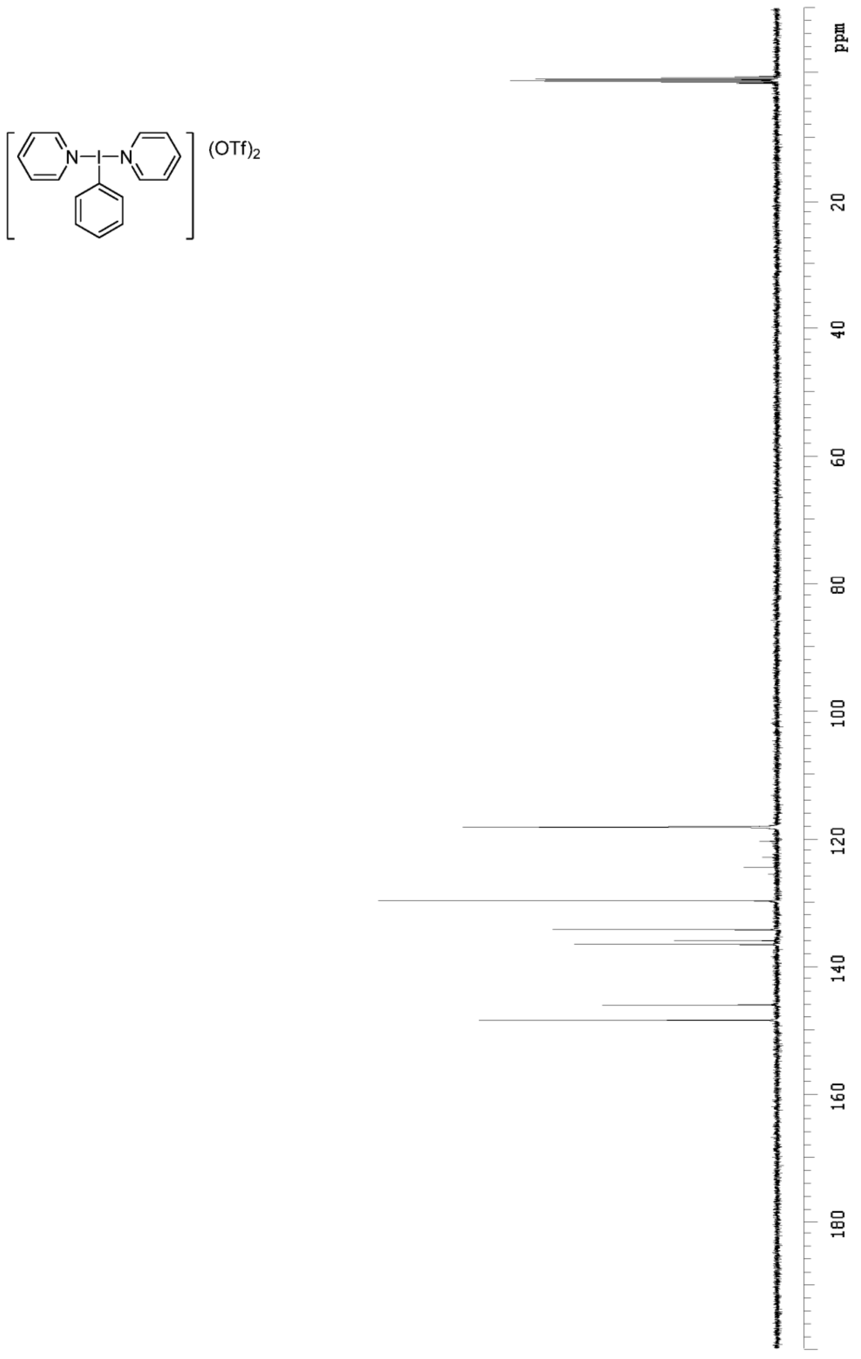

^{13}C NMR (CD_3CN, 23 ºC) of **S3**

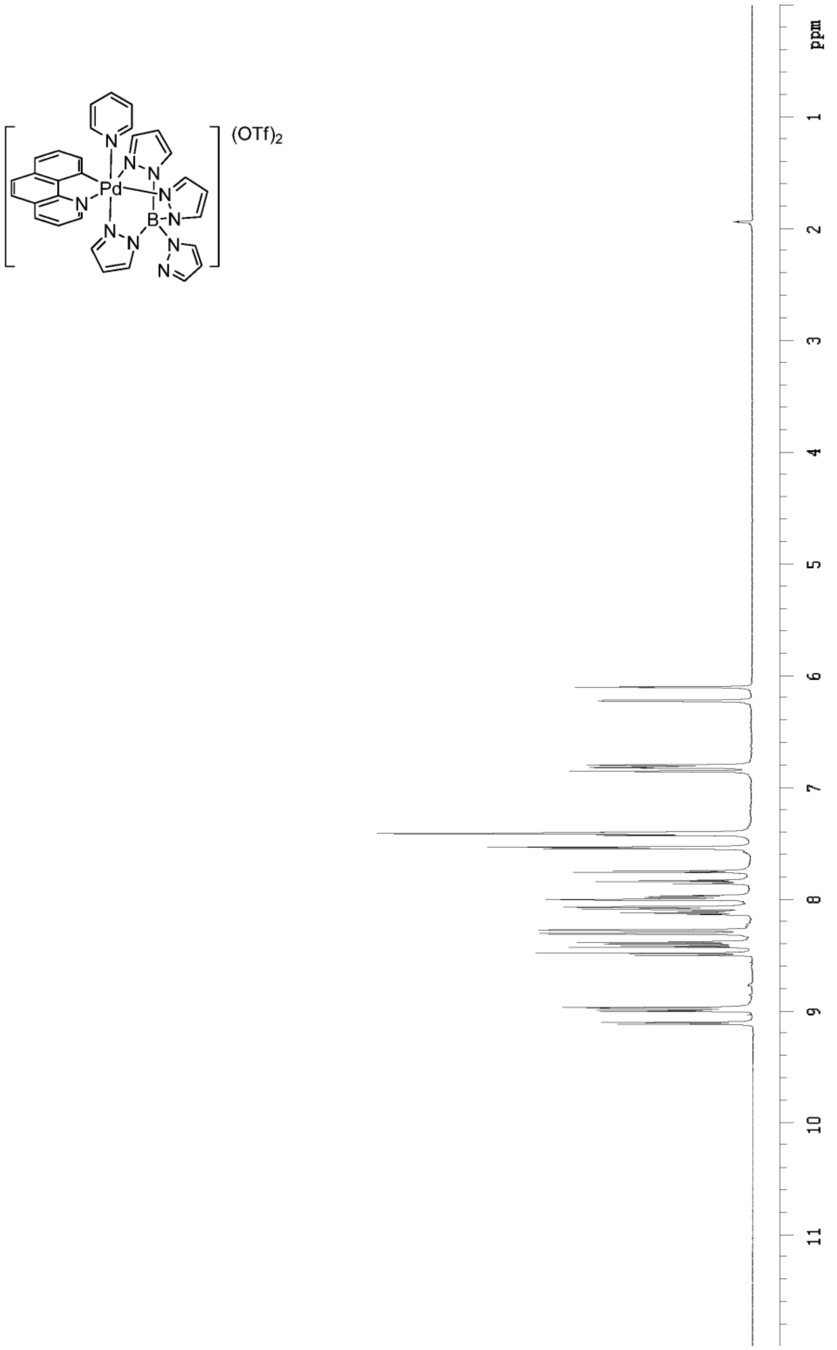

^{1}H NMR ($CDCl_3$, 23 ºC) of **S4**

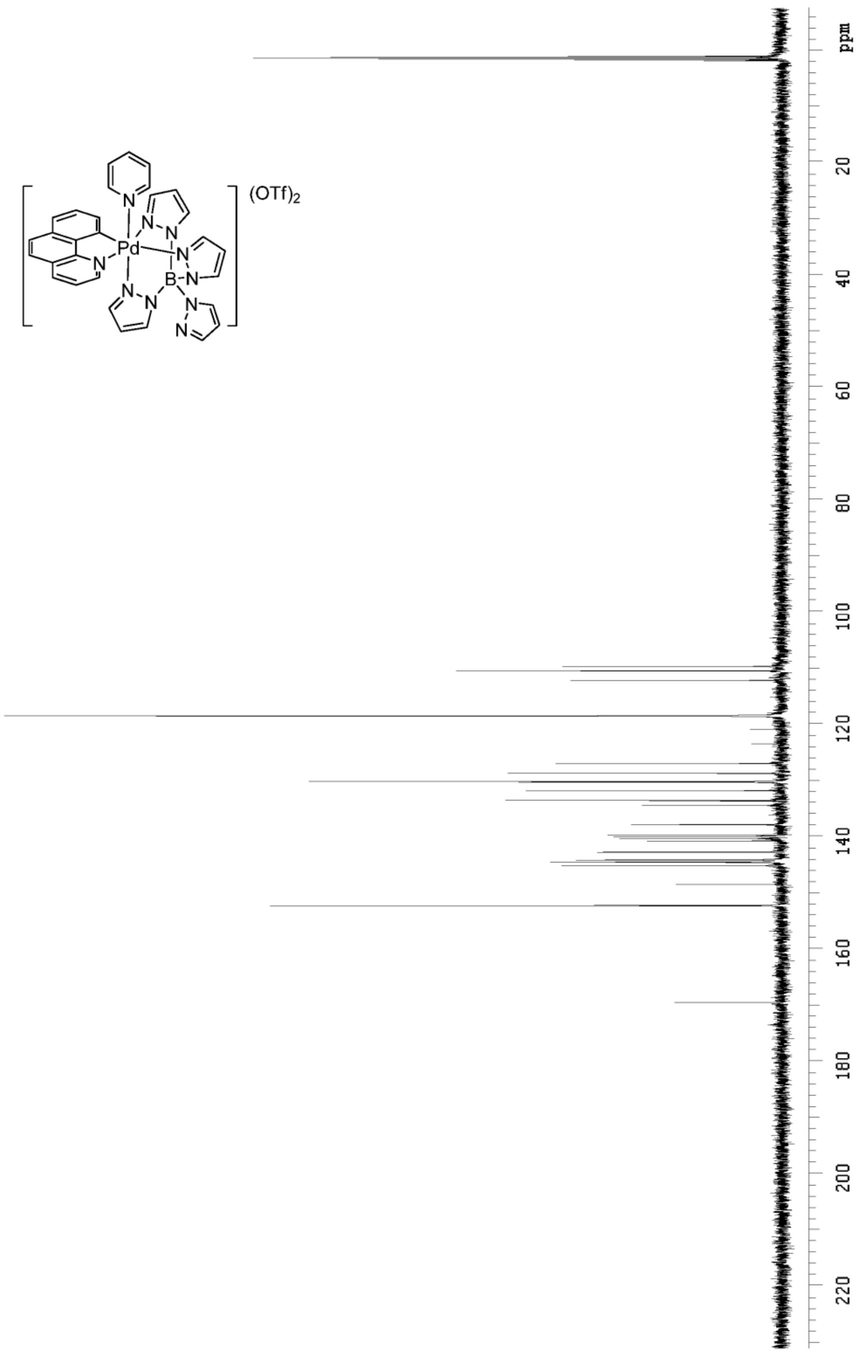

^{13}C NMR ($CDCl_3$, 23 ºC) of **S4**

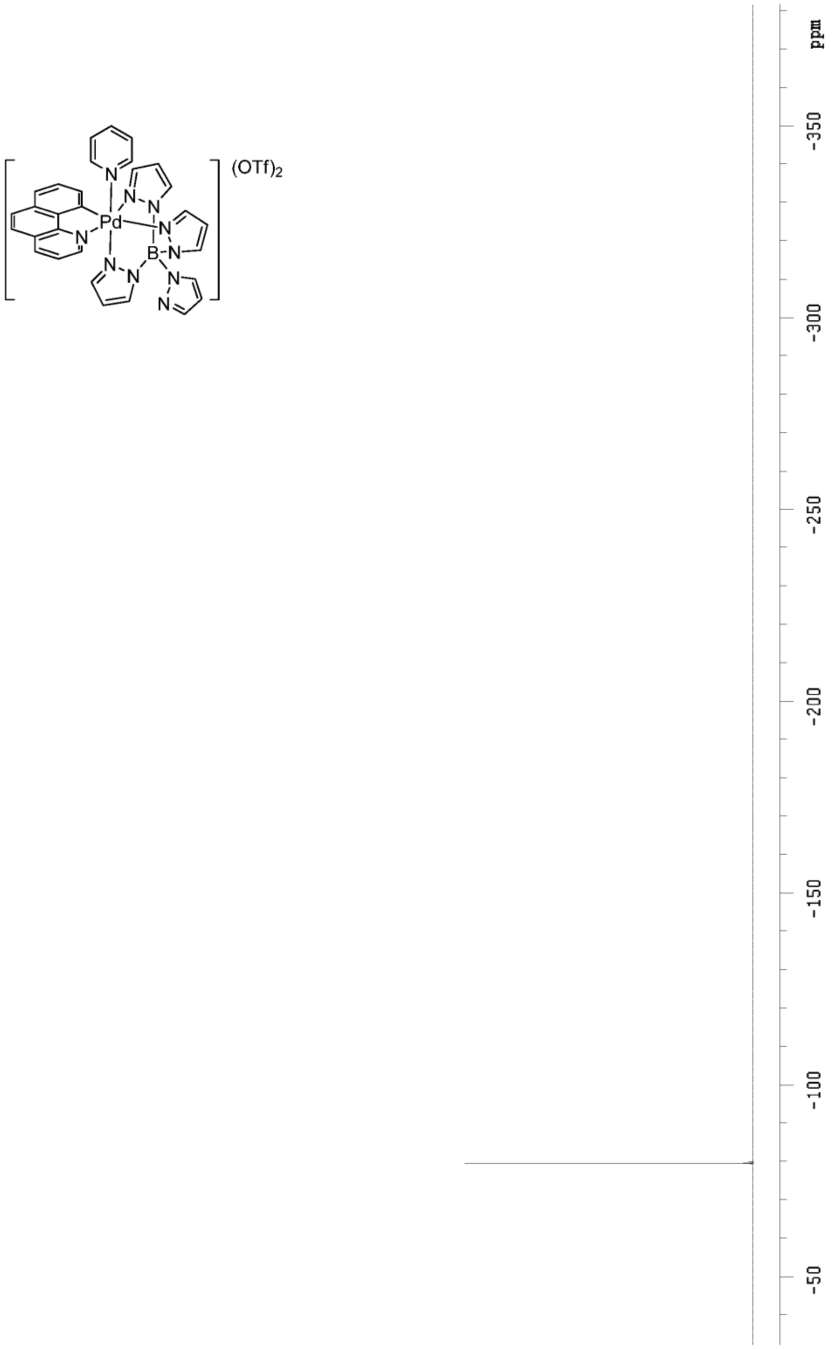

^{19}F NMR (CDCl$_3$, 23 ºC) of **S4**

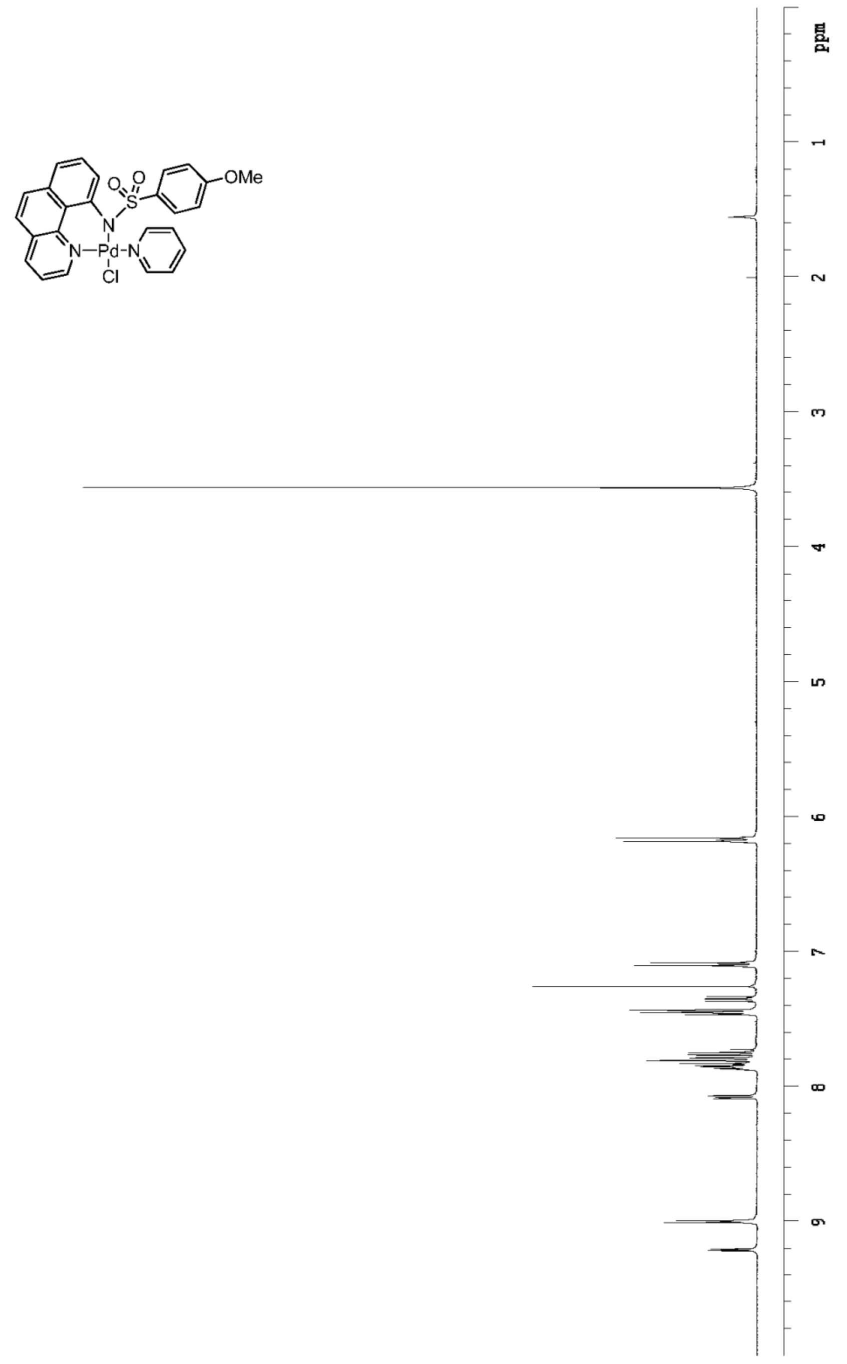

^{1}H NMR ($CDCl_3$, 23 ºC) of **S7**

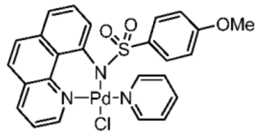

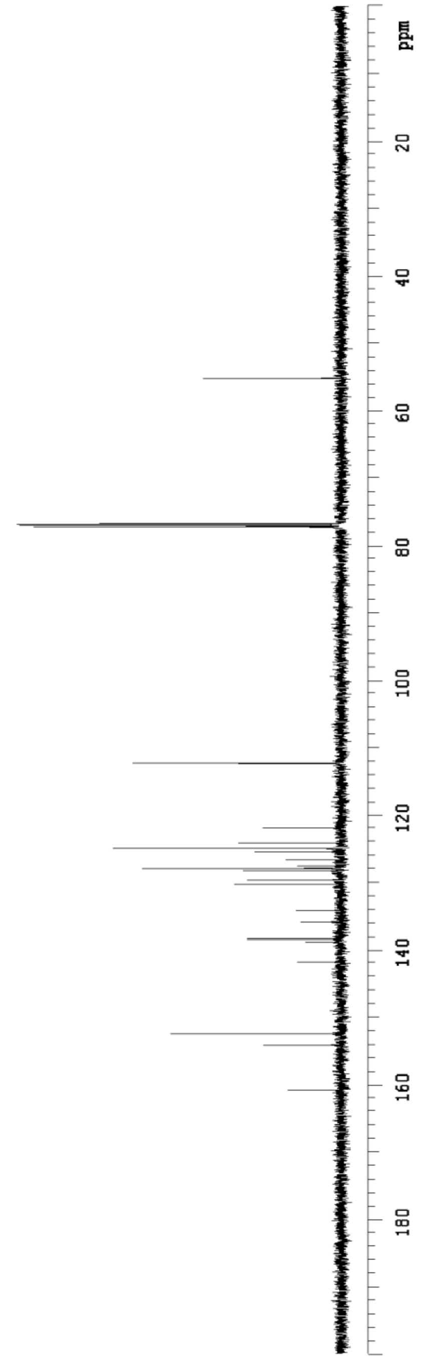

^{13}C NMR (CDCl$_3$, 23 ºC) of **S7**

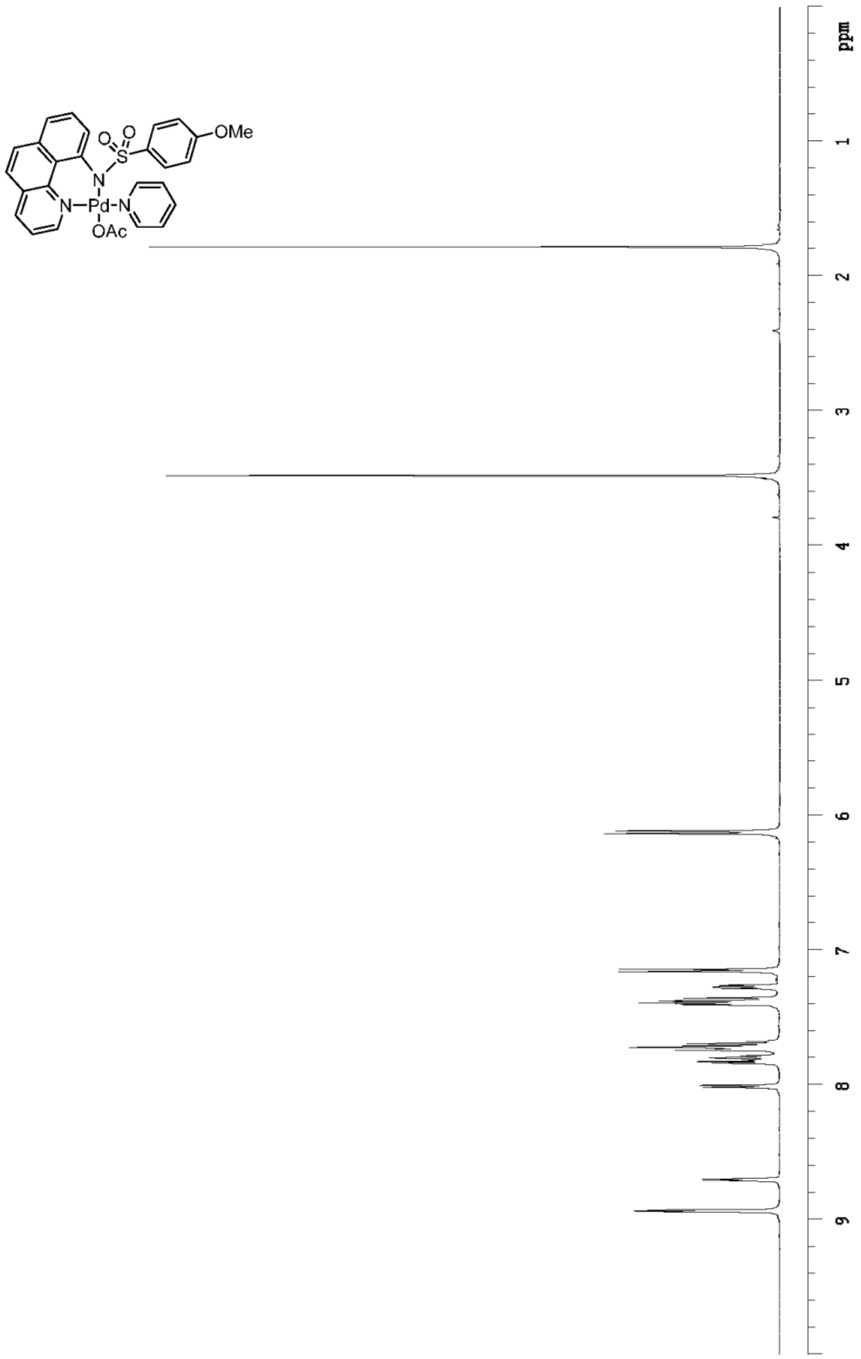

^{1}H NMR ($CDCl_3$, 23 ºC) of **8**

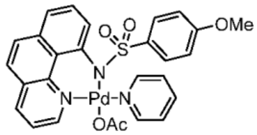

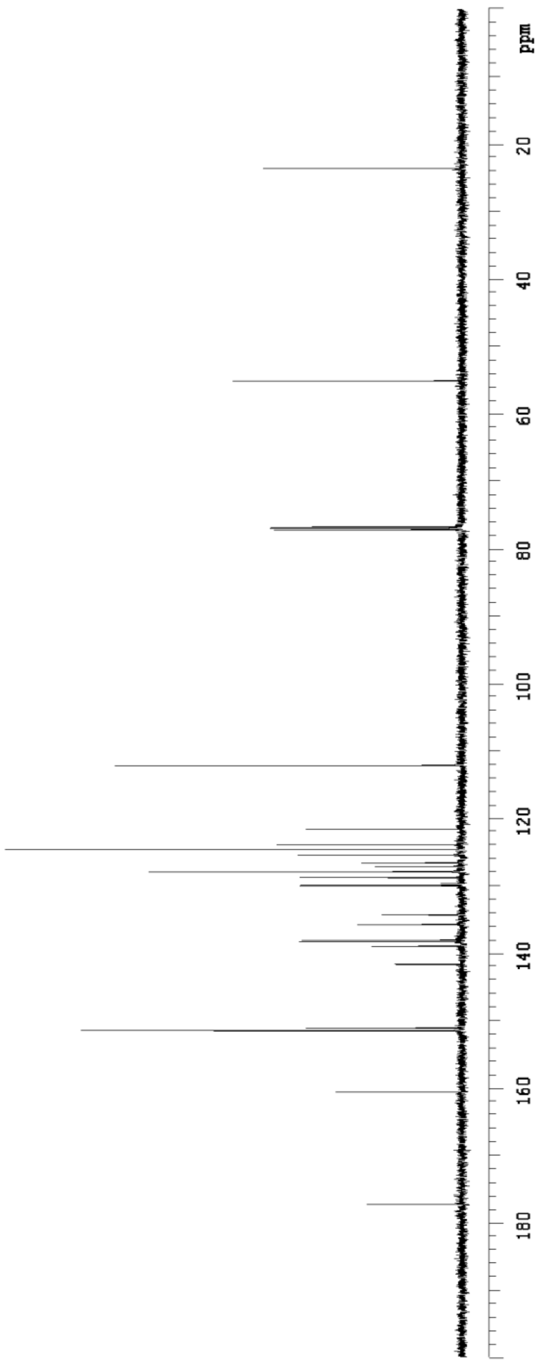

^{13}C NMR ($CDCl_3$, 23 ºC) of **8**

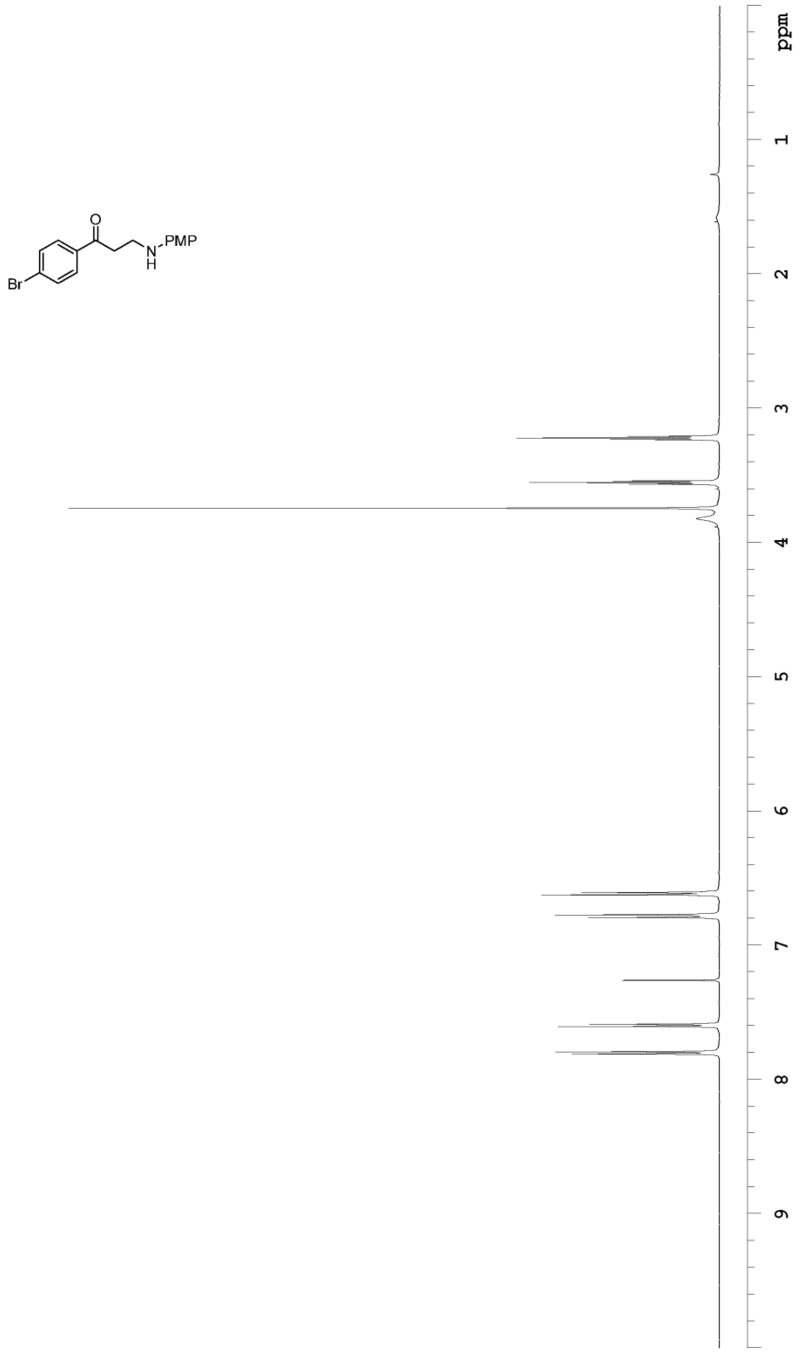

^{1}H NMR ($CDCl_3$, 23 ºC) of **S8**

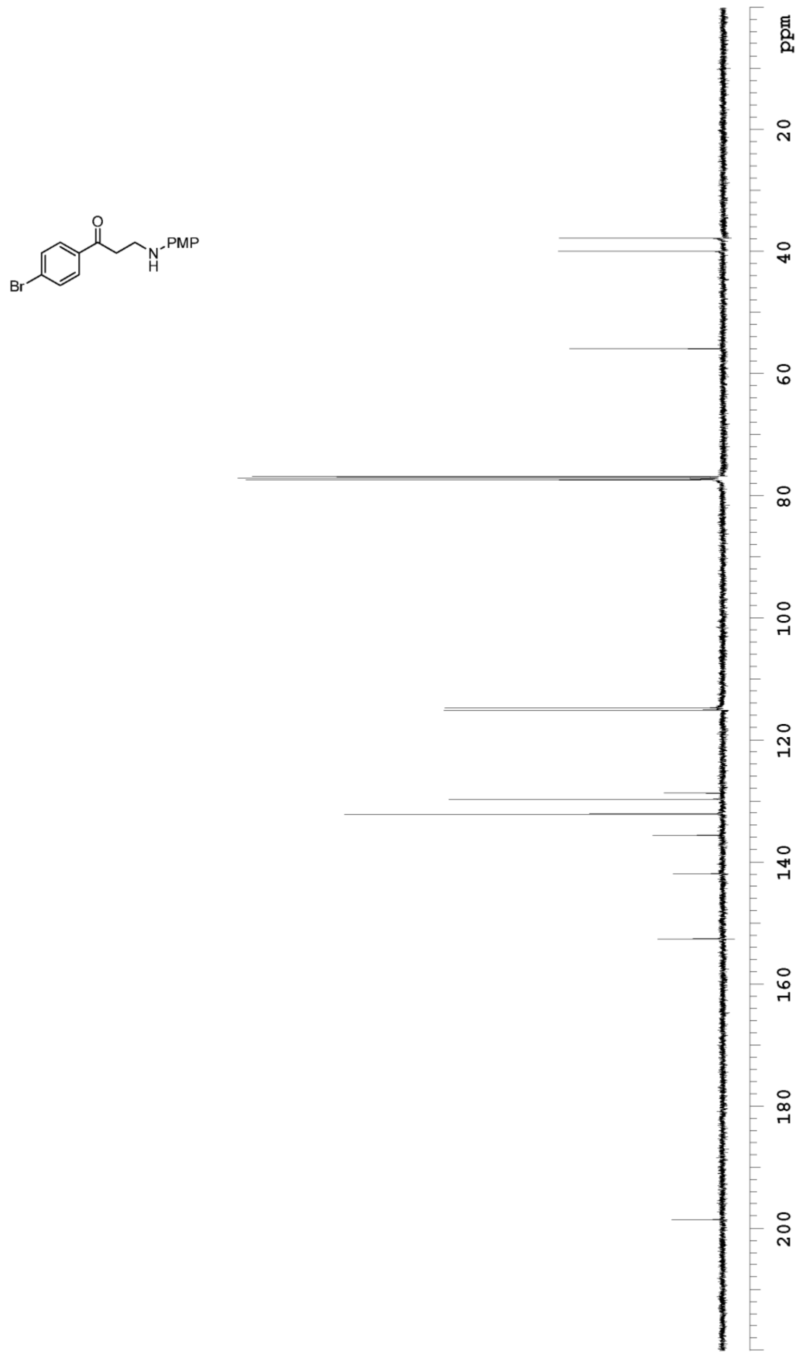

^{13}C NMR ($CDCl_3$, 23 ºC) of **S8**

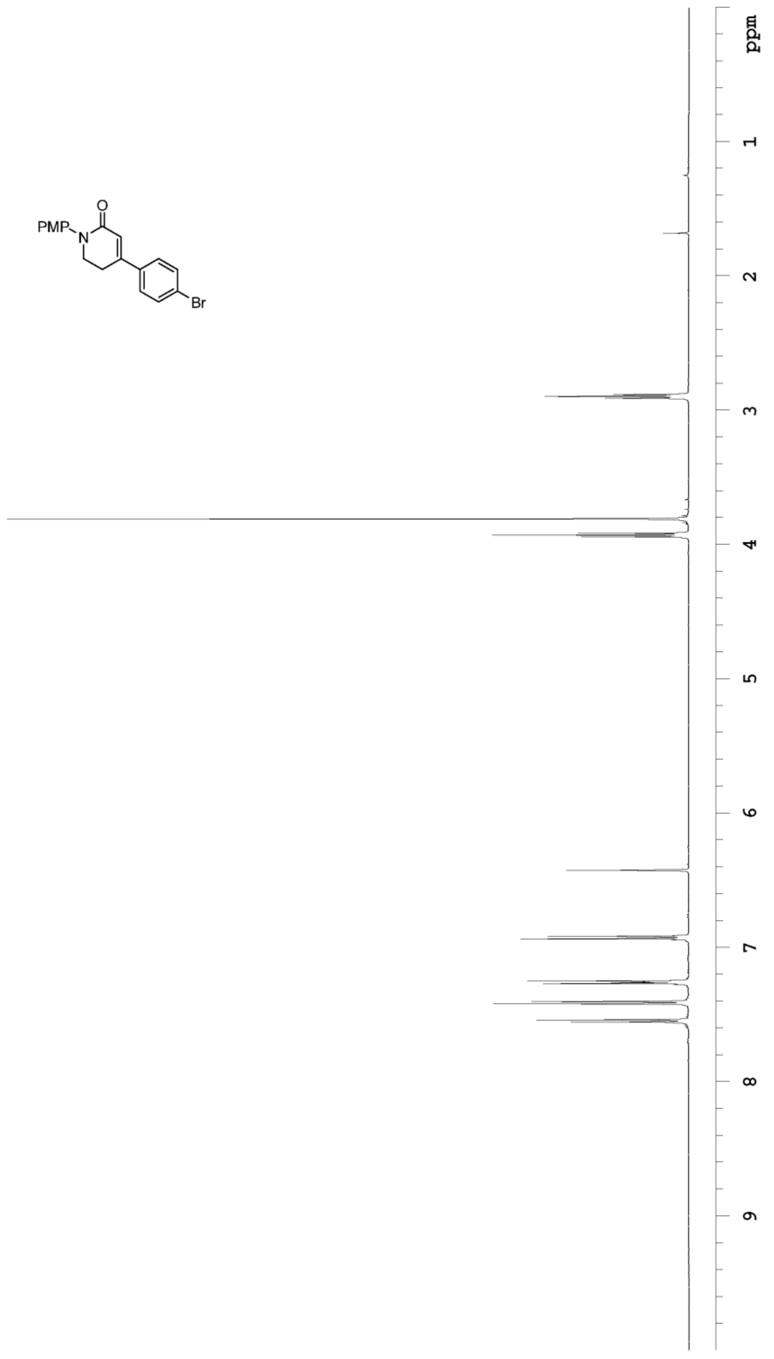

^{1}H NMR ($CDCl_3$, 23 ºC) of **10**

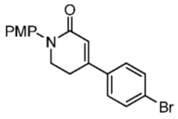

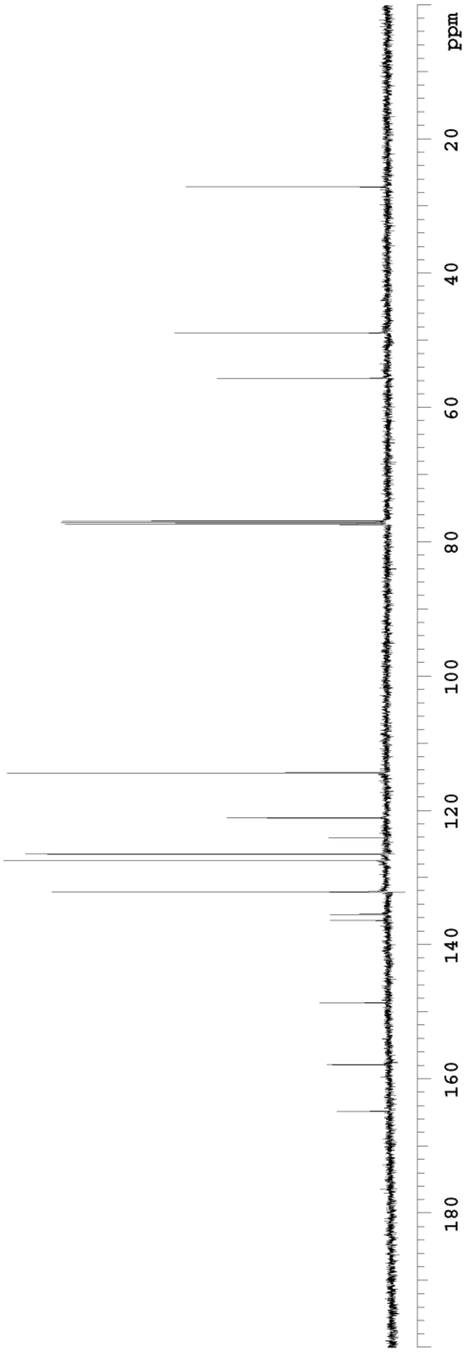

^{13}C NMR ($CDCl_3$, 23 ºC) of **10**

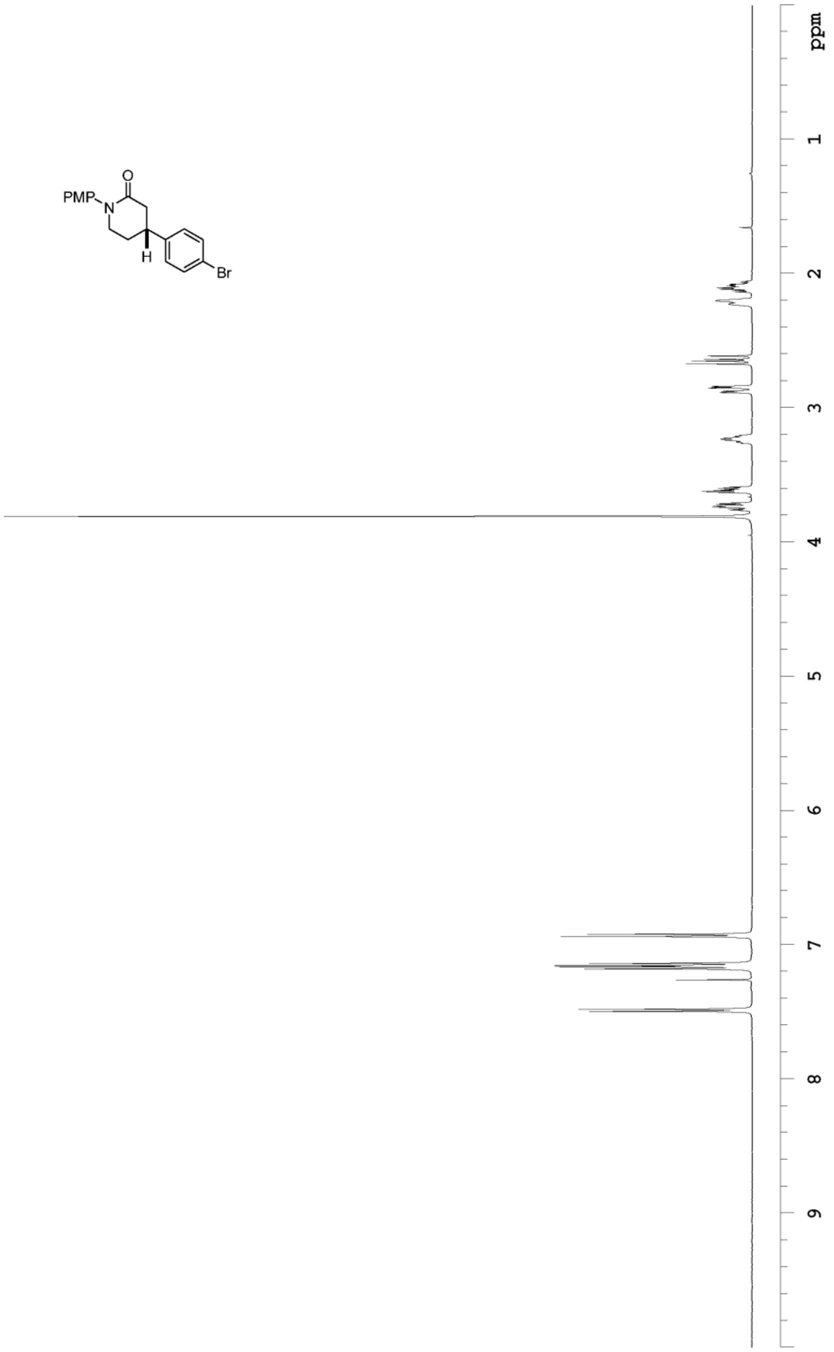

^{1}H NMR ($CDCl_3$, 23 ºC) of **11**

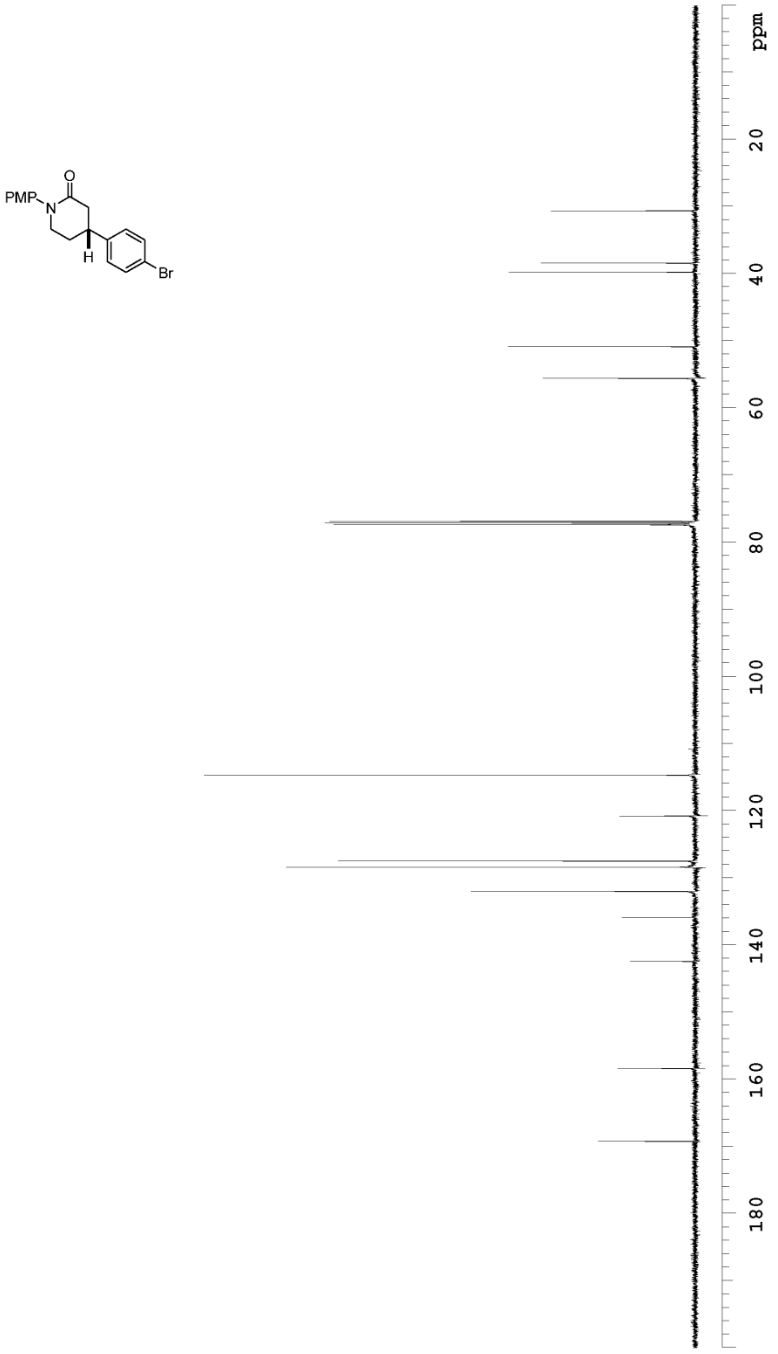

^{13}C NMR ($CDCl_3$, 23 ºC) of **11**

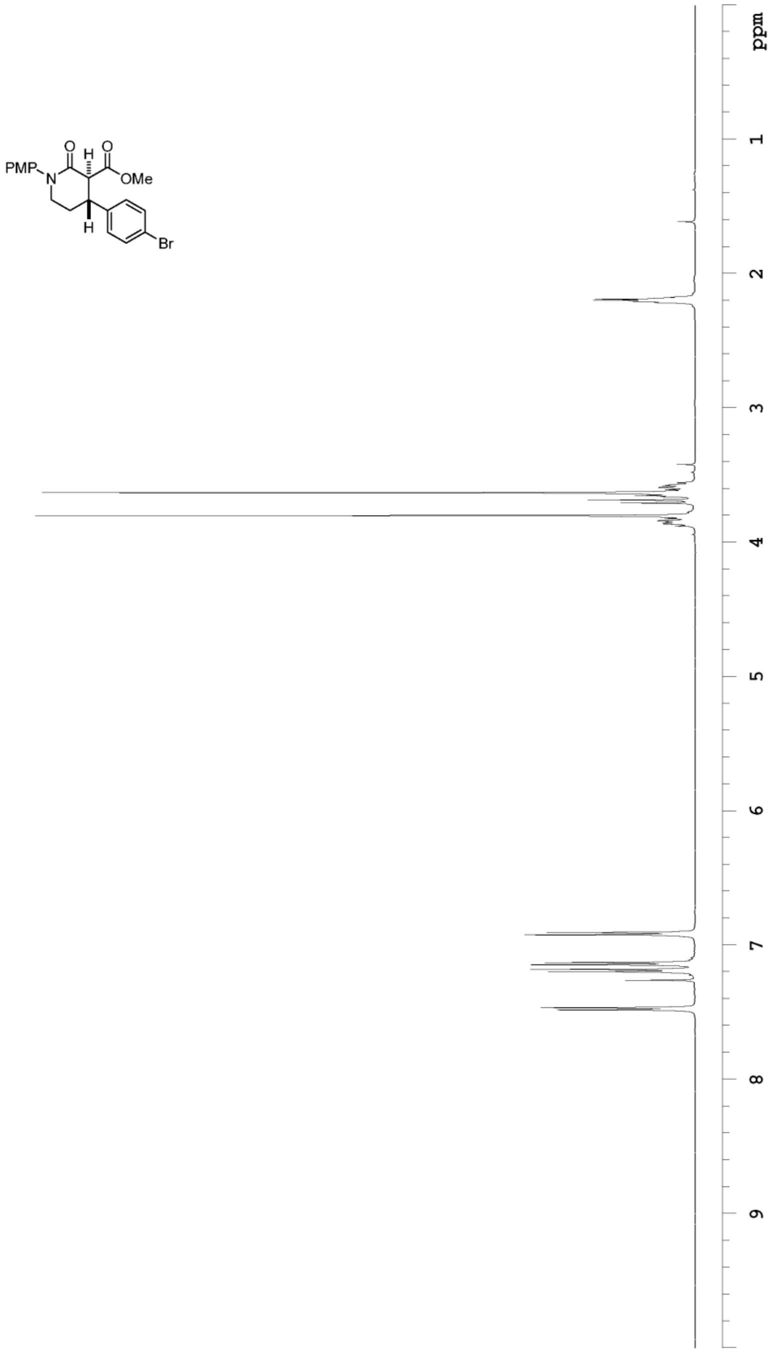

^{1}H NMR ($CDCl_3$, 23 ºC) of **S9**

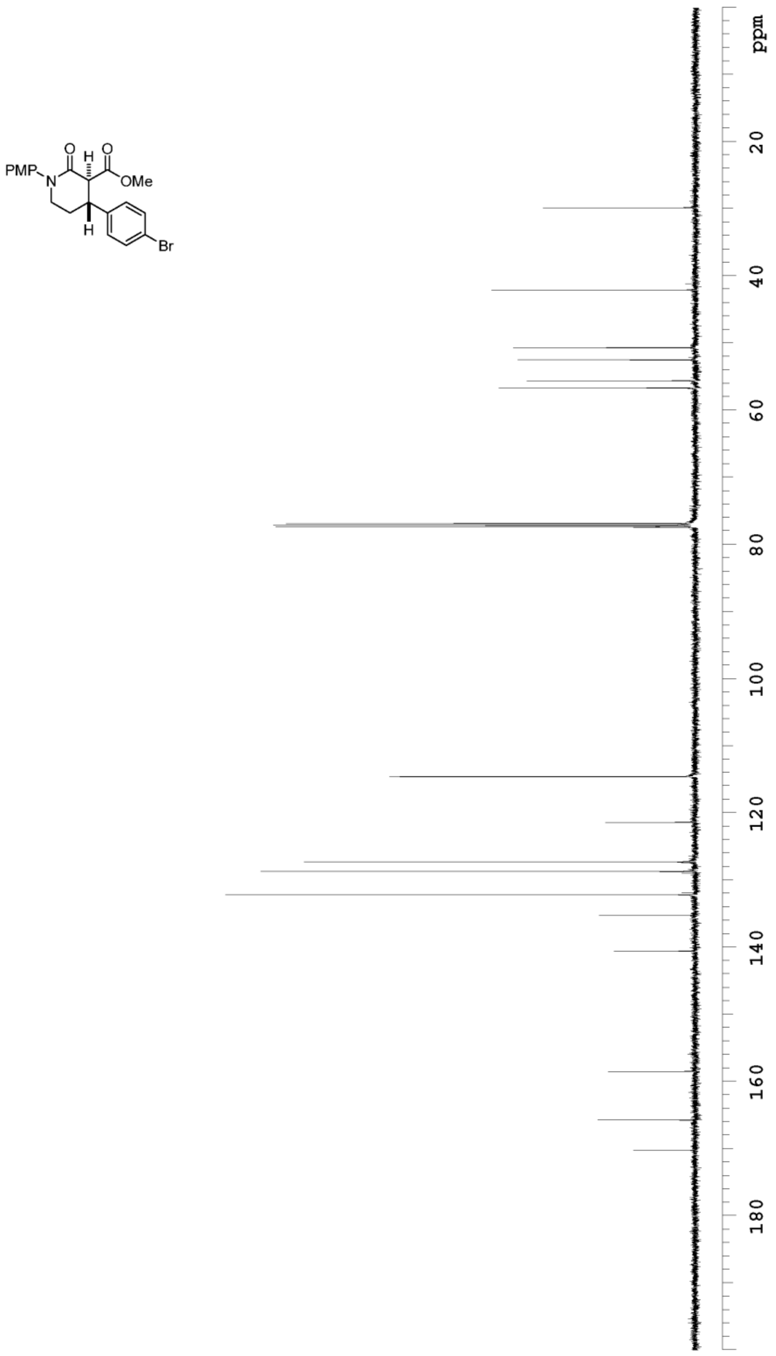

^{13}C NMR ($CDCl_3$, 23 ºC) of **S9**

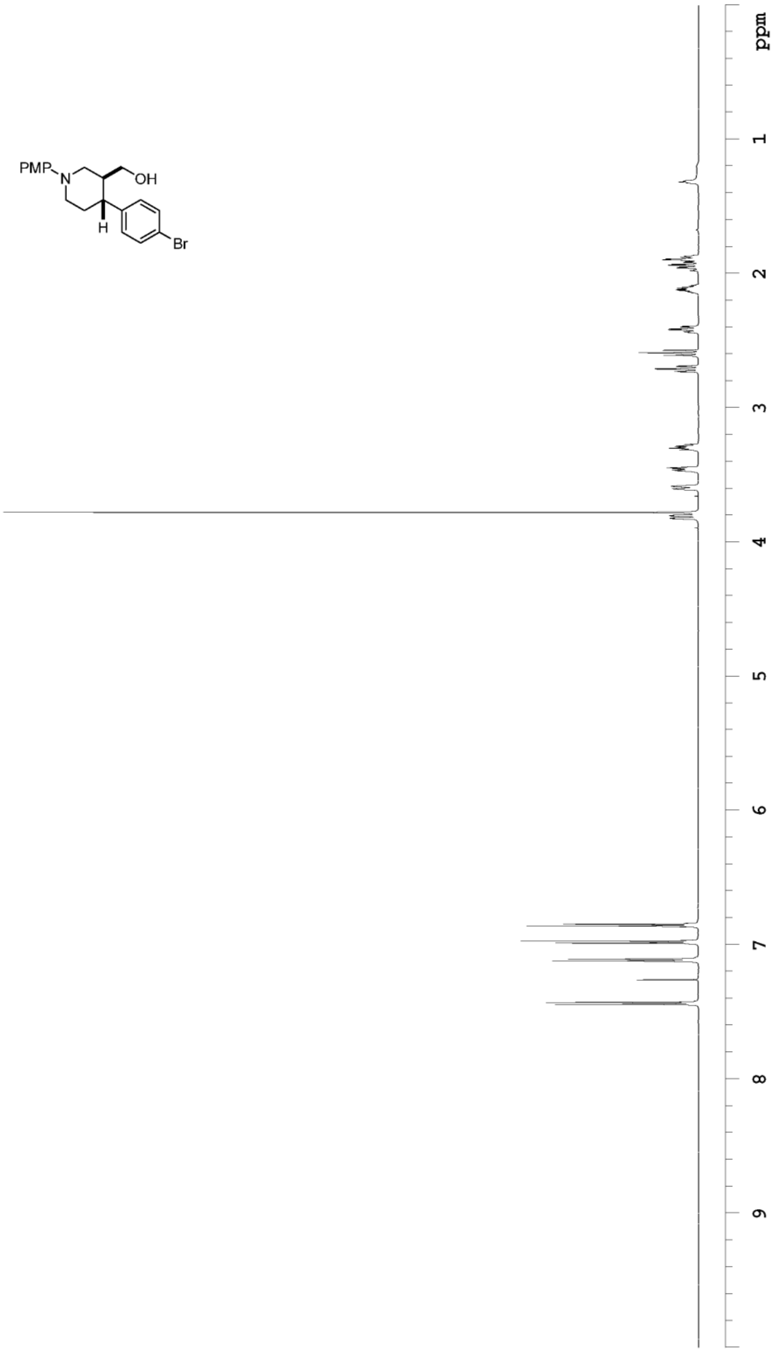

^{1}H NMR ($CDCl_3$, 23 ºC) of **12**

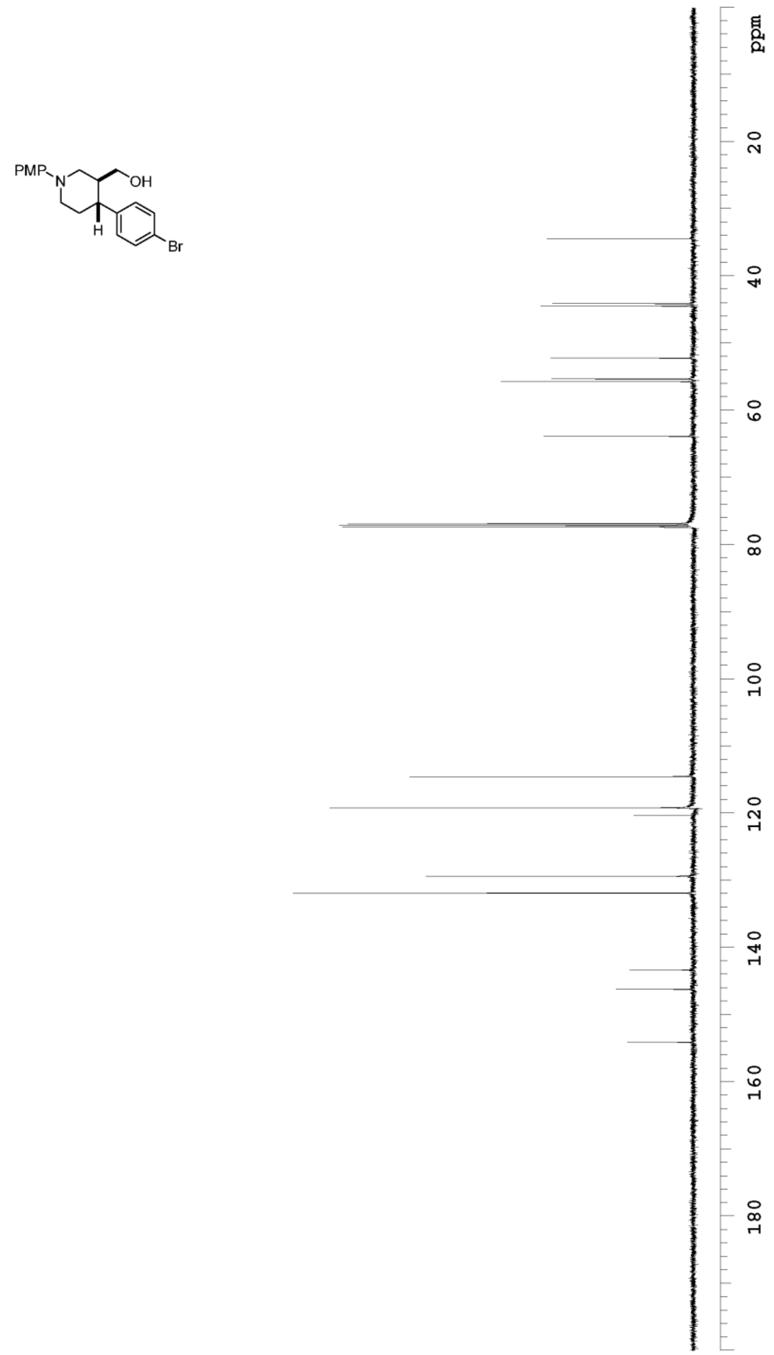

^{13}C NMR ($CDCl_3$, 23 ºC) of **12**

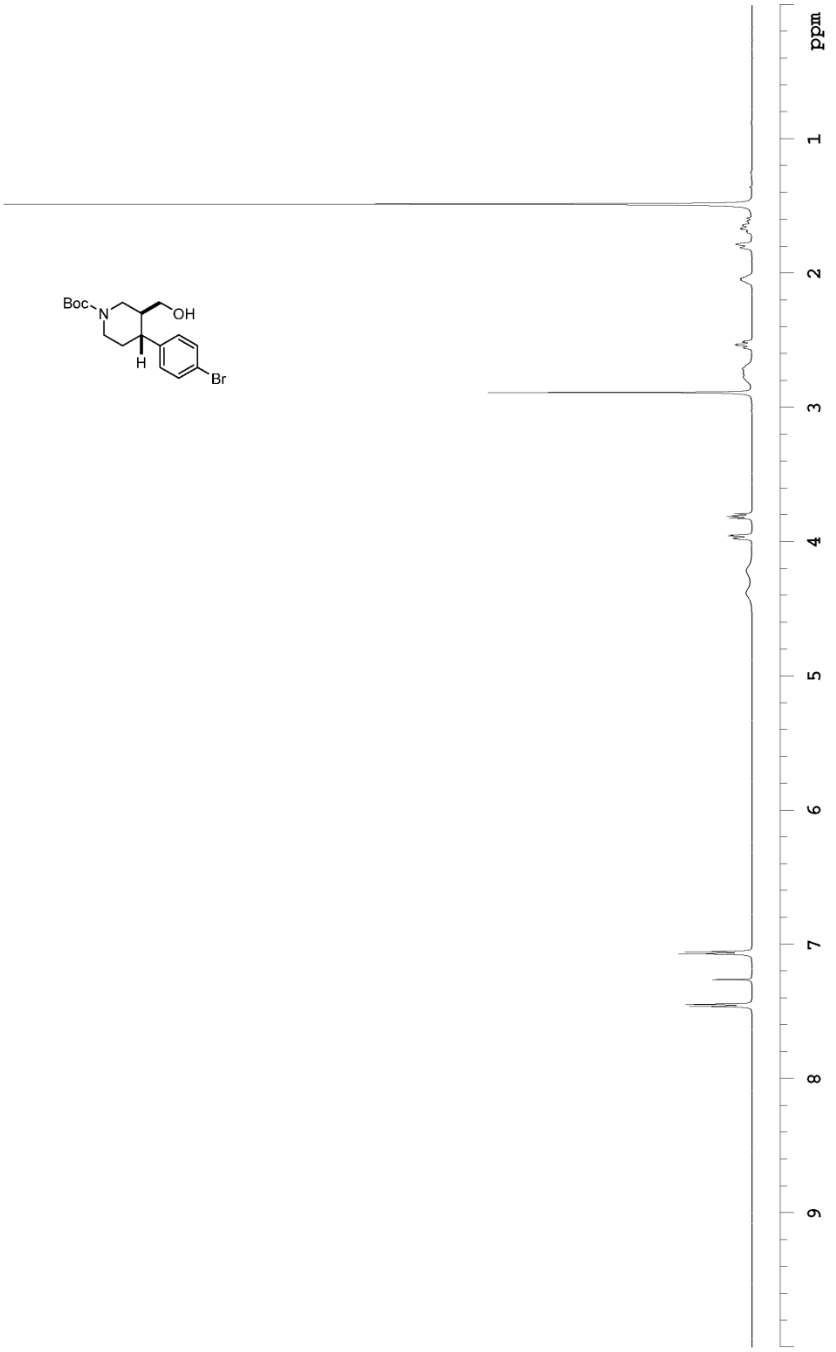

^{1}H NMR ($CDCl_3$, 23 ºC) of **S10**

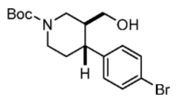

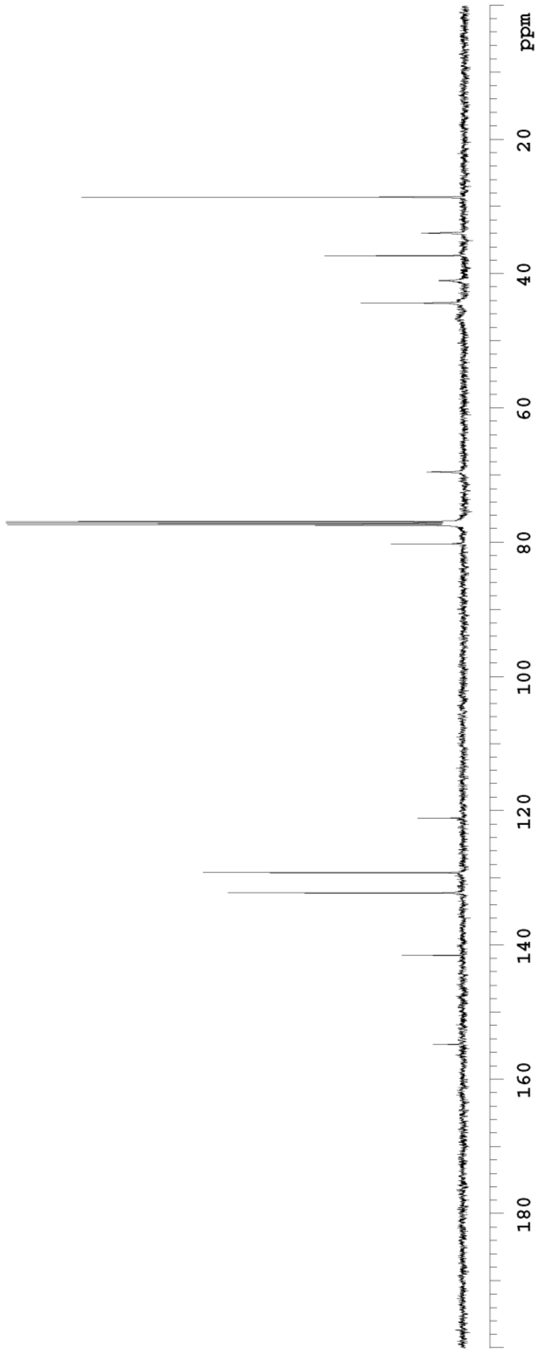

^{13}C NMR ($CDCl_3$, 23 ºC) of **S10**

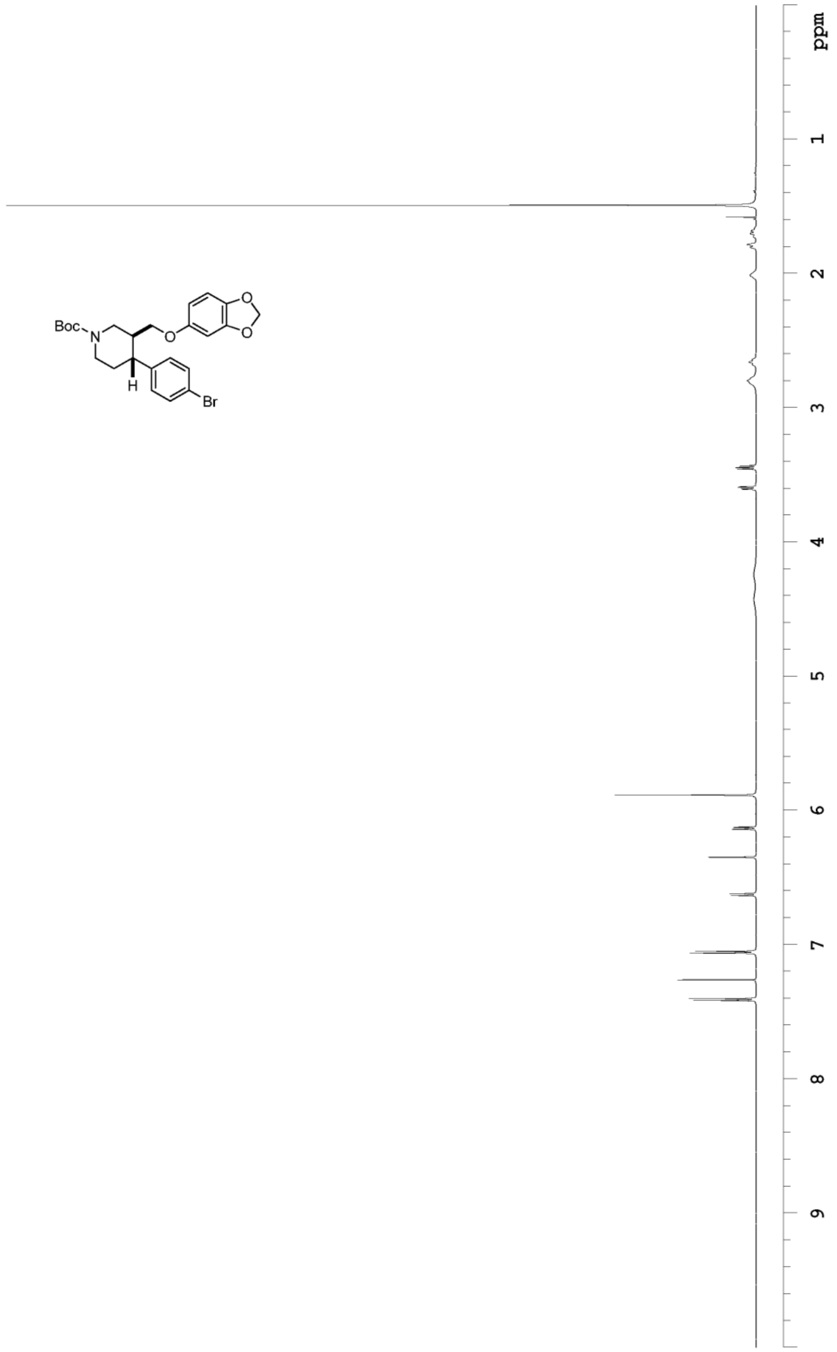

^{1}H NMR ($CDCl_3$, 23 ºC) of **13**

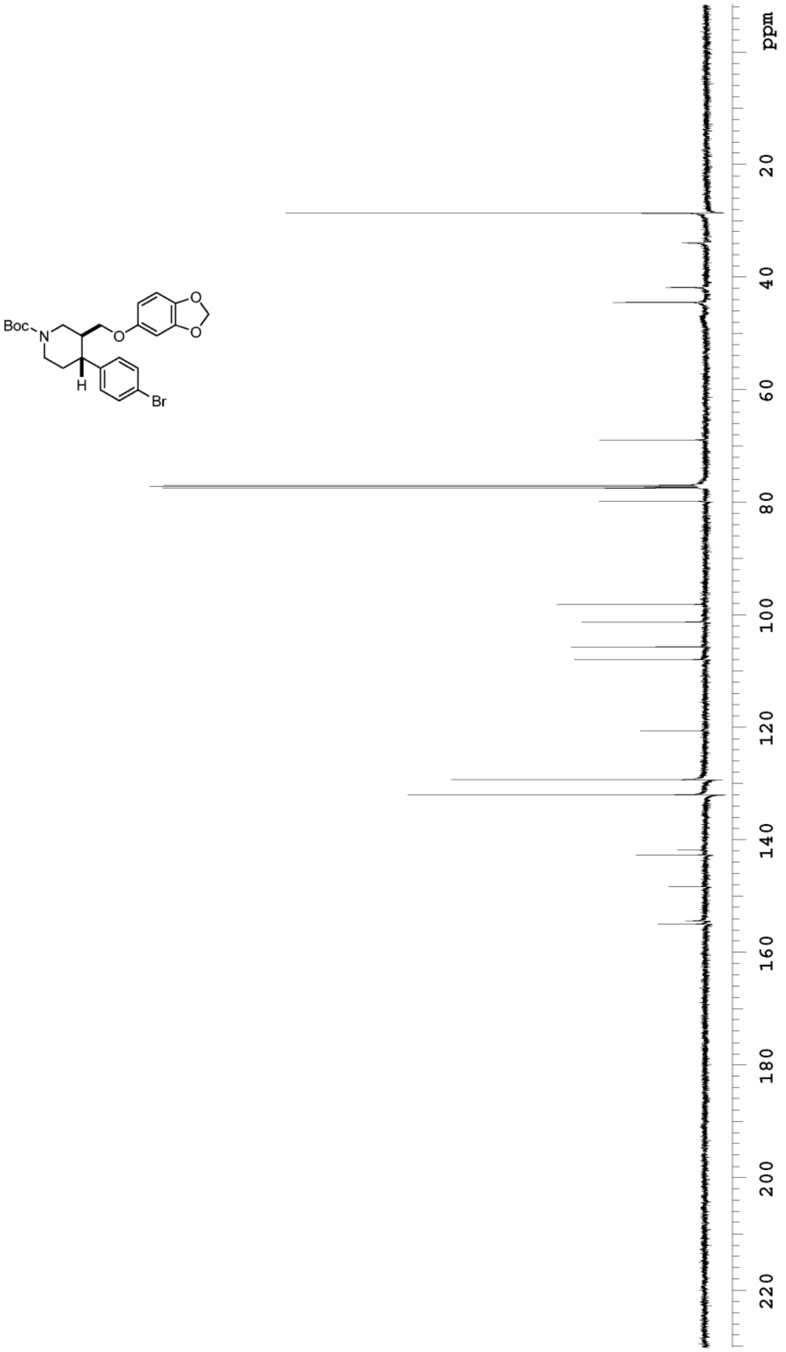

^{13}C NMR ($CDCl_3$, 23 ºC) of **13**

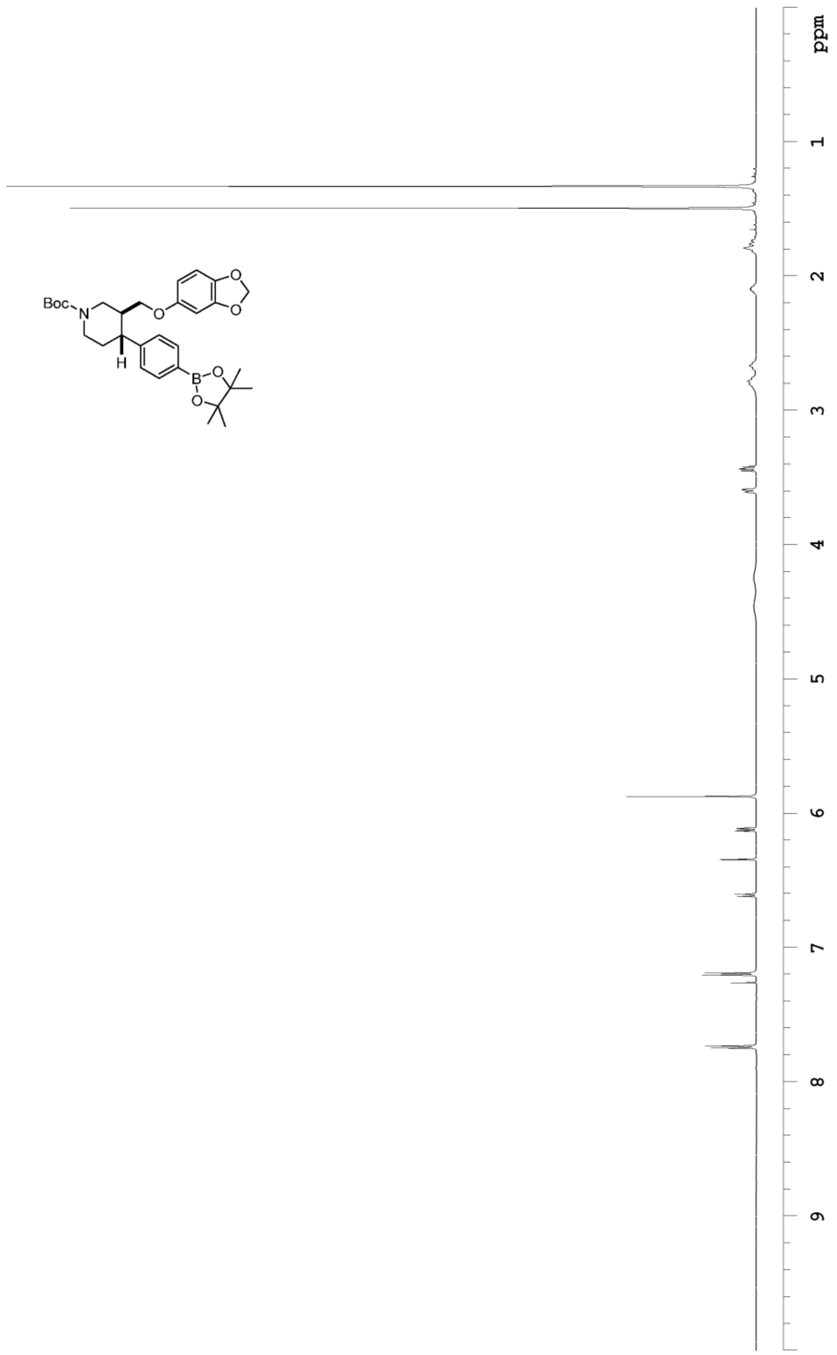

^{1}H NMR ($CDCl_3$, 23 ºC) of **14**

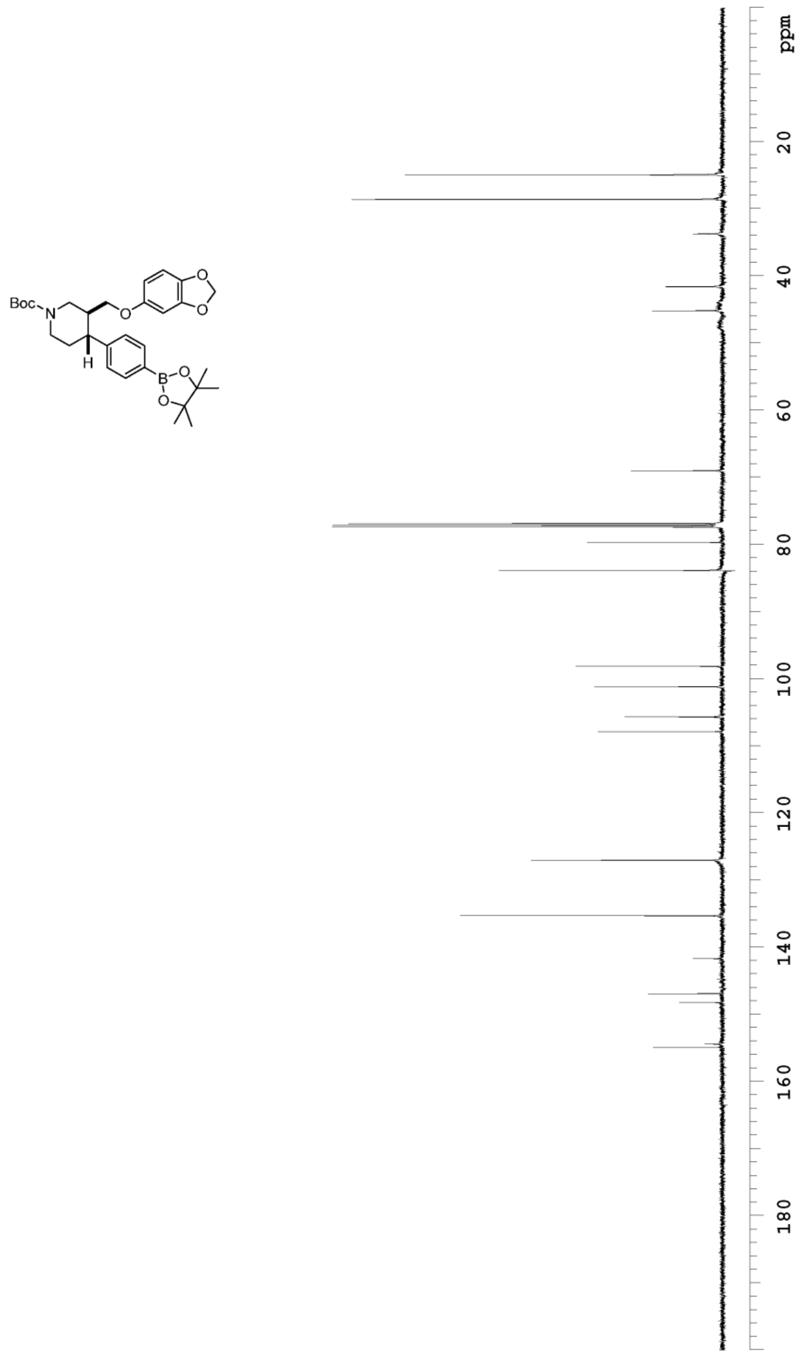

^{13}C NMR ($CDCl_3$, 23 ºC) of **14**

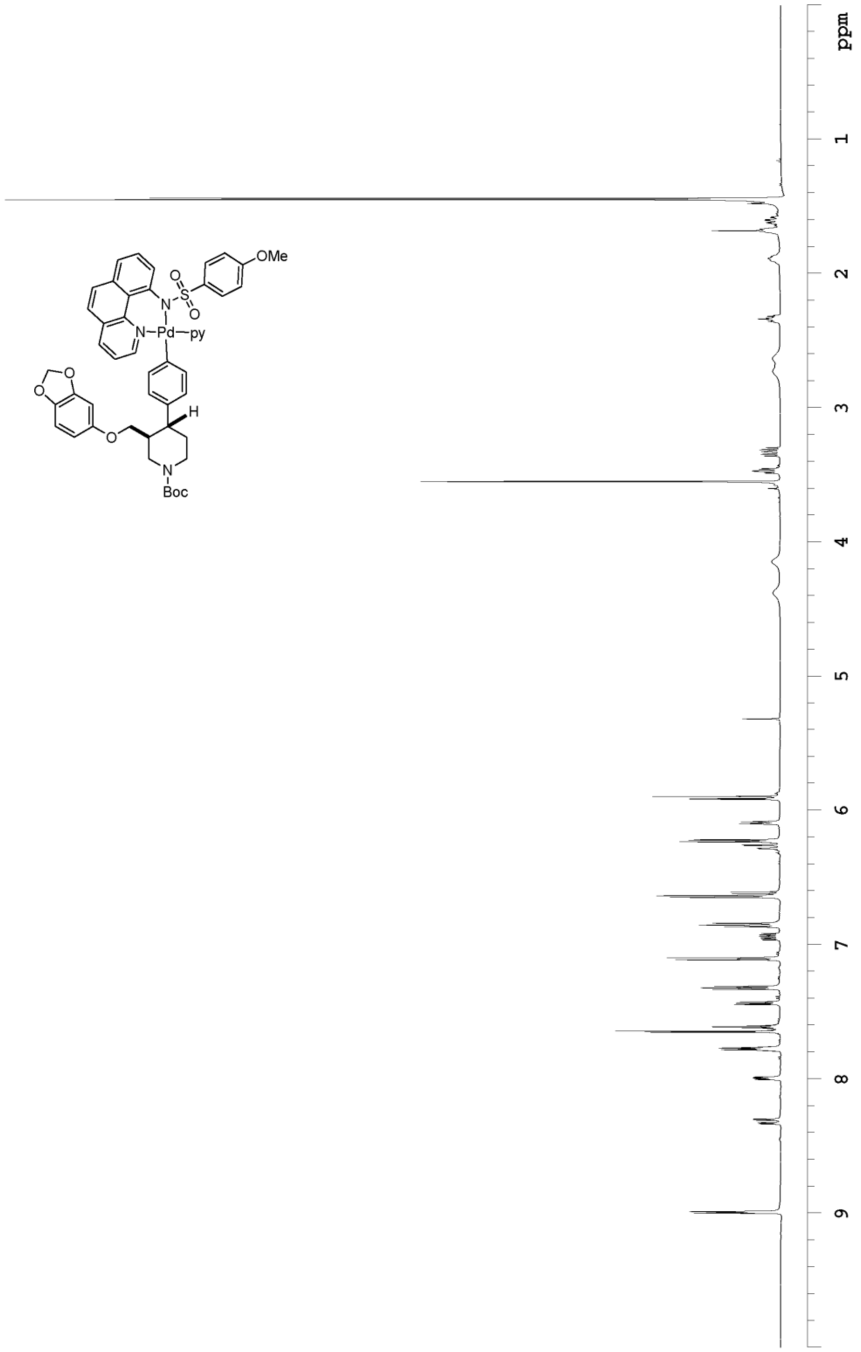

^{1}H NMR (CD_2Cl_2, 23 ºC) of **15**

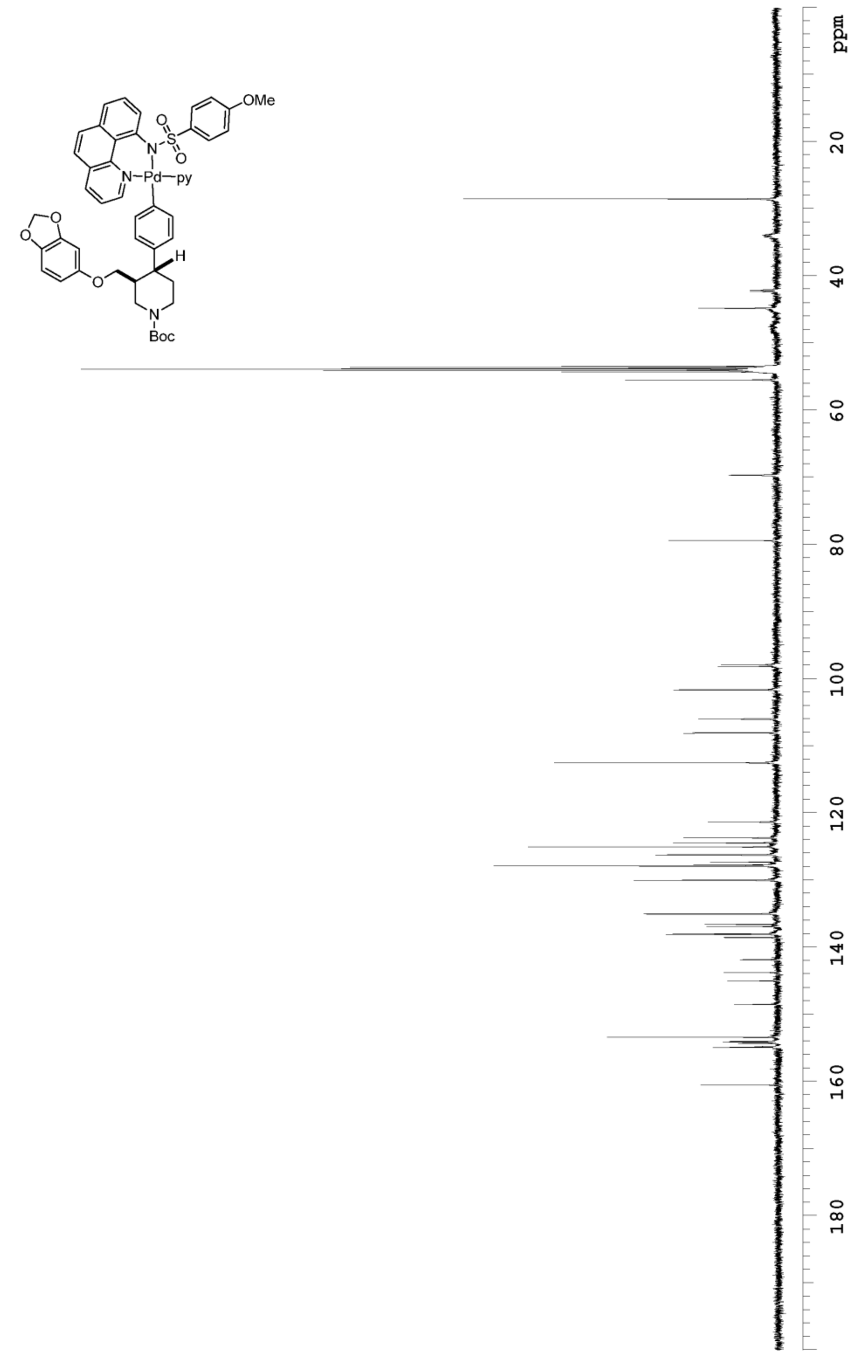

^{13}C NMR (CD_2Cl_2, 23 ºC) of **15**

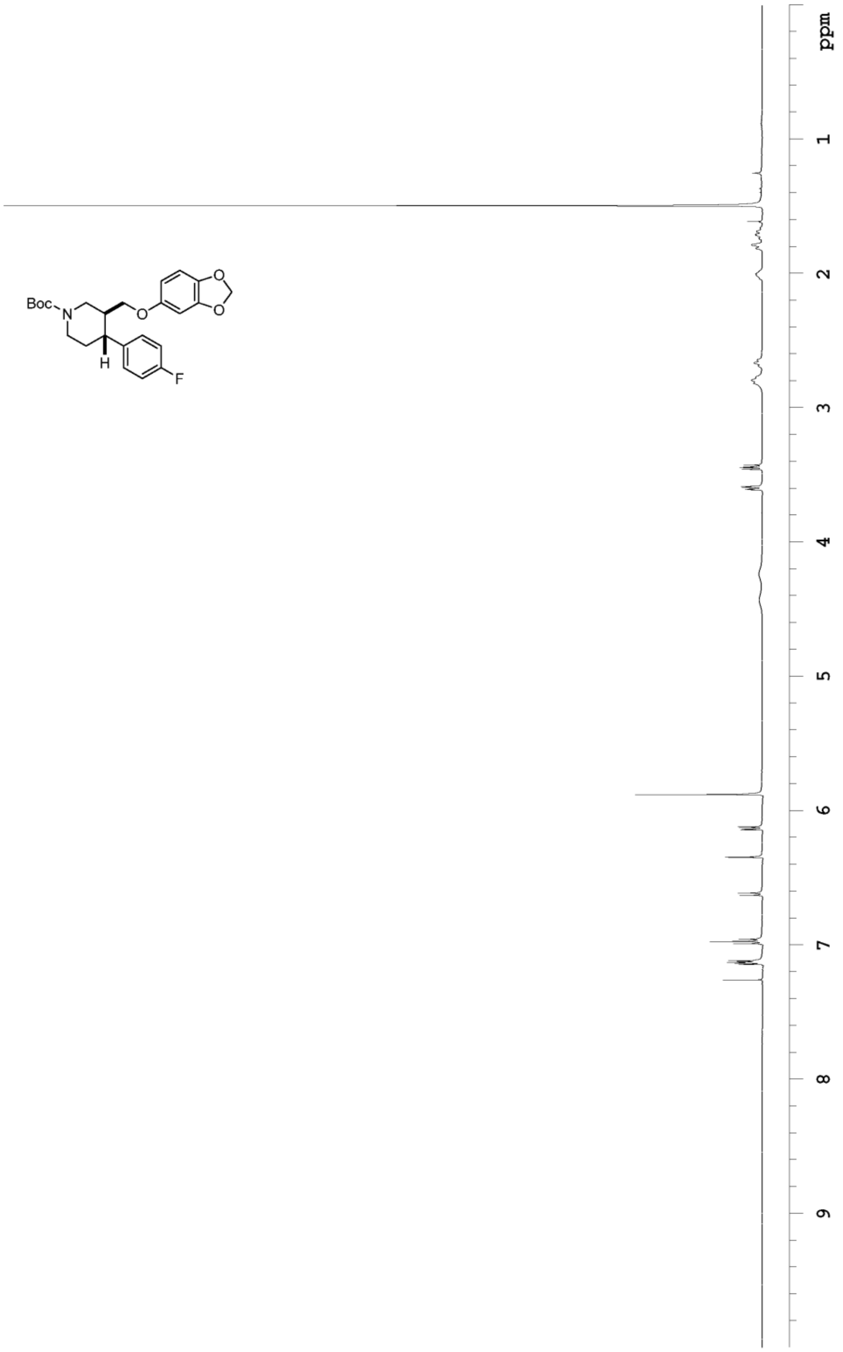

^{1}H NMR ($CDCl_3$, 23 ºC) of **21**

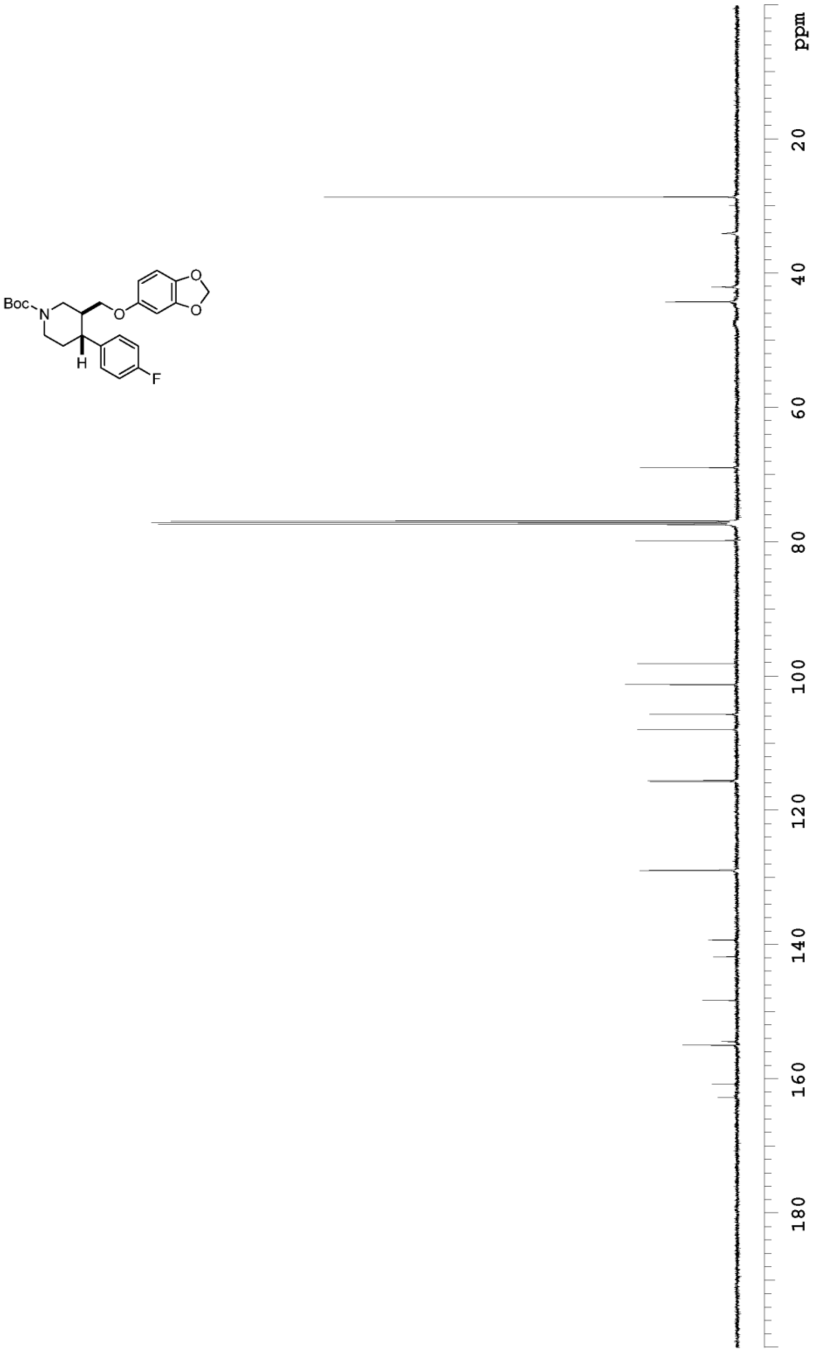

^{13}C NMR ($CDCl_3$, 23 ºC) of **21**

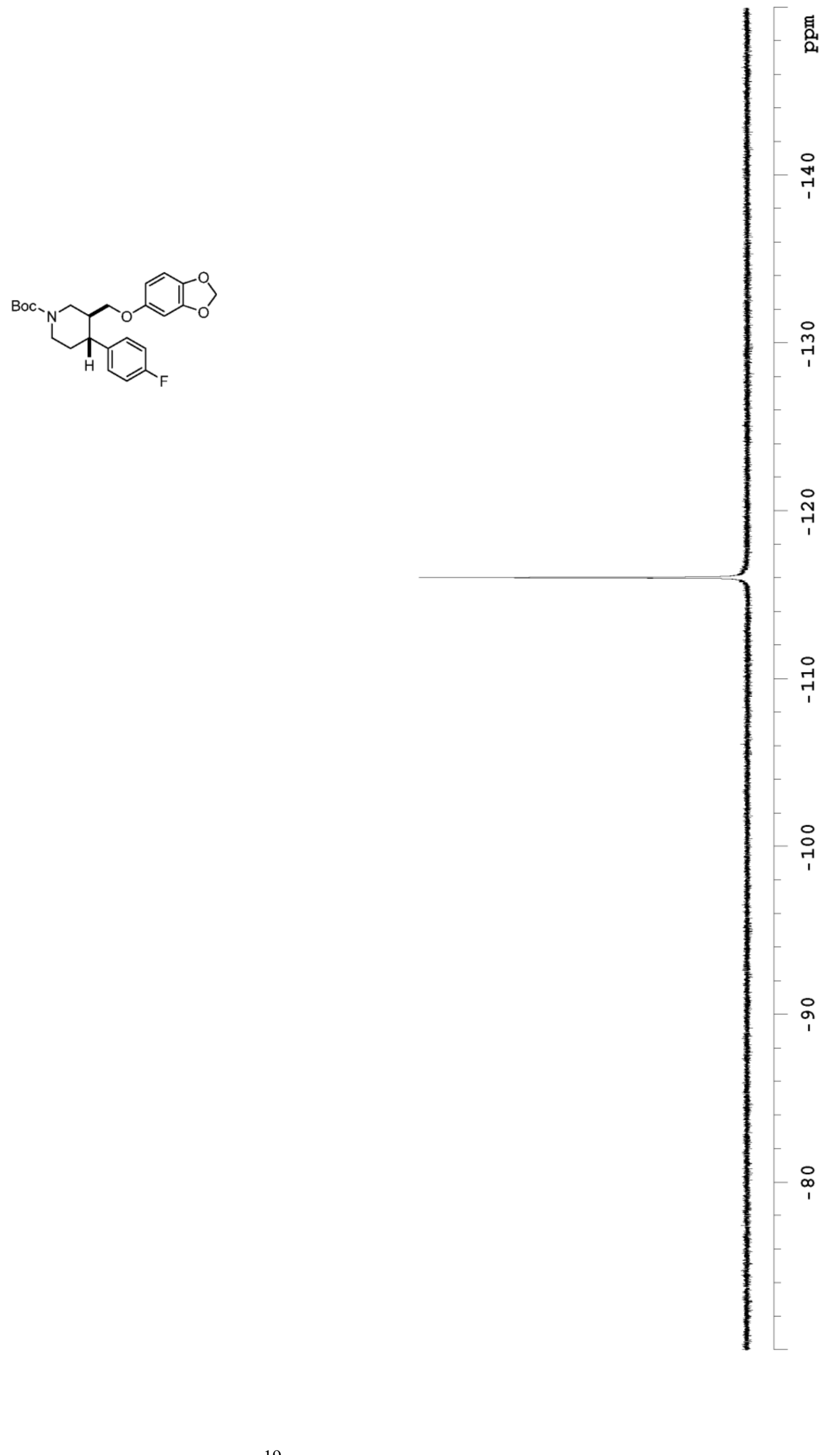

^{19}F NMR ($CDCl_3$, 23 ºC) of **21**

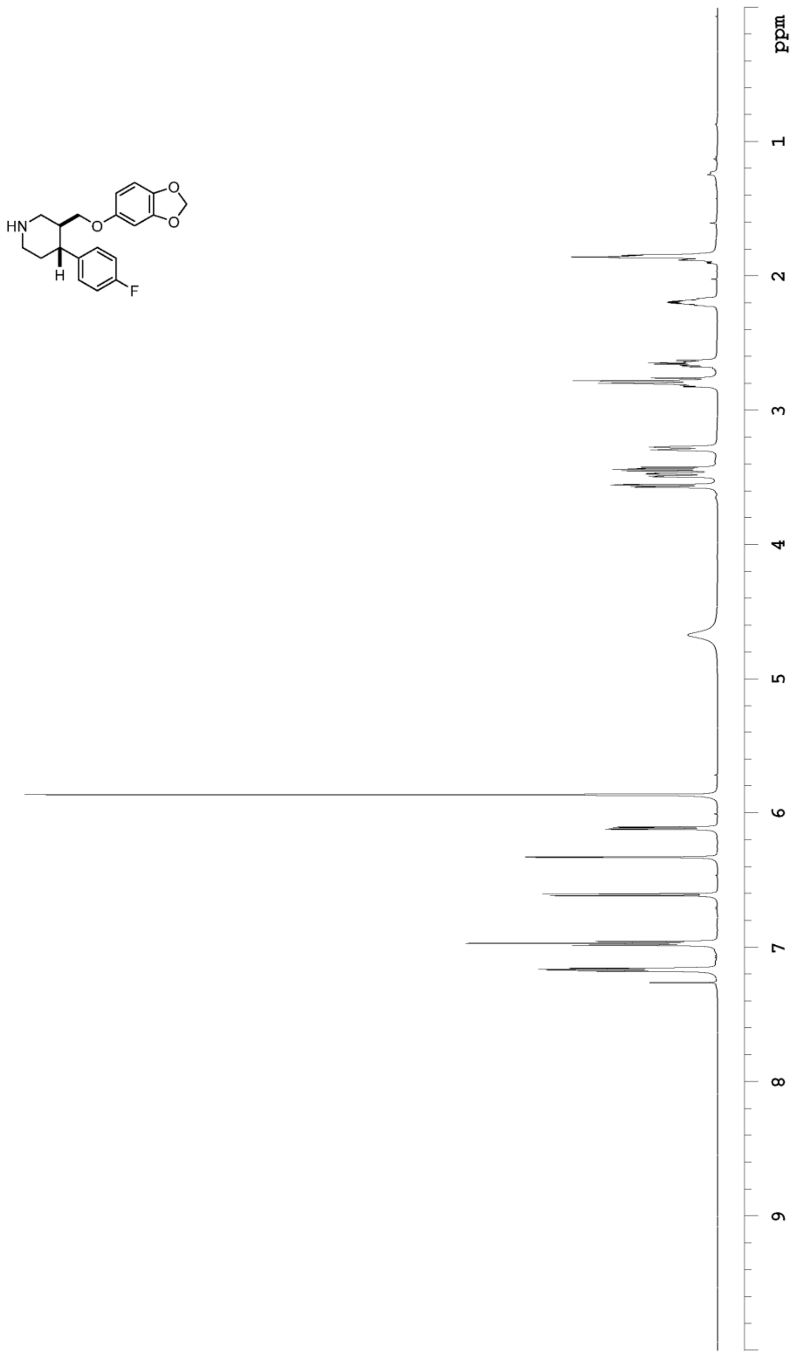

^{1}H NMR ($CDCl_3$, 23 ºC) of **1**

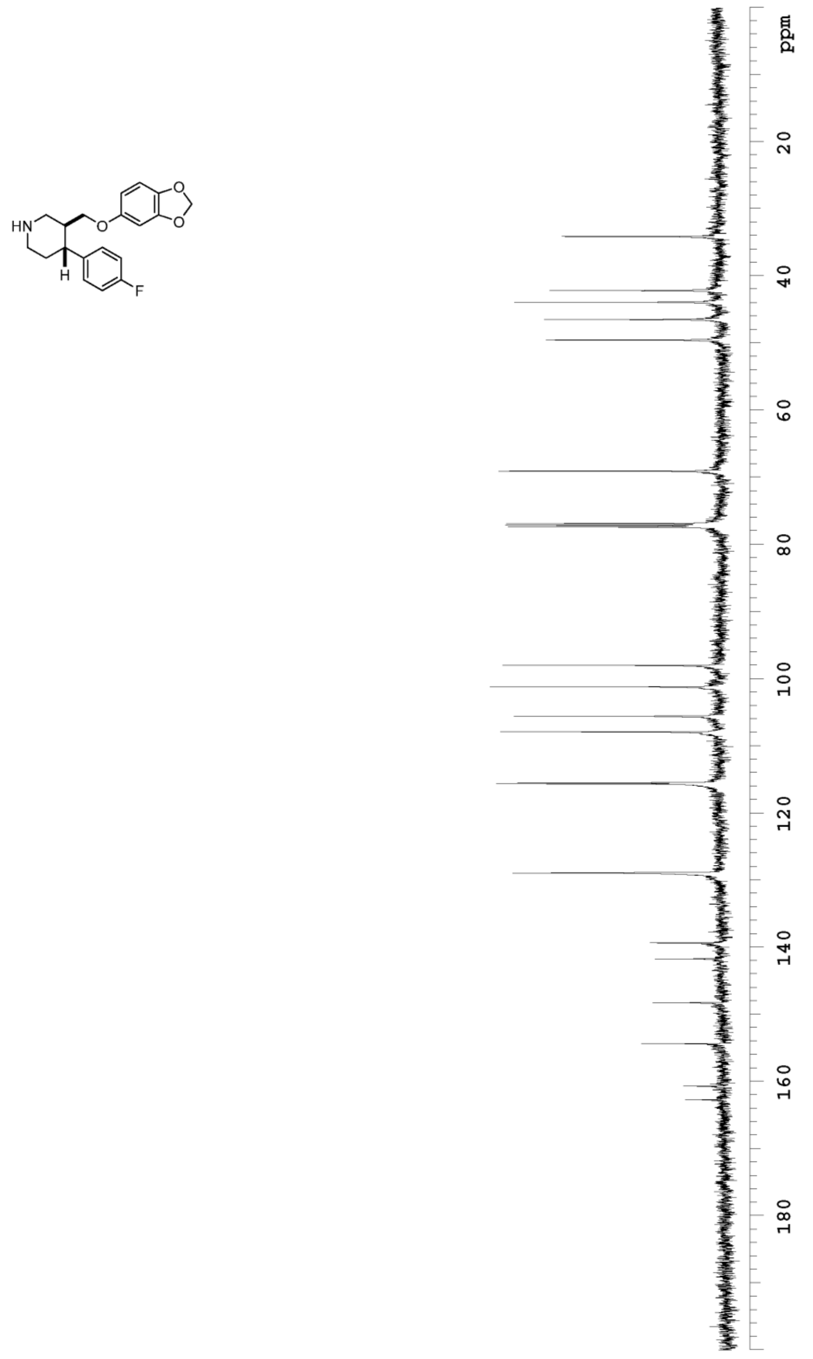

^{13}C NMR ($CDCl_3$, 23 ºC) of **1**

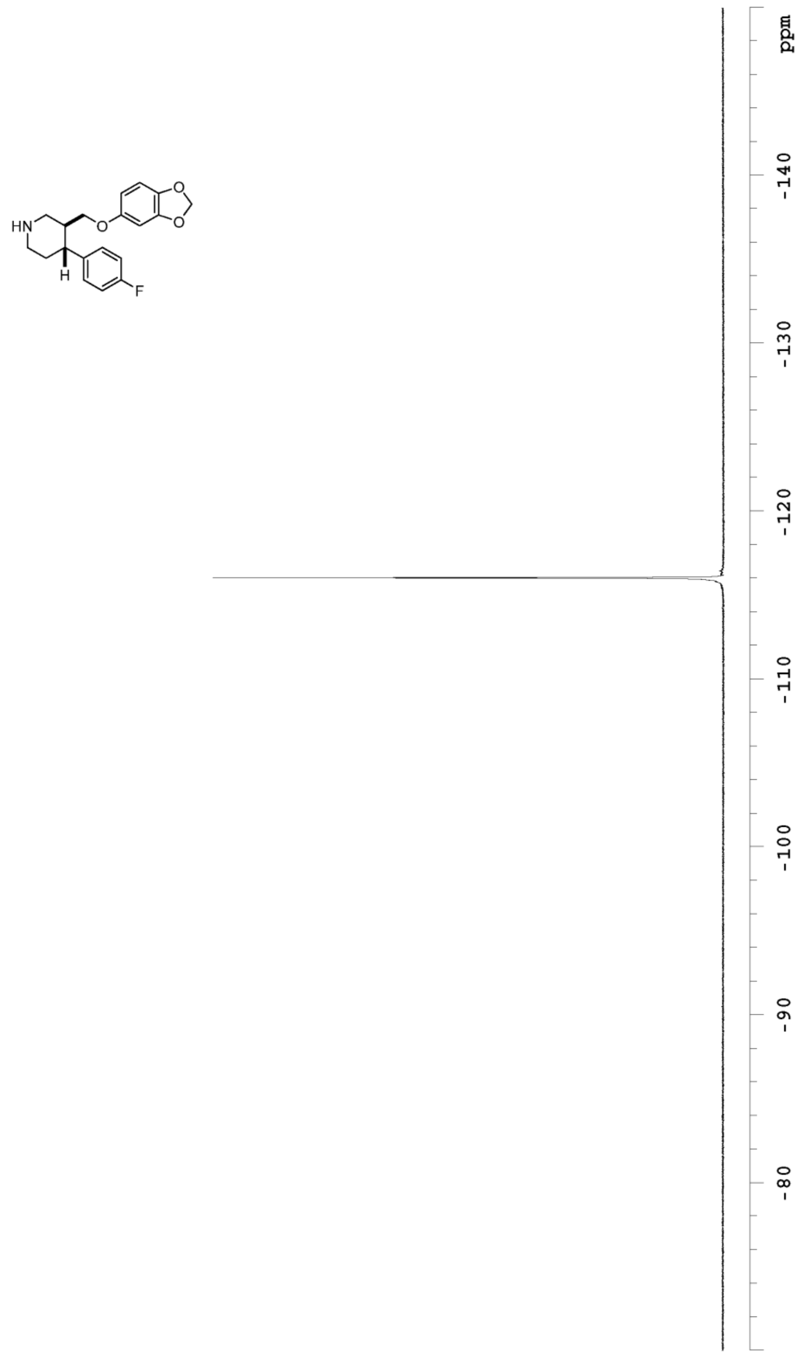

^{19}F NMR ($CDCl_3$, 23 ºC) of **1**

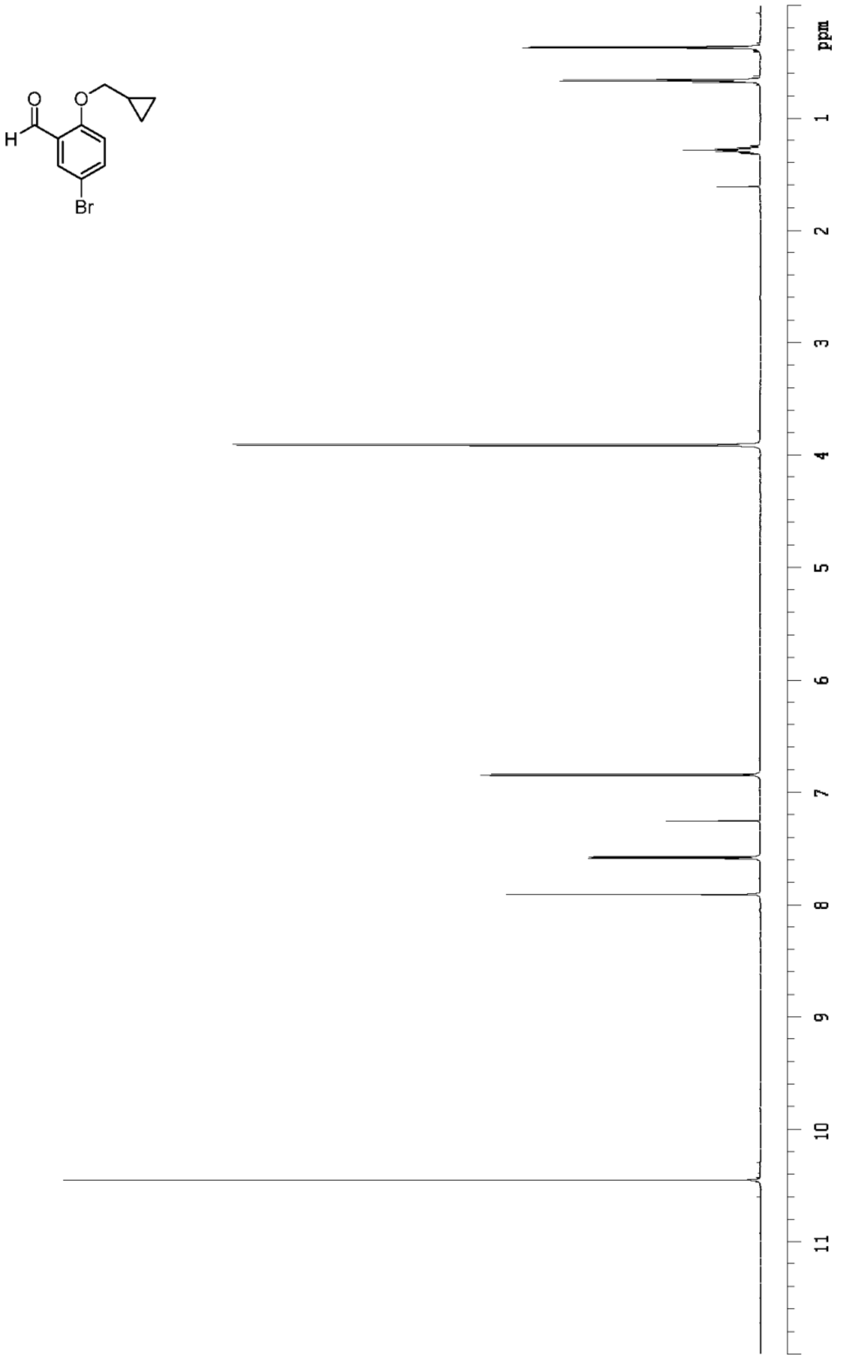

^{1}H NMR ($CDCl_3$, 23 ºC) of **S11**

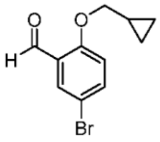

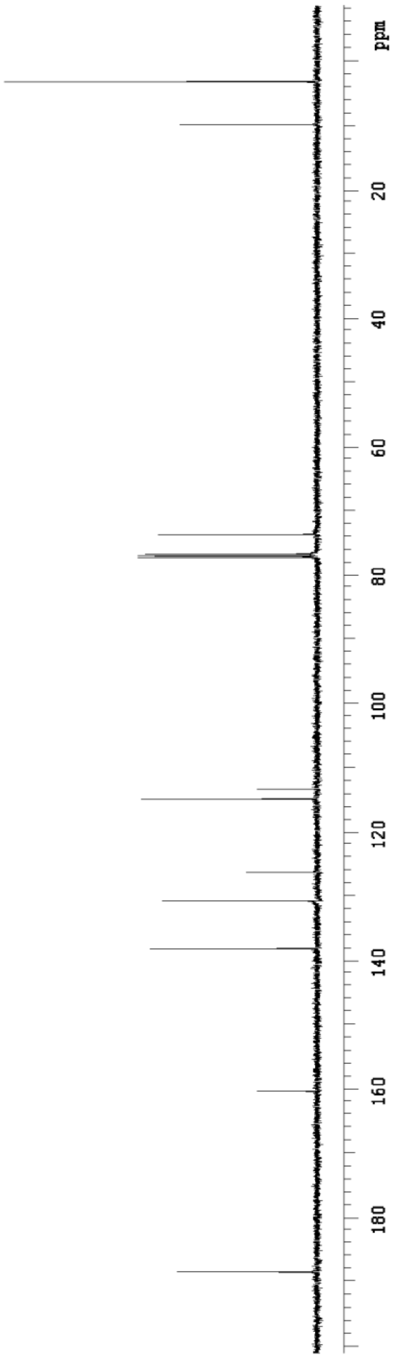

^{13}C NMR ($CDCl_3$, 23 ºC) of **S11**

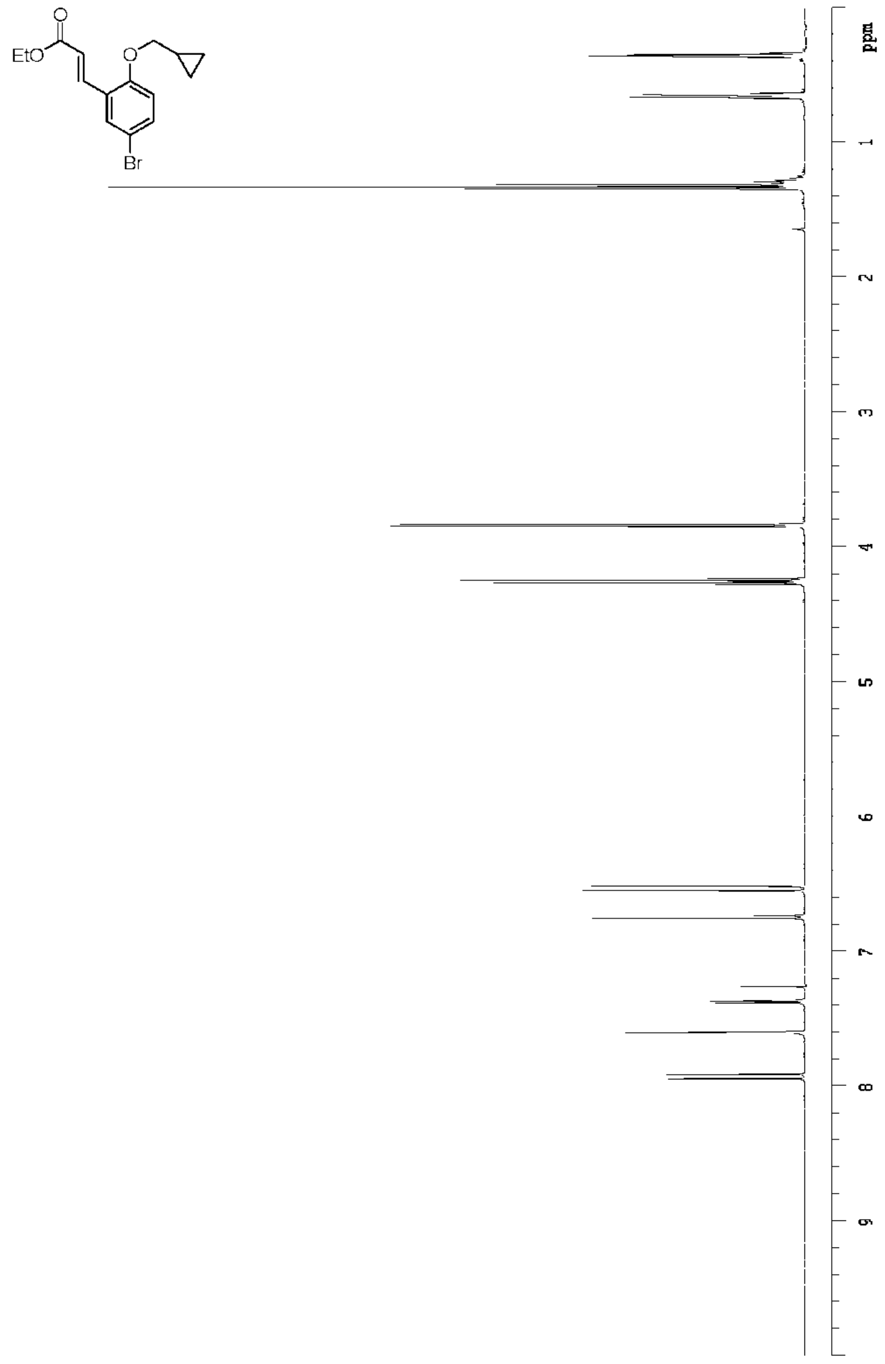

^{1}H NMR ($CDCl_3$, 23 ºC) of S**12**

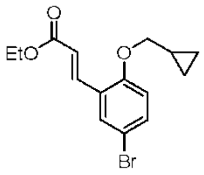

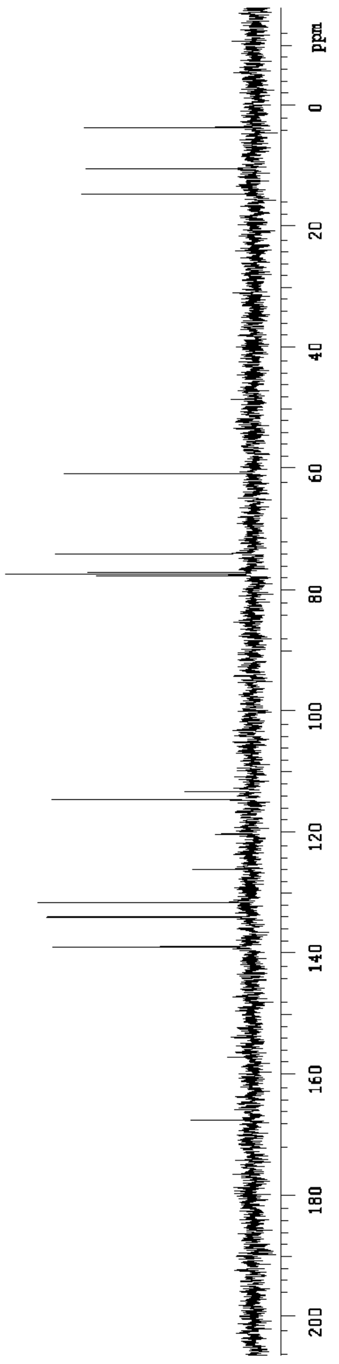

^{13}C NMR ($CDCl_3$, 23 ºC) of **S12**

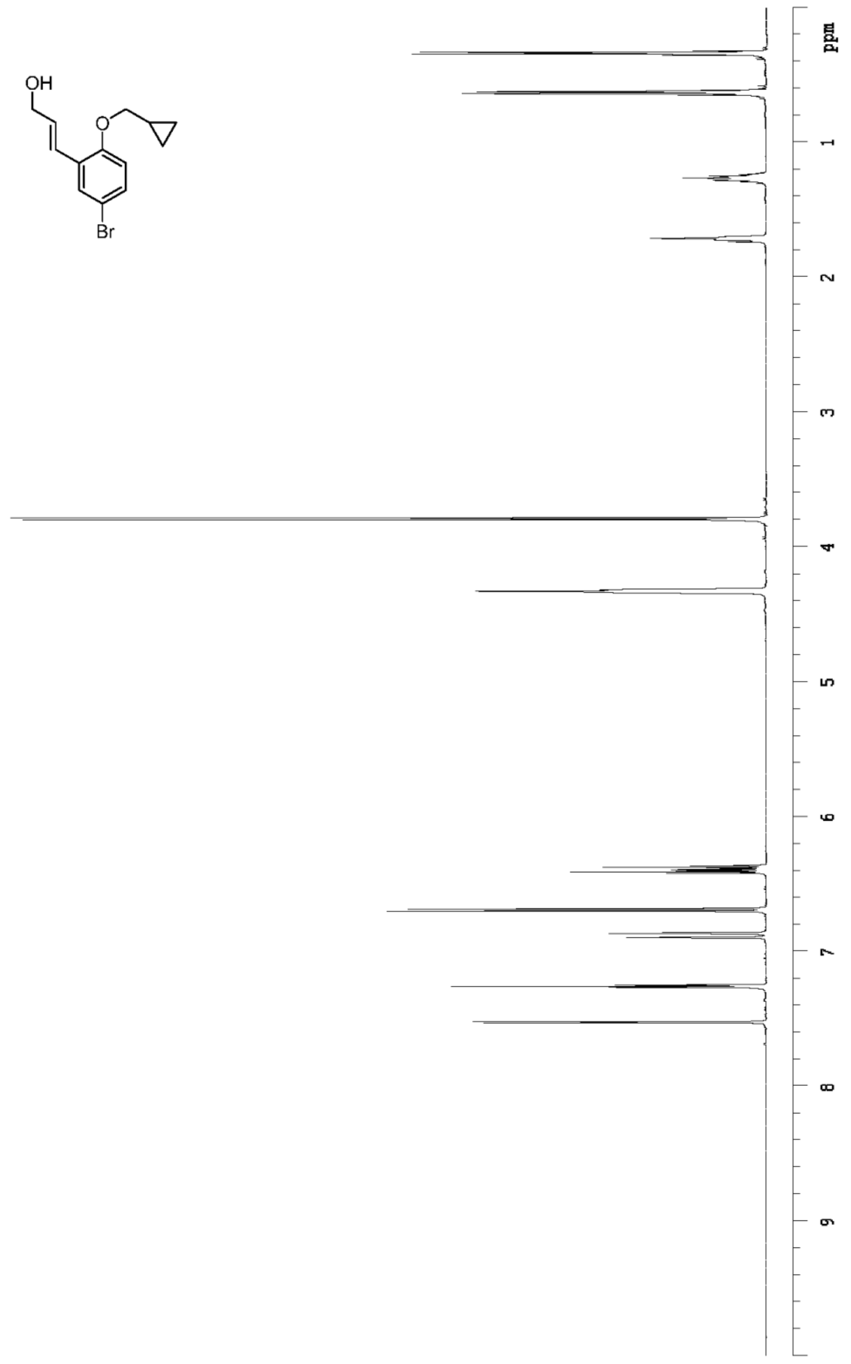

^{1}H NMR ($CDCl_3$, 23 ºC) of **16**

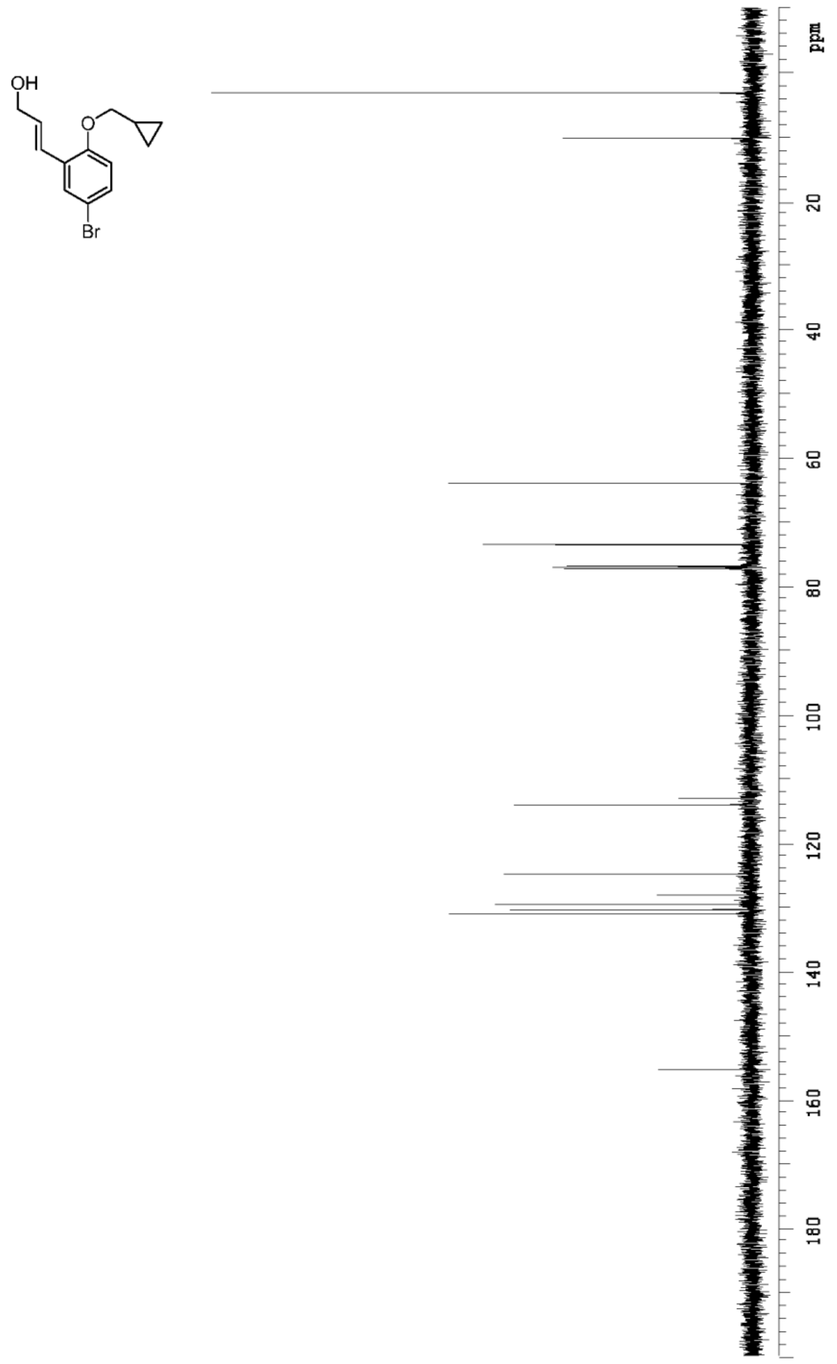

^{13}C NMR ($CDCl_3$, 23 ºC) of **16**

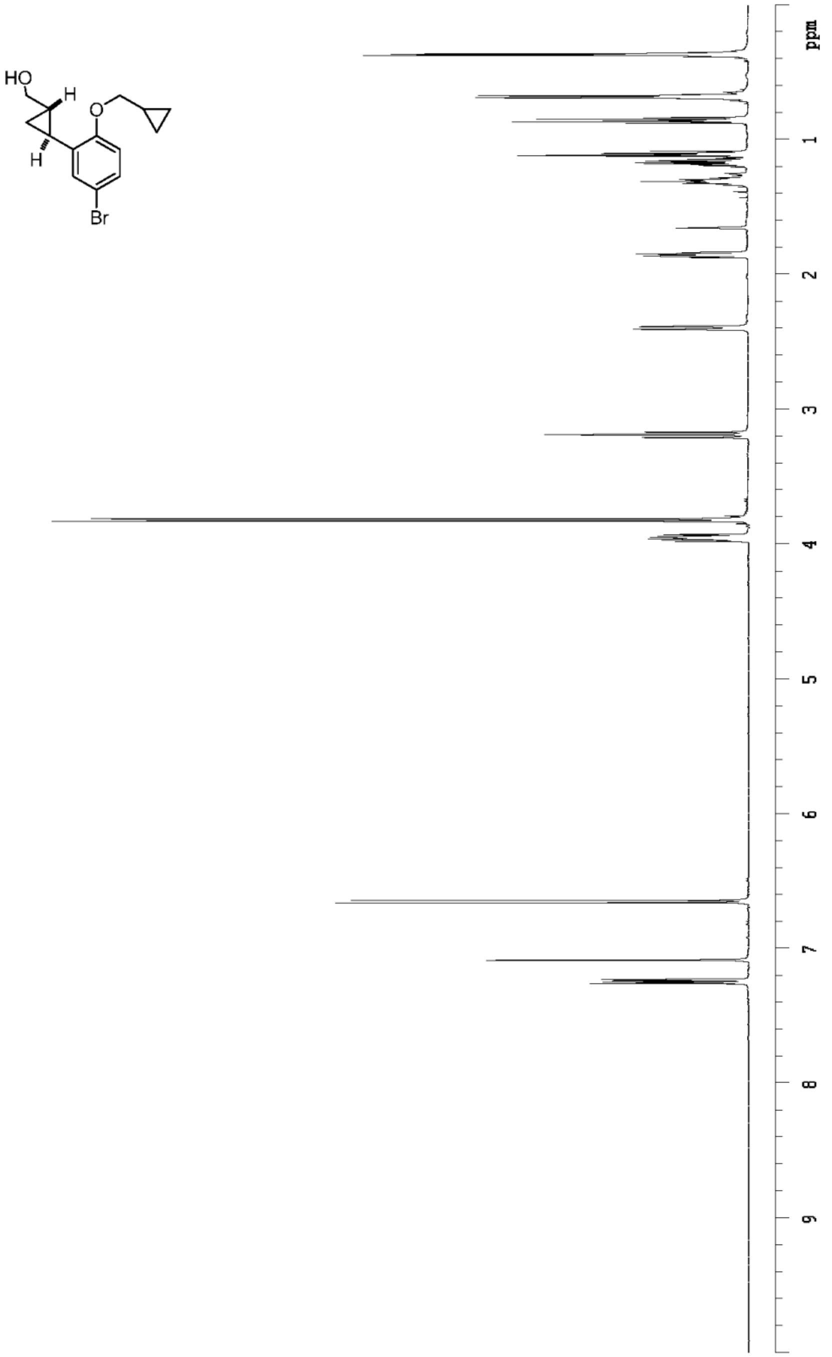

^{1}H NMR ($CDCl_3$, 23 ºC) of **17**

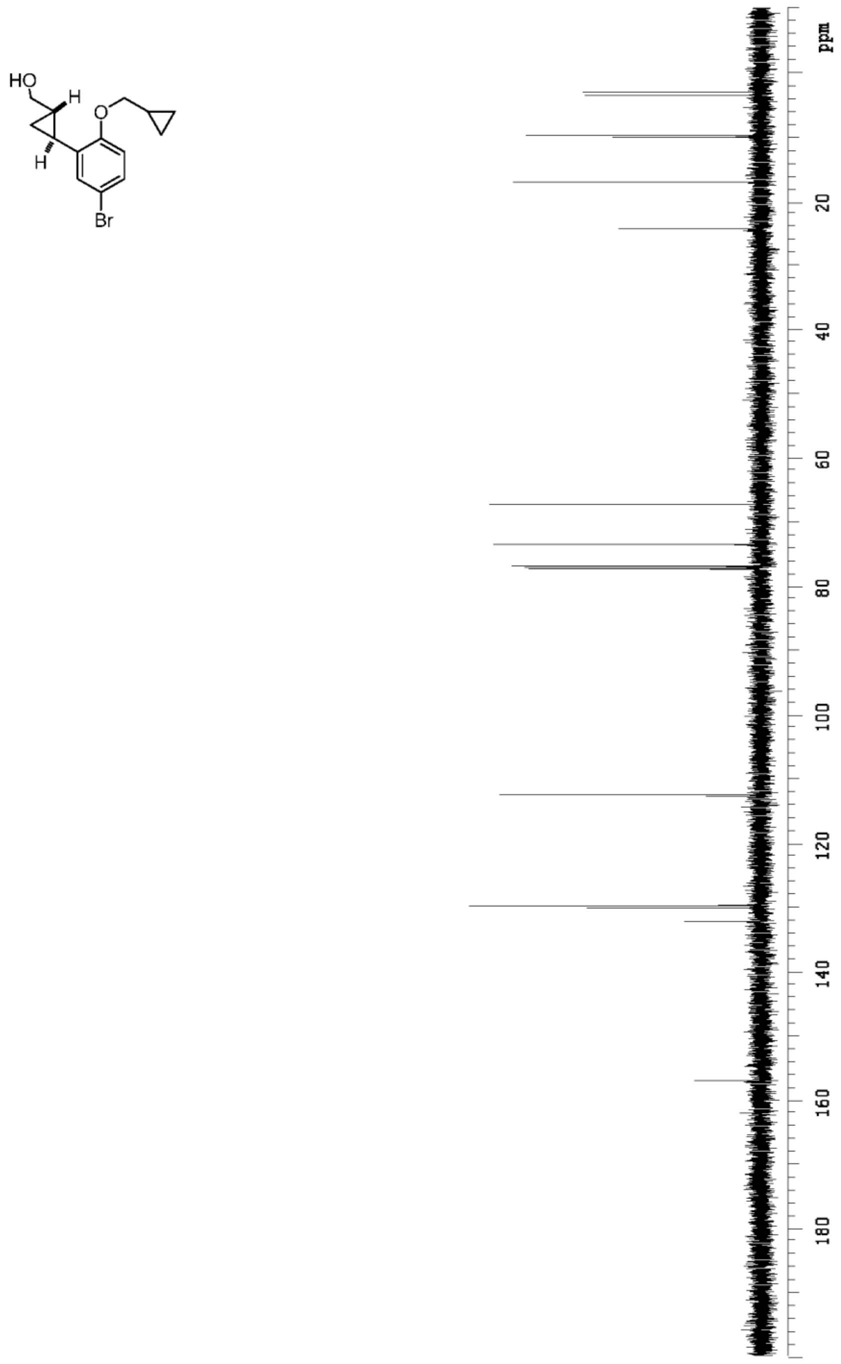

^{13}C NMR ($CDCl_3$, 23 ºC) of **17**

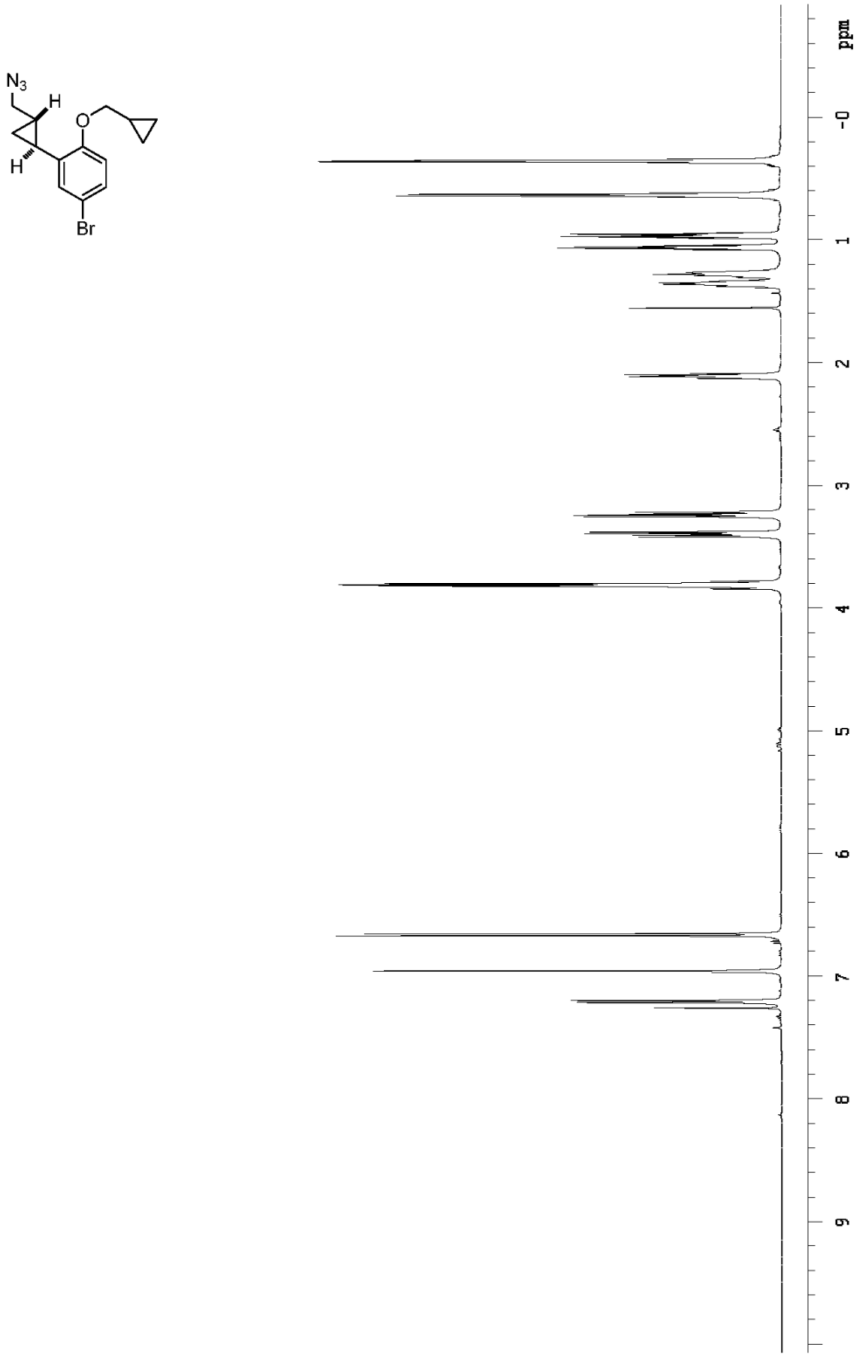

^{1}H NMR ($CDCl_3$, 23 ºC) of **S13**

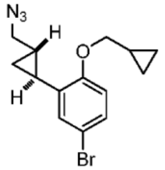

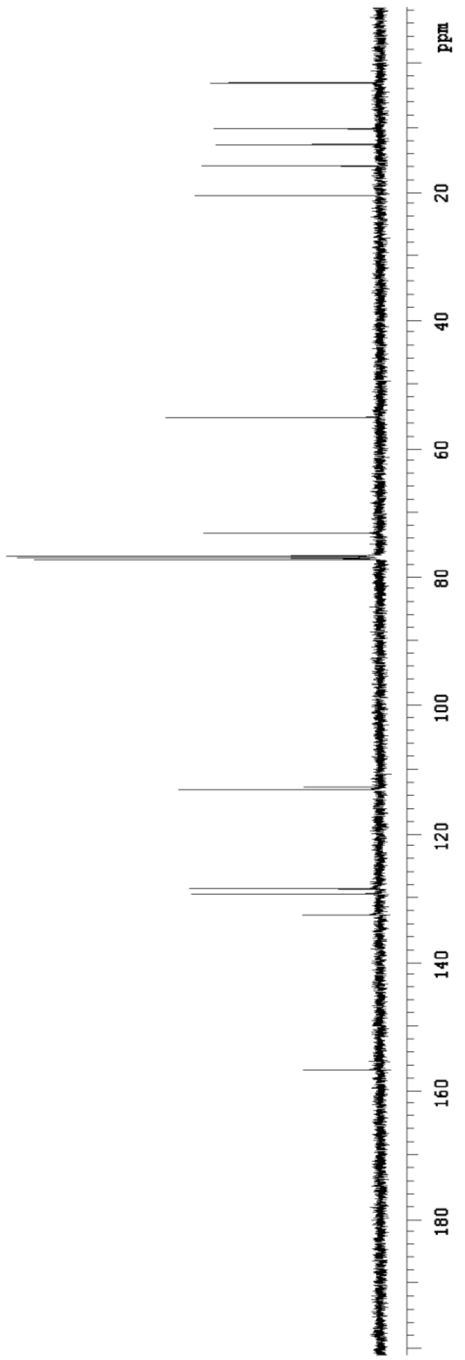

^{13}C NMR ($CDCl_3$, 23 ºC) of **S13**

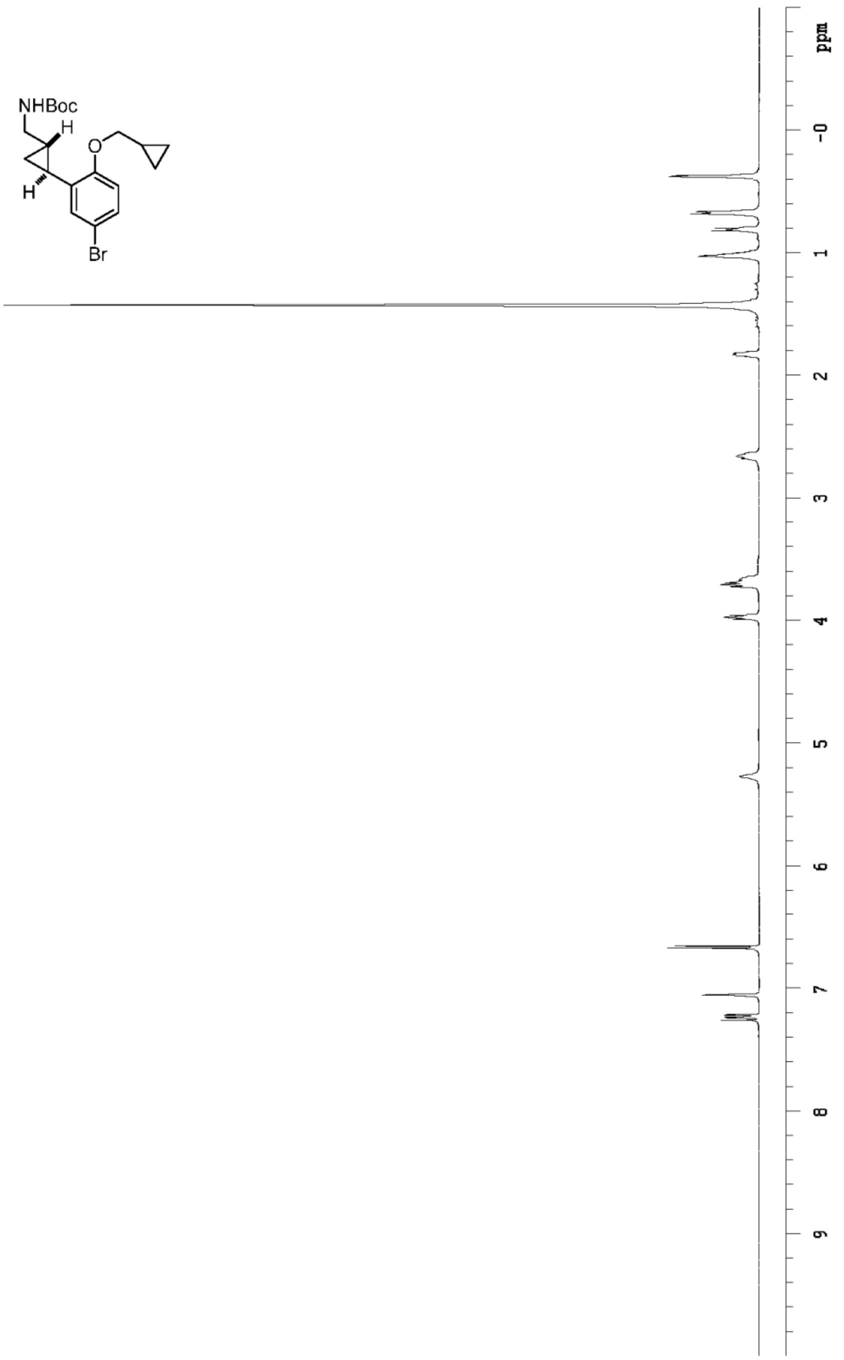

^{1}H NMR ($CDCl_3$, 23 ºC) of **18**

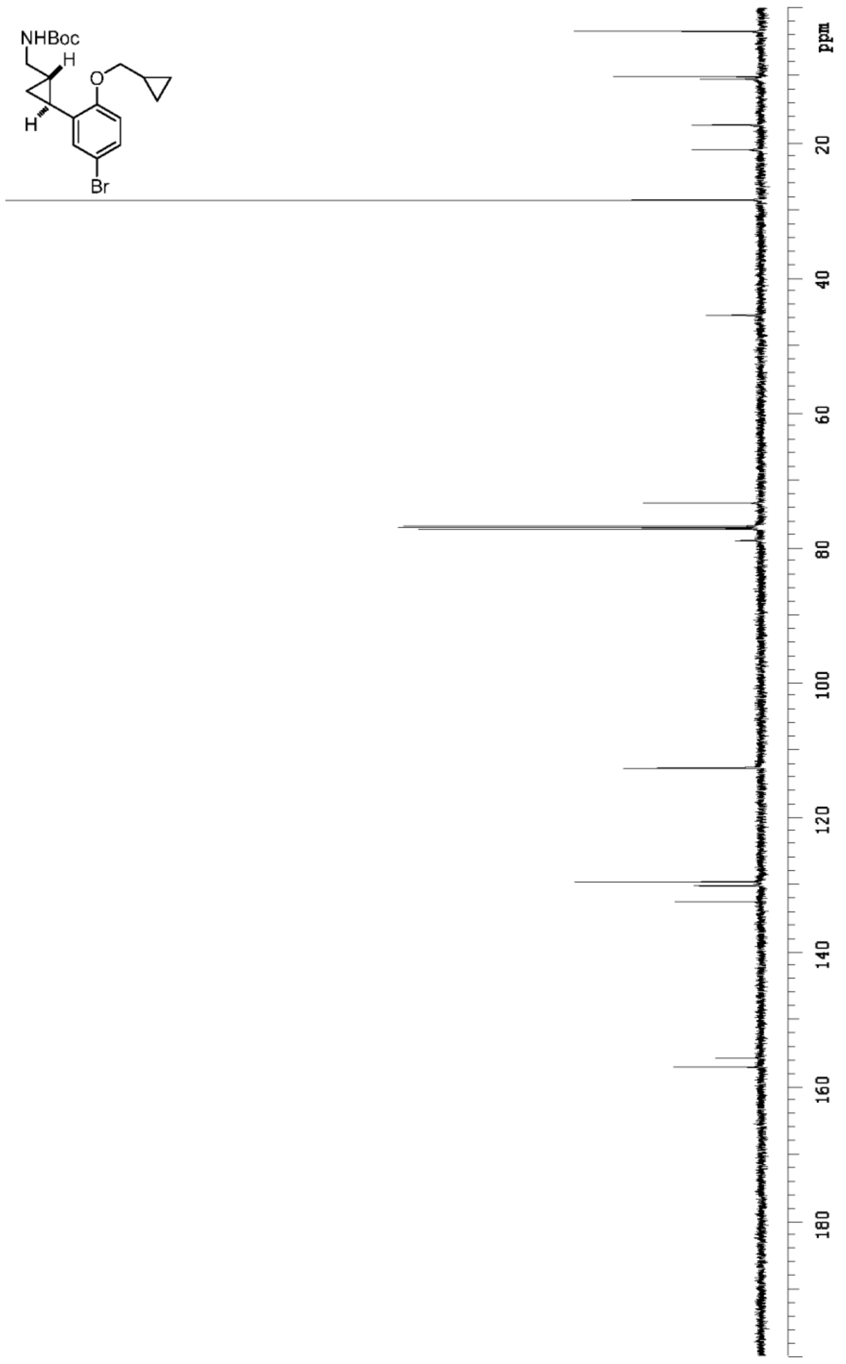

^{13}C NMR ($CDCl_3$, 23 ºC) of **18**

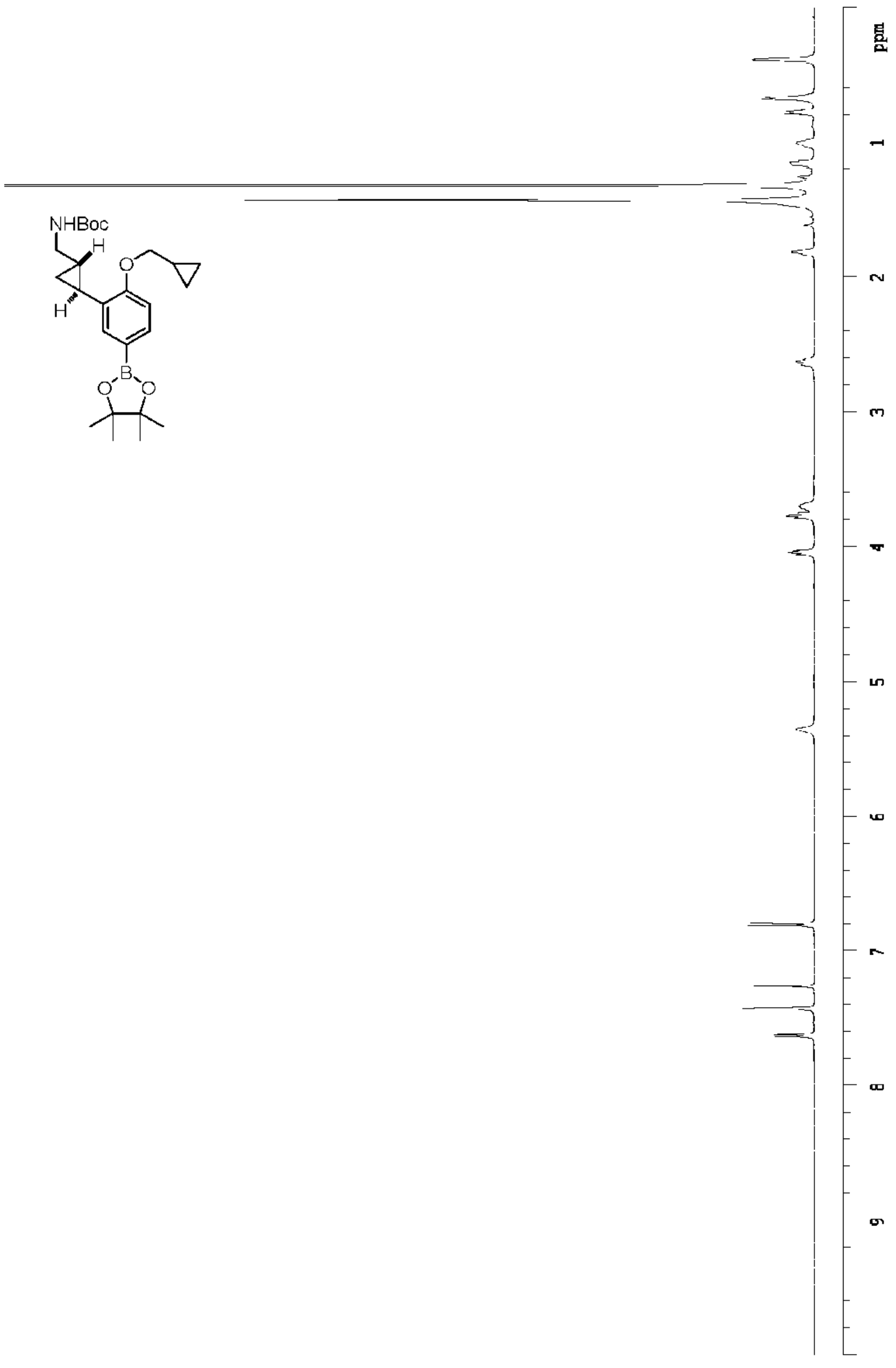

^{1}H NMR ($CDCl_3$, 23 ºC) of **19**

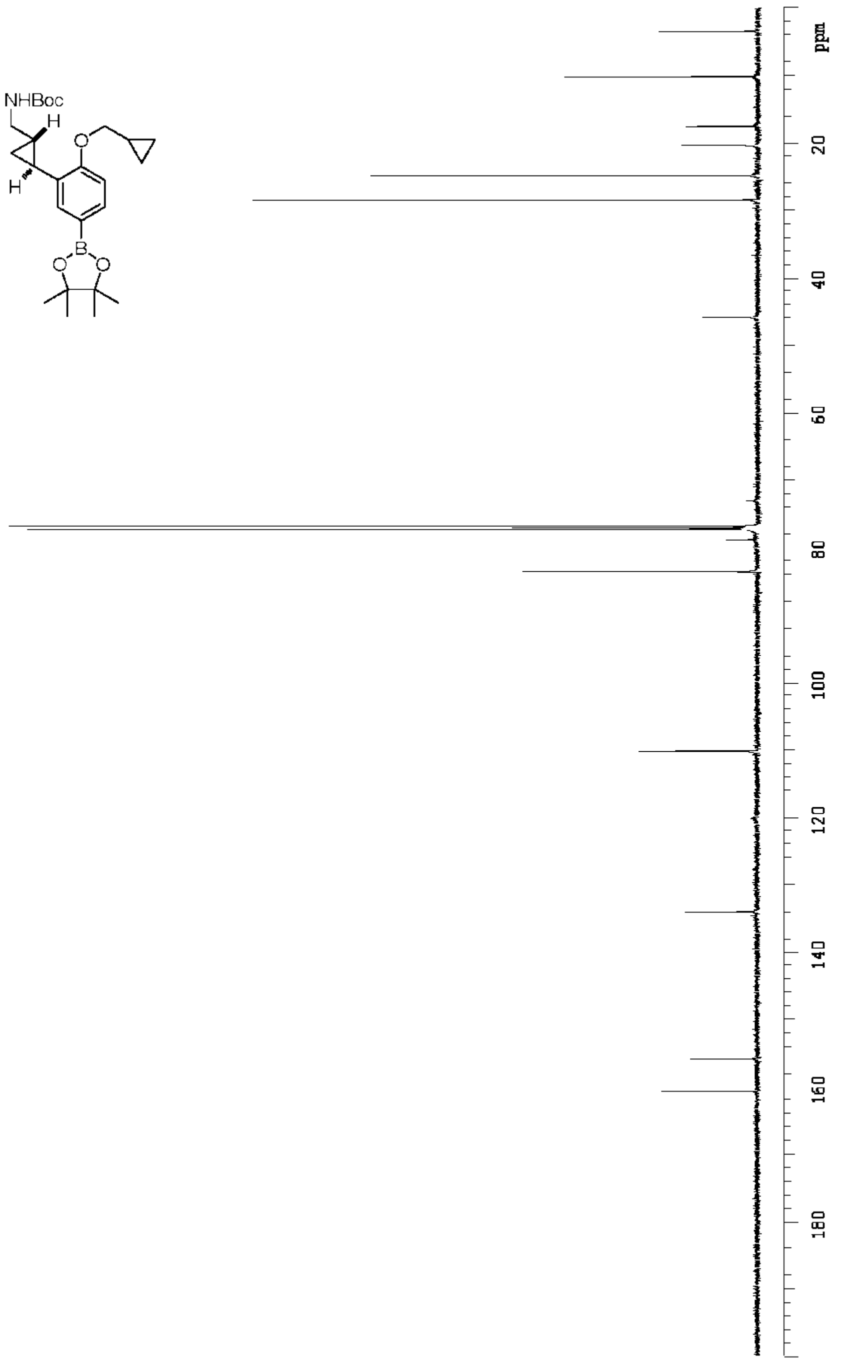

^{13}C NMR ($CDCl_3$, 23 ºC) of **19**

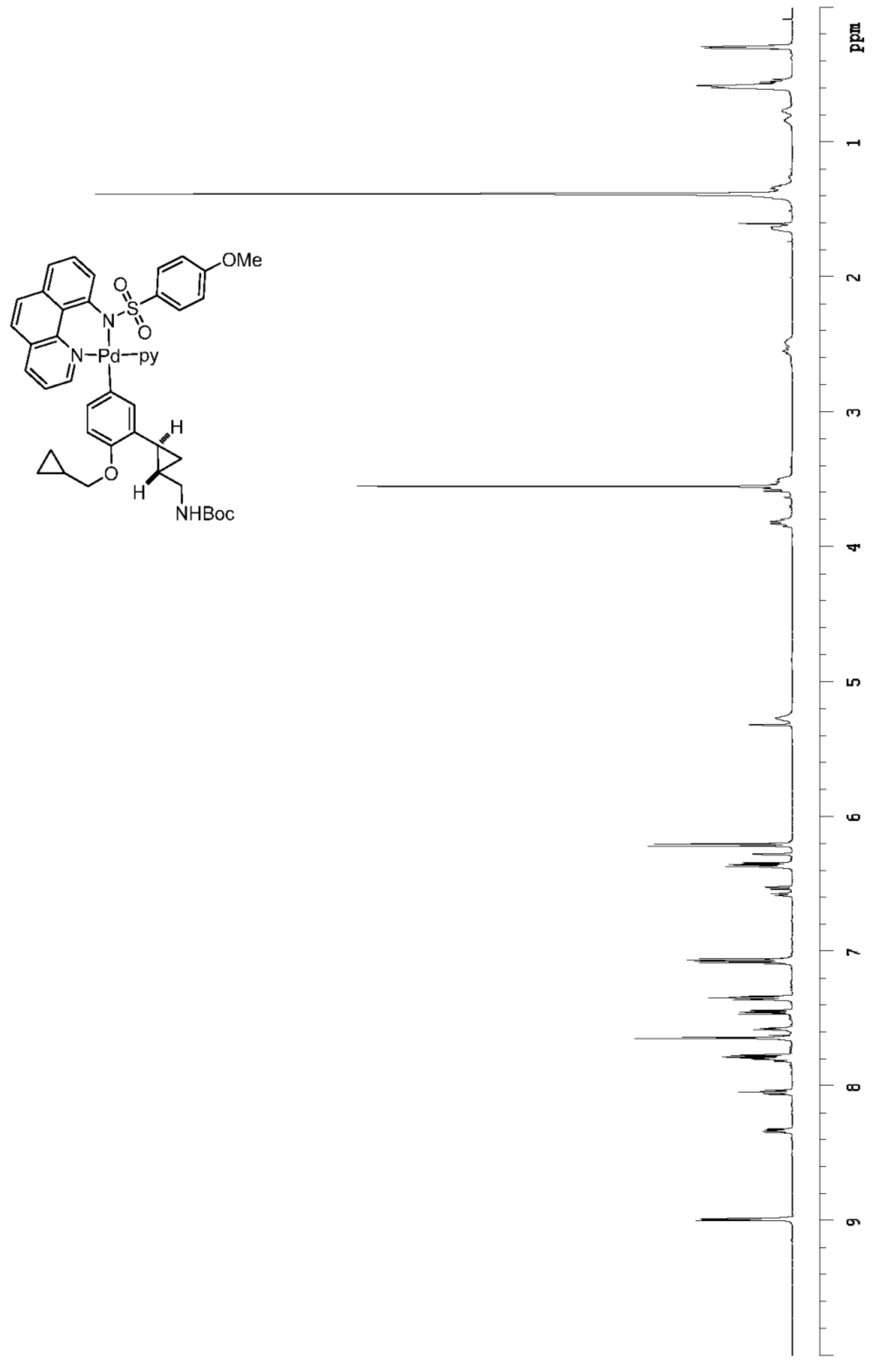

^{1}H NMR (CD_2Cl_2, 23 ºC) of **20**

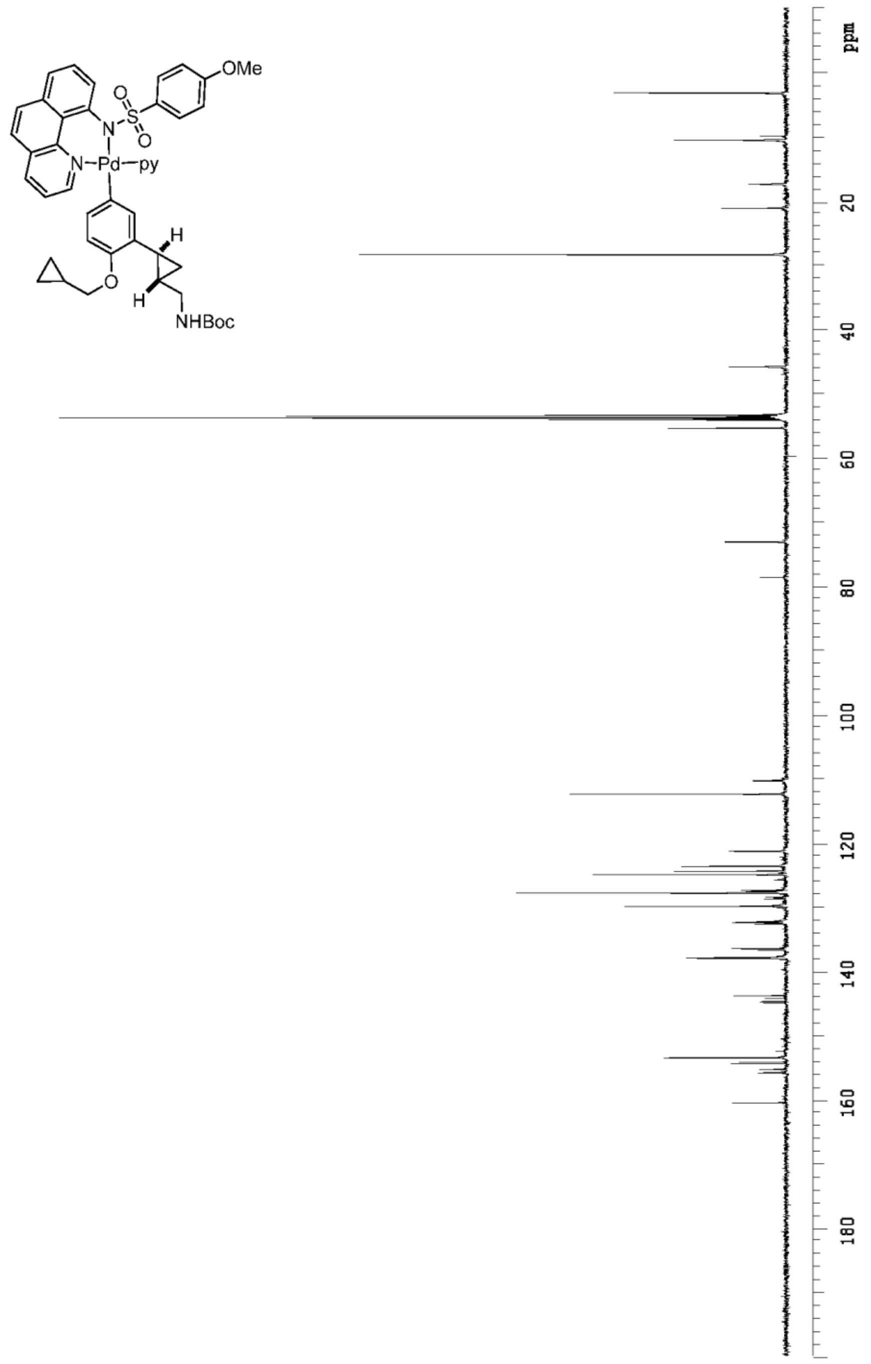

^{13}C NMR (CD_2Cl_2, 23 ºC) of **20**

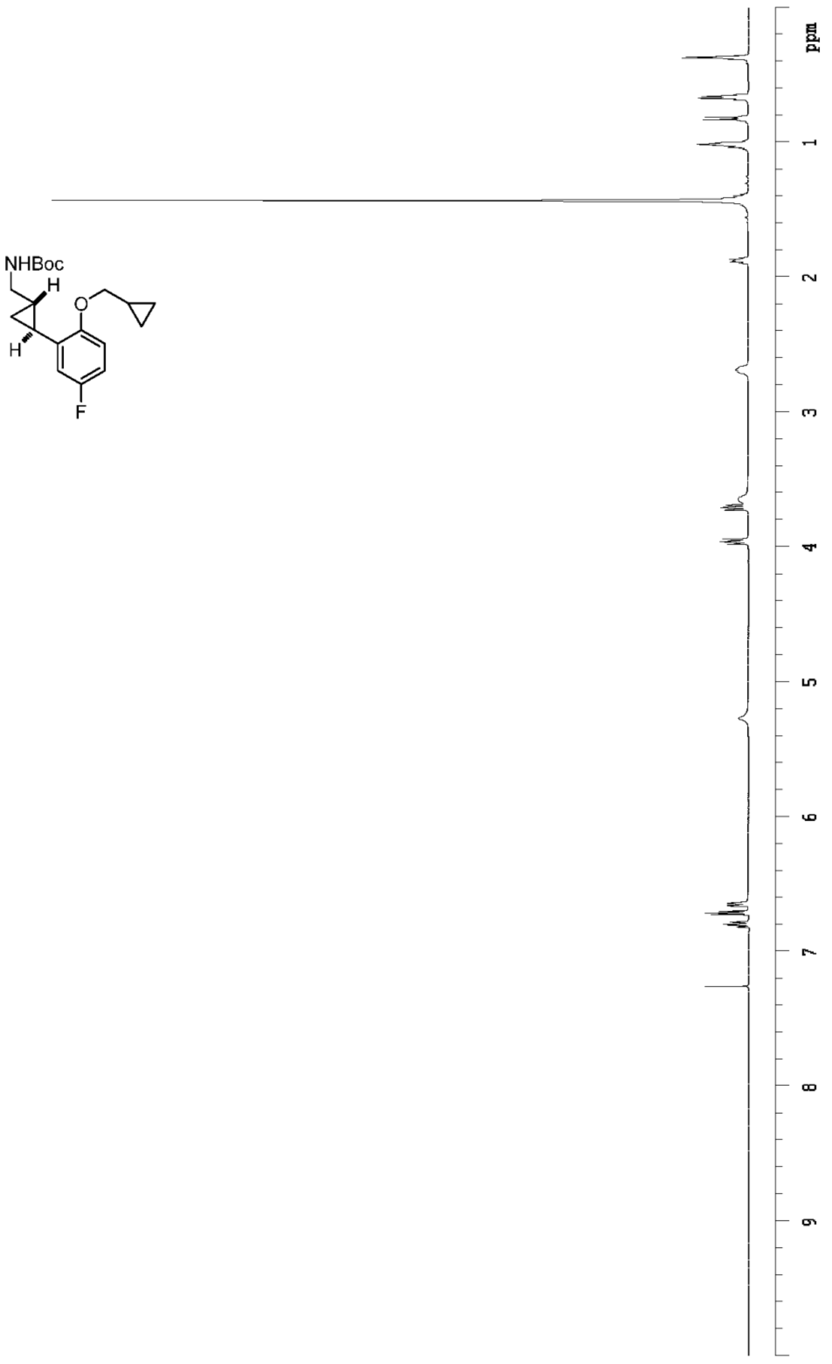

^{1}H NMR ($CDCl_3$, 23 ºC) of **22**

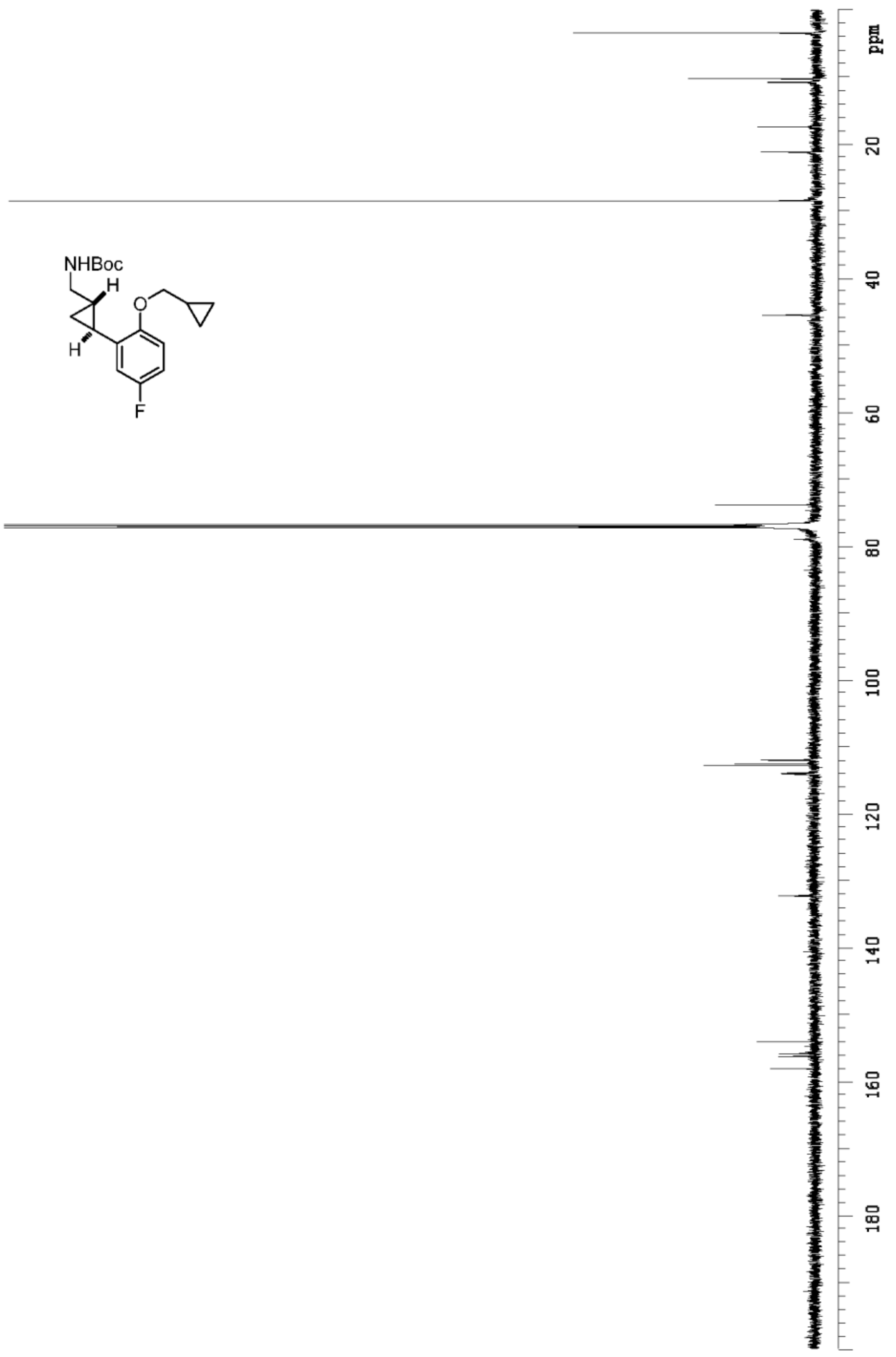

^{13}C NMR ($CDCl_3$, 23 ºC) of **22**

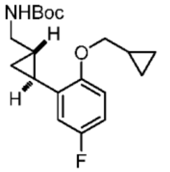

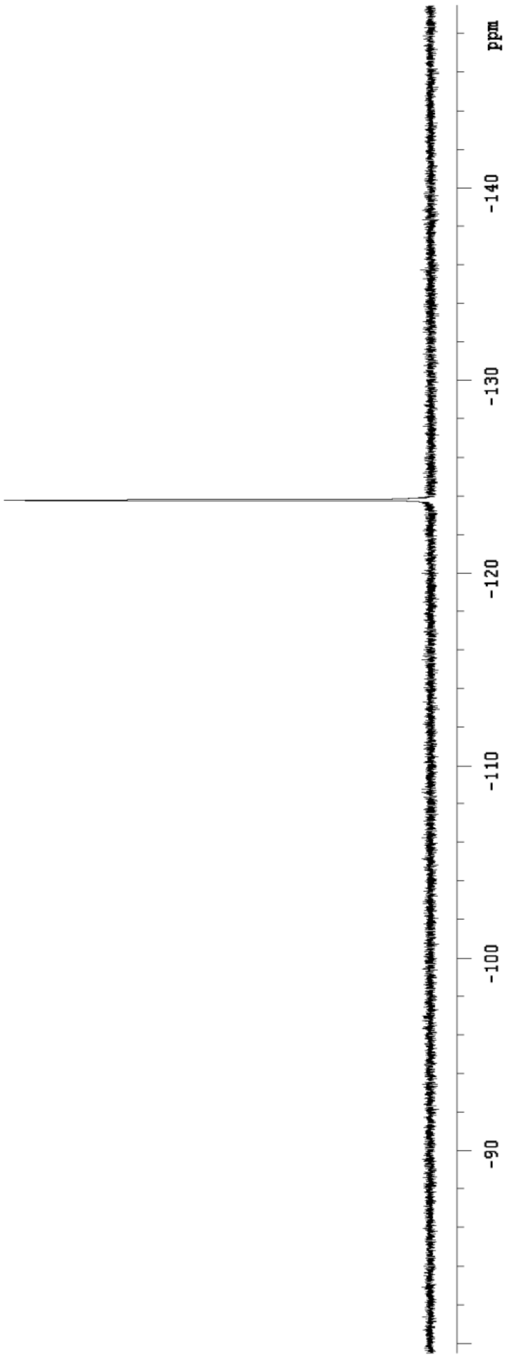

^{19}F NMR ($CDCl_3$, 23 ºC) of **22**

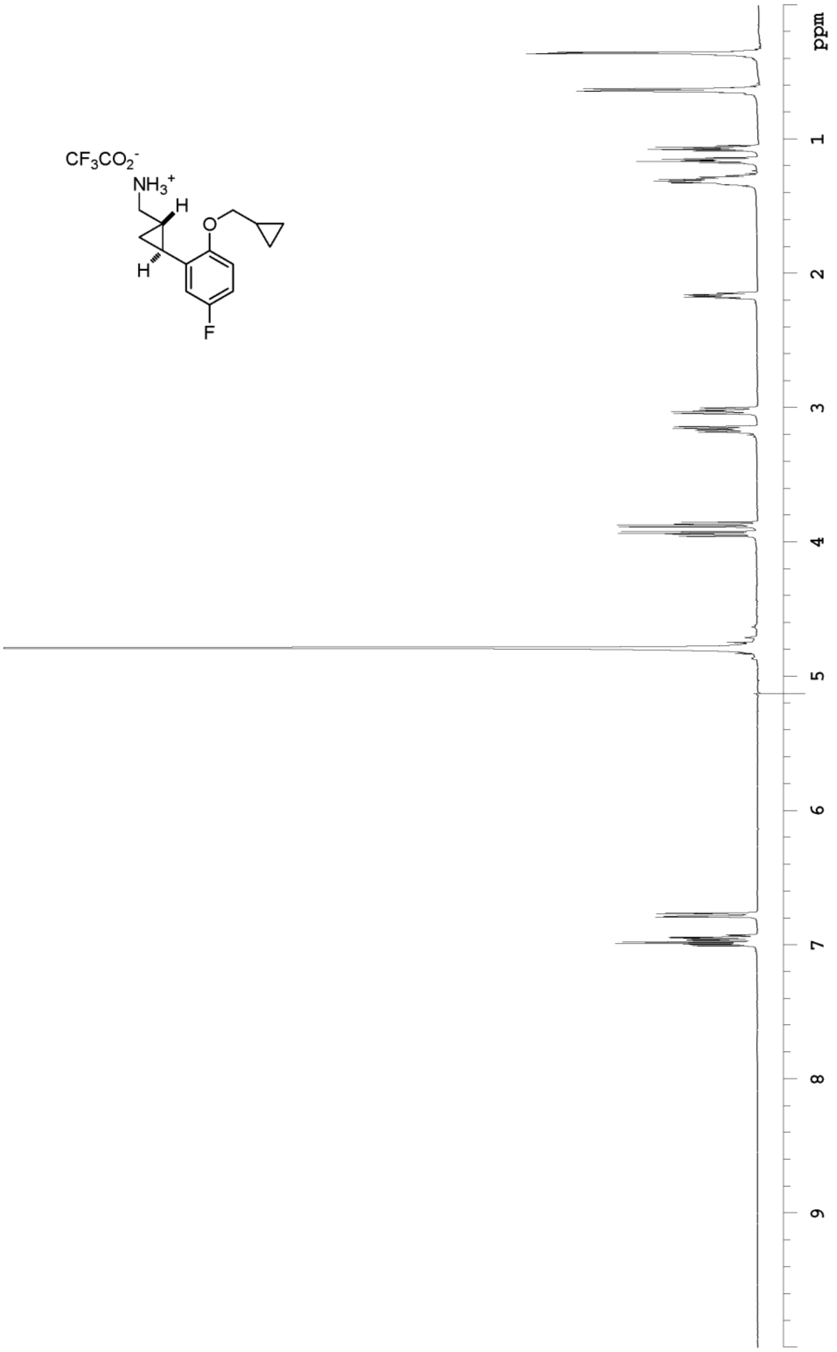

^{1}H NMR (D_2O, 23 ºC) of **2·CF_3CO_2H**

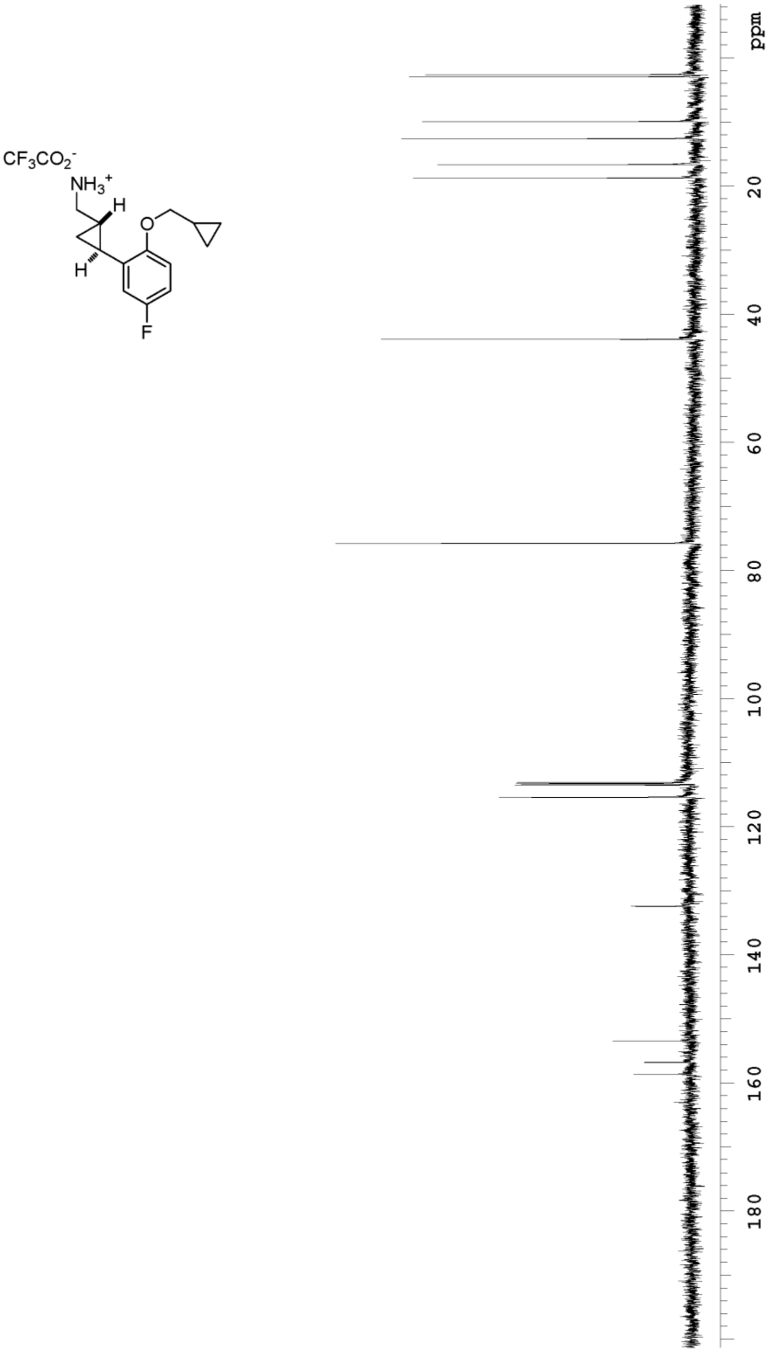

^{13}C NMR (D_2O, 23 ºC) of **2·CF_3CO_2H**

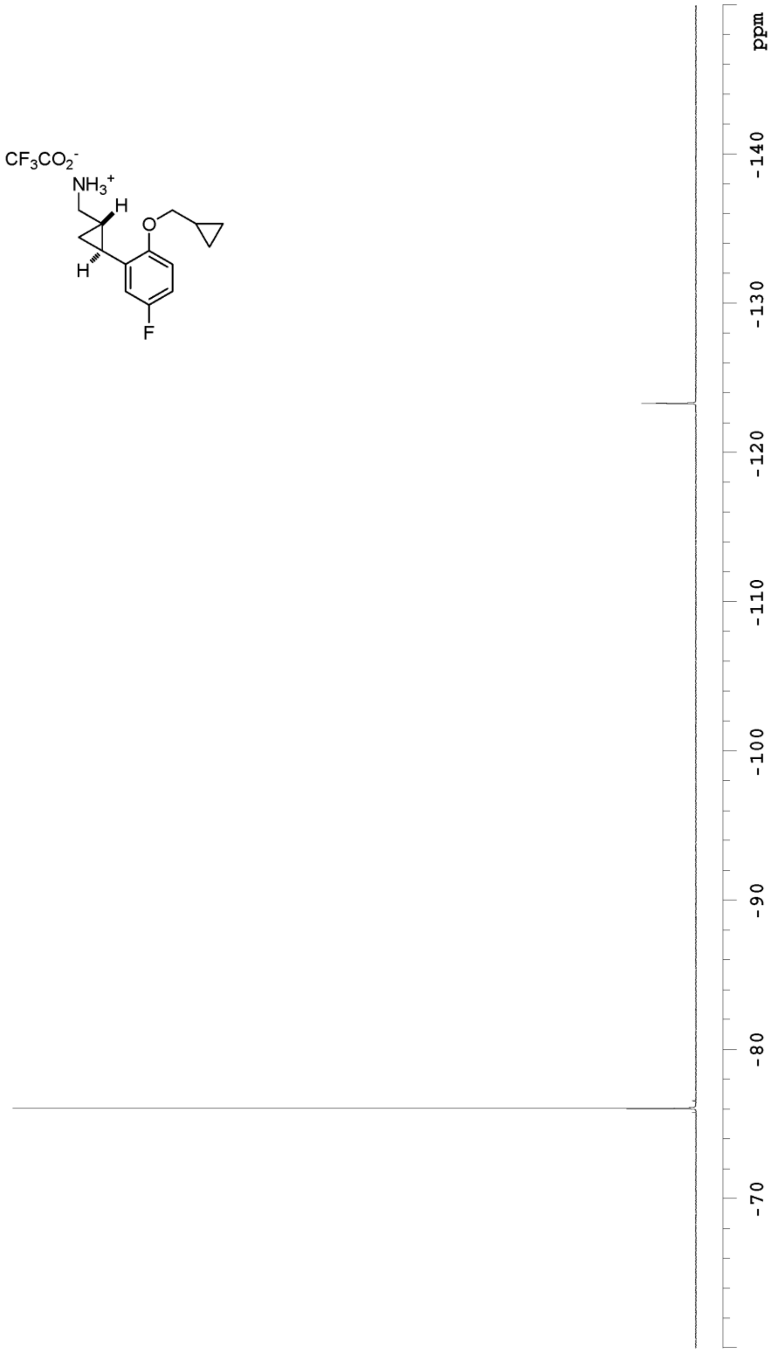

^{19}F NMR (D_2O, 23 ºC) of **2·CF$_3$CO$_2$H**

References

(1) Pangborn, A. B.; Giardello, M. A.; Grubbs, R. H.; Rosen, R. K.; Timmers, F. J. *Organometallics* 1996, *15*, 1518.

(2) Matthews, W. S.; Bares, J. E.; Bartmess, J. E.; Bordwell, F. G.; Cornforth, F. J.; Drucker, G. E.; Margolin, Z.; McCallum, R. J.; McCollum, G. J.; Vanier, N. R. *J. Am. Chem. Soc.* 1975, *97*, 7006.

(3) Fulmer, G. R.; Miller, A. J. M.; Sherden, N. H.; Gottlieb, H. E.; Nudelman, A.; Stoltz, B. M.; Bercaw, J. E.; Goldberg, K. I. *Organometallics* 2010, *29*, 2176.

(4) Dick, A. R.; Hull, K. L.; Sanford, M. S. *J. Am. Chem. Soc.* 2004, *126*, 2300.

(5) Niedenzu, K.; Niedenzu, P. M. *Inorg. Chem.* 1984, *23*, 3713.

(6) Onishi, M.; Yushichiro, O.; Sugimura, K.; Hiraki, K. *Chem. Lett.* 1976, 955.

(7) Weiss, R.; Seubert, J. *Angew. Chem., Int. Ed. Engl.* 1994, *33*, 891.

(8) Muller, P.; Baud, C.; Jacquier, Y. *Can. J. Chem.* 1998, *76*, 738.

(9) Taylor, S.; Gullick, J.; McMorn, P.; Bethell, D.; Page, P. C. B.; Hancock, F. E.; King, F.; Hutchings, G. J. *J. Chem. Soc.-Perkin Trans. 2* 2001, 1714.

(10) Hartwell, G. E.; Lawrence, R. V.; Smas, M. J. *J. Chem. Soc. Chem. Commun.* 1970, 912.

(11) Dick, A. R.; Remy, M. S.; Kampf, J. W.; Sanford, M. S. *Organometallics* 2007, *26*, 1365.

(12) Hughes, G.; Kimura, M.; Buchwald, S. L. *J. Am. Chem. Soc.* 2003, *125*, 11253.

(13) Charette, A. B.; Juteau, H.; Lebel, H.; Molinaro, C. *J. Am. Chem. Soc.* 1998, *120*, 11943.
